# Supplementary material for: One-pot synthesis of cyclic-aminotropiminium carboxylate derivatives with DNA binding and anticancer properties
Source: Commun Chem. 2022 Dec 27;5:179. doi: 10.1038/s42004-022-00798-x (PMC9814901; doi:10.1038/s42004-022-00798-x)
Supplement: Supplementary file 2 — Supplemental Information [file 42004_2022_798_MOESM2_ESM.pdf]

Supplemental Material

One-Pot synthesis of cyclic-aminotropiminium carboxylate derivatives with DNA binding and anticancer properties

Bibhuti Bhusana Palai[a,c,#], Saket Awadhesbhai Patel[b,c,#], Nagendra K. Sharma*[,a.c], Manjusha Dixit*[,b,c]

[#]Authors equally contributed

*Corresponding Authors

[a]School of Chemical Sciences, National Institute of Science Education and Research (NISER) Bhubaneswar, PO: Jatani-752050, Odisha, India, Phone no. +91-674-249-4141; E-mail: nagendra@niser.ac.in

[b]School of Biological Sciences, National Institute of Science Education and Research (NISER) Bhubaneswar, PO: Jatani-752050, Odisha, India, Phone no. +91-674-249-4195; E-mail: manjusha@niser.ac.in

[c]Homi Bhabha National Institute, Training School Complex, Anushaktinagar, Mumbai 400094, India

Contents Page No.

1. **Supplementary Methods** S3
2. **General Information** S3
3. **Experimental procedures** S3
4. **Analytical Data** S5
5. **Crystal structures and data** S15
6. **Time dependent ^{1}H-NMR spectra in CD_3CN** S25
7. Time dependent Mass analysis of cATC formation S27
8. UV-Visible and Fluorescence Spectroscopic Studies formation of cATC-derivatives S29
9. Computational Studies of proposed reaction mechanism S30
10. **Photophysical studies of cATC derivatives** S33
11. **HOMO-LUMO Calculation of cATC derivatives** S38
12. **Binding studies of cATC derivatives with DNA** S42
13. **Cell viability studies of cATC derivatives** S50
14. **Supplementary References** S61

1. **Supplementary Methods**
2. **General Information**

*Material and Instrumentation***:** All required materials were obtained from commercial and used without purification. Anhydrous Acetonitrile was freshly prepared by distilling over calcium hydride. Reactions were monitored by thin-layer chromatography, visualized by UV and Ninhydrin. Column chromatography was performed in 230-400 and 100-200 mesh silica. Mass spectra were obtained from Bruker MicrOTOF-Q II Spectrometer.NMR spectra were recorded on Bruker AV-700MHz and Bruker AV-400 (^{1}H:400MHz ,^{13}C:100.6 MHz).^{1}H and ^{13}C NMR chemical shift were recorded in ppm downfield from tetramethylsilane, splitting patterns are abbreviated as s, singlet; d, doublet; dd, doublet of doublet; t, triplet; q, quartet; dq, doublet of quartet; m, multiplet. The crystal data of two compounds were collected on a Rigaku Oxford diffractometer at 293 K respectively. Absorption spectra were obtained using Jasco V-730 spectrometer. Fluorescence spectra were obtained from Perkin-Elmer LS-55 using a Xenon lamp. All spectroscopic measurements were carried out with spectroscopic grade, degassed solvents, and at 20°C. Relative fluorescence quantum yields were determined by comparing with Cumarine quantum yield in methanol.

3. **Experimental procedures**

(2.1) *Synthesis of Trag monomer and peptide***:** previously we reported the synthesis of Troponyl ester and amide synthesis; Here we followed the same procedure for synthesis of monomer and peptides.[1, 2] In this study we are reporting the Trapping ketene intermediate [2+2] cycloaddition reaction under the mild acidic condition to form a novel Troponyl six-member cyclic with novel pyrazine derivatives.

(2.2) *General Procedure of Schiff Base Synthesis*: Schiff bases were prepared by condensation of aldehyde (1 eq.) with amines (1 eq.). Different aldehydes (1 eq.) and amines (1 eq.) were

dissolved in MeOH, anhydrous Na_2SO_4 was added as a drying agent and the mixture was refluxed for 6 h. The completion of the reaction was monitored by TLC. After complete conversion, the reaction mixture was filtered using a glass funnel. Then the filtrate was dried under reduced pressure and the crude products were used for the next step of synthesis without any purification.[3]

(2.3) *Trapping Ketene Intermediate via [2+2] Cycloaddition reaction with Imine*: The *N*-troponyl-*N*-phenylethyl glycinate (*tr-pheneg*) ester (**2**) (1 eq.) was taken in a round bottom flask under Argon atmosphere in dry Acetonitrile and treated with 5% TFA 0 °C after 5 min stirring Imine (**5**) (4.0 equivalent) was added and the reaction mixtures allowed to stir for 6-12 h at rt. The reaction mixture was monitored by TLC, after completion of the reaction, the reaction mixture was concentrated under reduced pressure. The concentrated reaction mixture was dissolved in DCM (30ml) and washed with water trice (3*30ml) followed by saturated sodium bicarbonate (20ml) and brine (20ml). The organic layers were combined, dried over sodium sulphate, and concentrated under vacuum. The crude reaction mixture was purified by column chromatography using DCM:MeOH (95:5) desired product was obtained. The obtained product was well characterized by $^{1}H/^{13}C$-NMR and HRMS. The characterizing data of all newly synthesized compounds are provided below.

4. **Analytical Data**

N-troponyl-*N*-phenylethyl glycinate (tr-pheneg) ester (**2**): Synthesize by following the reported procedure.[1, 2]

^{1}H NMR (400 MHz, CD_3CN) δ 7.33 (q, *J* = 6.4 Hz, 4H), 7.28 – 7.21 (m, 1H), 7.16 – 7.05 (m, 2H), 6.76 (dd, *J* = 20.8, 11.3 Hz, 2H), 6.66 – 6.59 (m, 1H), 4.31 (s, 2H), 4.15 (q, *J* = 7.1 Hz, 2H), 3.73 – 3.65 (m, 2H), 3.00 (dd, *J* = 8.9, 7.0 Hz, 2H), 1.24 (t, *J* = 7.1 Hz, 3H).

N-benzylidene-phenylmethaneamine (**5a**)

^{1}H NMR (400 MHz, $CDCl_3$) δ 8.40 – 8.29 (m, 1H), 7.89 – 7.79 (m, 3H), 7.50 (ddd, *J* = 9.8, 5.7, 1.8 Hz, 1H), 7.43 (s, 2H), 7.41 – 7.36 (m, 3H), 7.34 – 7.27 (m, 1H), 4.82 (d, *J* = 5.7 Hz, 2H). Mass (ESI-TOF) m/z: $[M+H]^+$ found 196.1293.

Benzyl phenethyl phenyl *cyclic*-aminotroponiminium carboxylate (**6a**)

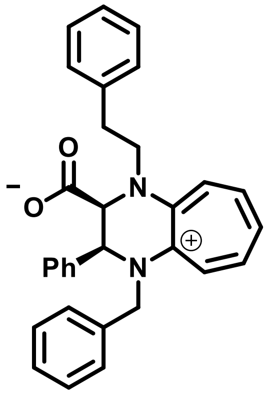

6a

The *cyclic*-aminotroponiminium compound (**6a**) was synthesized according to general procedures **(2.3)**. The crude reaction was purified by column chromatography using silica gel (100-200 mesh size), giving a yellow viscous liquid 60mg (**91%** yield in 4% MeOH/DCM)

M.P 135–140 °C. FT-IR (KBr plate) ν (cm^{-1}) = 3032, 2925, 1744, 1699, 1598, 1522, 1493, 1477, 1464, 1450, 1435, 1402, 1343, 1302, 1264, 1222, 1210, 1191, 1130. ^{1}H NMR (400 MHz, $CDCl_3$) δ 7.45 – 7.13 (m, 16H), 6.89-6.82 (dd, *J* = 12.0, 8.3 Hz, 4H), 6.72-6.769 (d, *J* = 11.5 Hz, 1H), 5.95 (s, 1H), 4.78 – 4.58 (m, 3H), 3.86 (dt, *J* = 15.6, 8.0 Hz, 1H), 3.69 (ddd, *J* = 14.4, 9.2, 5.2 Hz, 1H), 3.44 (s, 1H), 2.51 (dt, *J* = 16.2, 8.3 Hz, 1H), 2.35 (ddd, *J* = 14.1, 8.9, 5.4 Hz, 1H). ^{13}C NMR (101 MHz, $CDCl_3$) δ 167.39 , 152.07, 151.00, 139.28, 139.10 , 138.21, 136.95, 132.44, 129.61, 129.45, 128.85, 128.70, 128.54, 128.25,

127.08, 126.92, 126.39, 125.97, 116.68, 116.54, 71.38, 67.63, 56.86, 55.71, 50.61, 31.31. HRMS (ESI-TOF) m/z :[M+H]$^+$ Calcd. For $C_{38}H_{28}N_2O_2$ 461.2224, found 461.2200.

Benzyl-dimethoxyphenyl phenethyl *cyclic*-aminotroponiminium carboxylate **(6b)**

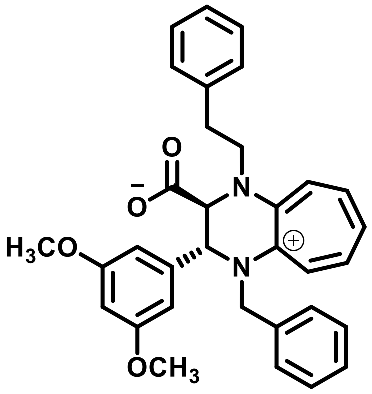

6b

The *cyclic*-aminotroponiminium compound (**6b**) was synthesized according to general procedures **(2.3)**. The crude reaction was purified by column chromatography using silica gel (100-200 mesh size), giving a yellow viscous liquid 150mg (**68%** yield in 3% MeOH/DCM). FT-IR (KBr plate) ν (cm^{-1}) = 3051, 2928, 1596, 1520, 1494, 1454, 1430, 1343, 1264, 1221, 1204, 1158. ^{1}H NMR (400 MHz, $CDCl_3$) δ 7.43 (dd, *J* = 13.9, 7.3 Hz, 2H), 7.32-7.20 (m, 5H), 6.90 – 6.82 (m, 3H), 6.63 (d, *J* = 11.5 Hz, 1H), 6.46 (s, 1H), 6.32 (d, *J* = 1.8 Hz, 1H), 5.85 (s, 1H), 4.76 – 4.54 (m, 1H), 3.95 – 3.77 (m, 5H), 3.48 (s, 1H), 2.71 – 2.53 (m, 1H). ^{13}C NMR (176 MHz, $CDCl_3$) δ 167.06, 161.66, 150.96, 140.78, 139.15, 132.26, 129.67, 128.91, 128.27, 127.02, 126.21, 116.51, 104.23, 99.90, 71.03, 67.61, 56.73, 55.98, 55.69, 55.55, 53.45, 31.73. HRMS (ESI-TOF) m/z: [M+H]$^+$ Calcd. For $C_{32}H_{30}N_2O_4$ 507.2278, found 507.2269.

Nitrophenyl diphenethyl *cyclic*-aminotroponiminium carboxylate (**6c**)

The *cyclic*-aminotroponiminium compound (**6c**) was synthesized according to general procedures **(2.3)**. The crude reaction was purified by column chromatography using silica gel (230-400 mesh size), giving a black viscous liquid 100mg (**75%** yield in 6% MeOH/DCM).100mg (75% yield) of the pure product was isolated as a black viscous liquid.

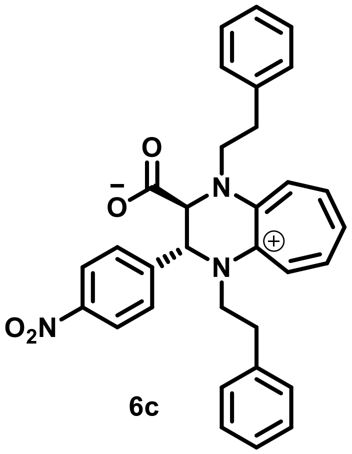

6c

M.P 125–130 °C. FT-IR (KBr plate) ν (cm^{-1}) = 3028, 2948, 1632, 1594, 1515, 1492, 1466, 1453, 1408, 1329, 1275, 1239, 1210, 1196, 1152, 1108, 1087, 1068. ^{1}H NMR (700 MHz, $CDCl_3$) δ 8.22 (d, *J* = 8.2 Hz, 2H), 7.48 (t, *J* = 10.1 Hz, 1H), 7.36 (t, *J* = 10.1 Hz, 1H), 7.32 (d, *J* = 8.2 Hz, 2H), 7.27 (d, *J* = 3.8 Hz, 4H), 7.23 – 7.13 (m, 4H), 7.00 – 6.93 (m, 2H), 6.82 (d, *J* = 3.0 Hz, 2H), 6.73 (d, *J* = 11.3 Hz, 1H), 6.16 (s, 1H), 4.59 (s, 1H), 3.92 – 3.74 (m, 3H), 3.48 (dd, *J* = 17.1, 7.3 Hz, 1H), 3.26 (dd, *J* = 16.2, 7.1 Hz, 1H), 3.21 – 3.11 (m, 1H), 2.61 – 2.53 (m, 1H), 2.52 – 2.44 (m, 1H). ^{13}C NMR (176 MHz, $CDCl_3$) δ 165.97, 151.49, 149.84, 147.88, 145.18, 139.57, 139.39, 136.99, 136.55, 128.95, 128.88, 128.86, 128.30, 127.50, 127.09, 127.05, 126.92, 124.59, 116.53, 115.94, 70.97, 66.67, 55.67, 54.15, 31.53, 31.27. HRMS (ESI-TOF) m/z: $[M+H]^+$ Calcd. For $C_{32}H_{30}N_2O_4$ 520.2231, found 520.2259.

Benzyl nitrophenyl phenethyl *cyclic*-aminotroponiminium carboxylate **(6d)**

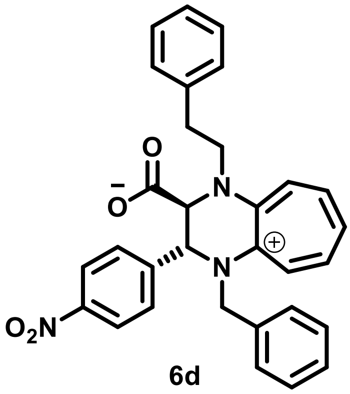

6d

The *cyclic*-aminotroponiminium compound (**6d**) was synthesized according to general procedures **(2.3)**. 170mg (60%) of the pure product was obtained as a yellow viscous. FT-IR (KBr plate) ν (cm^{-1}) = 3063, 2924, 2855, 1673, 1519, 1495, 1473, 1432, 1398, 1346, 1185, 1136. ^{1}H NMR (400 MHz, $CDCl_3$) δ 8.24 (t, *J* = 8.1 Hz, 3H), 7.63-7.52 (m, 3H), 7.38 – 7.18 (m, 12H), 7.04 (d, *J* = 11.4 Hz, 1H), 6.92 – 6.91 (m, 2H), 5.87 (s, 1H), 4.86 – 4.69 (m, 3H), 4.25 (dd, *J* = 19.5, 8.4 Hz, 1H), 3.90 – 3.69 (m, 3H), 3.62 – 3.49 (m, 2H), 2.64-2.52 (m, 2H). ^{13}C NMR (176 MHz, $CDCl_3$) δ 151.91, 150.85, 147.93, 147.84, 144.30, 140.11, 139.94, 136.12, 131.54, 129.39, 129.15, 129.01, 128.75, 128.54, 128.50, 128.40, 128.18, 127.07, 127.03, 126.47, 124.43, 123.94, 118.23, 117.88, 66.09, 57.16, 56.41, 55.35, 31.35. HRMS (ESI-TOF) m/z: $[M+H]^+$ Calcd. For $C_{31}H_{27}N_3O_4$ 506.2074, found 506.2079.

Benzyl phenethyl (m-tolyl) *cyclic*-aminotroponiminium carboxylate (**6e**)

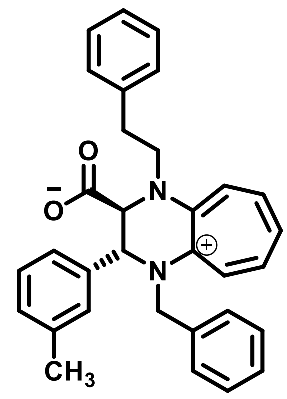

6e

The *cyclic*-aminotroponiminium compound (**6e**) was synthesized according to general procedures **(2.3)**. 170mg (45% yield) of the pure product was isolated as a yellow viscous liquid. FT-IR (KBr plate) ν (cm^{-1}) = 3456, 3029, 2924, 2057, 1660, 1596, 1 517, 1482, 1465, 1442, 1408, 1340, 1270, 1200, 1163, 1123. ^{1}H NMR (700 MHz, $CDCl_3$) δ 7.43 – 7.31 (m, 6H), 7.30 – 7.24 (m, 2H), 7.17 (d, *J* = 11.0 Hz, 4H), 7.02 (s, 1H), 6.88 (t, *J* = 9.3 Hz, 3H), 6.83 (d, *J* = 5.4 Hz, 2H), 6.70 (d, *J* = 11.4 Hz, 1H), 5.87 (s, 1H), 4.74 (d, *J* = 16.8 Hz, 1H), 4.69 – 4.56 (m, 2H), 3.86 – 3.76 (m, 1H), 3.66 (s, 1H), 2.56 – 2.44 (m, 1H), 2.44 – 2.30 (m, 4H). ^{13}C NMR (176 MHz, $CDCl_3$) δ 168.07, 152.03, 151.06, 139.38, 139.34, 139.19, 137.89, 137.07, 132.47, 129.53, 128.81, 128.56, 128.23, 127.34, 127.02, 126.88, 126.45, 126.01, 122.72, 116.93, 116.60, 70.96, 67.46, 56.79, 55.70, 31.38, 21.50. HRMS (ESI-TOF) m/z: $[M+H]^+$ Calcd. For $C_{32}H_{30}N_2O_2$ 475.2380, found 475.2368.

cyanobenzyl phenethyl phenyl *cyclic*-aminotroponiminium carboxylate **(6f)**

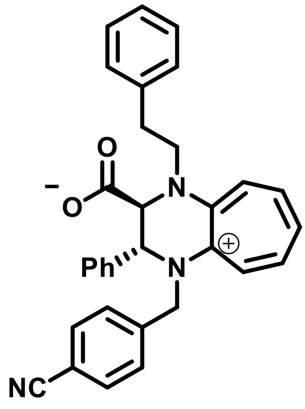

6f

The *cyclic*-aminotroponiminium compound (**6f**) was synthesized according to general procedures **(2.3)**. 60mg (88% yield) of the pure product was isolated as yellow viscous. FT-IR (KBr plate) ν (cm^{-1}) = 3407, 2921, 2851, 2229, 1672, 1599, 1519, 1495, 1452, 1346, 1198, 1129. ^{1}H NMR (400 MHz, CD_3CN) δ 7.76 (d, *J* = 7.0 Hz, 2H), 7.60 (dt, *J* = 20.2, 10.1 Hz, 2H), 7.41 (dd, *J* = 17.2, 6.8 Hz, 3H), 7.34 – 7.15 (m, 10H), 7.07 (d, *J* = 6.1 Hz, 2H), 4.98 (d, *J* = 17.0 Hz, 1H), 4.72 (d, *J* = 17.0 Hz, 1H), 4.25 (d, *J* = 13.1 Hz, 1H), 3.98 (d, *J* = 13.0 Hz, 1H), 3.81 (d, *J* = 5.7 Hz, 2H), 2.67 (d, *J* = 5.6 Hz, 1H), 2.47 (s, 1H). ^{13}C NMR (101 MHz, CD_3CN) δ 161.50, 151.44, 150.11, 141.98, 139.85, 139.73, 137.16, 133.03, 132.94, 129.22, 129.17, 128.79, 128.73, 128.20, 127.54, 126.93, 126.76, 120.80, 118.96, 118.38, 118.20, 117.91, 115.02, 112.33, 63.07,

55.34, 55.32, 54.89, 30.81. HRMS (ESI-TOF) m/z: $[M+H]^+$ Calcd. For $C_{32}H_{37}N_3O_2$ 486.2176, found 486.2190.

Benzyl-dimethoxynaphthalen-yl phenethyl *cyclic*-aminotroponiminium carboxylate (**6g**)

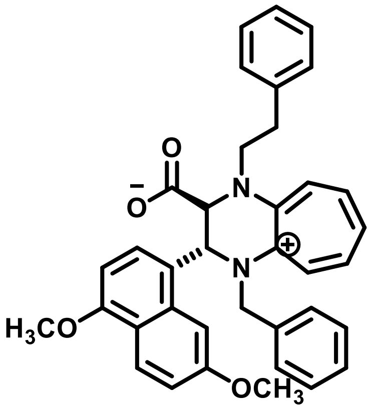

6g, 52%

The *cyclic*-aminotroponiminium compound (**6g**) was synthesized according to general procedures **(2.3)**. 35mg (52%) of the pure product was obtained as a yellow viscous.

FT-IR (KBr plate) ν (cm^{-1}) = 3374, 3058, 2924, 1970, 1617, 1595, 1518, 1494, 1481, 1466, 1440, 1409, 1339, 1268, 1212, 1167, 1076. ^1H NMR (700 MHz, $CDCl_3$) δ 7.51 – 7.38 (m, 9H), 7.33 (t, *J* = 10.0 Hz, 3H), 7.21 (d, *J* = 7.5 Hz, 6H), 6.94 – 6.86 (m, 3H), 6.85 (d, *J* = 6.5 Hz, 2H), 6.69 (d, *J* = 11.2 Hz, 1H), 5.98 (s, 1H), 4.77 (d, *J* = 16.9 Hz, 1H), 4.68 (d, *J* = 17.0 Hz, 1H), 4.59 (s, 1H), 4.02 (d, *J* = 11.8 Hz, 1H), 3.95 – 3.86 (m, 1H), 3.85 – 3.71 (m, 1H), 2.60 – 2.50 (m, 1H), 2.49 – 2.40 (m, 1H). ^{13}C NMR (176 MHz, $CDCl_3$) δ 167.01, 152.21, 151.11, 139.14, 138.26, 136.93, 132.25, 129.65, 129.47, 128.90, 128.72, 128.48, 128.30, 126.99, 126.31, 125.96, 116.58, 116.35, 71.47, 67.65, 56.82, 55.82, 31.37, 22.34, 14.06. HRMS (ESI-TOF) m/z: $[M]^+$ Calcd. For $C_{37}H_{35}N_2O_4$ 571.2591, found 571.2556.

sec-Butylphenethyphenyl *cyclic*-aminotroponiminium carboxylate(**6h**):

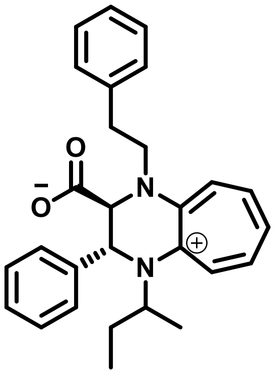

6h

The *cyclic*-aminotroponiminium compound (**6h**) was synthesized according to general procedures **(2.3)**53mg (88% yield) of the pure product was isolated as a yellow viscous solid. FT-IR (KBr plate) ν (cm^{-1}) = 3398, 3053, 2929, 2855, 1631, 1594, 1515, 1494, 1444, 1407, 1347, 1264, 1228, 1160, 1094. ^1H NMR (400 MHz, $CDCl_3$) δ 7.46 – 7.31 (m, 5H), 7.19 (s, 5H), 6.82 (s, 3H), 6.56 (dd, *J* = 20.0, 10.6 Hz, 1H), 5.92 (s, 1H), 4.54 (d, *J* = 12.7 Hz, 1H), 4.04 (s, 1H), 3.69 (s, 2H), 2.65 (s, 3H),

2.35 (s, 1H), 2.20 (s, 1H), 2.01 (d, J = 26.2 Hz, 1H), 1.53 (s, 2H), 1.26 (s, 1H), 1.05 (d, J = 8.5 Hz, 3H), 0.80 (s, 2H). ^{13}C NMR (101 MHz, $CDCl_3$) δ 166.72, 150.82, 150.72, 149.97, 139.49, 138.56, 138.36, 138.31, 136.95, 129.23, 129.18, 128.84, 128.41, 126.91, 125.64, 125.47, 125.24, 113.93, 113.88, 113.59, , 72.64, 54.86, 30.85, 27.54, 24.99, 17.56, 14.59, 11.47, 10.86. HRMS (ESI-TOF) m/z: $[M+H]^+$ Calcd. For $C_{28}H_{30}N_2O_2$ 427.2380, found 427.2368.

Phenethyl phenyl propynyl *cyclic*-aminotroponiminium carboxylate (**6i**)

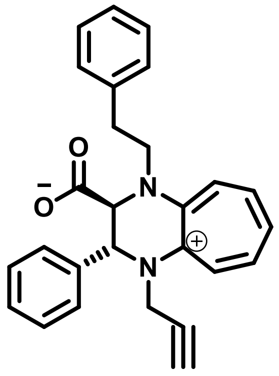

6i

The *cyclic*-aminotroponiminium compound (**6i**) was synthesized according to general procedures **(2.3)**. 95mg (82%) of the pure product was obtained as a yellow viscous. M.P 122–125 °C. FT-IR (KBr plate) ν (cm^{-1}) = 3282, 2923, 2117, 1977, 1686, 1596, 1518, 1494, 1481, 1451, 1411, 1342, 1283, 1223, 1197, 1166, 1120. ^{1}H NMR (700 MHz, Chloroform-*d*) δ 7.51 (t, J = 10.1 Hz, 1H), 7.42 – 7.33 (m, 4H), 7.21 – 7.13 (m, 6H), 6.99 (t, J = 9.3 Hz, 1H), 6.82 – 6.78 (m, 2H), 6.70 (d, J = 11.5 Hz, 1H), 5.96 (s, 1H), 4.61 – 4.49 (m, 2H), 4.23 (dd, J = 18.4, 1.9 Hz, 1H), 3.81 (dt, J = 15.7, 7.8 Hz, 1H), 3.73 (ddd, J = 14.8, 9.3, 5.5 Hz, 1H), 3.45 – 3.24 (m, 2H), 2.47 (h, J = 8.4, 7.6 Hz, 2H), 2.37 (ddd, J = 14.2, 8.8, 5.5 Hz, 1H). ^{13}C NMR (176 MHz, $CDCl_3$) δ 152.08, 149.93, 148.64, 139.71, 139.47, 139.28, 138.93, 137.39, 136.92, 129.30, 128.86, 128.66, 128.47, 127.65, 126.95, 125.87, 117.51, 116.98, 76.30, 74.50, 72.01, 68.64, 55.73, 43.26, 31.28.HRMS (ESI-TOF) m/z :$[M+H]^+$ Calcd. For $C_{27}H_{24}N_2O_2$ 409.1911, found 409.1955.

Octyl phenethyl phenyl *cyclic*-aminotroponiminium carboxylate (**6j**):

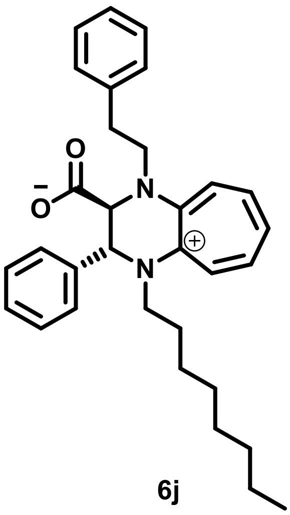

6j

The *cyclic*-aminotroponiminium compound (**6j**) was synthesized according to general procedures **(2.3)**.160mg (59%) of the pure product obtained as a yellow viscous. FT-IR (KBr plate) ν (cm^{-1}) = 3026, 2920, 2851, 1730, 1599, 1518, 1493, 1453, 1413, 1349, 1179, 1155. ^{1}H NMR (400 MHz, $CDCl_3$) δ 7.44 – 7.38 (m, 4H), 7.30 – 7.11 (m, 7H), 6.89-6.84 (m, 2H), 6.78-6.74 (m, 2H), 6.52 (d, *J* = 12.0 Hz, 1H), 5.90 (s, 1H), 4.53 (s, 1H), 3.79 (t, *J* = 7.2 Hz, 2H), 3.58 – 3.51 (m, 1H), 3.32 (td, *J* = 14.1, 4.8 Hz, 1H), 2.67 (s, 2H), 2.53 – 2.35 (m, 2H), 2.05 (s, 1H), 1.86 – 1.84 (m, 1H), 1.32 – 1.26 (m, 10H), 0.87 (t, *J* = 6.3 Hz, 3H). ^{13}C NMR (176 MHz, $CDCl_3$) δ 166.65, 151.07, 150.01, 138.76, 138.61, 137.53, 136.97, 129.32, 128.76, 128.56, 128.48, 128.33, 126.82, 125.90, 125.68, 115.10, 114.69, 71.31, 67.04, 63.56, 55.50, 52.91, 31.6, 31.18, 29.16, 28.89, 26.85, 25.65, 24.70, 22.49, 13.99. HRMS (ESI-TOF) m/z: $[M+H]^+$ Calcd. For $C_{38}H_{38}N_2O_2$ 483.3006, found 483.3015.

4-fluorophenyl octyl phenethyl *cyclic*-aminotroponiminium carboxylate **(6k)**

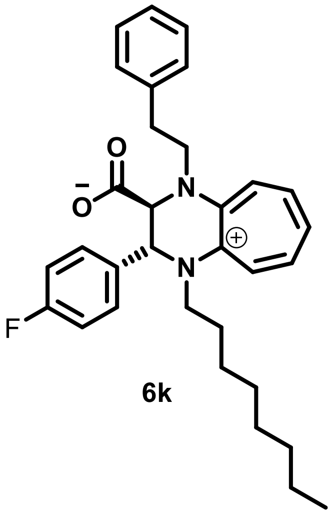

6k

The *cyclic*-aminotroponiminium compound (**6k**) was synthesized by according to general procedures **(2.3)**.110mg (76% yield) of the pure product was obtained as a light-yellow viscous liquid. M.P 123–125 °C. FT-IR (KBr plate) ν (cm^{-1}) = 2925, 2852, 1626, 1596, 1515, 1494, 1455, 1408, 1319, 1278, 1214, 1166, 1135. ^{1}H NMR (700 MHz, $CDCl_3$) δ 7.42 (t, *J* = 9.7 Hz, 1H), 7.28 (dd, *J* = 13.1, 7.1 Hz, 1H), 7.23 – 7.16 (m, 3H), 7.13 – 7.07 (m, 4H), 6.91 – 6.82 (m, 4H), 6.60 (d, *J* = 11.3 Hz, 1H), 5.88 (d, *J* = 6.7 Hz, 1H), 4.47 (s, 1H), 3.87 – 3.74 (m, 2H), 3.52 (t, *J* = 12.9 Hz, 1H), 3.29 (dd, *J* = 16.7, 9.4 Hz, 1H),

2.59 – 2.36 (m, 3H), 2.04 (s, 1H), 1.82 (s, 1H), 1.38 – 1.15 (m, 11H), 0.86 (t, *J* = 6.7 Hz, 3H). ^{13}C NMR (176 MHz, $CDCl_3$) δ 166.41, 163.33, 161.92, 151.19, 150.01, 139.00, 138.83, 136.87, 133.64, 128.87, 128.38, 127.59, 127.54, 127.00, 126.20, 116.45, 116.33, 115.40, 114.99, 31.69, 31.38, 29.24, 28.97, 26.93, 24.77, 22.57, 14.07. HRMS (ESI-TOF) m/z: $[M+H]^+$ Calcd. For C31H38N_2O_2F 457.3014, found 457.3033.

Cyclohexyl-hydroxyphenyl phenethyl *cyclic*-aminotroponiminium carboxylate (**6l**)

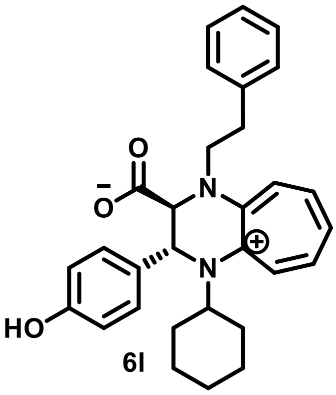

6l

The *cyclic*-aminotroponiminium compound (**6l**) was synthesized according to general procedures **(2.3)**. 80mg (76% yield) of the pure product was isolated as a yellow viscous solid. FT-IR (KBr plate) ν (cm^{-1}) = 3390, 2927, 2854, 1693, 1594, 1515, 1494, 1451, 1408, 1349, 1264, 1228, 1160, 1094. ^{1}H NMR (400 MHz, $CDCl_3$) δ 7.65 (dd, *J* = 15.8, 14.1 Hz, 1H), 7.50 – 7.28 (m, 4H), 7.24 – 7.13 (m, 4H), 6.97 – 6.77 (m, 4H), 6.55 (dd, *J* = 20.3, 11.2 Hz, 1H), 5.90 (d, *J* = 4.8 Hz, 1H), 4.53 (d, *J* = 13.0 Hz, 1H), 4.12 – 3.97 (m, 1H), 3.80 – 3.54 (m, 2H), 2.39 – 2.29 (m, 1H), 2.21 (ddd, *J* = 12.4, 8.8, 4.2 Hz, 1H), 1.96 (ddd, *J* = 23.4, 18.5, 10.8 Hz, 3H), 1.68 (s, 1H), 1.50 (s, 1H), 1.22 (s, 2H), 1.09 – 0.97 (m, 4H), 0.93 – 0.76 (m, 4H). ^{13}C NMR (101 MHz, $CDCl_3$) δ 190.67, 150.83, 150.04, 139.39, 138.62, 138.34, 136.92, 129.25, 129.20, 128.86, 128.40, 128.31, 126.95, 125.50, 125.33, 113.92, 113.73, 54.94, 30.88, 29.71, 25.00, 24.71, 17.57, 11.46, 10.86,HRMS (ESI-TOF) m/z: $[M+H]^+$ Calcd. For $C_{30}H_{32}N_2O_3$ 469.2471, found 469.2486.

Fluorobenzyl phenethyl phenyl *cyclic*-aminotroponiminium carboxylate(**6m**)

The *cyclic*-aminotroponiminium compound (**6m**) was synthesized according to general procedures **(2.3)**. 105mg (87% yield) of the pure product was isolated as a yellow viscous

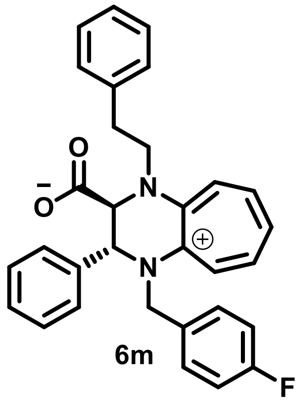

6m

liquid. M.P 120–122 °C. FT-IR (KBr plate) ν (cm^{-1}) = 3399, 3057, 2923, 1630, 1597, 1508, 1472, 1450, 1415, 1396, 1332, 1268, 1230, 1157, 1101, 1066. ^{1}H NMR (700 MHz, $CDCl_3$) δ 7.41 (d, *J* = 9.0 Hz, 3H), 7.27 – 7.18 (m, 7H), 7.10 (t, *J* = 8.1 Hz, 2H), 7.06 (d, *J* = 7.2 Hz, 1H), 6.93 (dt, *J* = 21.2, 9.1 Hz, 5H), 6.13 (s, 1H), 4.61 (s, 1H), 3.92 (dd, *J* = 17.5, 10.8 Hz, 2H), 2.69 – 2.59 (m, 1H), 2.53 (dd, *J* = 13.5, 6.4 Hz, 1H), 2.08 (s, 2H). ^{13}C NMR (176 MHz, $CDCl_3$) δ 167.00, 163.27, 161.85, 151.55, 150.77, 141.71, 139.01, 138.41, 136.78, 134.16, 129.41, 128.94, 128.41, 127.77, 127.69, 127.65, 127.09, 119.55, 117.51, 116.34, 116.21, 72.48, 69.52, 55.94, 31.40, 22.34, 14.06. HRMS (ESI-TOF) m/z: $[M+H]^+$ Calcd. For $C_{30}H_{25}FN_2O_2$ 465.1973, found 465.1973.

Benzyl phenethy styrenyl *cyclic*-aminotroponiminium carboxylate(**6n**)

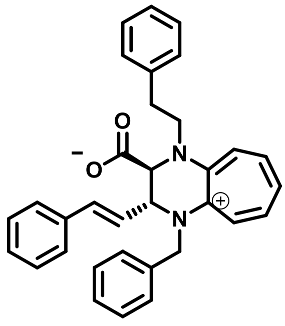

6n

The *cyclic*-aminotroponiminium compound (**6n**) was synthesized according to general procedures **(2.3)**. 105mg (67% yield) of the pure product was isolated as a yellow viscous solid. M.P 118–120 °C. FT-IR (KBr plate) ν (cm^{-1}) = 3279, 2928, 2355, 1620, 1597, 1520, 1495, 1466, 1441, 1406, 1345, 1276, 1223, 1207, 1166, 1075. ^{1}H NMR (400 MHz, $CDCl_3$) δ 7.43 (s, 5H), 7.34 (s, 5H), 7.18 (s, 7H), 6.95 – 6.73 (m, 4H), 6.49 (d, *J* = 15.6 Hz, 1H), 6.23 – 6.11 (m, 1H), 5.33 (d, *J* = 29.0 Hz, 1H), 4.90 (d, *J* = 16.5 Hz, 1H), 4.64 (dd, *J* = 40.0, 23.2 Hz, 2H), 4.50 (s, 1H), 4.13 – 3.84 (m, 2H), 3.65 (d, *J* = 33.1 Hz, 1H), 3.02 (s, 2H). ^{13}C NMR (101 MHz, $CDCl_3$) δ 167.22, 152.11, 151.62, 139.34, 139.23, 139.12, 139.02, 138.24, 136.99, 136.91, 135.17, 133.93, 132.54, 132.37, 129.63, 129.59, 129.48, 128.95, 128.86, 128.77, 128.53, 128.26, 127.12, 127.09, 126.93, 126.76, 126.44, 126.28, 125.93, 123.99, 117.22, 117.09, 116.58, 71.46, 68.80, 67.62, 66.00, 56.52, 55.97, 32.10, 31.31. HRMS (ESI-TOF) m/z: $[M+Na]^+$ Calcd. For $C_{33}H_{30}N_2O_2Na$ 509.2199, found 509.2216.

Benzyl-methoxyphenylphenethyl *cyclic*-aminotroponiminium carboxylate **(6o):**

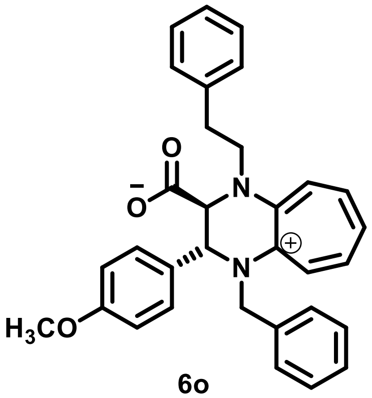

6o

The *cyclic*-aminotroponiminium compound (**6o**) was synthesized according to general procedures **(2.3)**. 52mg (41% yield) of the pure product was isolated as a yellow viscous liquid. FT-IR (KBr plate) ν (cm^{-1}) = 3054, 2940, 1673, 1597, 1514, 1495, 1442, 1409, 1347, 1263, 1178, 1136. ^{1}H NMR (400 MHz, $CDCl_3$) δ 7.42 (s, 5H), 7.36 – 7.31 (m, 1H), 7.18 (d, *J* = 5.0 Hz, 5H), 7.13 – 7.01 (m, 4H), 6.96 – 6.84 (m, 4H), 5.71 (d, *J* = 30.2 Hz, 1H), 5.07 (s, 1H), 4.72 (t, *J* = 15.8 Hz, 2H), 4.14 (d, *J* = 6.9 Hz, 1H), 3.84 – 3.71 (m, 3H), 3.64 (s, 1H), 3.27 (s, 11H), 3.06 (s, 1H), 2.53 (s, 1H). ^{13}C NMR (101 MHz, $CDCl_3$) δ 160.32, 151.95, 151.71, 140.12, 139.99, 136.57, 131.56, 129.79, 129.73, 129.42, 128.95, 128.66, 128.61, 127.33, 127.13, 126.25, 126.20, 125.96, 118.12, 117.45, 114.98, 77.37, 76.74, 65.68, 56.31, 55.45, 31.53, 22.67, 14.15. HRMS (ESI-TOF) m/z: $[M+H]^+$ Calcd. For $C_{33}H_{30}N_2O_3$ 491.2329, found 491.2312.

5. **Crystal structures and data**

Good quality crystals of compounds were obtained in solvent mixture dichloromethane and hexane by slow evaporation method. The crystals data of two compounds were collected on a Rigaku Oxford diffractometer at 293 K respectively. Selected data collection parameters and other crystallographic results are summarized below. The program package SHELXTL[1] and Olex2 was used for structure solution and packing diagram carried out by DIAMOND-3.2 software.

CCDC deposition number of **2** (2035166)**, 6a** (1889119), **6d** (2035165) and **6k** (2035167) contain the supplementary crystallographic data, these data can be obtained free of charge via https://www.ccdc.cam.ac.uk/data.

Table S1. Crystal data and structure refinement for *tr-pheneg* methyl ester (**2**)

Identification code	**CCDC 2035166**
Empirical formula	$C_{19}H_{21}NO_3$
Formula weight	311.37
Temperature/K	293(2)
Crystal system	monoclinic
Space group	$P2_1/c$
a/Å	5.4825(3)
b/Å	23.8368(16)
c/Å	13.0746(8)
α/°	90
β/°	95.892(5)
γ/°	90
Volume/Å^3	1699.63(18)
Z	4
ρ_{calc}g/cm^3	1.217
μ/mm^{-1}	0.082
F(000)	664.0
Crystal size/mm^3	0.021 × 0.0111 × 0.003
Radiation	MoKα (λ = 0.71073)
2Θ range for data collection/°	6.838 to 52.744
Index ranges	$-6 \leq h \leq 6$, $-27 \leq k \leq 29$, $-16 \leq l \leq 16$
Reflections collected	15011
Independent reflections	3463 [R_{int} = 0.0692, R_{sigma} = 0.0470]
Data/restraints/parameters	3463/0/209
Goodness-of-fit on F^2	1.050
Final R indexes [I>=2σ (I)]	R_1 = 0.0771, wR_2 = 0.2111
Final R indexes [all data]	R_1 = 0.1018, wR_2 = 0.2297
Largest diff. peak/hole / e Å^{-3}	0.41/-0.19

(a)

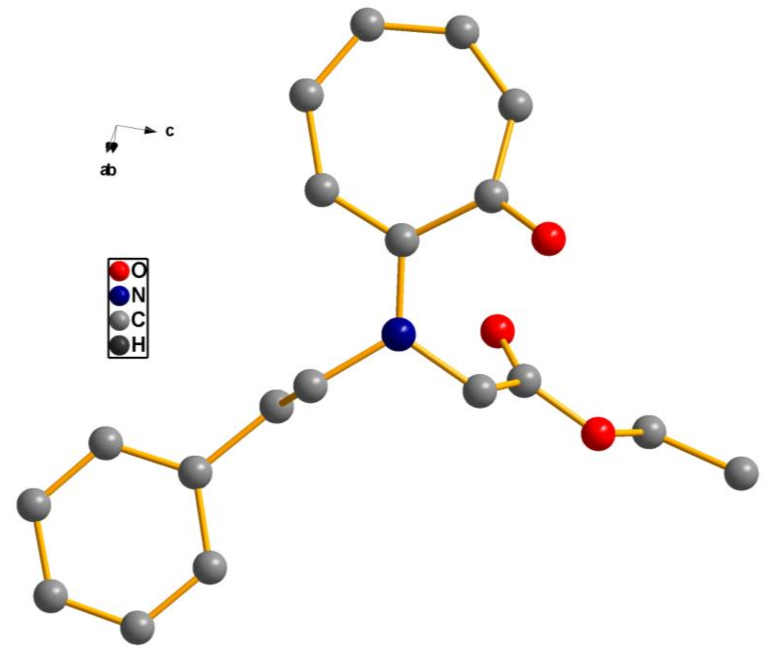

(b)

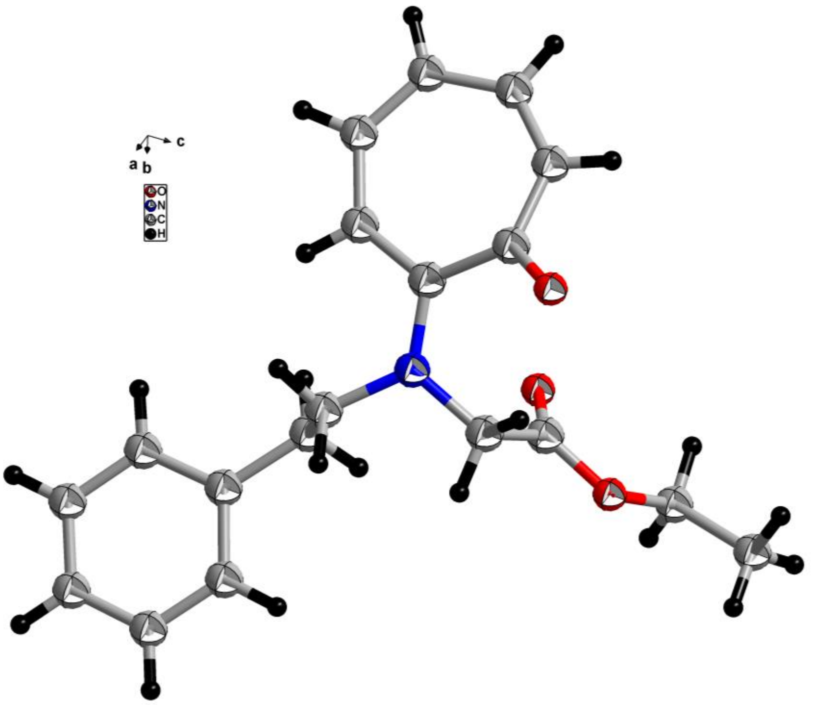

Fig S1. X-ray structure: (a) single crystal structure, and (b) ORTEP diagram of compound **2**

Table S2. *Comparison of selective ^{13}C peaks of hydrapyrazine (**6a-6o**) and tr-pheneg methyl ester (2)*

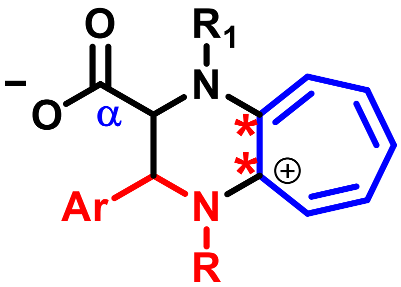

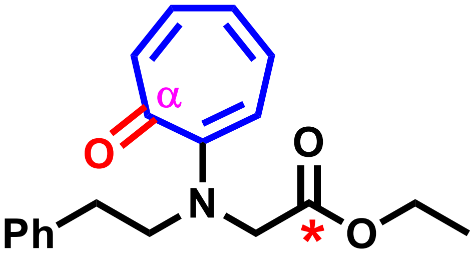

2

Entry No.	***Compounds***	*α*	**
01	***6a***	*167.3*	*151.0, 150.0*
02	***6b***	*167.0*	*152.4, 150.9*
03	***6c***	*no*	*151.1, 148.9*
04	***6d***	*no*	*151.9, 150.8*
05	***6e***	*167.2*	*151.1, 151.0*
06	***6f***	*161.5*	*151.4, 150.3*
07	***6g***	*no*	*150.5, 150.4*
08	***6h***	*166.7*	*150.8, 150.0*
09	***6i***	*167.0*	*152.0, 149.9*
10	***6j***	*167.7*	*151.1, 150.1*
11	***6k***	*164.1*	*150.0, 149.7*
12	***6l***	*no*	*150.8, 150.0*
13	***6m***	*170.0*	*151.8, 150.7*
14	***6n***	*167.7*	*151.6, 150.1*
15	***6o***	*no*	*151.9, 151.7*
16	***2***	*181.8*	*170.9*
			*ACS Omega **2018**, 3, 997−1013*

no= not observed/ very small possibly because of steric effect of bulky aryl group.

(a)

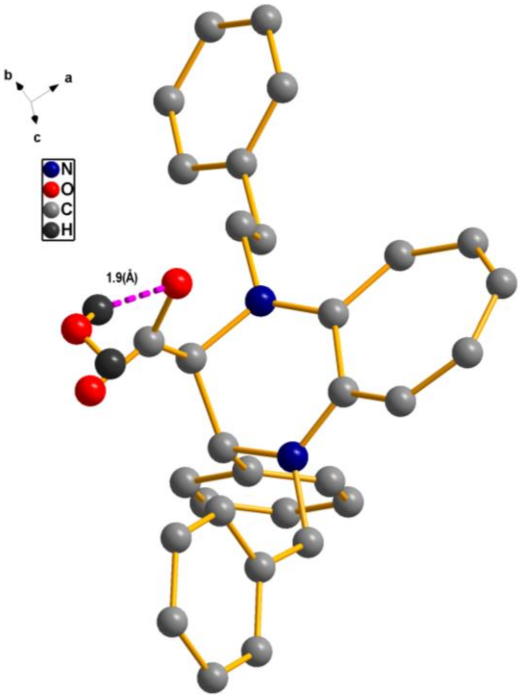

(b)

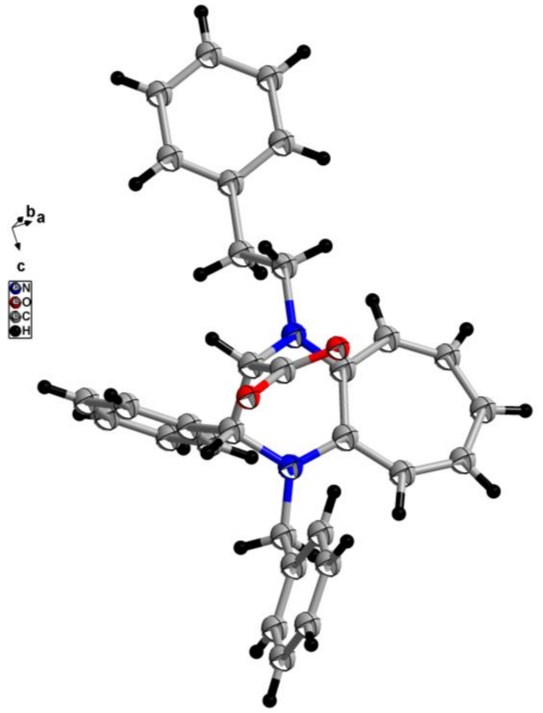

Fig S2. ORTEP diagram of cATC derivative **6a**

Table S3. Crystal data and structure refinement for *N*-benzyl-*N*-phenylethyl phenyl *cyclic*-aminotroponiminium carboxylate (**6a)**

Identification code	**CCDC 1889119**
Empirical formula	C31H30N2O3
Formula weight	478.57
Temperature/K	293(2)
Crystal system	monoclinic
Space group	
Unit cell dimensions:	P21/n
a/Å	9.1107(3)
b/Å	16.5195(9)
c/Å	17.0838(5)
α/°	90
β/°	99.724(3)
γ/°	90
Volume/Å3	2534.24(18)
Z	4
ρcalcg/cm3	1.254
μ/mm 1	0.642
F(000)	1016.0
Crystal size/mm3	0.14 × 0.12 × 0.1
Radiation	CuKα (λ = 1.54184)
2Θ range for data collection/°	7.496 to 149.002
Index ranges	-11 ≤ h ≤ 11, -20 ≤ k ≤ 20, -21 ≤ l ≤ 20
Reflections collected	19921
Independent reflections	5056 [Rint = 0.0896, Rsigma = 0.0730]
Data/restraints/parameters	5056/0/328
Goodness-of-fit on F2	1.059
Final R indexes [I>=2σ (I)]	R1 = 0.0842, wR2 = 0.2280
Final R indexes [all data]	R1 = 0.1141, wR2 = 0.2509
Largest diff. peak/hole / e Å-3	0.47/-0.32

Benzyl nitrophenyl phenethyl cyclic-aminotroponiminium carboxylate ***(6d)***

Table S4: Crystal data and structure refinement for *N*-benzyl-*N*-phenylethyl nitrophenyl *cyclic*-aminotroponiminium **(6d)**

Identification code	**CCDC 2035165**
Empirical formula	$C_{31}H_{28}N_3O_4$
Formula weight	506.56
Temperature/K	297(1)
Crystal system	monoclinic
Space group	$P2_1/n$
a/Å	9.6632(8)
b/Å	22.944(4)
c/Å	16.041(2)
α/°	90
β/°	101.653(12)
γ/°	90
Volume/Å^3	3483.3(9)
Z	4
ρ_{calc}g/cm^3	0.966
μ/mm^{-1}	0.521
F(000)	1068.0
Crystal size/mm^3	0.3 × 0.2 × 0.1
Radiation	CuKα (λ = 1.54184)
2Θ range for data collection/°	6.818 to 136.5
Index ranges	$-11 \leq h \leq 7$, $-27 \leq k \leq 27$, $-19 \leq l \leq 19$
Reflections collected	35399
Independent reflections	6151 [R_{int} = 0.2125, R_{sigma} = 0.1301]
Data/restraints/parameters	6151/0/344
Goodness-of-fit on F^2	1.192
Final R indexes [I>=2σ (I)]	R_1 = 0.1592, wR_2 = 0.3887
Final R indexes [all data]	R_1 = 0.2473, wR_2 = 0.4536
Largest diff. peak/hole / e Å^{-3}	0.56/-0.54

(a)

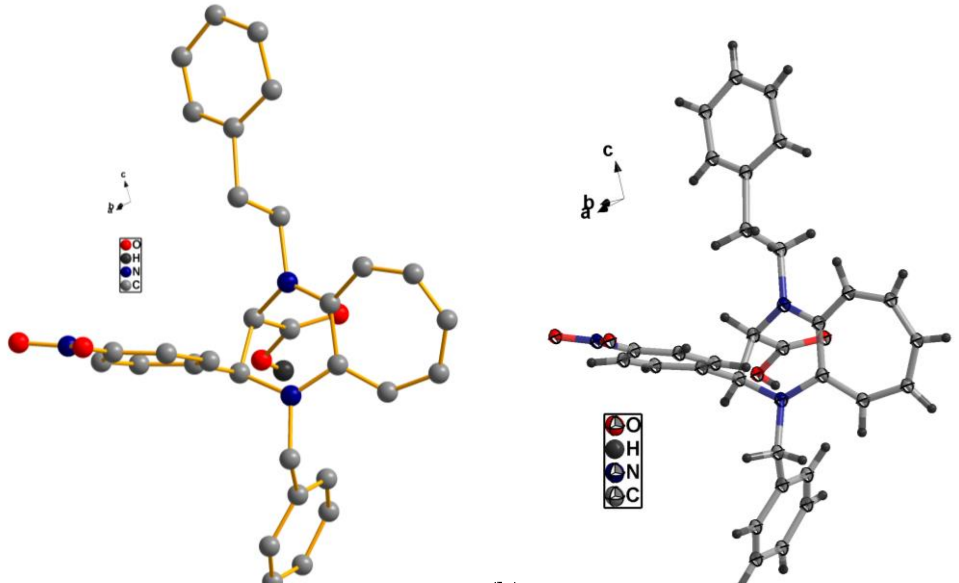

(b)

Fig S3. Crystal struutre of **6d** and ORTEP diagram of cATC derivative **6d.**

Table S5. Crystal data and structure refinement for *N*-octayl-*N*-phenylethyl 4-flurophenyl *cyclic*-aminotroponiminium carboxylate **(6k)**

Identification code	**CCDC 2035167**
Empirical formula	C32H37FN2O2
Formula weight	37.20
Temperature/K	295(2)
Crystal system	triclinic
Space group	P-1
a/Å	10.5590(3)
b/Å	15.1481(3)
c/Å	15.2096(4)
α/°	111.565(2)
β/°	105.940(2)
γ/°	95.356(2)
Volume/Å^3	2123.51(10)
Z	40
ρ_{calc}g/cm^3	1.163
μ/mm^{-1}	0.710
F(000)	796.0
Crystal size/mm^3	0.12 × 0.1 × 0.02
Radiation	CuKα (λ = 1.54184)
2Θ range for data collection/°	6.628 to 148.996
Index ranges	$-13 \le h \le 13$, $-18 \le k \le 14$, $-19 \le l \le 18$
Reflections collected	28676
Independent reflections	8486 [R_{int} = 0.0499, R_{sigma} = 0.0414]
Data/restraints/parameters	8486/3/510
Goodness-of-fit on F^2	1.732
Final R indexes [I>=2σ (I)]	R_1 = 0.1211, wR_2 = 0.3988
Final R indexes [all data]	R_1 = 0.1488, wR_2 = 0.4366
Largest diff. peak/hole / e Å^{-3}	1.39/-0.47

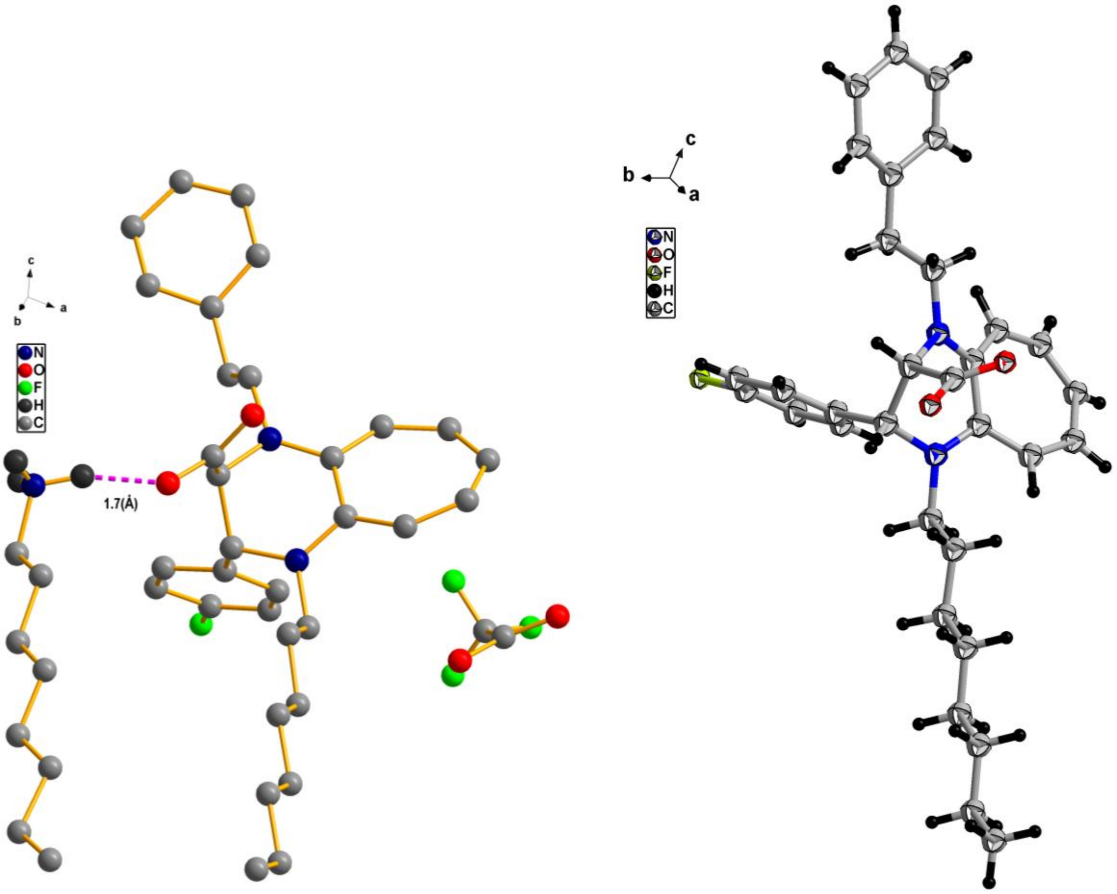

Fig S4. Crystal structure (A) and ORTEP diagram of cATC derivative **6k.**

6. Time dependent ^{1}H-NMR spectra in CD_3CN

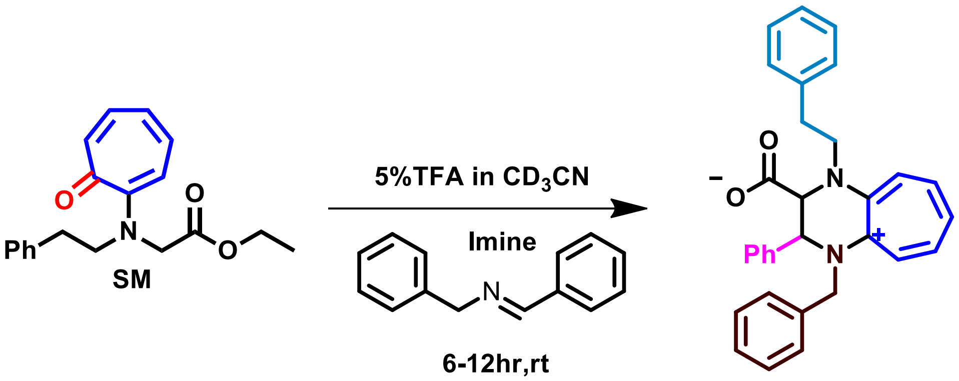

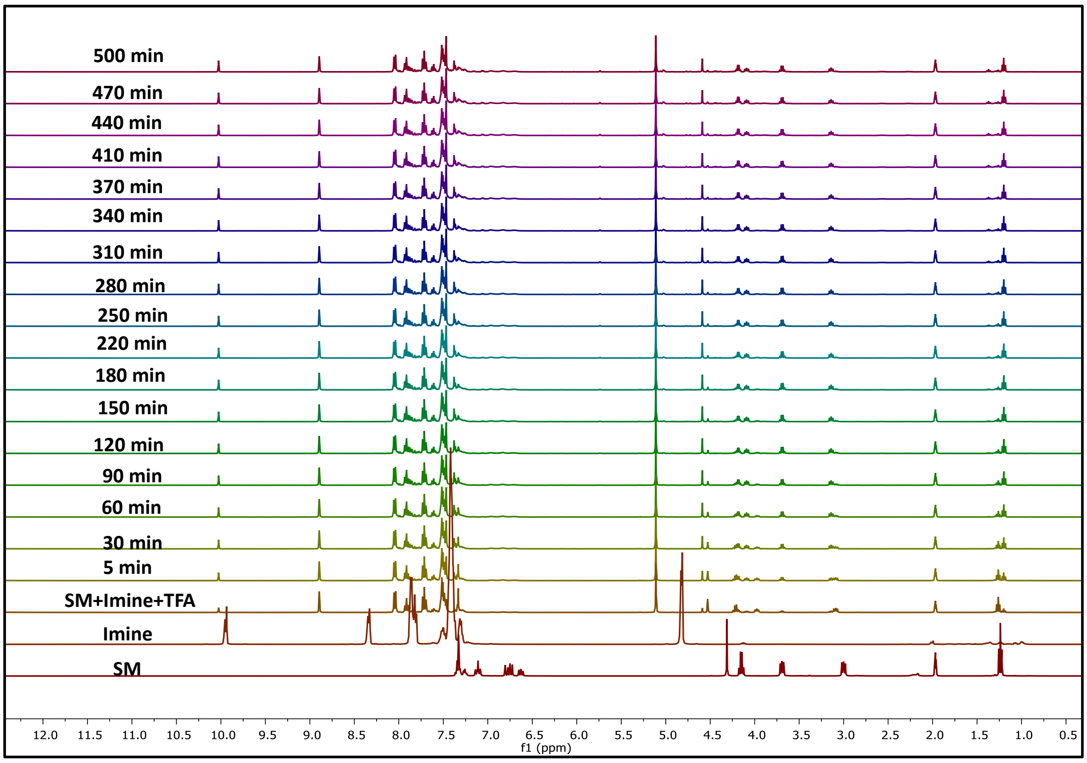

Fig S5: Time dependent NMR in CD_3CN.

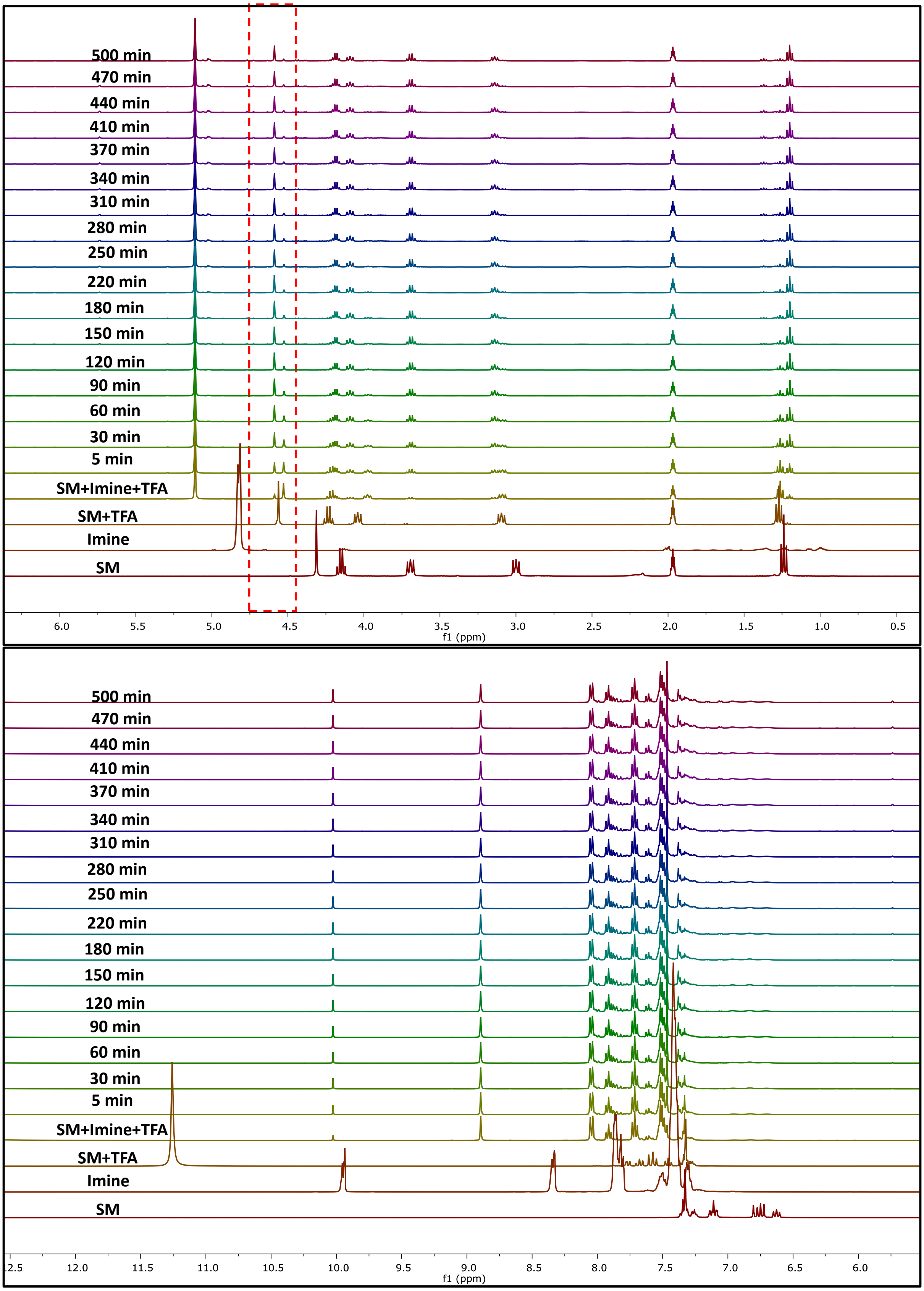

Fig S6: Expanded Spectra of Time depended NMR in CD_3CN.

7. Time dependent Mass analysis of cATC formation

Fig

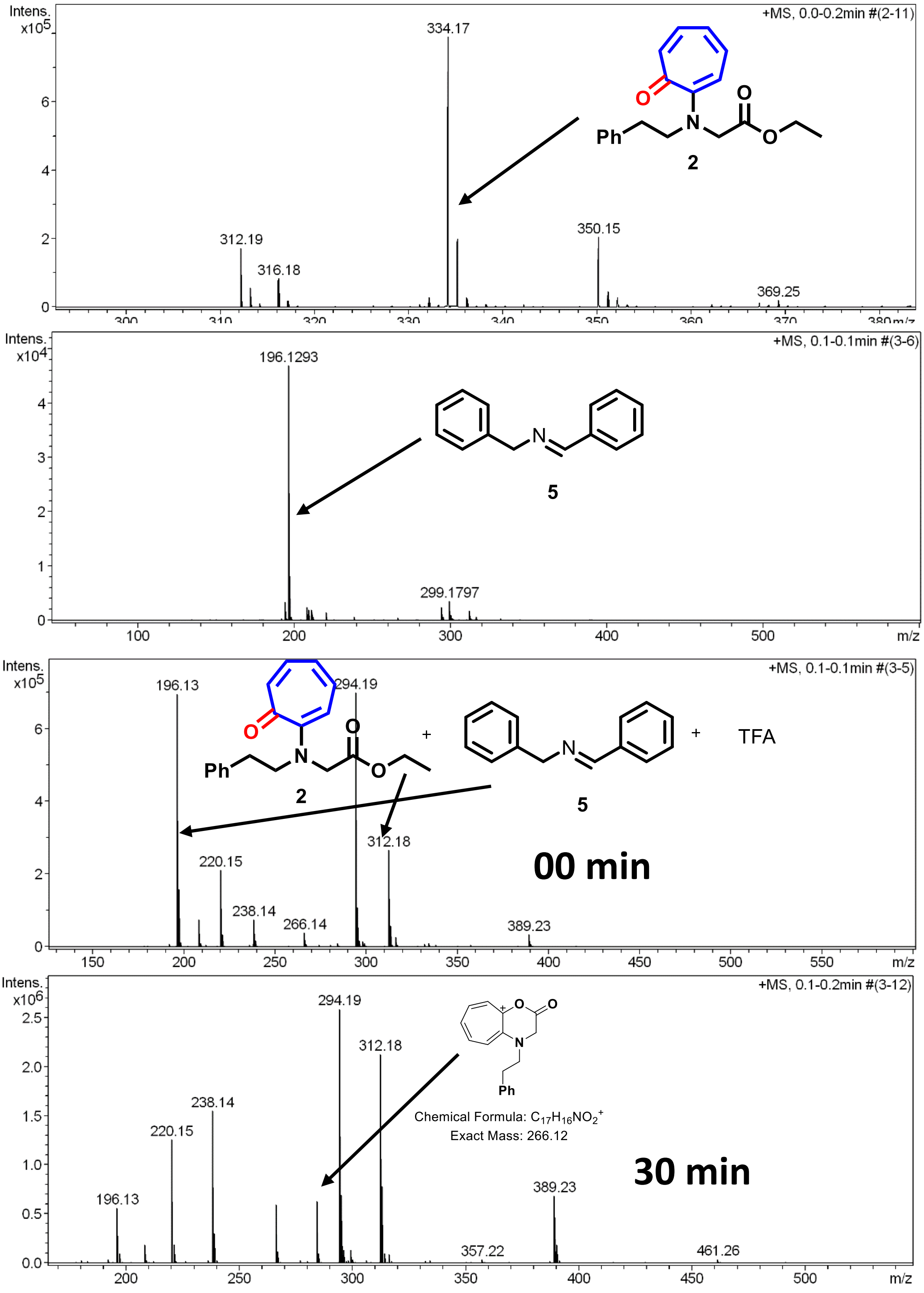

Fig S7. Time dependent mass of cATC formation

Continue..

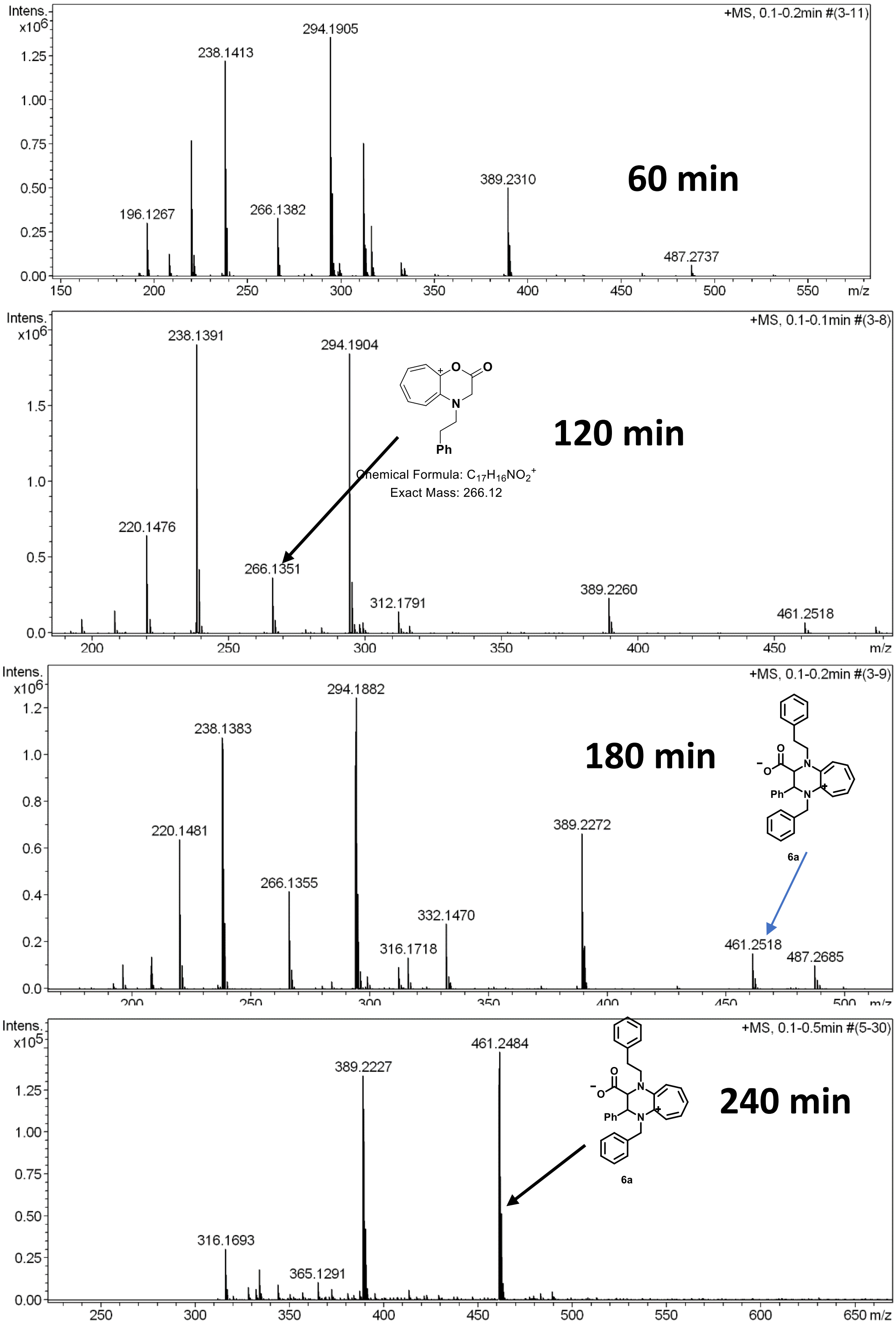

Fig 8 . Time dependent mass of cATC formation

8. UV-Visible and Fluorescence Spectroscopic Studies formation of cATC-derivatives

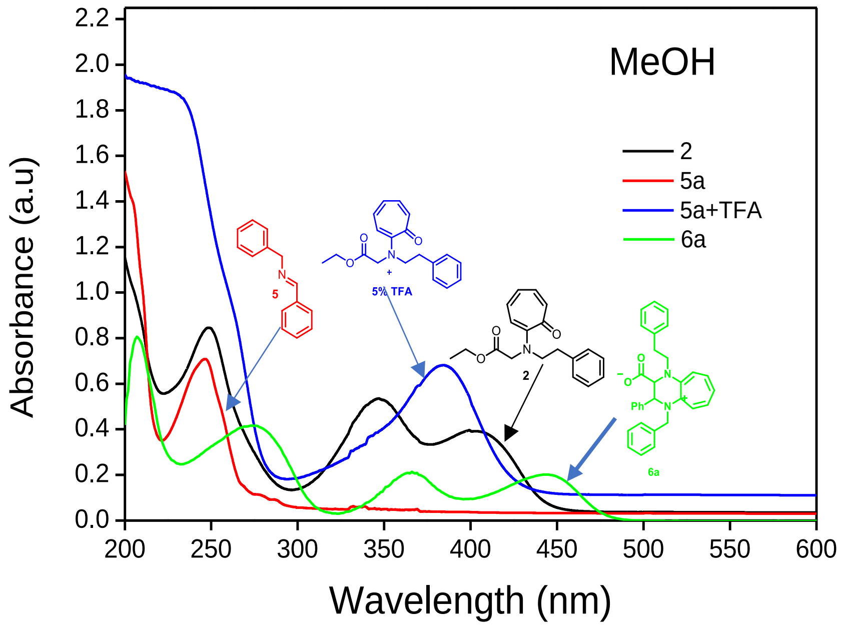

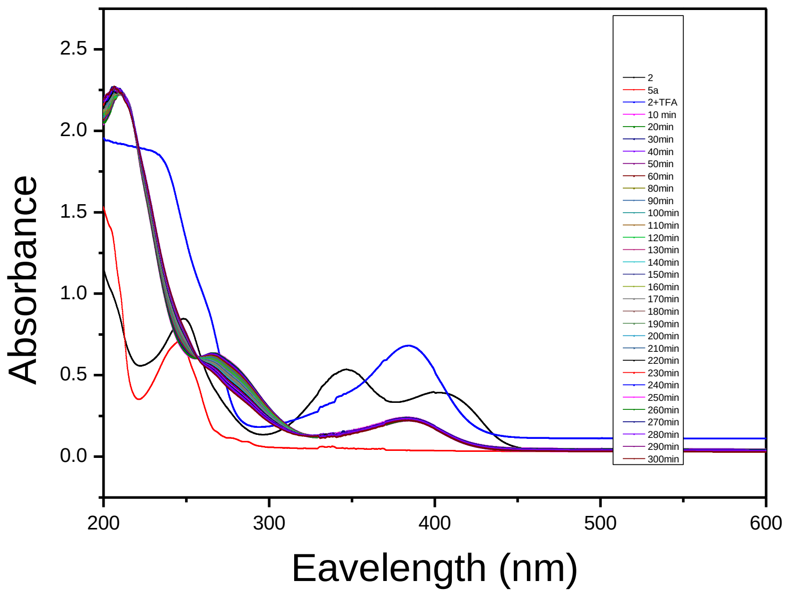

Fig S9: Time dependent UV-Vis spectra in 5% TFA (CH_3CN)

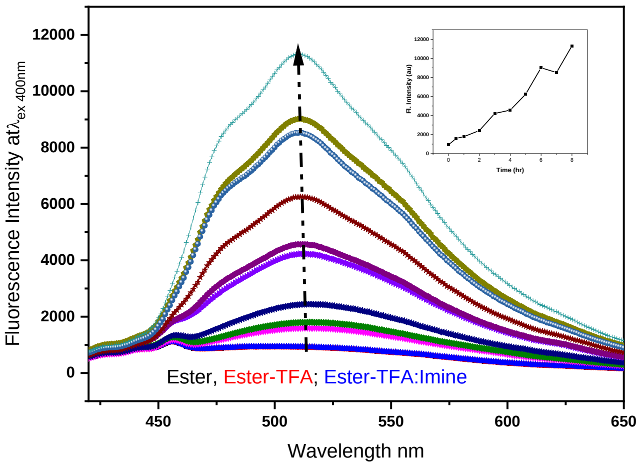

Fig S10: Time dependent fluorescence studies of reaction mixture 5% TFA (CH_3CN)

9. Computational Studies of proposed reaction mechanism

Methodology

Geometry optimizations of all stationary points on the PES were done in the gas phase using the B3LYP functional [1] and the 6-311G** [2] basis set. Frequency calculations were performed on the optimized geometries to verify all minimum energy structures have only positive Hessian matrix. A minima on the PES was characterized by 3N-6 positive frequencies whereas a transition state was characterized by one imaginary frequency representing the desired reaction coordinate and remaining 3N-7 positive frequencies. All calculations were carried out using the Gaussian16 suite of quantum chemical program. [3]

Table S1. Relative free energies (Hartree/Particle) obtained at the B3LYP/6-311G (d,p) level of theory for the different stationary points for the reaction. The energies are calculated with respect to the sum of energies of the Reactants.

Reaction Mechanism	
Stationary points	ΔG (Hartree/Particle)
Ts1	-7.028
Int1	-9.6886
Ts2	32.04642
Int2	-1.3303
Ts3	0.251
Pdt	-11.8723

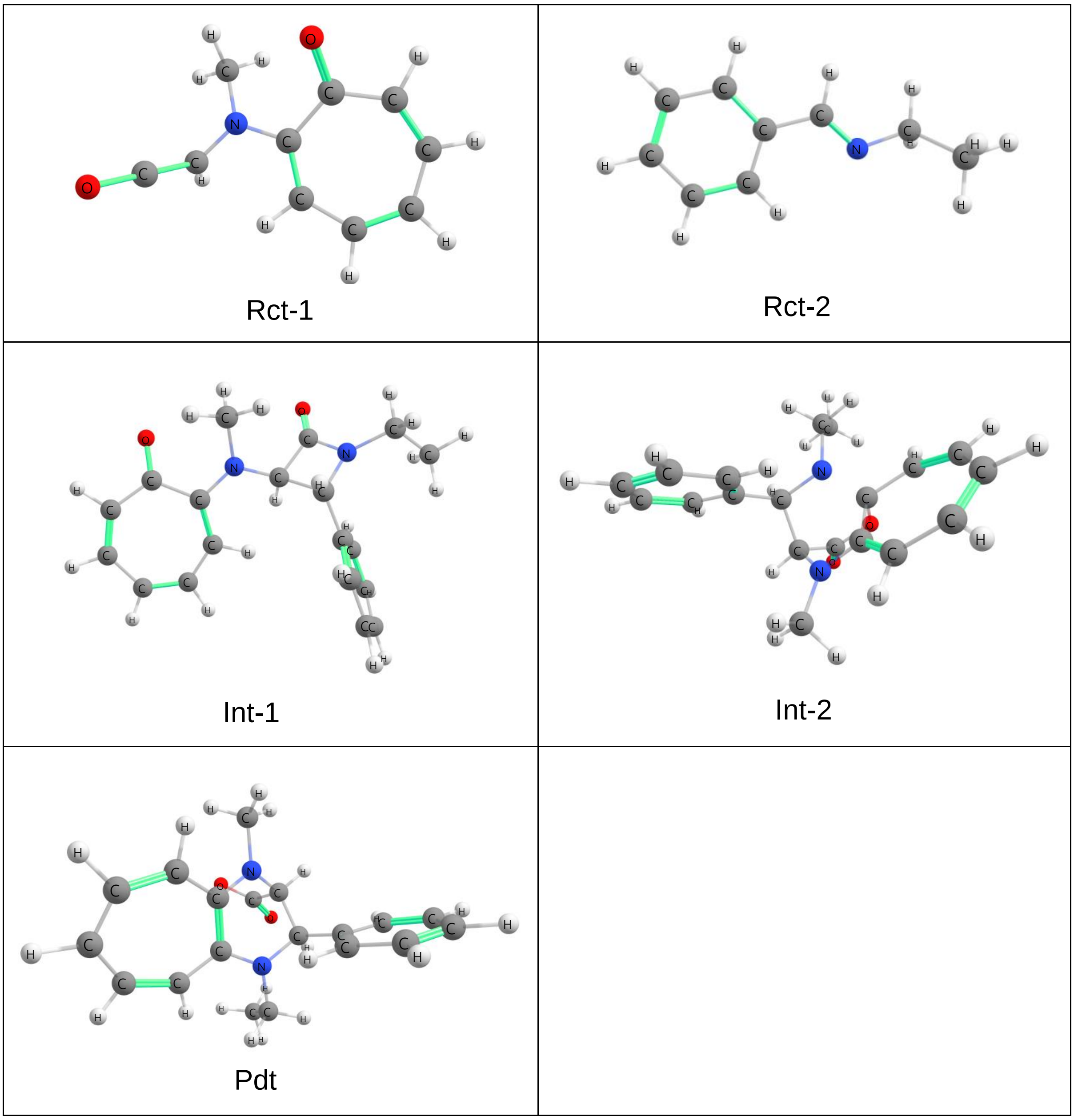

Fig S11. Optimized structures of reactants, intermediates and product

Proposed pathway for the reaction

We performed DFT calculation (B3LYP) of cATC model compound (**S0**) to establish the reaction mecanism theoratically. Compound (S0) formed *ketene* under acidic conditions that reacted with imine intermediate to produce lactam intermediate (**Int1**) via transition state (Ts1) transition state. This lactam intermideiate (**Int1**) rearranged to lactone intermediate (**Int2**) via transition state Ts2. Finally **Int-2** lead to the product through Ts3.

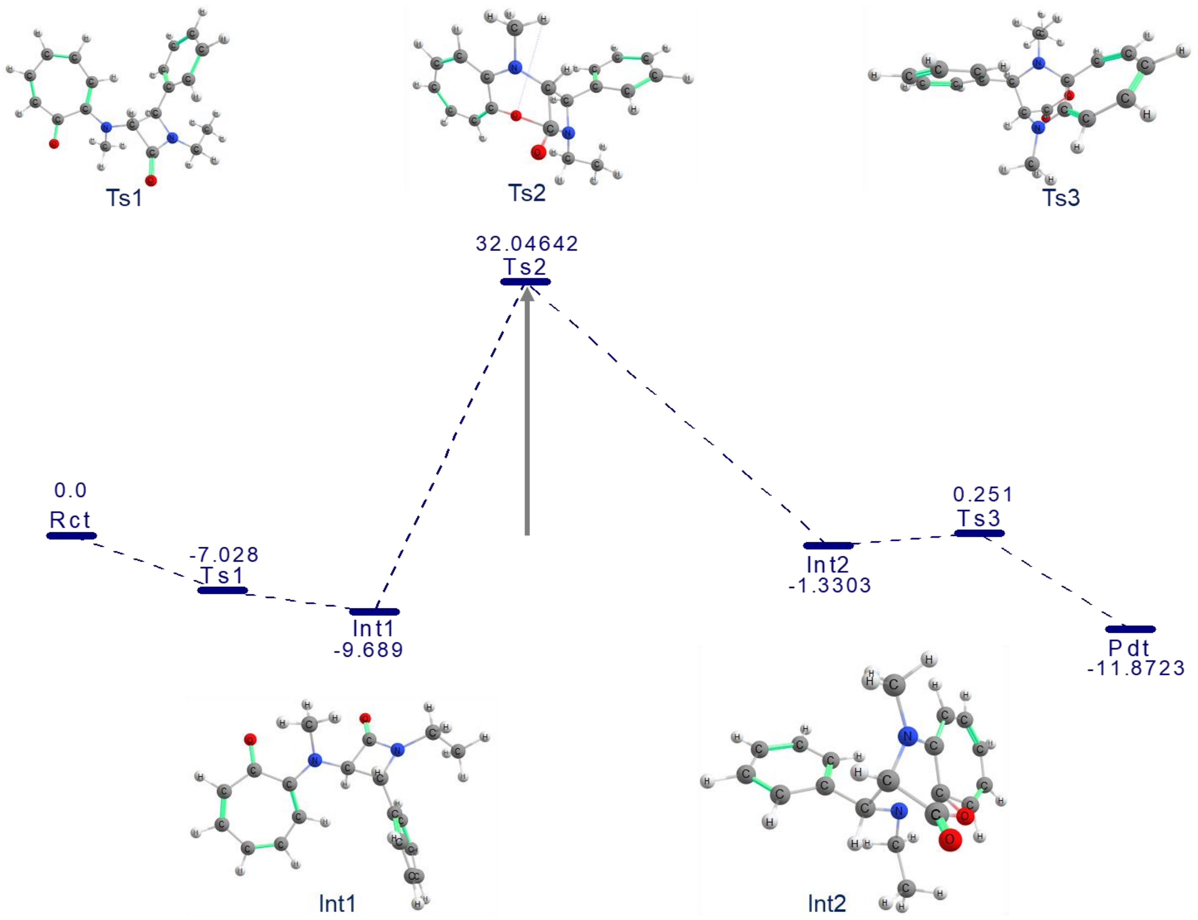

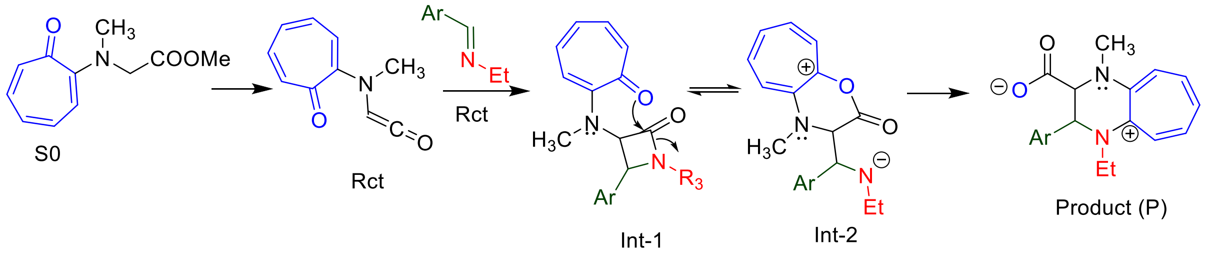

Fig S12. Proposed pathways of cATC formation reaction

10. **Photophysical studies of cATC derivatives**

(a) UV-Vis Spectra if cATC derivatives

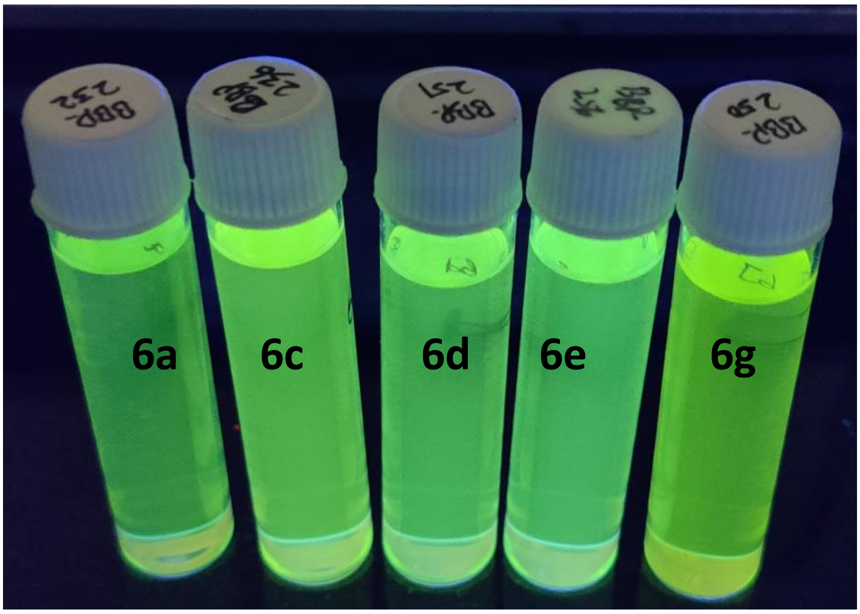

Fig S13. Fluorescence images of cATC derivative samples (**6a/6c/6d/6e/6g**)

(b) Photophysical parameters of cATC derivatives

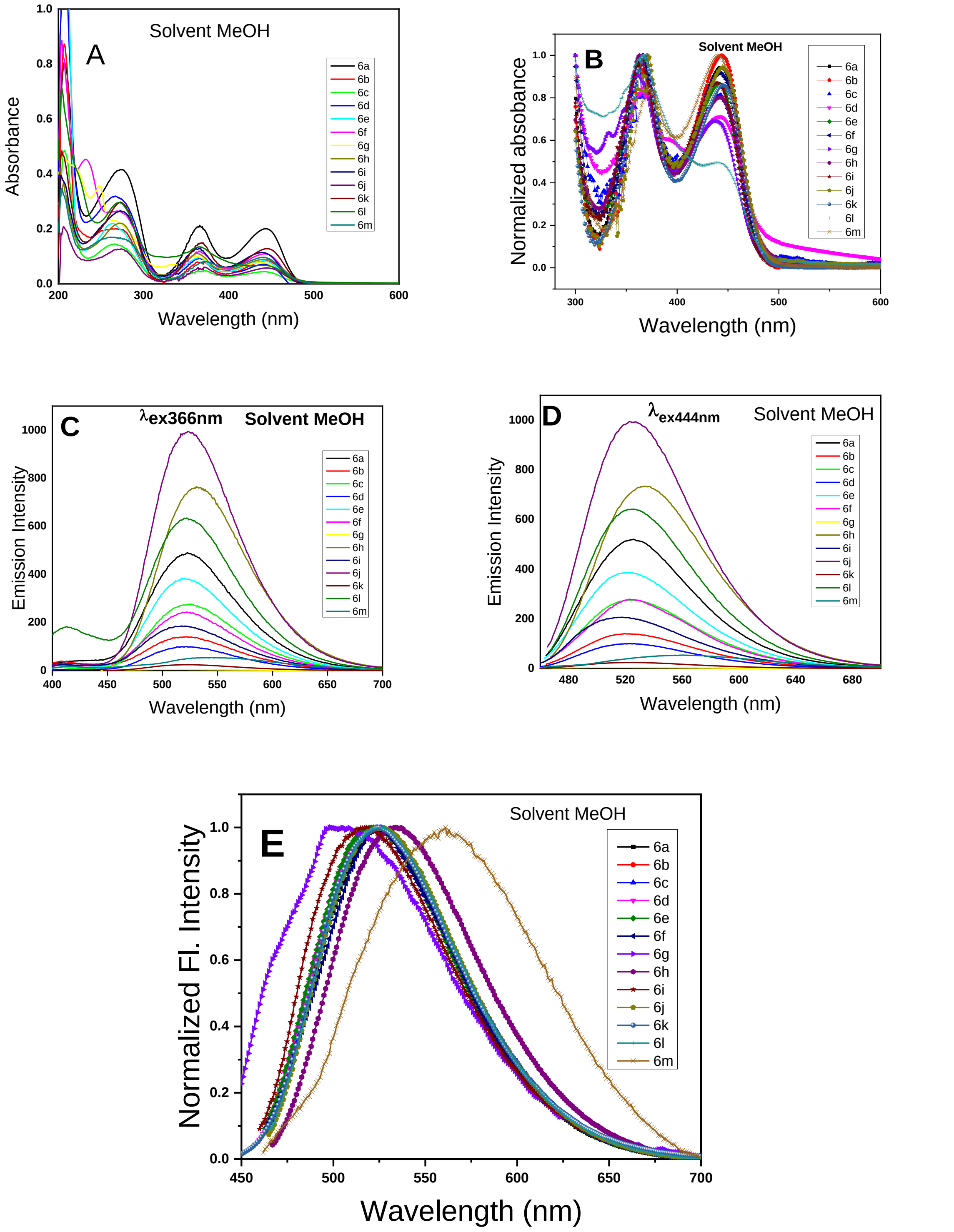

Fig S14:.A) UV-Vis absorption spectra of cATC derivatives (**6a-m**); B) their Normalized UV-Vis spectra; C) Emission spectra of cATC derivatives (**6a-m**) at $\lambda_{ex,\ 366nm}$; D) Emission spectra of cATC derivatives (**6a-m**) at $\lambda_{ex,\ 444nm}$; E) Normalized emission spectra of cATC derivatives (**6a-m**) at $\lambda_{ex,\ 444nm}$. The concentration of cATC derivatives (**6a-m**) in methanol solvent was constant as [cATC] = $5x10^{-5}$M.

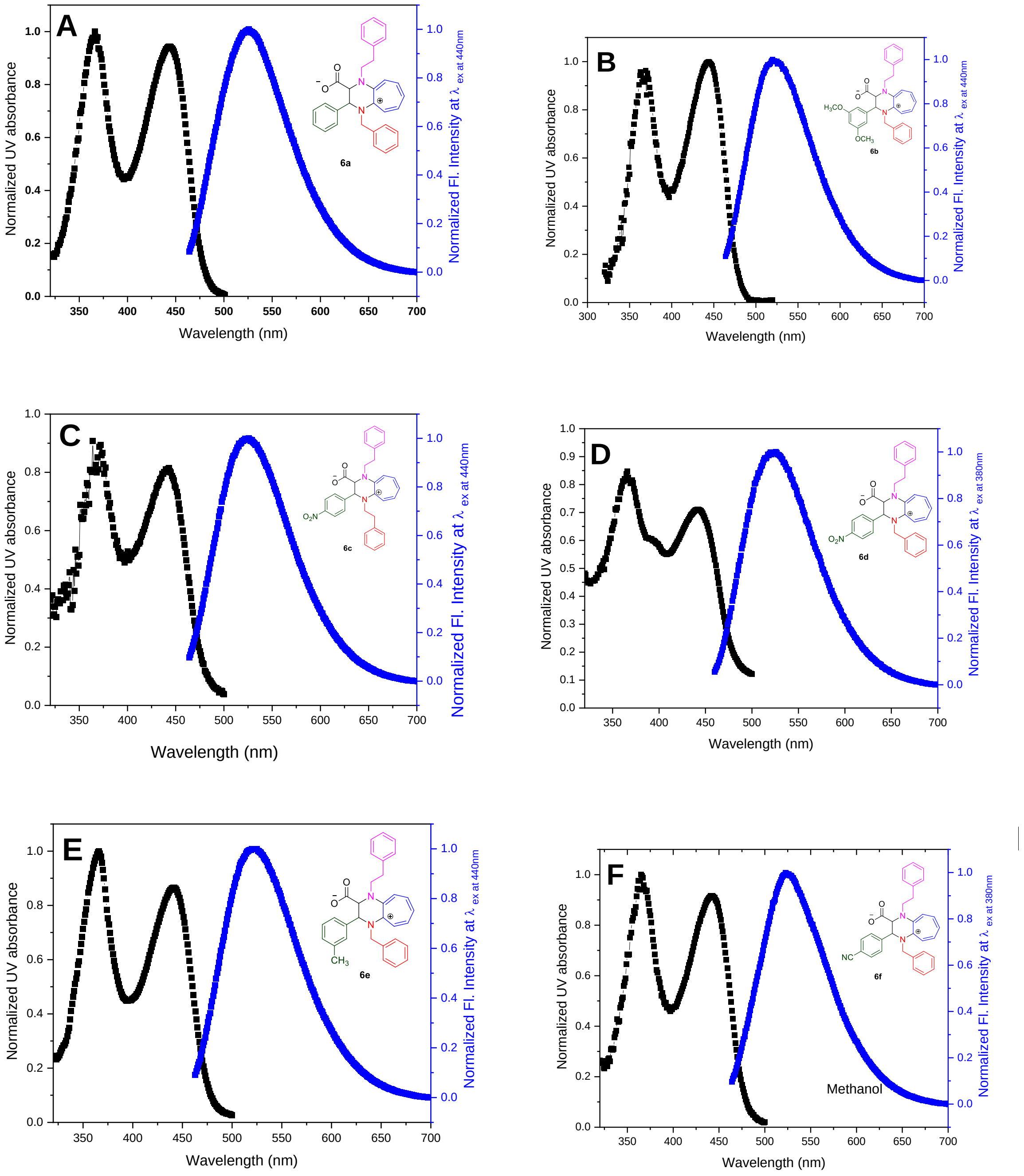

Fig S15: Normalized absorption-emission spectra of cATC derivatives (**6a-f**) in solvent methanol at $\lambda_{ex,\ 444nm}$. Their concentration is $5x10^{-5}$M in MeOH.

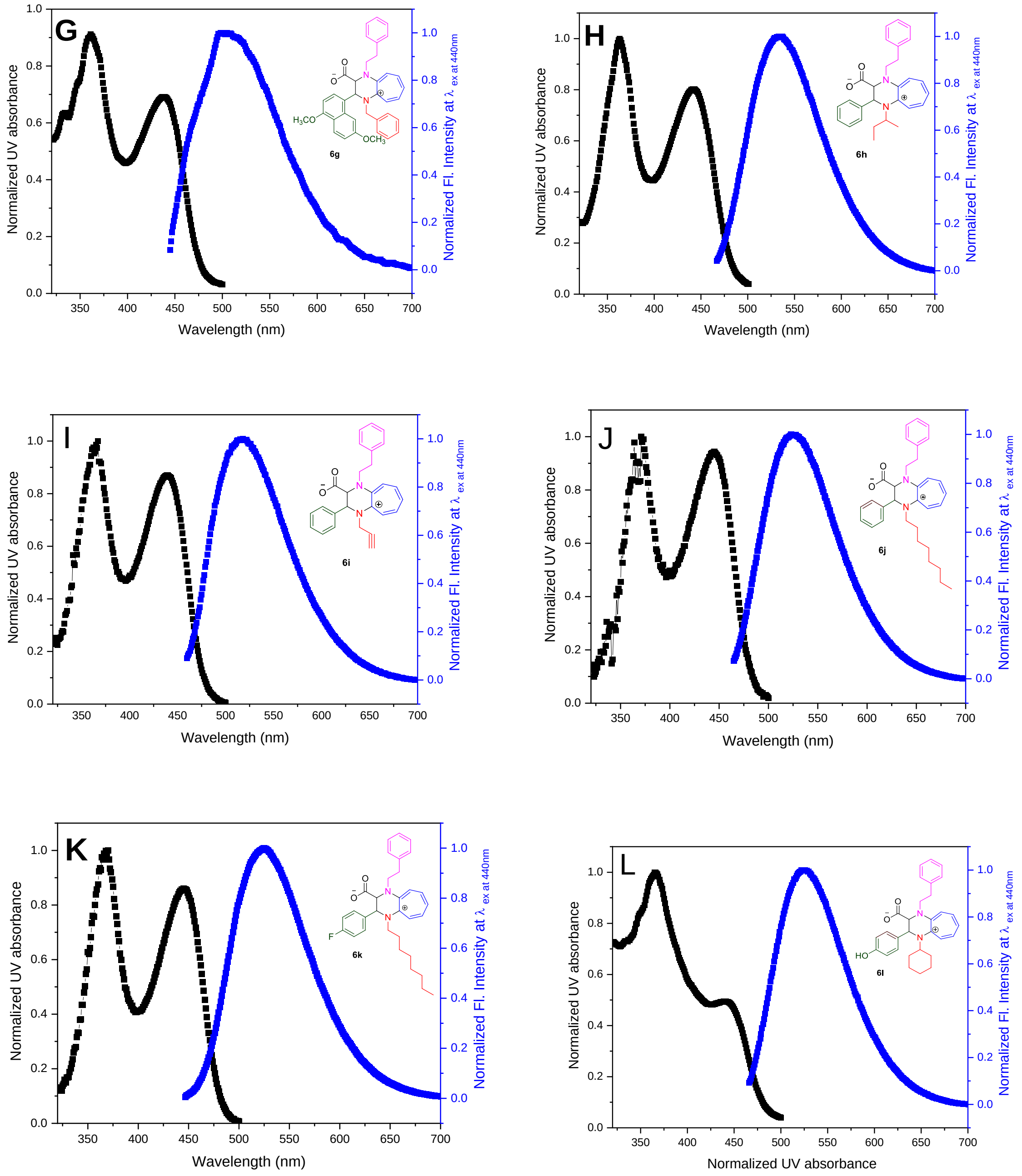

Fig S16. Normalized absorption-emission spectra of cATC derivatives (**6g-6l**) in solvent methanol at $\lambda_{ex,\ 444nm}$. Their concentration is $5x10^{-5}$M in MeOH.

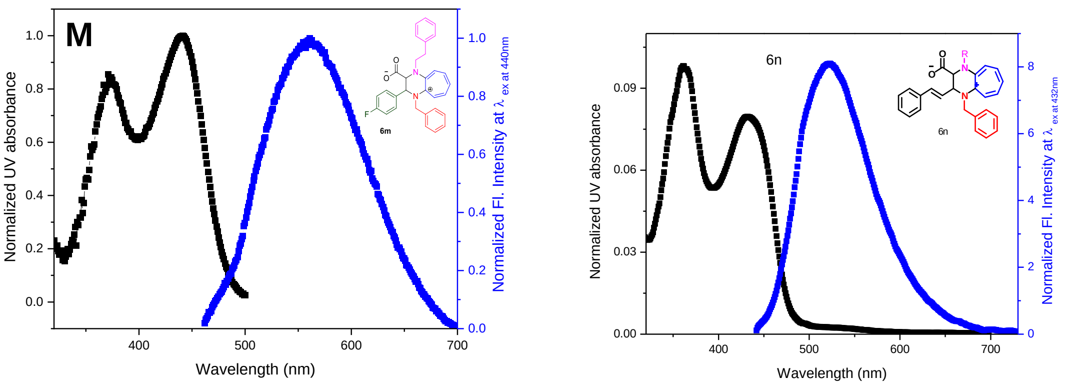

Fig S17: Normalized absorption-emission spectra of cATC derivatives (**6m/6n**) in solvent methanol at $\lambda_{ex,\ 444nm.}$ Their concentration is $5x10^{-5}$M in MeOH.

Table S6. Summary of Photophysical parameters of cATC derivatives (**6a-6m**)

**Entry**	**Compound**	**λ_{Abs} [a,c] (nm)**	**Abs[a]**	**λ em [a] (nm)**	**Σmax[a] (M^{-1} cm^{-1})**	**OD/Abs [a] (nm)**	**Φ_f [b]**
**1**	**6a**	366,443	0.9439	521,524	18878	428	0.111
**2**	**6b**	367,367	0.9793	520,521	19586	432	0.019
**3**	**6c**	368,442	0.8664	520,522	17328	434	0.100
**4**	**6d**	364,441	0.7510	517,520	15020	429	0.092
**5**	**6e**	366,440	0.8471	520,520	16942	436	0.106
**6**	**6f**	364,441	0.8998	520,523	17996	437	0.033
**7**	**6g**	362,440	0.7247	493,498	14492	433	0.007
**8**	**6h**	362,442	0.7743	527,532	15486	427	0.068
**9**	**6i**	363,439	0.8471	515,516	16942	432	0.055
**10**	**6j**	370,444	0.9363	521,522	18726	431	0.131
**11**	**6k**	368,444	0.8336	518,525	16674	435	0.039
**12**	**6l**	367,449	0.4368	522,525	8736	408	0.059
**13**	**6m**	369,440	0.9985	541,558	19970	424	0.006
**14**	**6n**	361,435	0.07872	526	15744	432	0.013

a. All measurements were carried out in methanol, b. Quantum yields were determined by considering cumarin in Methanol as standard reference, c. λex = 440 nm

11. **HOMO-LUMO Calculation of cATC derivatives**

Method: Calculation Gaussian 09, opt=calcfc freq b3lyp/6-31g(d,p), and Gaussian view 06 for visualization.[1]

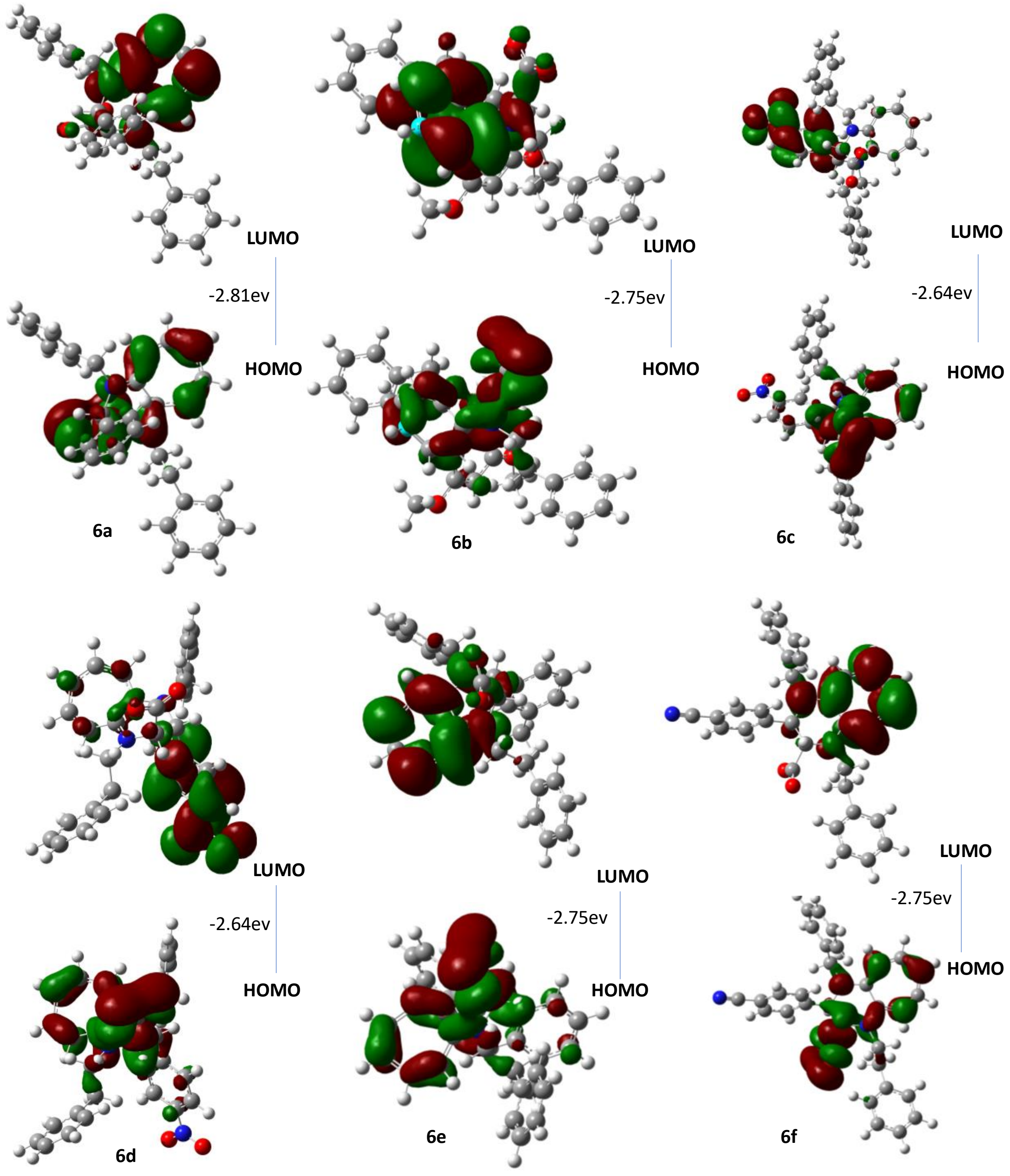

Fig S18. HOMO-LUMO of cATC derivative **6a-6f**

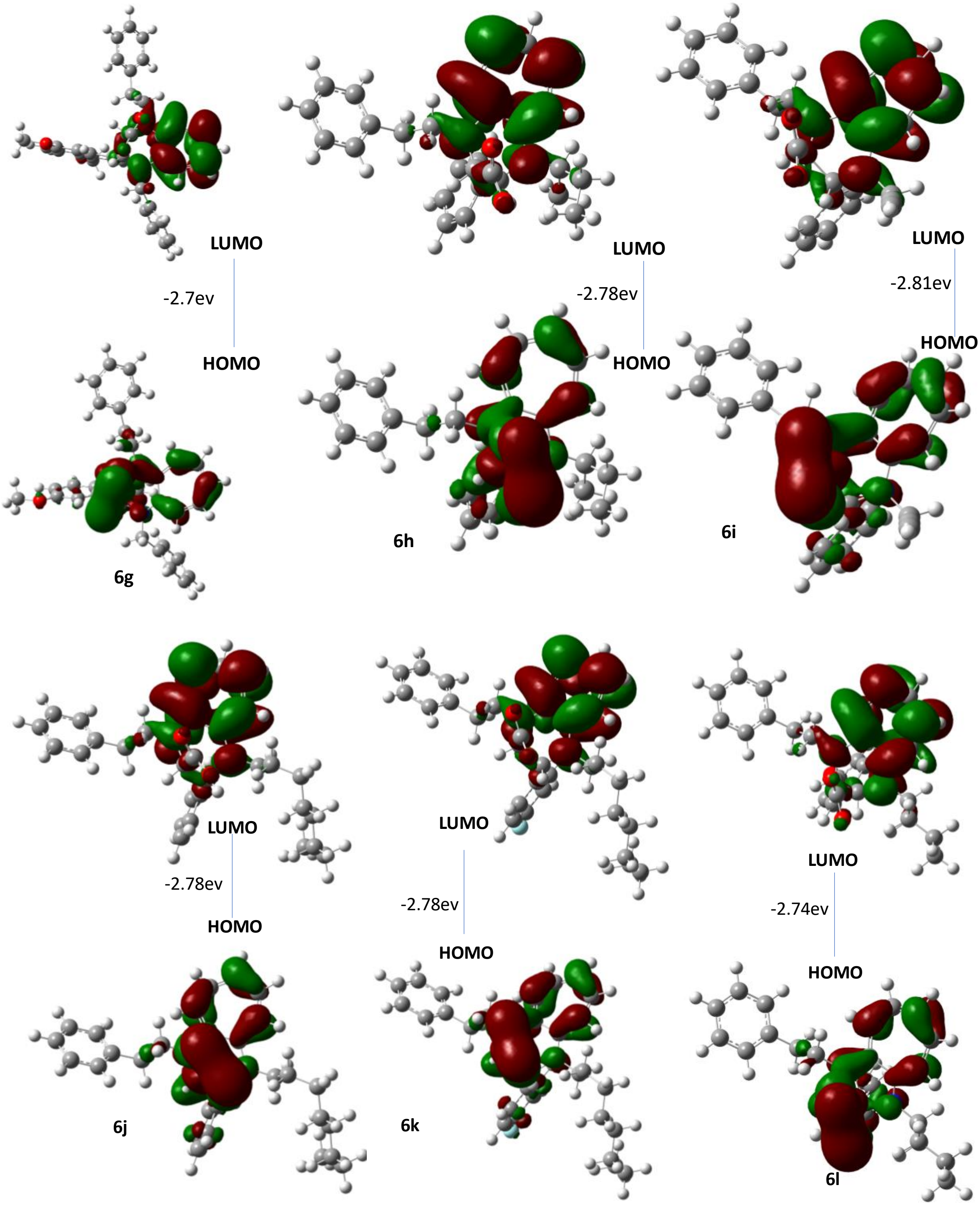

Fig S19. HOMO-LUMO of cATC derivative **6g-6l**

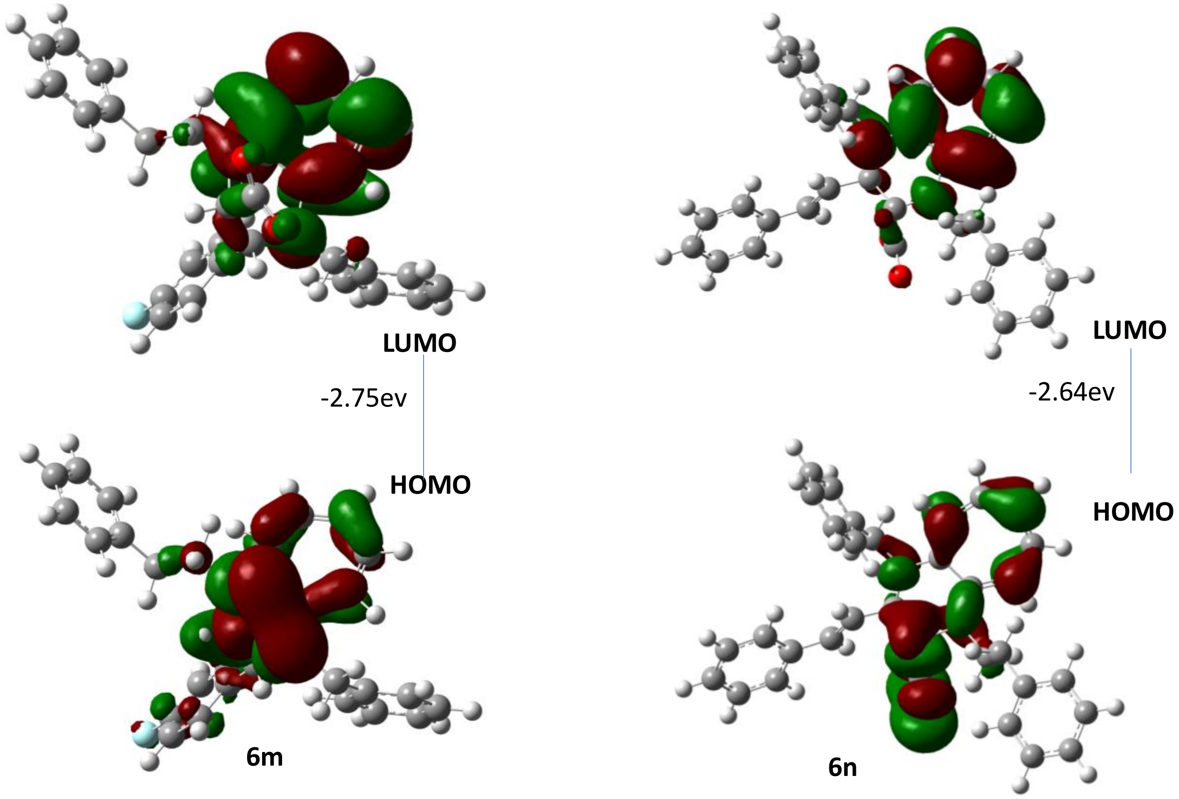

Fig S20. HOMO-LUMO of cATC derivative **6m** and **6n.**

Table S7. Theoratically calculated energy of HOMO-LUMO of optimized cATC derivatives

Compound	E_{HOMO}(eV)	E_{LUMO}(eV)	E_{HOMO}-E_{LUMO} (eV)
**6a**	-4.896	-2.0944	-2.8016
**6b**	-4.8688	-2.1216	-2.7472
**6c**	-5.1952	-2.5568	-2.6384
**6d**	-5.1952	-2.5568	-2.6384
**6e**	-4.8688	-2.1216	-2.7472
**6f**	-5.3856	-2.6384	-2.7472
**6g**	-4.7328	-2.0128	-2.72
**6h**	-4.896	-2.1216	-2.7744
**6i**	-4.8416	-2.2032	-2.6384
**6j**	-4.896	-2.1216	-2.7744
**6k**	-4.9776	-2.2032	-2.7744
**6l**	-4.8416	-2.0944	-2.7472
**6m**	-4.9504	-2.2032	-2.7472
**6n**	-4.9976	-2.312	-2.6656
**6o**	-4.9504	-2.1488	2.8016

12. **Binding studies of cATC derivatives with DNA**

(a) UV-Vis and Fluorescence spectra of cyclic-aminotroponiminium carboxylate (cATC).

UV-Vis Spectra: The UV-Vis spectra of cATC derivatives (**6a/6e/6j/6n**) were recrded at different concentartion of [ct-DNA] near physiologication conditions.

Fluorescence spectra: The fluorescence spectra of cAT derivatives (**6a/6e/6j/6n**) wre recorded at different concentartion of [ct-DNA] at $\lambda_{ex.440nm}$ near physiological conditions.

Displacement Assay: Investigation of cATC derivatives' role in the displacement of intercalative molecule (EtBr)) and groove binding molecule (Hoechst dye).

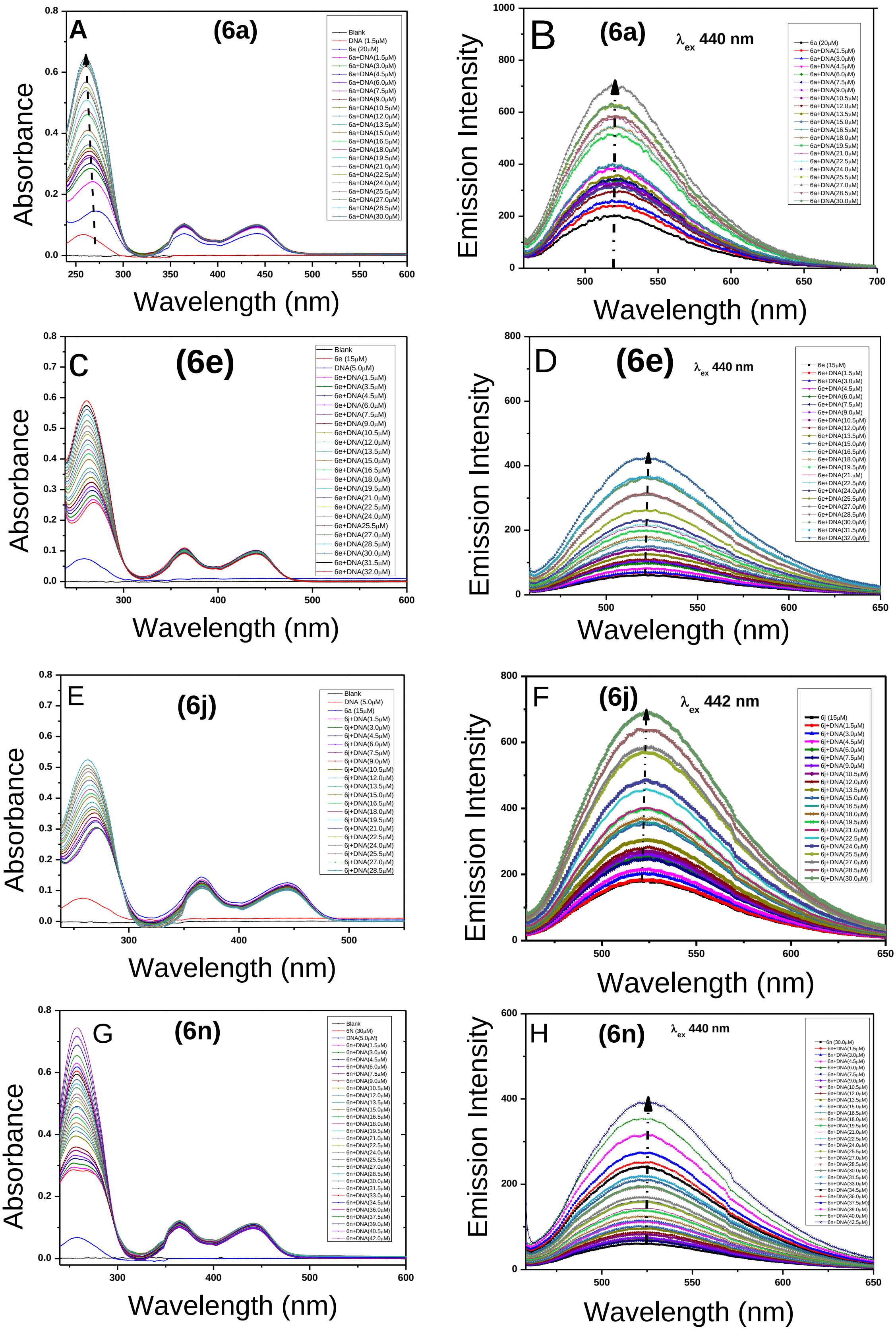

Fig S21. UV-Vis and Fluorescence spectra of UV-Vis spectra of cATC derivatives under different concentration of DNA at pH 7.0 (Room temperature): For cATC **6a** (A &B), cATC **6e** (C & D), cATC **6j** (E & F), and cATC **6n** (G & H)

(b) UV-Vis and Fluorescence spectra of EtBr under different concentrations of ct-DNA (control experiment)

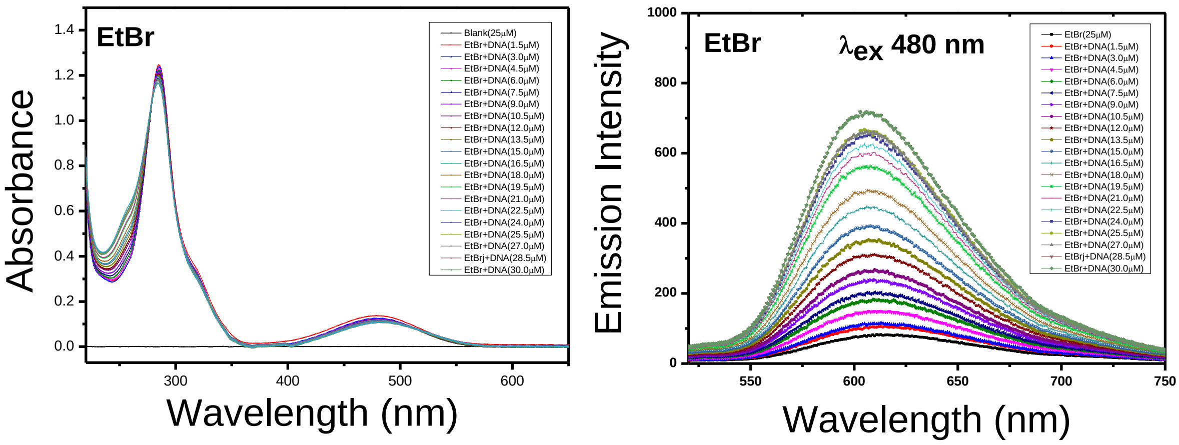

Fig S22. UV-Vis EtBr under different concentration of with ct-DNA (A); and Fluorescence spectra of EtBr under different concentration of with ct-DNA (B).

(c) *Displacement Assay: UV-Vis and Fluorescence spectra of cATC derivative (**6j**) with ct-DNA:EtBr/Hoechst complex*

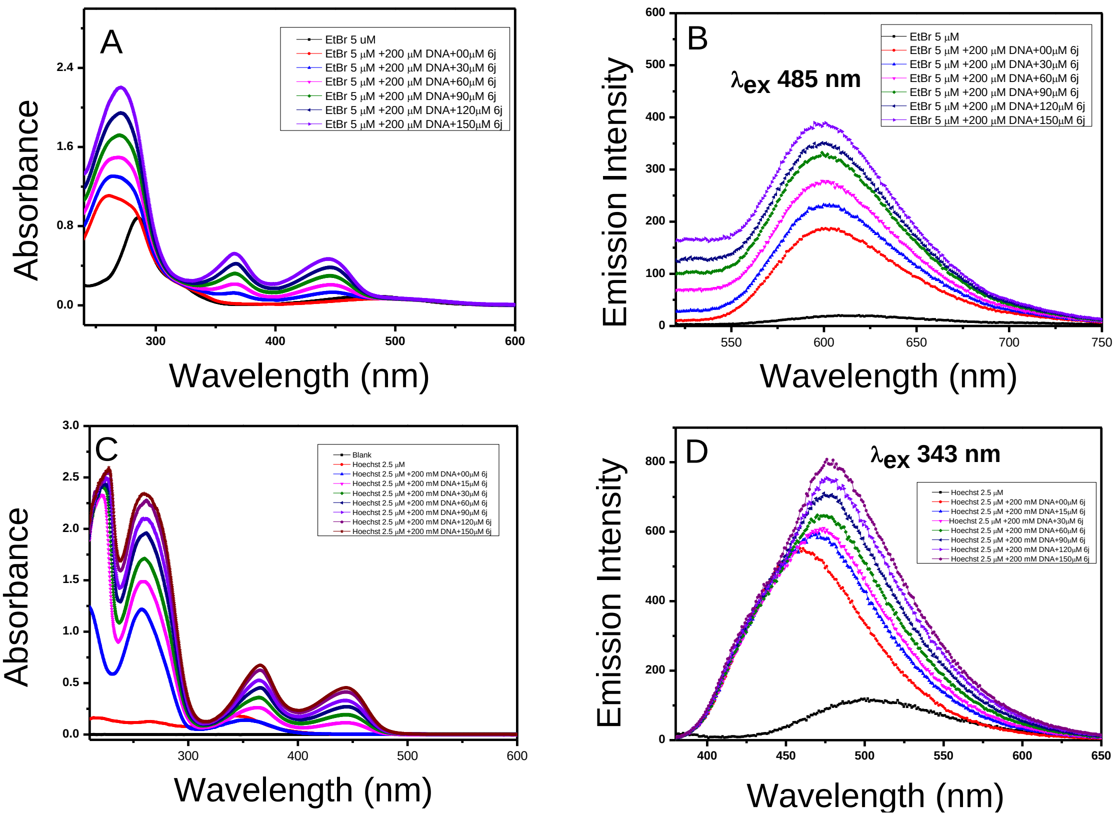

Fig S23. UV-Vis and Fluorescence spectra of *ct*-DNA under different concentration of *cATC* derivatives (**6j**): UV-Vis spectra of ct-DNA:EtBr:**6j** (A); Fluorescence spectra of ct-DNA:EtBr:**6j** (B); and UV-Vis spectra of ct-DNA: Hoechst:**6j** (**C**); Fluorescence spectra of ct-DNA:Hoechst:**6j** (D).

*(d) CD-spectra cyclic-aminotroponimine carboxylates derivatives (**6a/6e/6j/6n**)*

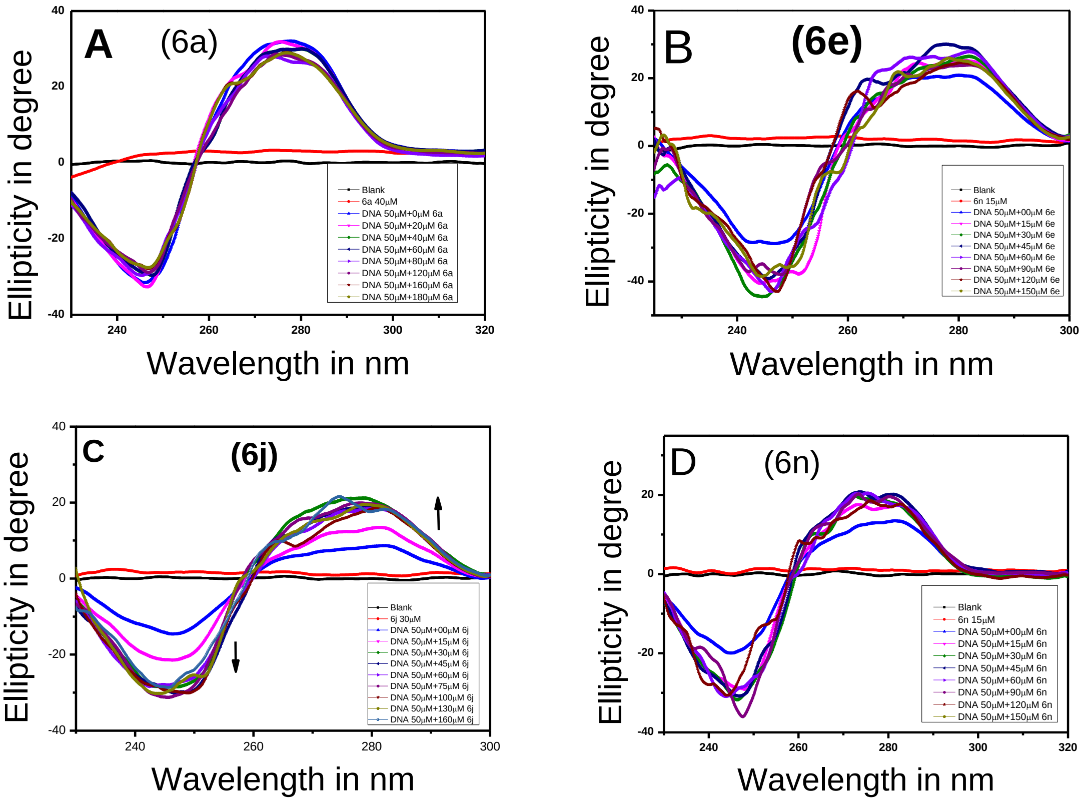

Fig S24. CD-spectra ***cATC*:ct-DNA** under different concentration of DNA at pH 7.0 (Room temperature): CD-spectra of *cATC (**6a**)*:ct-DNA (A); CD-spectra of *cATC (**6e**)*:ct-DNA (B); CD-spectra of cATC (**6j**):ct-DNA (C) and CD-spectra of cATC (**6n**):ct-DNA (D)

(e) Computational studies: Docking studies of cATC derivatives

AutoDock-Vina is well established computational programme for the virtual drug screening of small molecules (ligands) with biomacromolecules (receptors).[4] Herein we planned to examine the interaction of cATC derivatives with crystal structure of DNA duplex (PDB ID 1BNA). We used receptor as DNA (1BNA.pdqt) and ligand (cATC.pdqt) with following center and size: center_x = 14.8, center_y = 20.9, center_z = 8.8; size_x = 60, size_y = 60, size_z = 60; exhaustiveness = 8; We have confirmed of the single crystal structure of cATC derivatives (**6a/6d/6k**) by X-ray studies. We used these derivatives as ligands for the docking with crystal structure of dodecamer DNA duplex (PDB ID: 1BNA). The pdb files of cATC ligands

(**6a/6d/6k**) were generated by using softwares Diamond-3 and pyMOL (educational licences). The cATC ligand-DNA docked structures are provided in Fig S49-51 and their binding affinities are summarise in Table S7.

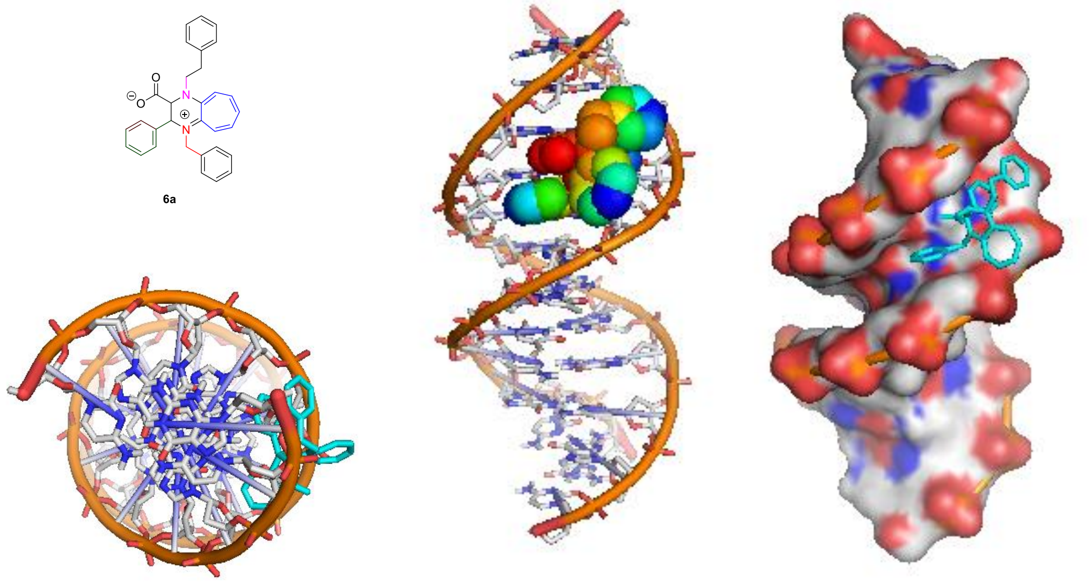

Fig S25. Binding site of cATC derivative **6a** with DNA duplex structure (PDB ID 1BNA).

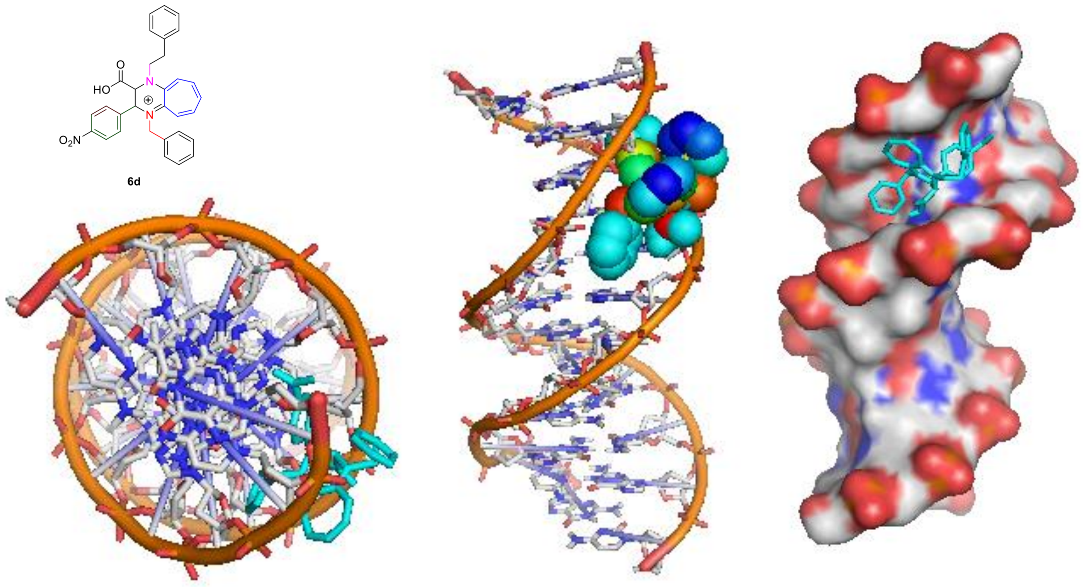

Fig S26. Binding site of cATC derivative **6d** with DNA duplex structure (PDB ID 1BNA).

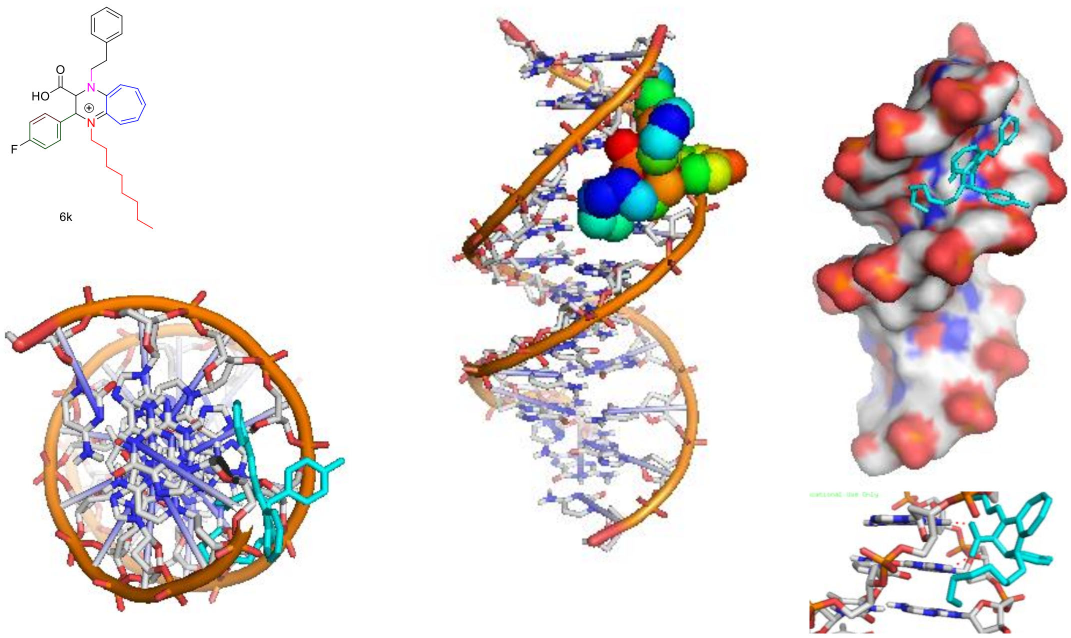

Fig S27. Binding site of *cATC* derivative **6a** with DNA duplex structure (PDB ID 1BNA).

Table S8. Docking results of cATC derivatives with DNA duplex structures

Entry	cATC Molecule	Binding affinity (Kcal/mol)	Binding Site of DNA duplex (PDB ID 1BNA)
1	**6a**	-6.3	*Minor groove*
2	**6d**	-7.7	*Minor groove*
3	**6k**	-6.7	*Minor groove* via hydrogen bonding between Carboxylic acid-(-C=O) and guaninyl-N-H

(e) Binding of cATC derivatives with DNA by Agarose gel and, Microscale thermophoresis

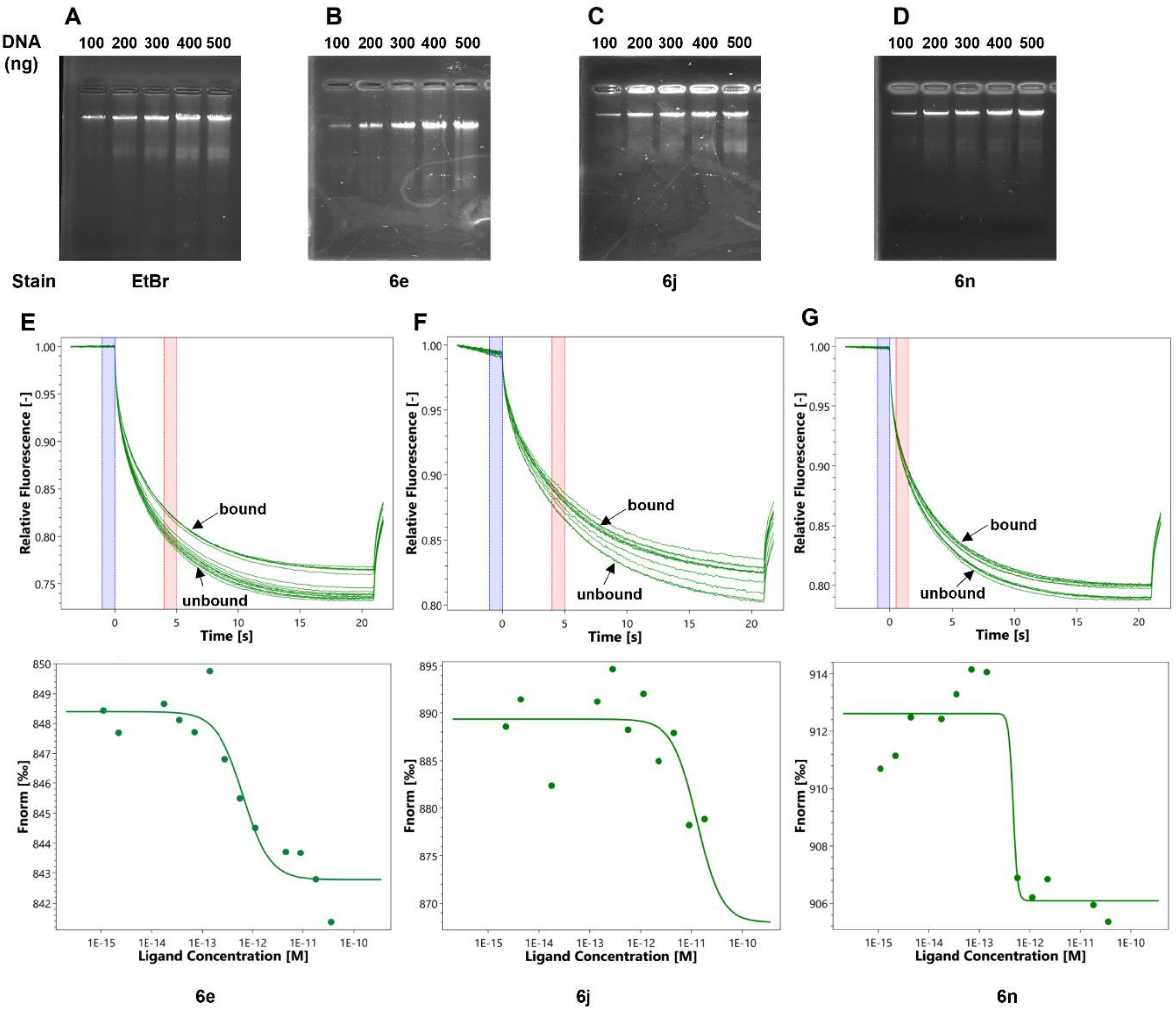

Fig S28: Binding of cATC derivatives with DNA. A-D, Agarose gel (1%) electrophoresis images of human genomic DNA (isolated from MDA-MB-231 cell line) stained with 0.5µg/ml of (**A**) EtBr, (**B**) **6e**, (**C**) **6j**, and (**D**) **6n**. **E-G,** Microscale thermophoresis traces graph and Hill's coefficient analysis of **6e** (**E**), **6j** (**F**), and **6n** (**G**).

13. **Cell viability studies of cATC derivatives**

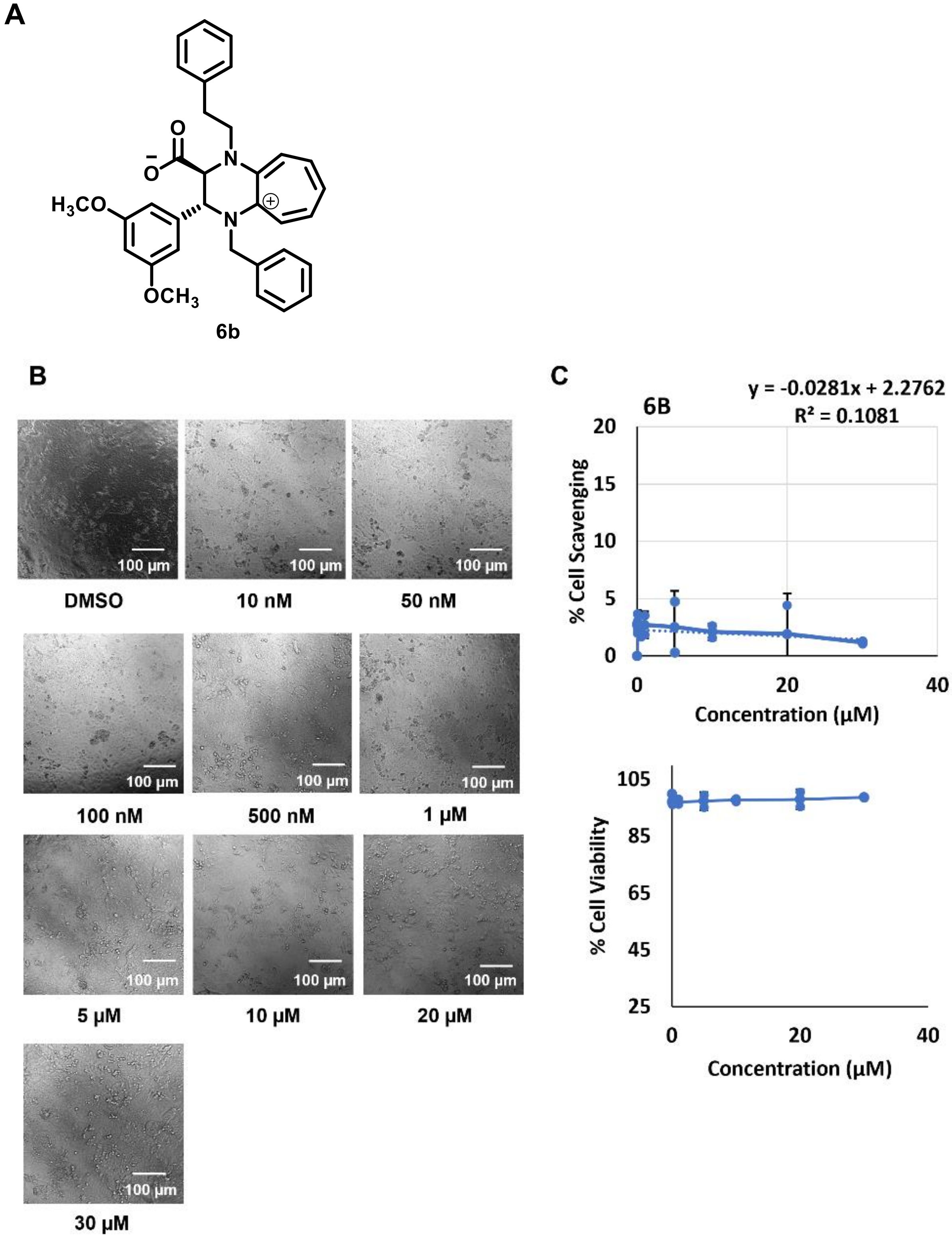

Fig S29. Effect of cATC derivatives (6b) on cell growth: a, HeLa cells were treated with indicated amount of **6b** for 18 hours **b,** Cell scavenging, and cell viability was determined relative to the cells treated with solvent (DMSO) only; $N = 2$; mean ± SD.

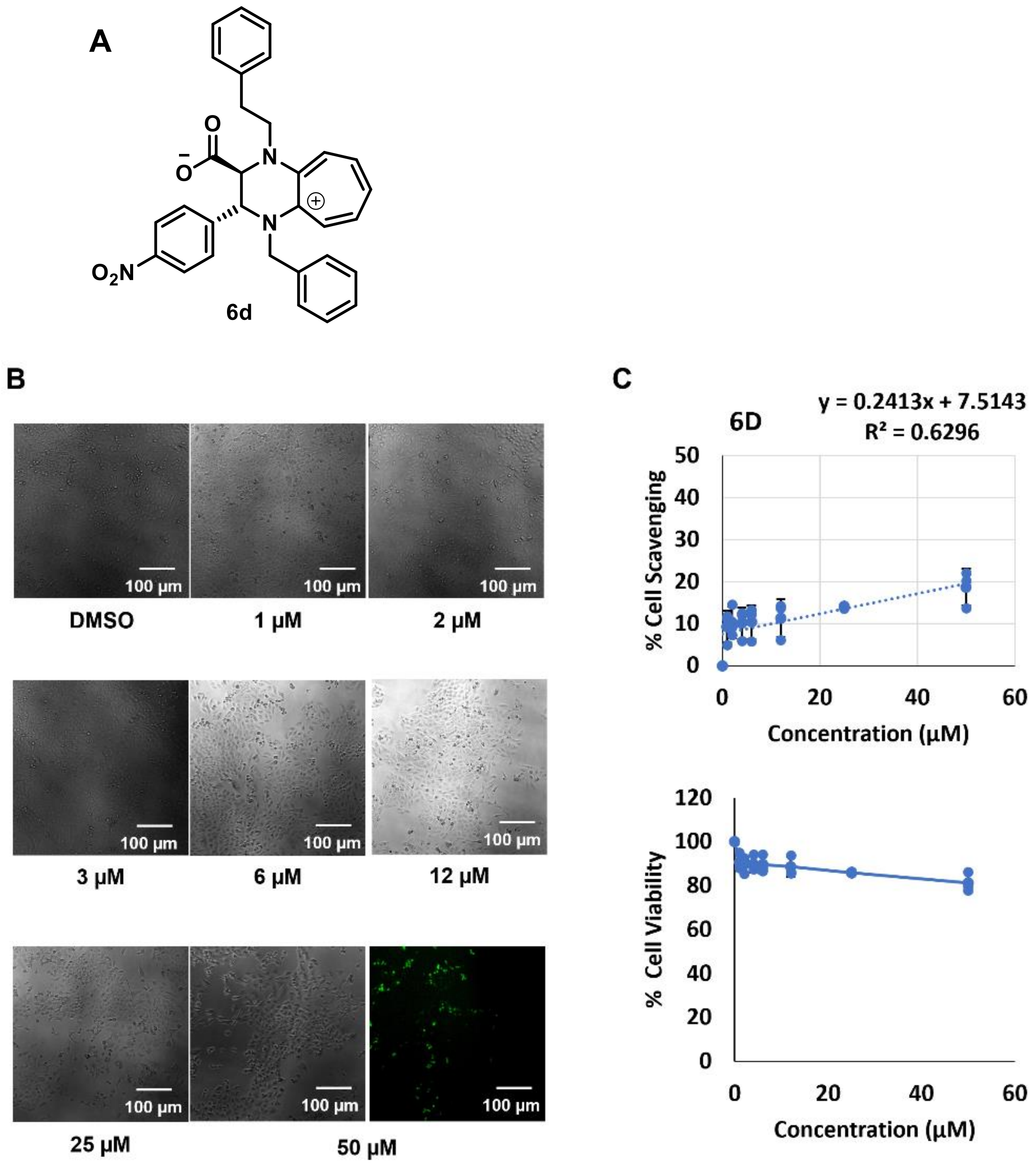

Fig S30. **Effect of cATC derivatives (6d) on cell growth: a,** HeLa cells were treated with indicated amount of **6d** for 18 hours **b**, Cell scavenging, and cell viability was determined relative to the cells treated with solvent (DMSO) only; $N = 3$; mean ± SD.

A

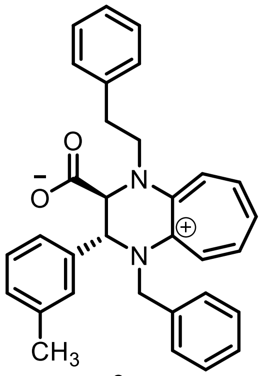

6e

B

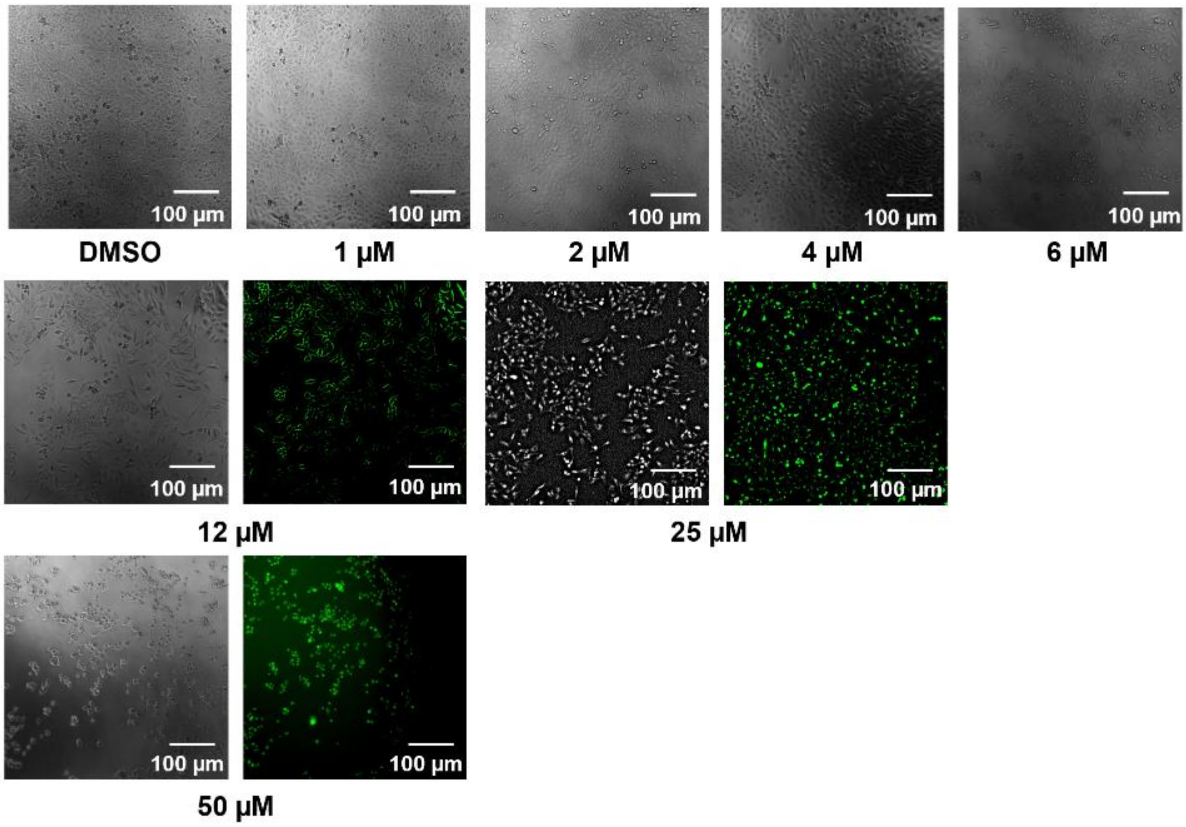

Fig S31. **Effect of cATC derivatives (6e) on cell growth: a,** HeLa cells were treated with indicated amount of **6e** for 18 hours; $N = 3$; mean ± SD.

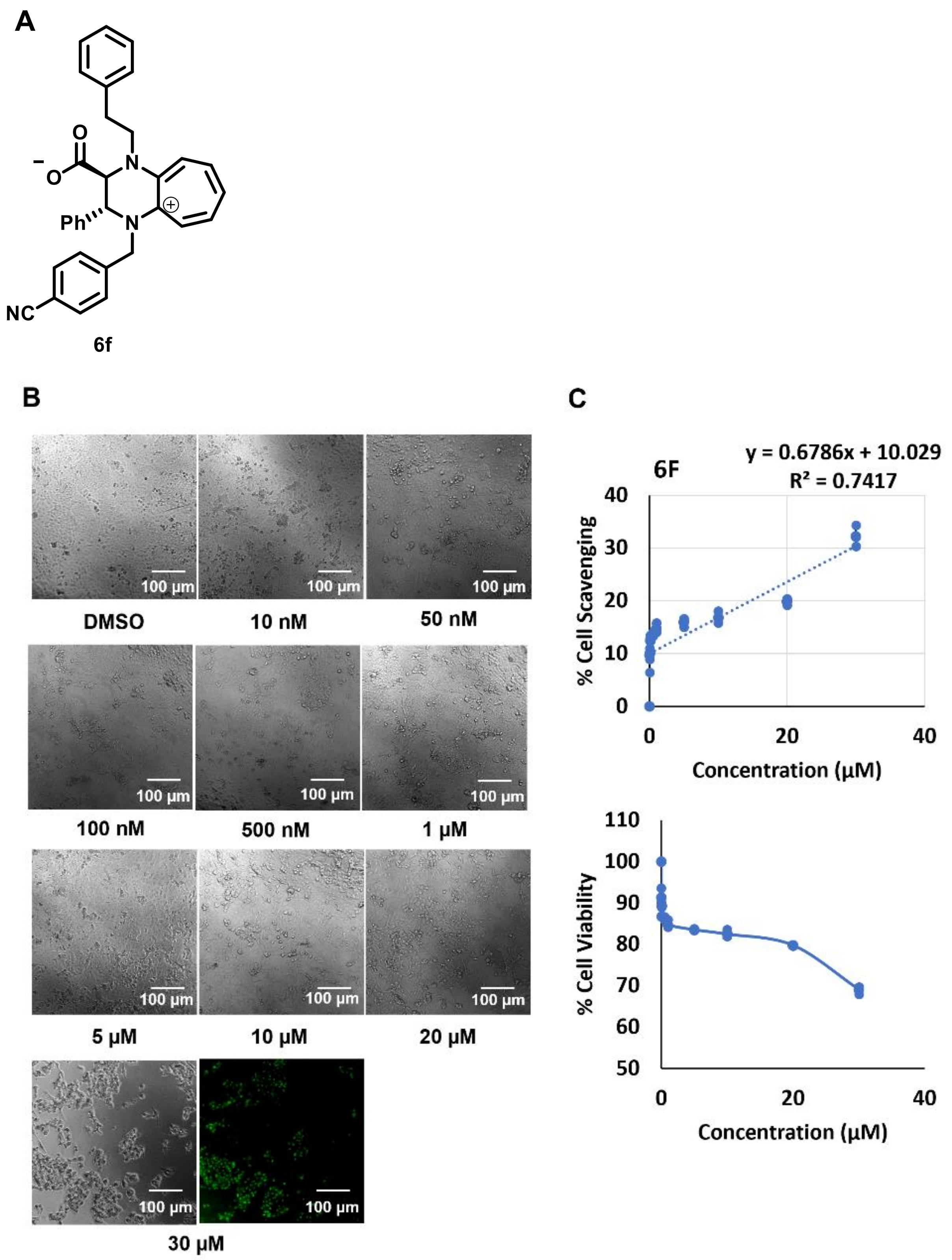

Fig S32. Effect of cATC derivatives (6f) on cell growth: a, HeLa cells were treated with indicated amount of **6f** for 18 hours **b**, Cell scavenging, and cell viability was determined relative to the cells treated with solvent (DMSO) only; *N* = 3; mean ± SD.

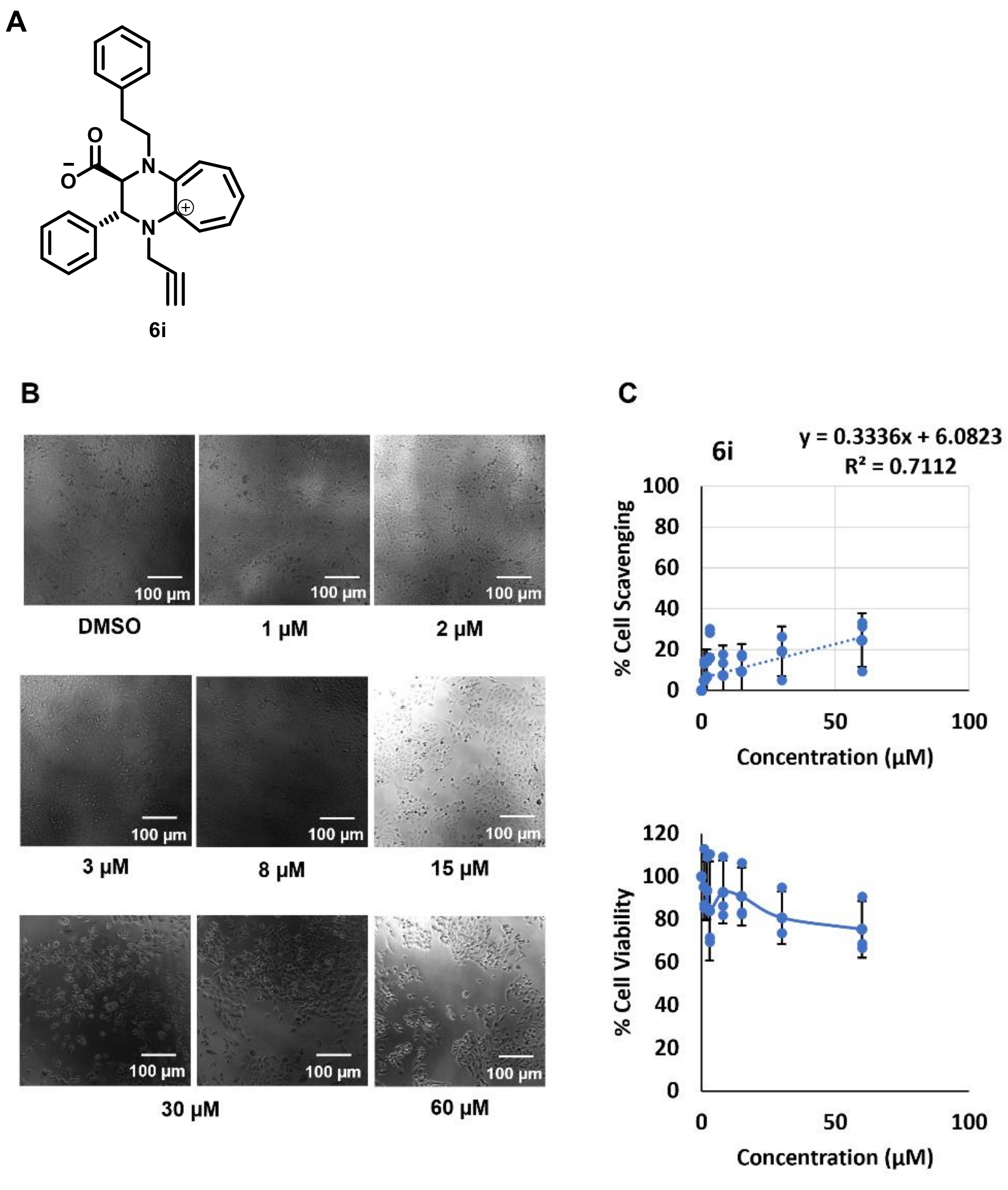

Fig S33. Effect of cATC derivatives (6i) on cell growth: a, HeLa cells were treated with indicated amount of **6i** for 18 hours **b**, Cell scavenging, and cell viability was determined relative to the cells treated with solvent (DMSO) only; $N = 3$; mean ± SD.

A

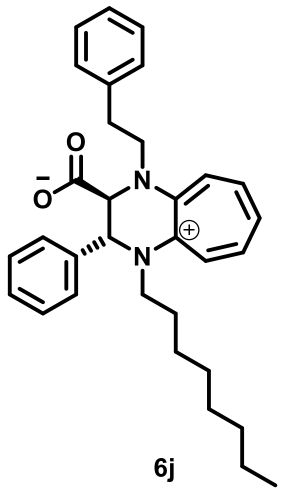

B

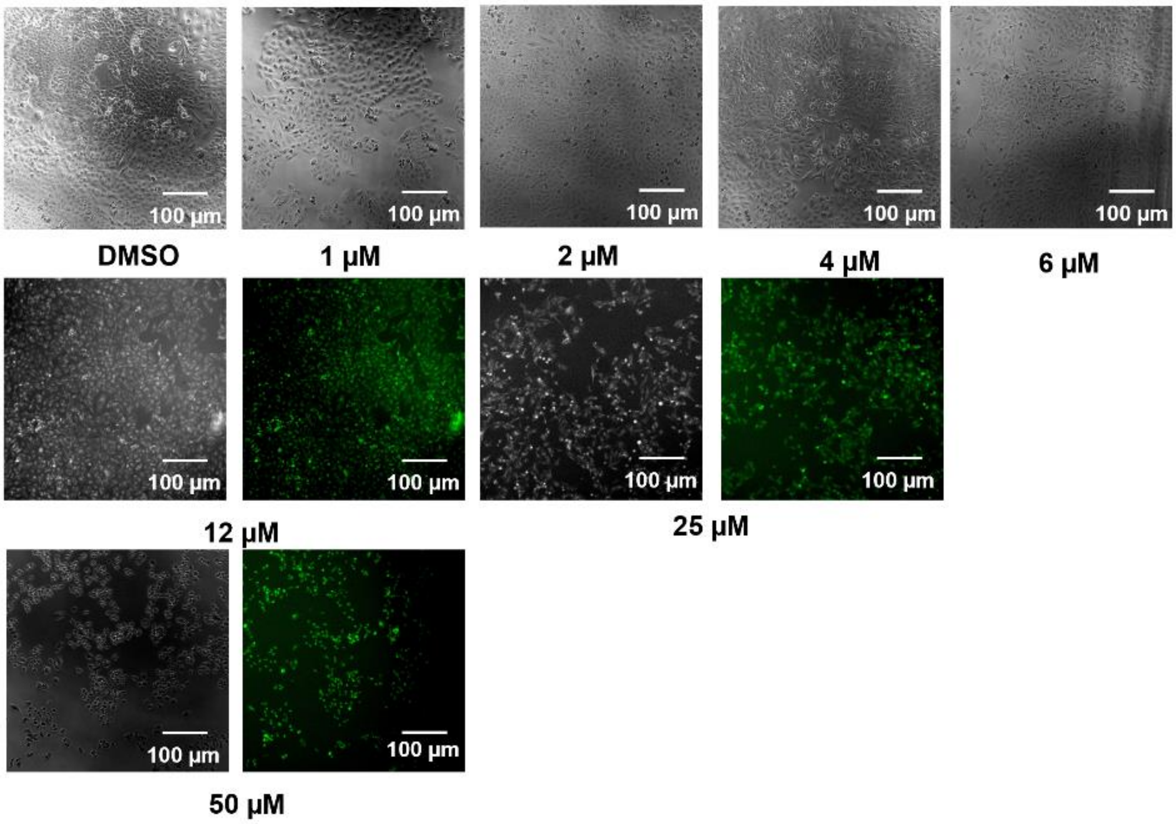

Fig S34. Effect of cATC derivatives (6j) on cell growth: a, HeLa cells were treated with indicated amount of **6j** for 18 hours; *N* = 3; mean ± SD.

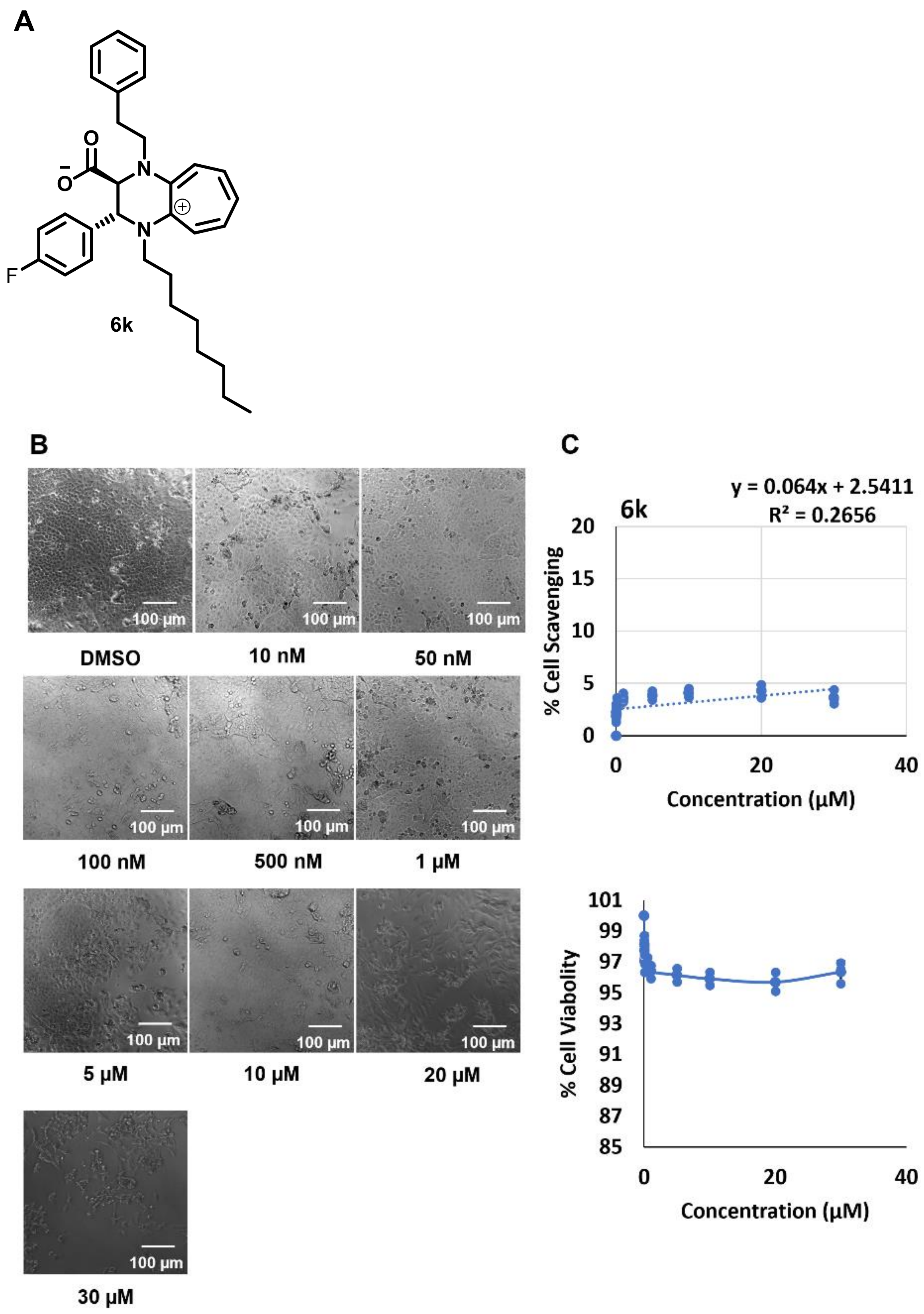

Fig S35. **Effect of cATC derivatives (6k) on cell growth: a,** HeLa cells were treated with indicated amount of **6k** for 18 hours **b**, Cell scavenging, and cell viability was determined relative to the cells treated with solvent (DMSO) only; $N = 3$; mean ± SD.

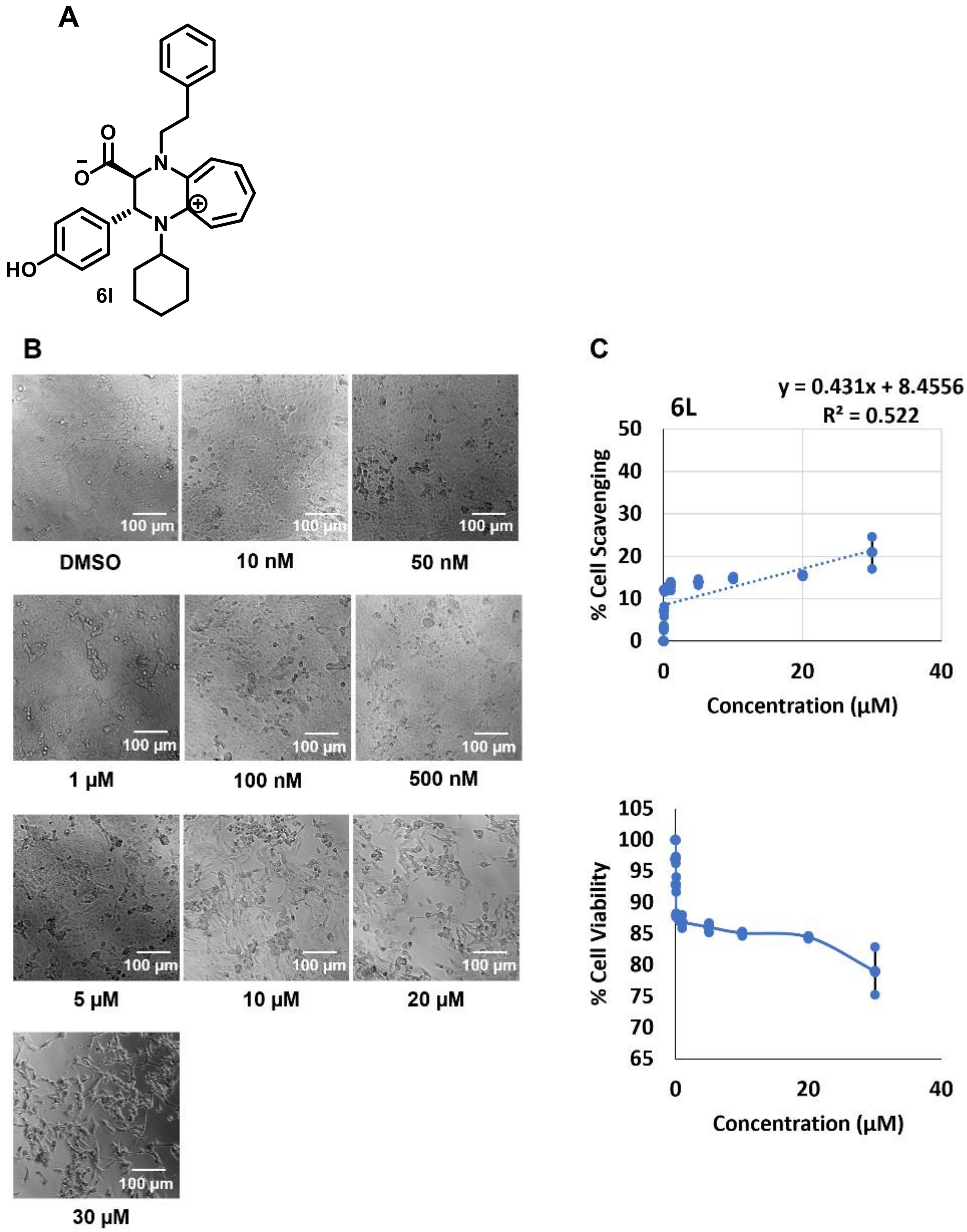

Fig S36. **Effect of cATC derivatives (6l) on cell growth: a,** HeLa cells were treated with indicated amount of **6L** for 18 hours **b**, Cell scavenging, and cell viability was determined relative to the cells treated with solvent (DMSO) only; $N = 3$; mean ± SD.

A

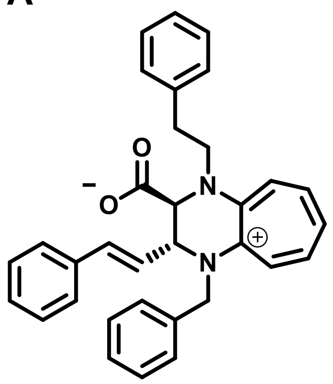

6n

B

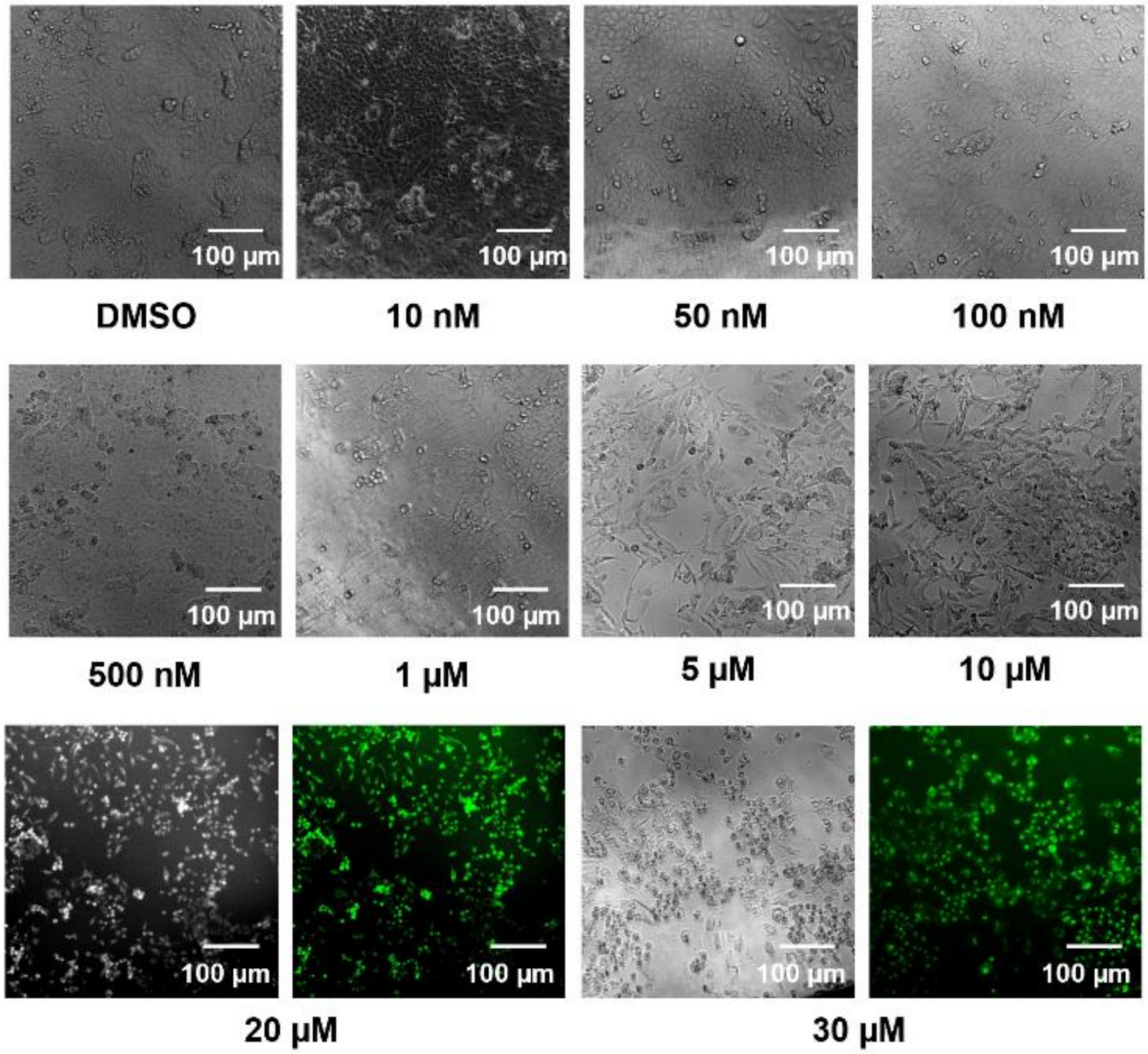

Fig S37. Effect of cATC derivatives (6n) on cell growth: a, HeLa cells were treated with indicated amount of **6n** for 18 hours **b**, Cell scavenging, and cell viability was determined relative to the cells treated with solvent (DMSO) only; $N = 3$; mean ± SD.

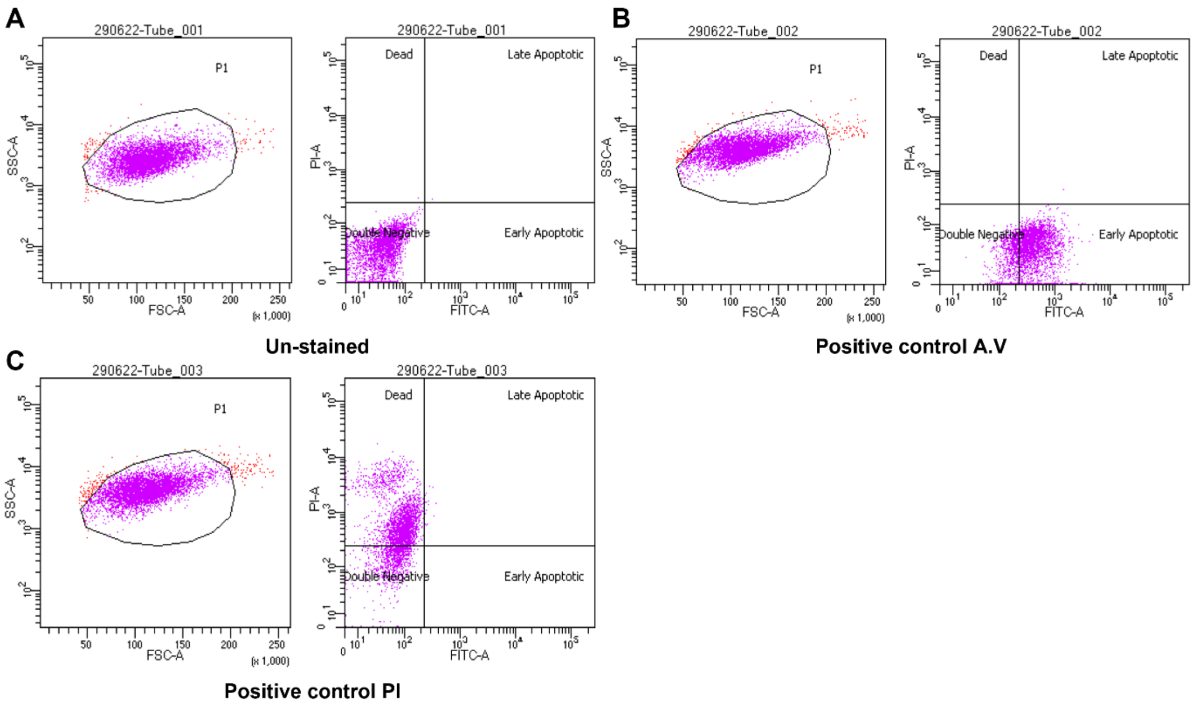

Fig S38. Flow cytometric analysis of apoptosis controls in HeLa cells. HeLa cells were treated under UV light exposure for 30 minutes. **a-c** Cells were stained with FITC-conjugated Annexin V (AV) and Propidium Iodide (PI), and analysed by flow cytometer. For negative and positive control cells were exposed to UV light for 30 min and stained with PI or FITC-conjugated AV., The scatter plots for cells unstained (**a**), positive control- PI (**b**), positive control- AV (**c**). The early apoptotic cells (FITC-AV^{+ve} /PI^{-ve}) are shown in lower right quadrant. The late apoptotic cells (FITC-AV^{+ve} /PI^{+ve}) are shown in upper right quadrant.

Table S9. IC_{50} values for HeLa cells of cATC derivatives

Compound name	IC_{50} concentration in (μM)
**6b**	1698.356
**6d**	176.070
**6e**	25.301
**6f**	58.90
**6i**	131.6478
**6j**	19.43
**6k**	741.545
**6l**	96.390
**6n**	19.40

Table S10. IC_{50} values for HEK293 cells of cATC derivatives

Compound name	IC_{50} concentration in (μM)
**6e**	25.093
**6j**	88.49
**6n**	56.263

14. **Supplementary References**

1. Balachandra, C.; Sharma, N. K., Instability of Amide Bond Comprising the 2-Aminotropone Moiety: Cleavable under Mild Acidic Conditions. *Org. Lett.* **2015,** *17* (16), 3948-3951.

2. Balachandra, C.; Sharma, N. K., Direct/Reversible Amidation of Troponyl Alkylglycinates via Cationic Troponyl Lactones and Mechanistic Insights. *ACS Omega* **2018,** *3* (1), 997-1013.

3. Poeschl, A.; Mountford, D., A facile manganese dioxide mediated oxidation of primary benzylamines to benzamides. *Org. Biomol. Chem.* **2014,** *12* (36), 7150-7158.

4. (a) Trott, O.; Olson, A. J., AutoDock Vina: improving the speed and accuracy of docking with a new scoring function, efficient optimization, and multithreading. *Journal of computational chemistry* **2010,** *31* (2), 455-461.

(b) Grimme, S.; Antony, J.; Ehrlich, S.; Krieg, H. J. Chem. Phys. 2010, 132, 154104.

(a) Hariharan, P. C.; Pople, J. A. Theor. Chim. Acta 1973, 28, 213. (c) Hehre, W. J.; Ditchfield, R.; Pople, J. A. J. Chem. Phys. 1972, 56, 2257.

9d0 Gaussian 16, Revision C.01, Frisch, M. J.; Trucks, G. W.; Schlegel, H. B.; Scuseria, G. E.; Robb, M. A.; Cheeseman, J. R.; Scalmani, G.; Barone, V.; Petersson, G. A.; Nakatsuji, H.; Li, X.; Caricato, M.; Marenich, A. V.; Bloino, J.; Janesko, B. G.; Gomperts, R.; Mennucci, B.; Hratchian, H. P.; Ortiz, J. V.; Izmaylov, A. F.; Sonnenberg, J. L.; Williams-Young, D.; Ding, F.; Lipparini, F.; Egidi, F.; Goings, J.; Peng, B.; Petrone, A.; Henderson, T.; Ranasinghe, D.; Zakrzewski, V. G.; Gao, J.; Rega, N.; Zheng, G.; Liang, W.; Hada, M.; Ehara, M.; Toyota, K.; Fukuda, R.; Hasegawa, J.; Ishida, M.; Nakajima, T.; Honda, Y.; Kitao, O.; Nakai, H.; Vreven, T.; Throssell, K.; Montgomery, J. A., Jr.; Peralta, J. E.; Ogliaro, F.; Bearpark, M. J.; Heyd, J. J.; Brothers, E. N.; Kudin, K. N.; Staroverov, V. N.; Keith, T. A.; Kobayashi, R.; Normand, J.; Raghavachari, K.; Rendell, A. P.; Burant, J. C.; Iyengar, S. S.; Tomasi, J.; Cossi, M.; Millam, J. M.; Klene, M.; Adamo, C.; Cammi, R.; Ochterski, J. W.; Martin, R. L.; Morokuma, K.; Farkas, O.; Foresman, J. B.; Fox, D. J. Gaussian, Inc., Wallingford CT, 2016.
