# Supplementary material for: One-pot synthesis of cyclic-aminotropiminium carboxylate derivatives with DNA binding and anticancer properties
Source: Commun Chem. 2022 Dec 27;5:179. doi: 10.1038/s42004-022-00798-x (PMC9814901; doi:10.1038/s42004-022-00798-x)
Supplement: Supplementary file 3 — Description of Additional Supplementary Files [file 42004_2022_798_MOESM3_ESM.pdf]

Description of Additional Supplementary Files

File name: Supplemental Data 1
Description: NMR, HRMS and FT-IR spectra

File name: Supplemental Data 2
Description: Cif file of crystal compound **2**

File name: Supplemental Data 3
Description: Cif file of crystal cATC compound **6a**

File name: Supplemental Data 4
Description: CheckCif file of crystal cATC compound **6d**

File name: Supplemental Data 5
Description: Cif file of crystal cATC compound **6k**

File name: Supplemental Data 6
Description: Computational studies results

File name: Supplemental Data 7
Description: Western blot images
