# Supplementary material for: One-pot synthesis of cyclic-aminotropiminium carboxylate derivatives with DNA binding and anticancer properties
Source: Commun Chem. 2022 Dec 27;5:179. doi: 10.1038/s42004-022-00798-x (PMC9814901; doi:10.1038/s42004-022-00798-x)
Supplement: Supplementary file 4 — Supplementary Data 1 [file 42004_2022_798_MOESM4_ESM.pdf]

Supplemental Material

One-Pot synthesis of cyclic-aminotropiminium carboxylate derivatives with DNA binding and anticancer properties

Bibhuti Bhusana Palai[a,c,#], Saket Awadhesbhai Patel[b,c,#], Nagendra K. Sharma*[,a.c], Manjusha Dixit*[,b,c]

[#]Authors equally contributed

*Corresponding Authors

[a]School of Chemical Sciences, National Institute of Science Education and Research (NISER) Bhubaneswar, PO: Jatani-752050, Odisha, India, Phone no. +91-674-249-4141; E-mail: nagendra@niser.ac.in

[b]School of Biological Sciences, National Institute of Science Education and Research (NISER) Bhubaneswar, PO: Jatani-752050, Odisha, India, Phone no. +91-674-249-4195; E-mail: manjusha@niser.ac.in

[c]Homi Bhabha National Institute, Training School Complex, Anushaktinagar, Mumbai 400094, India

Contents Page No.

1. NMR($^{1}H/^{13}C$) and HRMS of Cyclic-Aminotropiminium Carboxylate (**6a**) S3
2. NMR($^{1}H/^{13}C$) and HRMS of Cyclic-Aminotropiminium Carboxylate (**6b**) S5
3. NMR($^{1}H/^{13}C$) and HRMS of Cyclic-Aminotropiminium Carboxylate **(6c)** S7
4. NMR($^{1}H/^{13}C$) and HRMS of Cyclic-Aminotropiminium Carboxylate (**6d**) S9
5. NMR($^{1}H/^{13}C/^{13}C$DEPT -135) and HRMS of Cyclic-Aminotropiminium Carboxylate (**6e**) S11
6. NMR(1H/13C) and HRMS of Cyclic-Aminotropiminium Carboxylate **(6f)** S14
7. NMR($^{1}H/^{13}C$) and HRMS of Cyclic-Aminotropiminium Carboxylate **(6g)** S16
8. NMR($^{1}H/^{13}C/^{13}C$DEPT) and HRMS of Cyclic-Aminotropiminium Carboxylate **(6h)** S18
9. NMR($^{1}H/^{13}C$) and HRMS of Cyclic-Aminotropiminium Carboxylate **(6i)** S21
10. NMR($^{1}H/^{13}C$) and HRMS of Cyclic-Aminotropiminium Carboxylate **(6j)** S23
11. NMR($^{1}H/^{13}C/^{13}C$DEPT) and HRMS of Cyclic-Aminotropiminium Carboxylate **(6k)** S25
12. NMR(1H/13C) and HRMS of Cyclic-Aminotropiminium Carboxylate **(6l)** S28
13. NMR($^{1}H/^{13}C$) and HRMS of Cyclic-Aminotropiminium Carboxylate **(6m)** S30
14. NMR($^{1}H/^{13}C$) and HRMS of Cyclic-Aminotropiminium Carboxylate **(6n)** S32
15. NMR($^{1}H/^{13}C$) and HRMS of Cyclic-Aminotropiminium Carboxylate **(6o)** S34
1. FT-IR spectra of 6a-6o S38

1. NMR($^{1}H/^{13}C$) and HRMS of Cyclic-Aminotropiminium Carboxylate **(6a)**

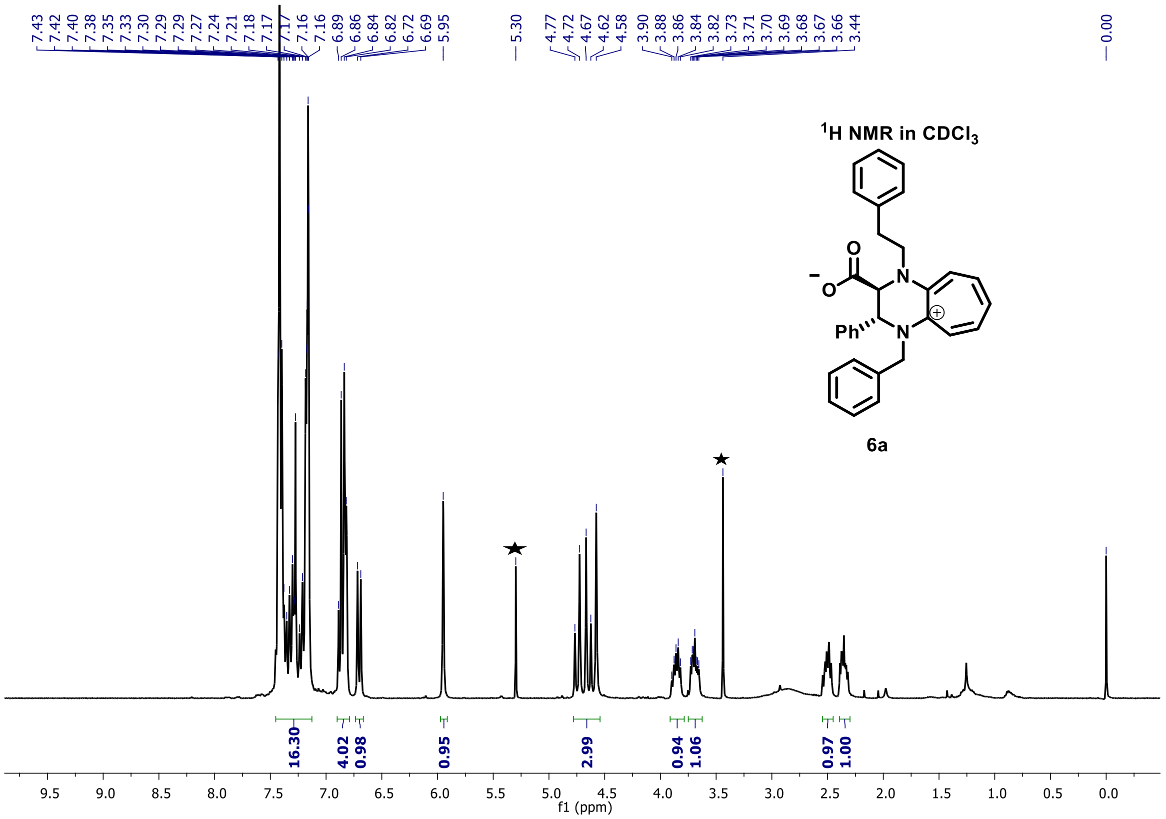

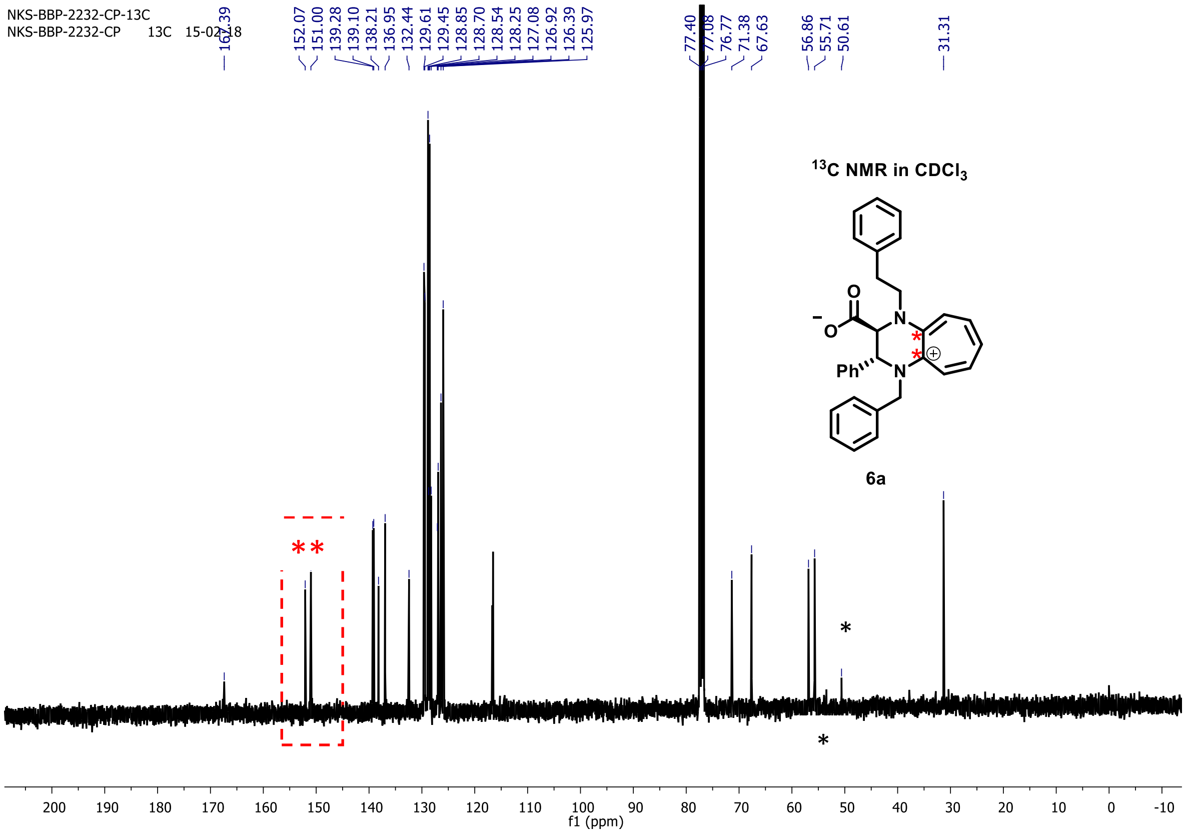

*Residual solvents and impurity grease

Fig S1. ^{1}H-/^{13}C-NMR spectra of **(6a)** in $CDCl_3$

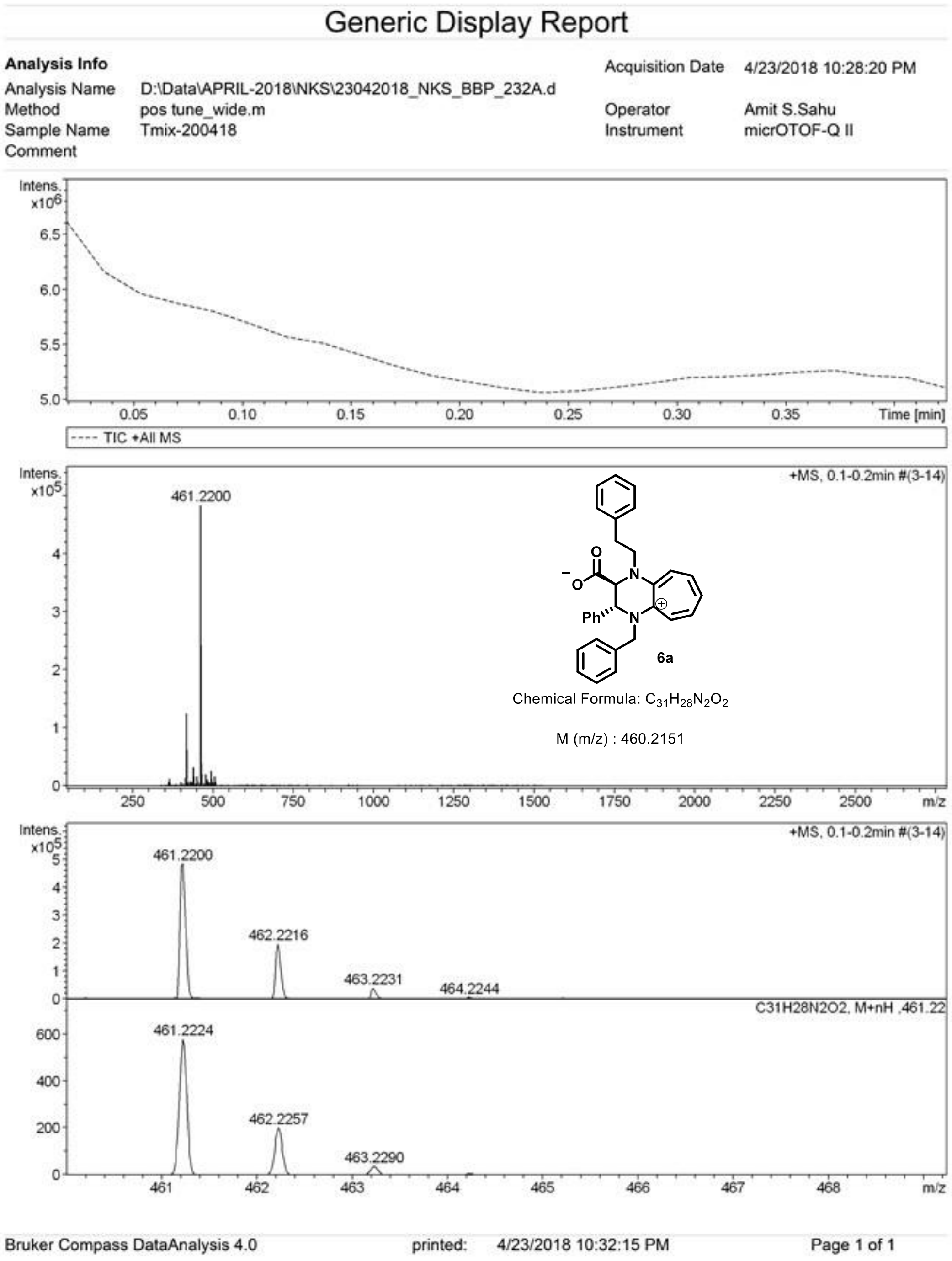

Fig S2. ESI/HRMS spectra of compound **(6a)**

2. NMR($^1H/^{13}C$) and HRMS of Cyclic-Aminotropiminium Carboxylate (**6b**)

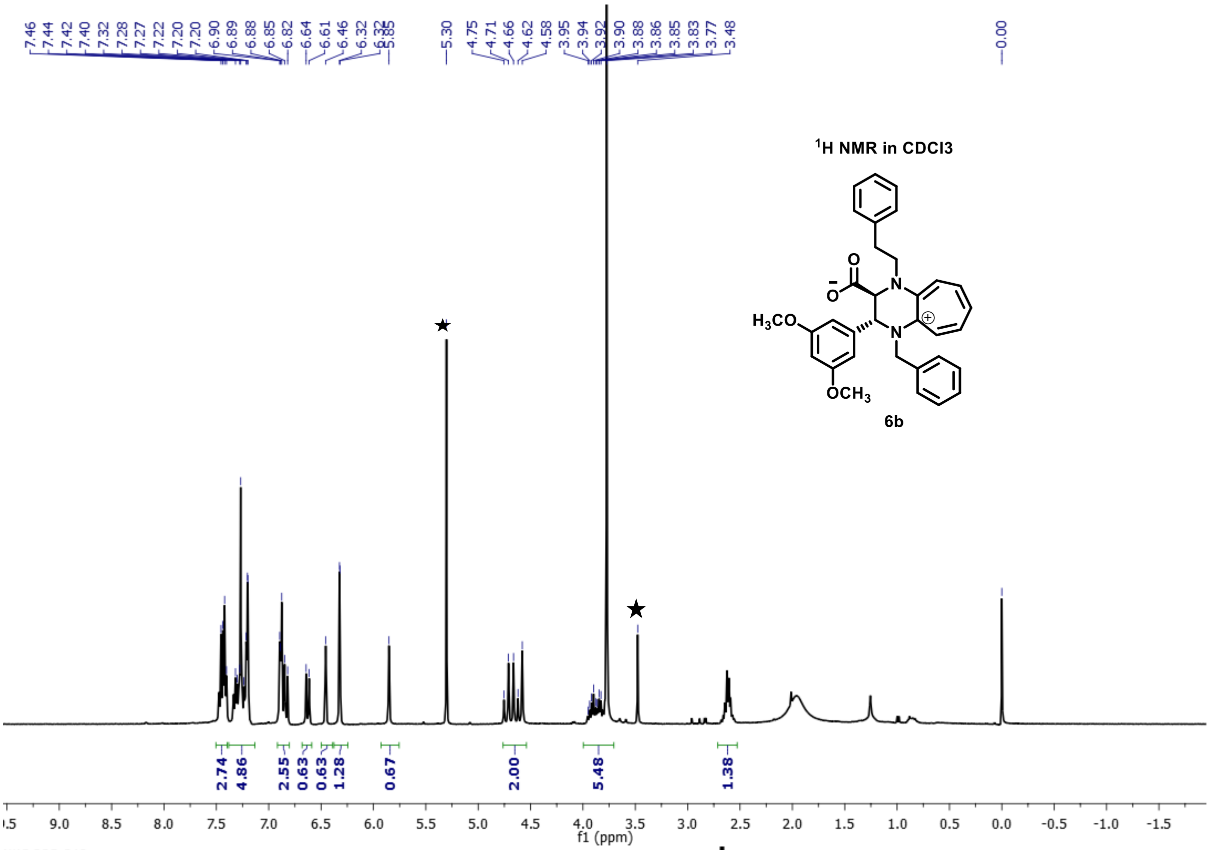

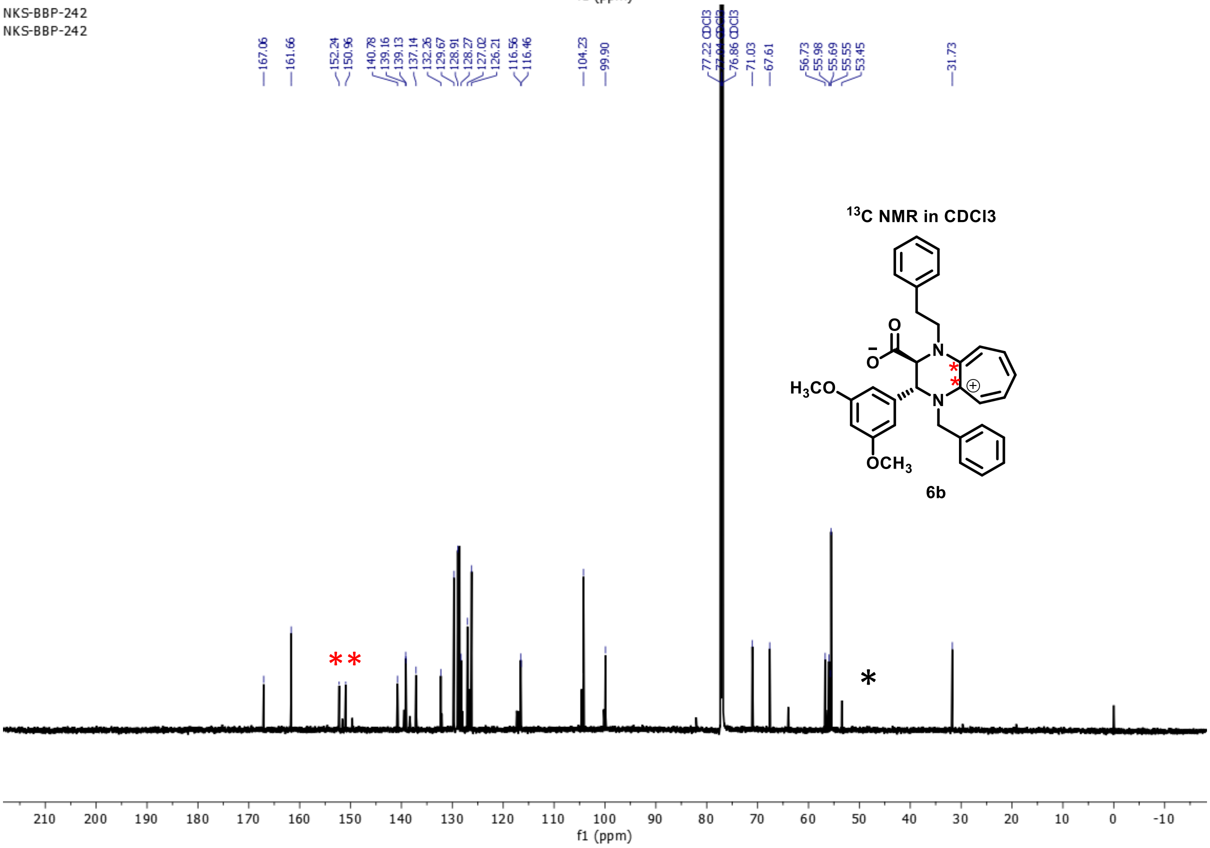

(*Residual solvents and impurity grease)

Fig S3. ^1H-/^{13}C-NMR spectra of **(6b)** in $CDCl_3$

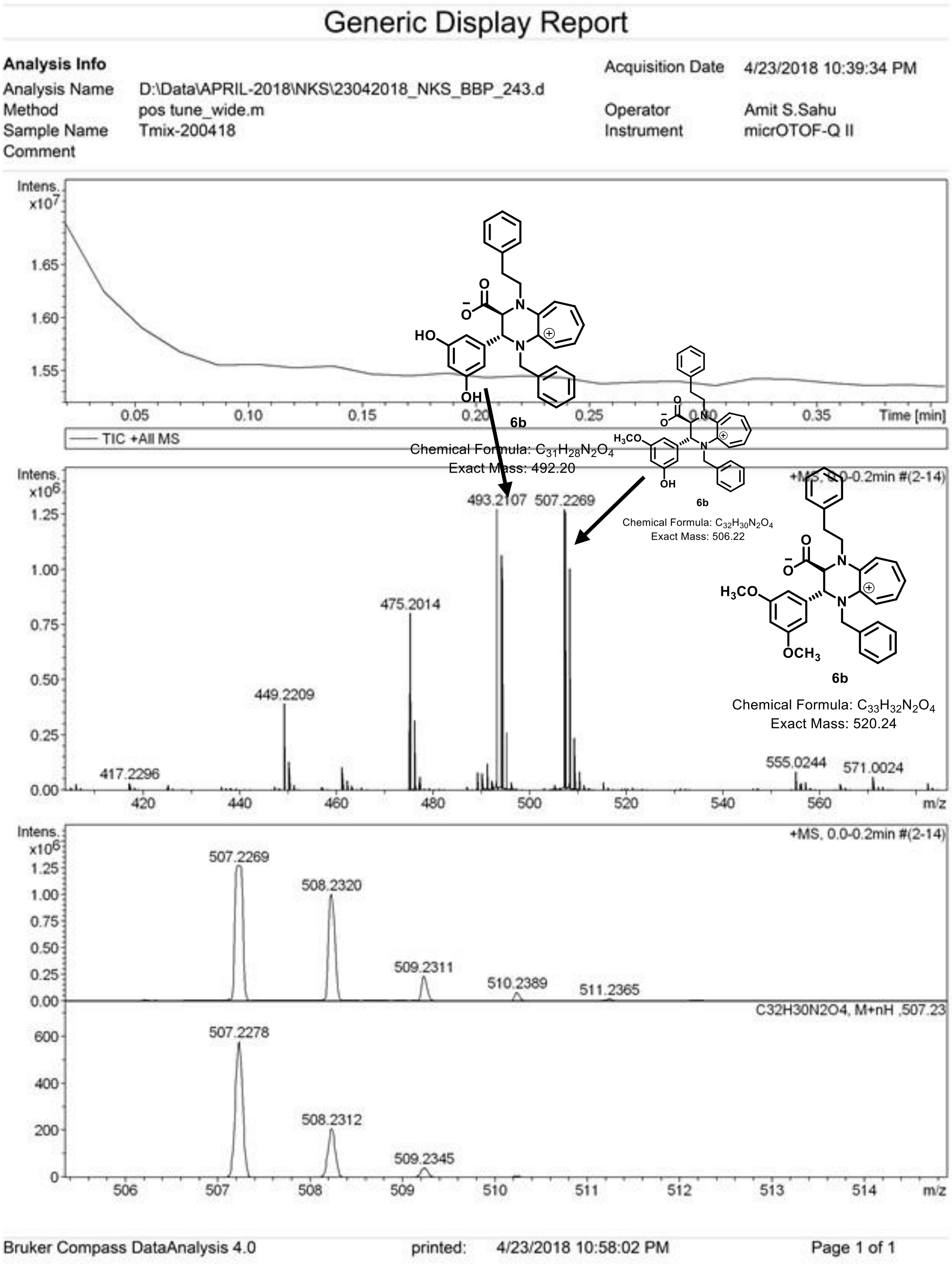

Fig S4. ESI/HRMS spectra of compound **(6b)**

3. NMR($^{1}H/^{13}C$) and HRMS of Cyclic-Aminotropiminium Carboxylate **(6c)**

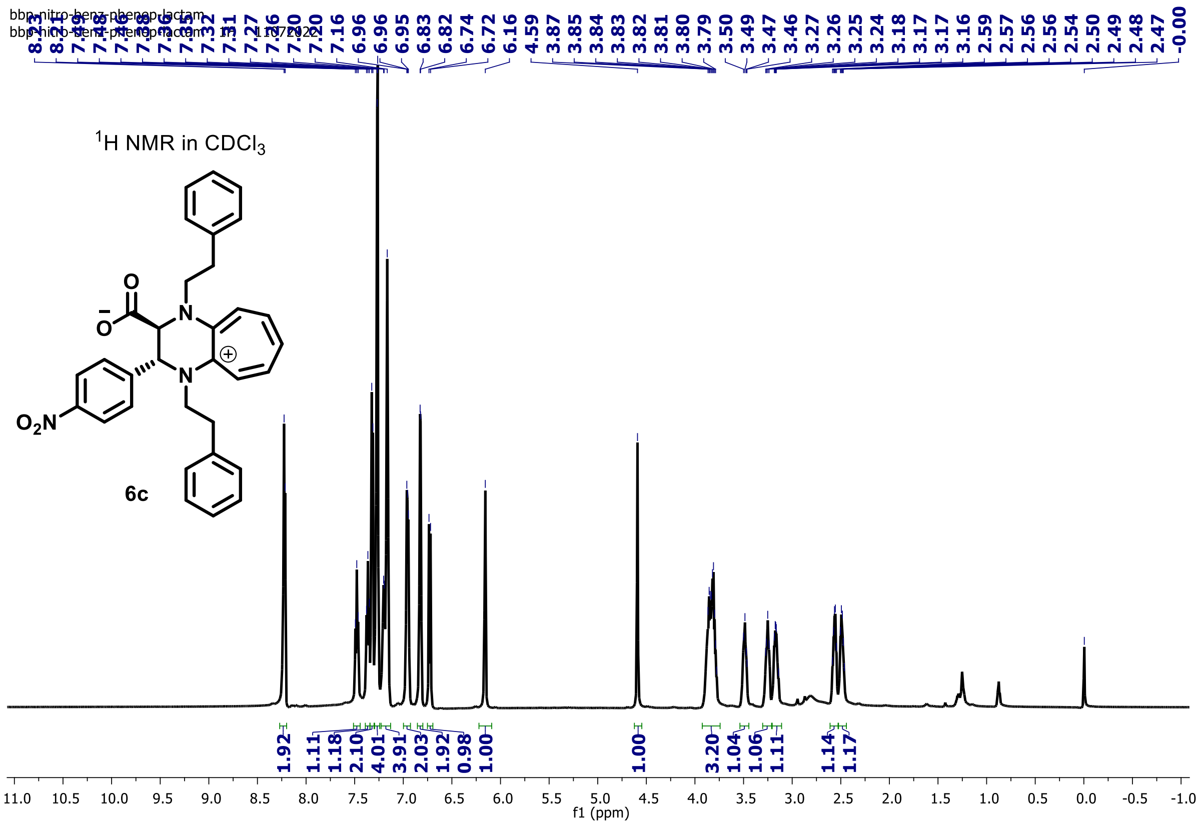

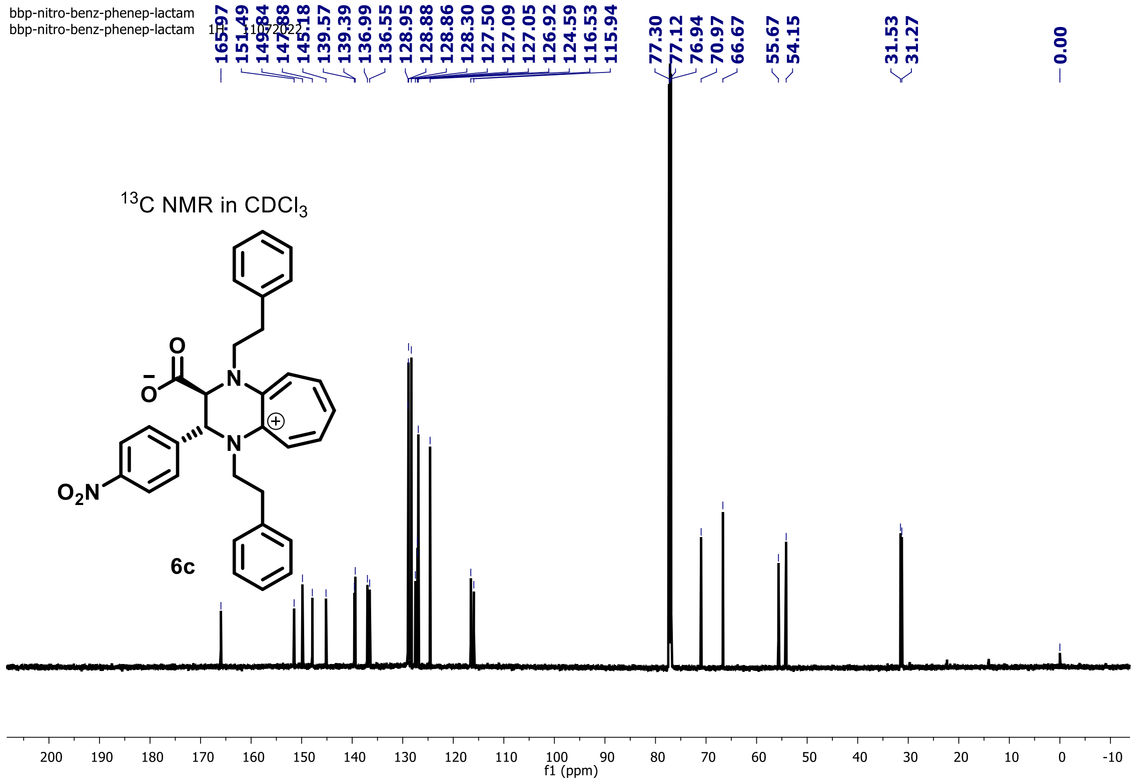

Fig S5. ^{1}H-/^{13}C-NMR spectra of **(6c)** in $CDCl_3$

Fig S6.

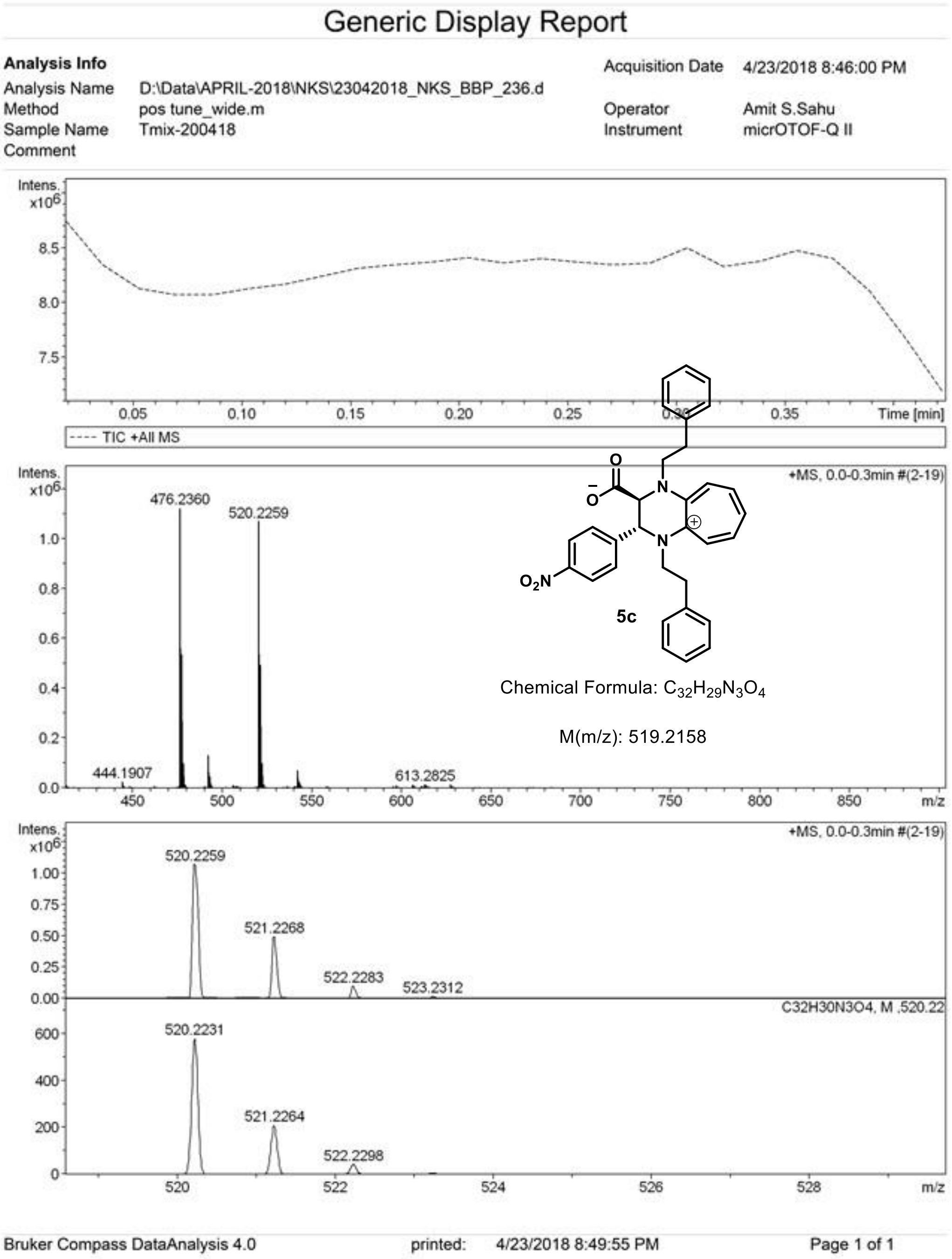

ESI/HRMS spectra of compound **(6c)**

4. NMR(^{1}H/^{13}C) and HRMS of Cyclic-Aminotropiminium Carboxylate (**6d**)

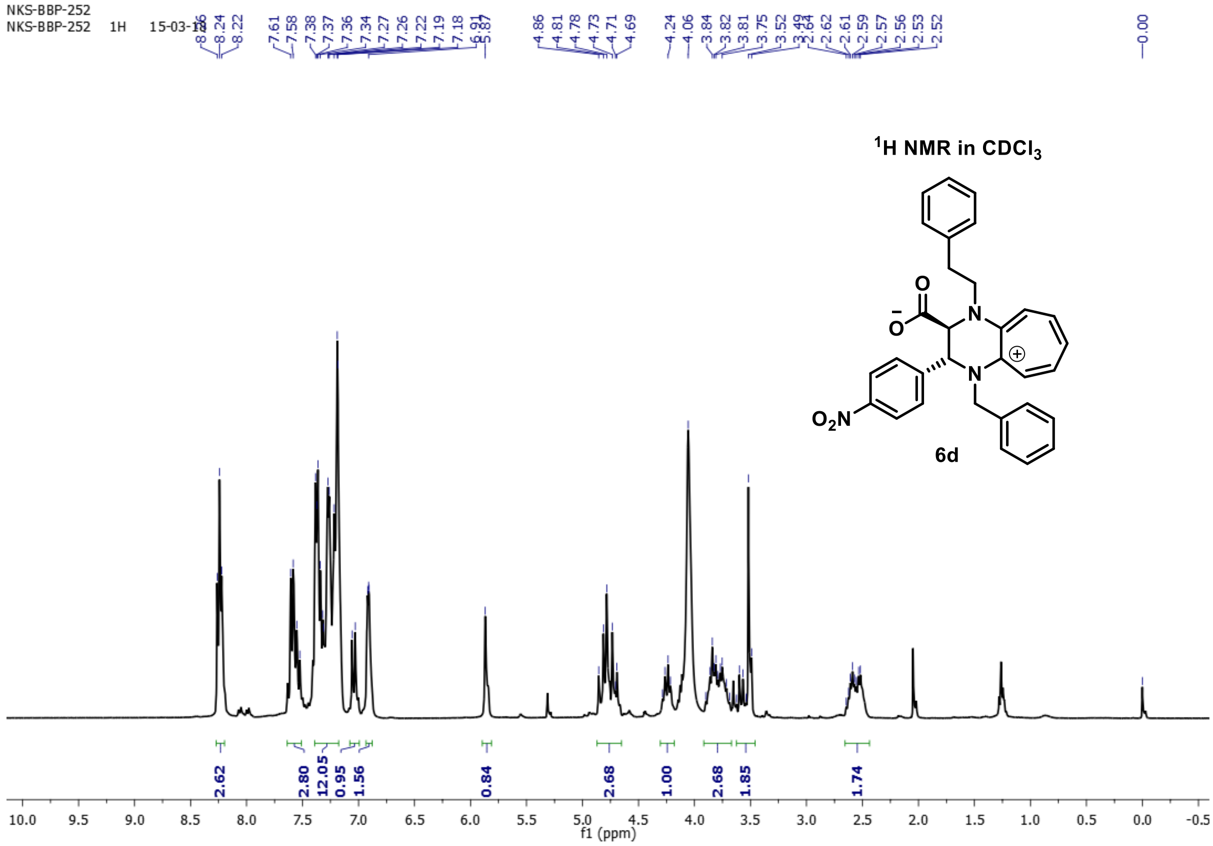

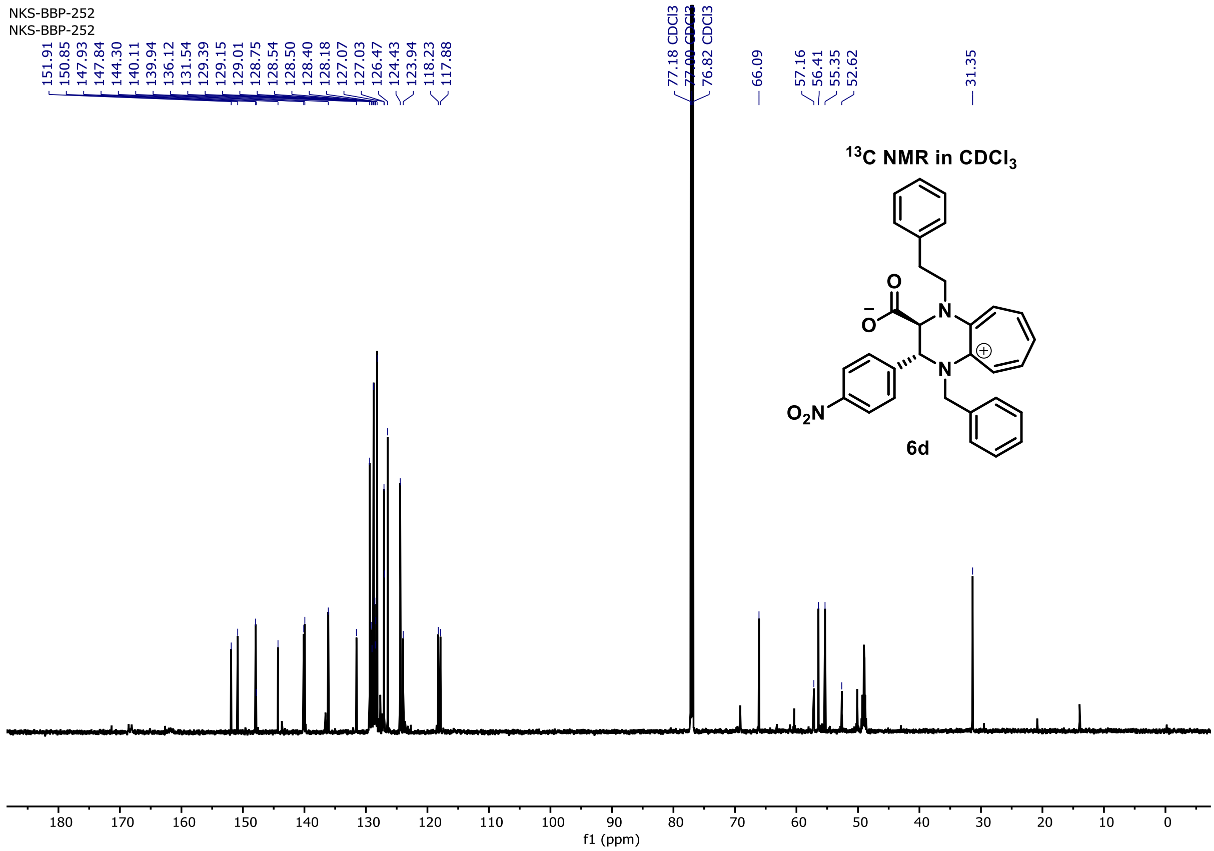

Fig S7. ^{1}H-/^{13}C-NMR spectra of **(6d)** in $CDCl_3$

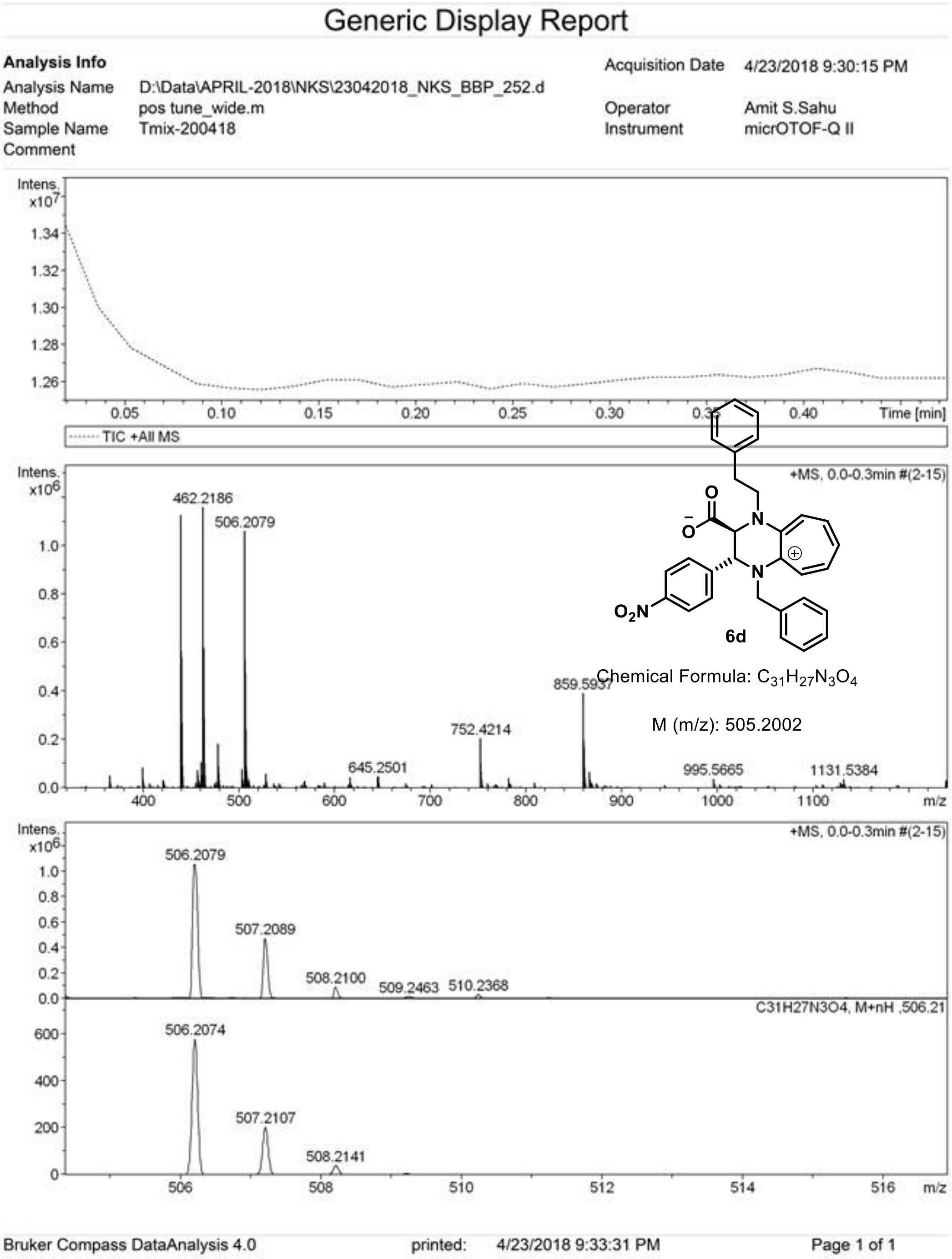

Fig S8. ESI/HRMS spectra of compound **(6d)**

5. NMR(^{1}H/^{13}C/^{13}CDEPT -135) and HRMS of Cyclic-Aminotropiminium Carboxylate (**6e**)

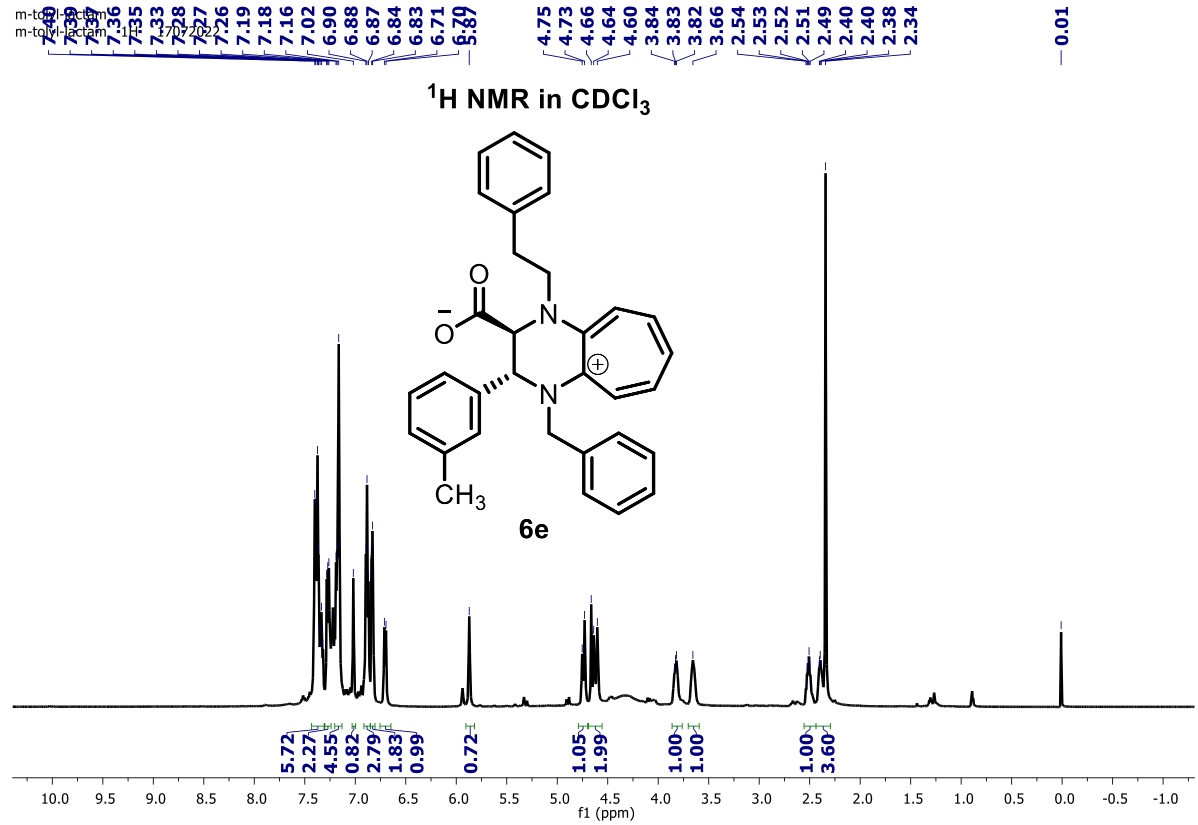

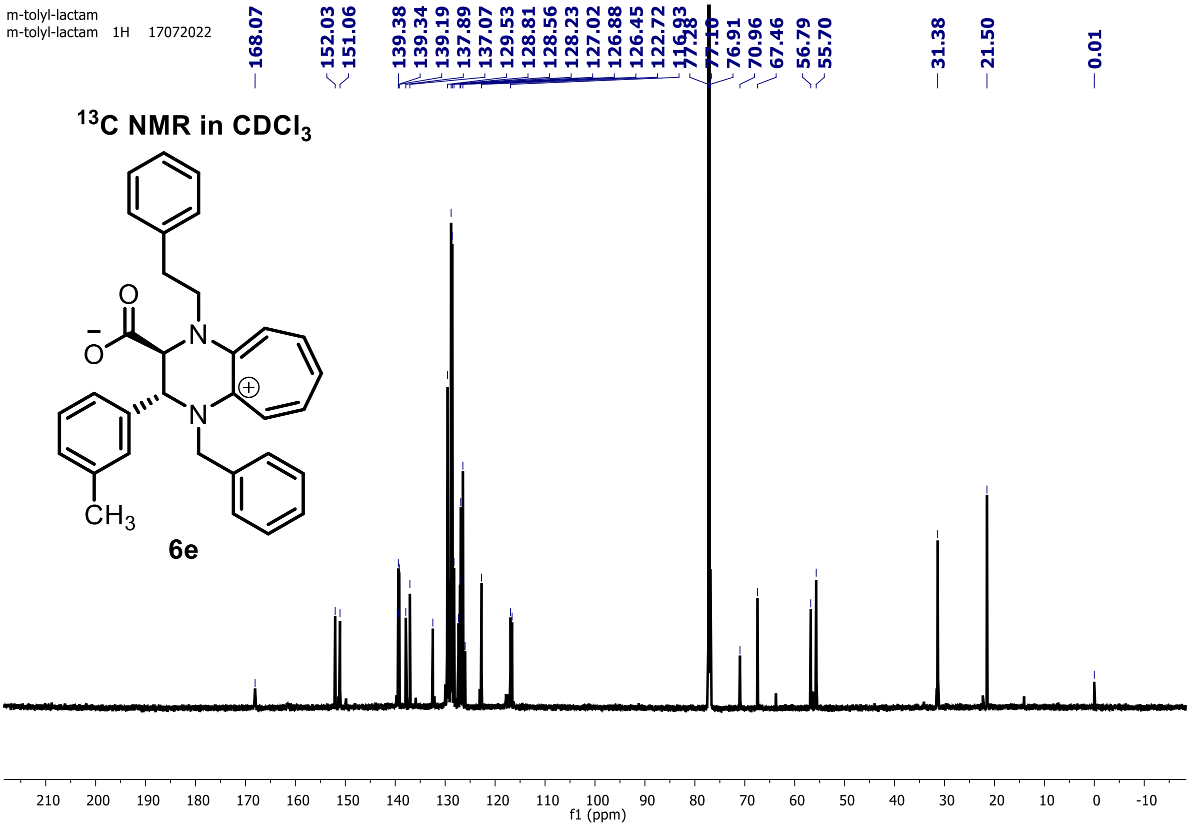

Fig S9. ^{1}H-/^{13}C-NMR spectra of **(6e)** in $CDCl_3$

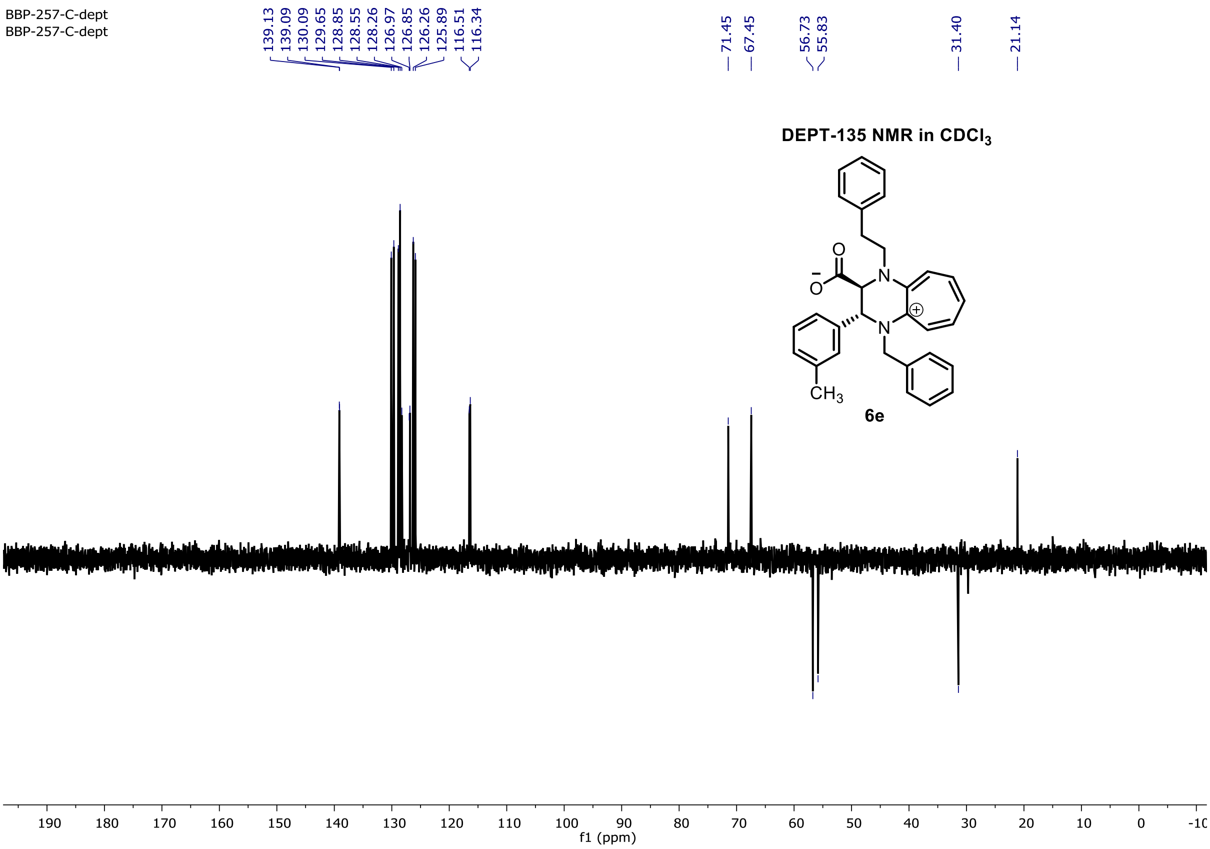

Fig S10. ^{13}CDEPT-135 NMR spectra of **(6e)** in $CDCl_3$

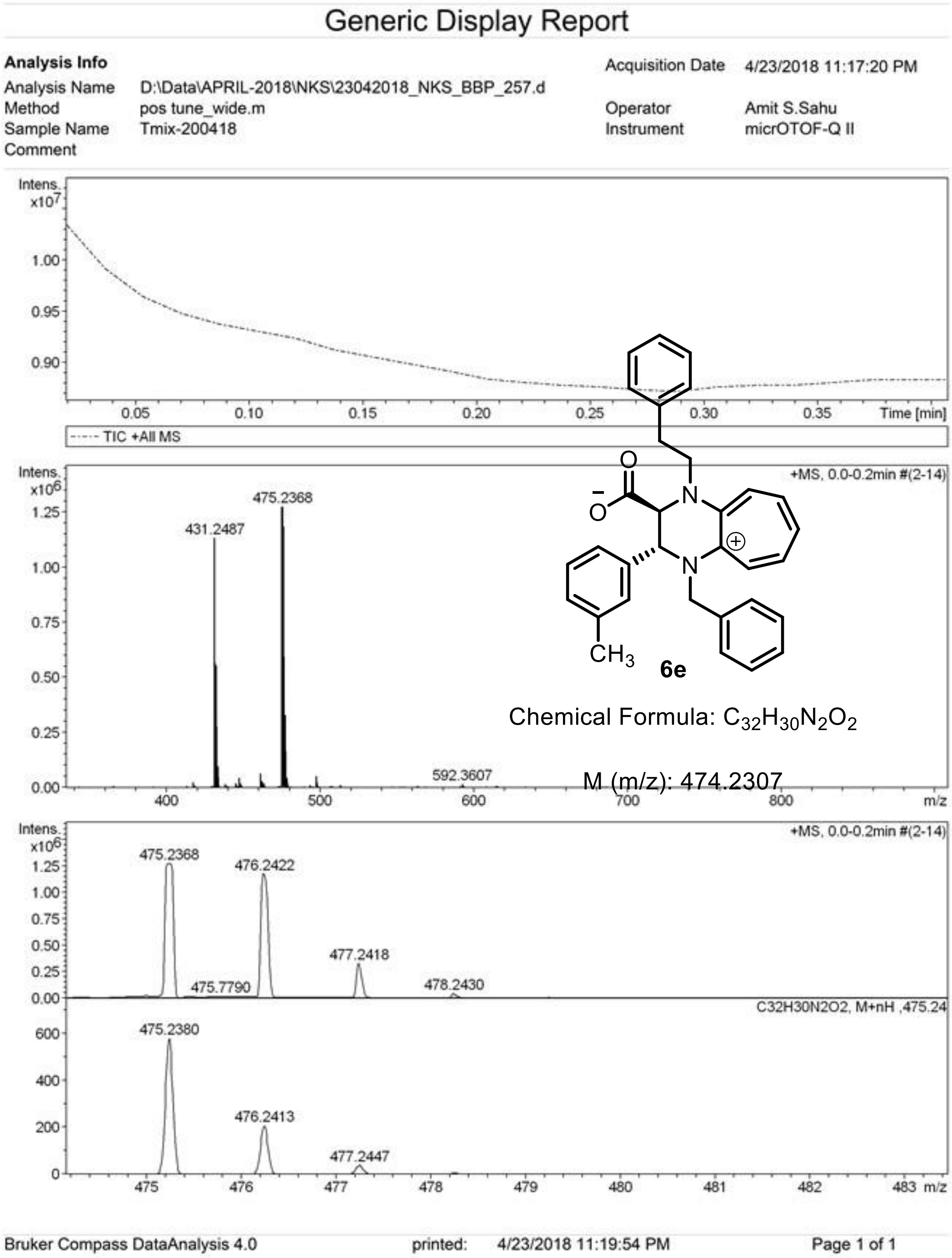

Fig S11. ESI/HRMS spectra of compound **(6e)**

6. NMR(1H/13C) and HRMS of Cyclic-Aminotropiminium Carboxylate **(6f)**

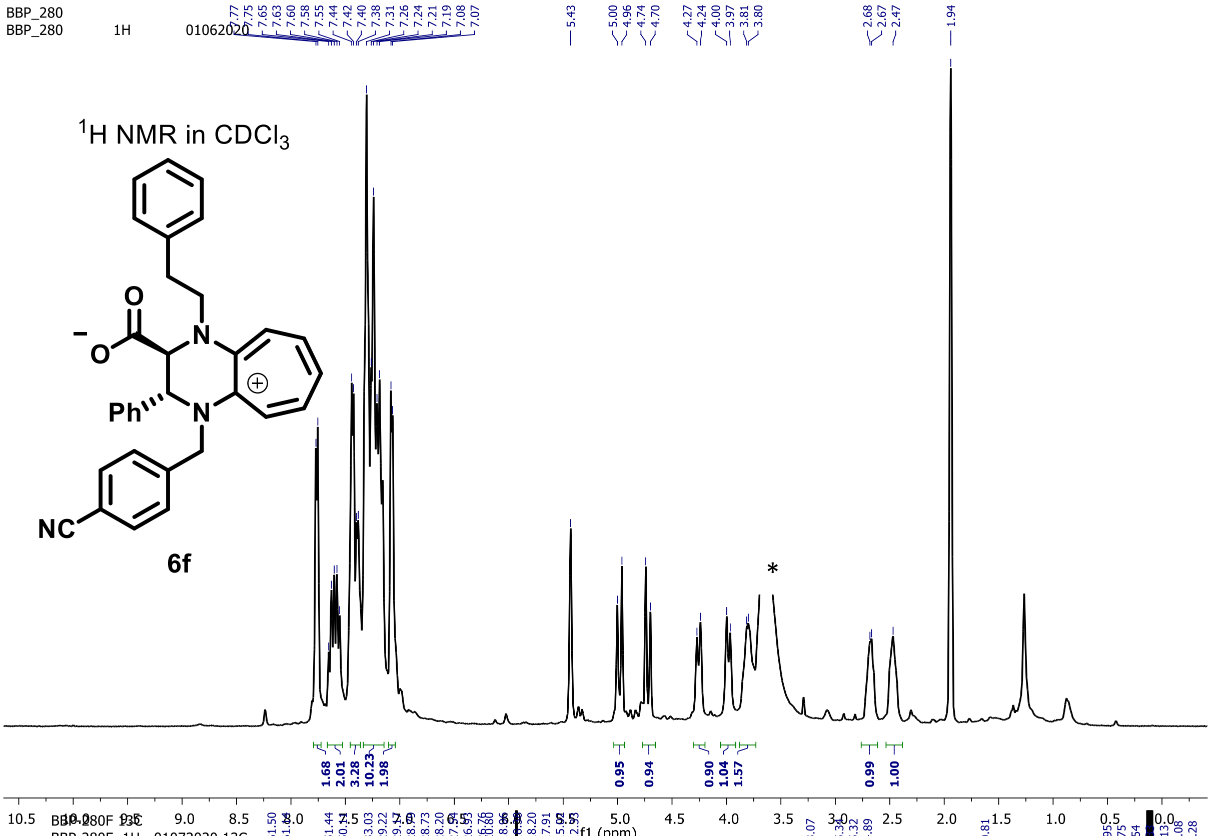

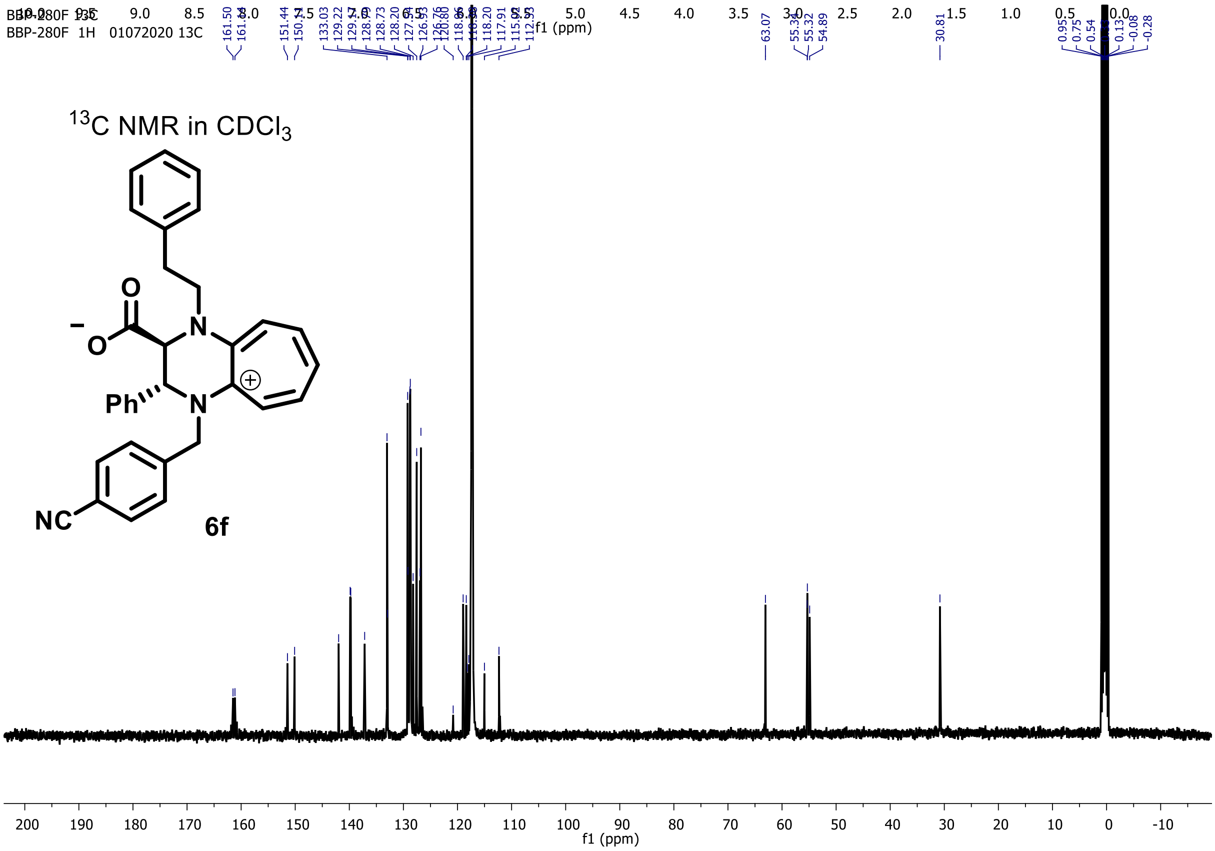

Fig S12. ^{1}H-/^{13}C- NMR spectra of **(6f)** in CDCl$_3$

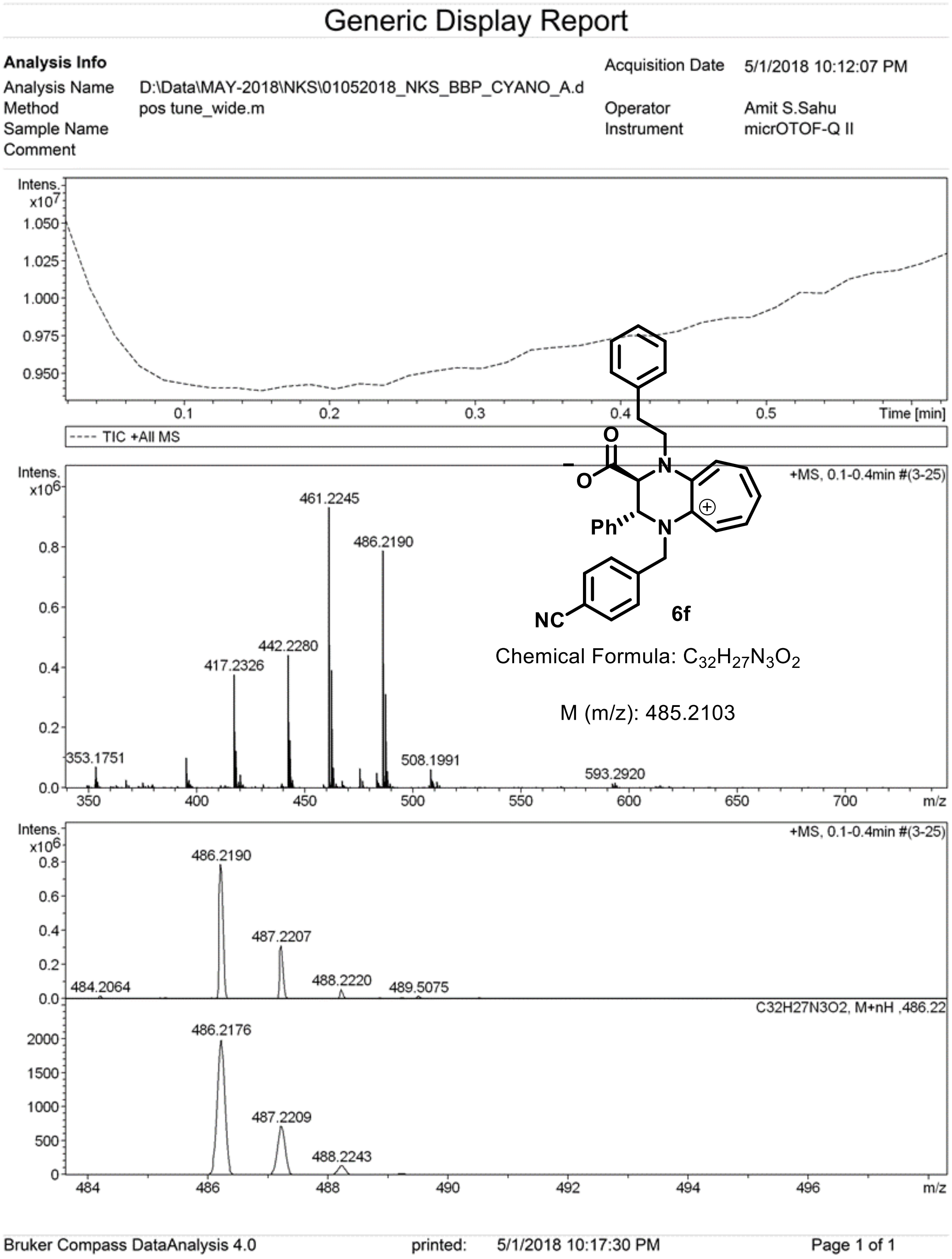

Fig S13. ESI/HRMS spectra of compound **(6f)**

7. NMR(^{1}H/^{13}C) and HRMS of Cyclic-Aminotropiminium Carboxylate **(6g)**

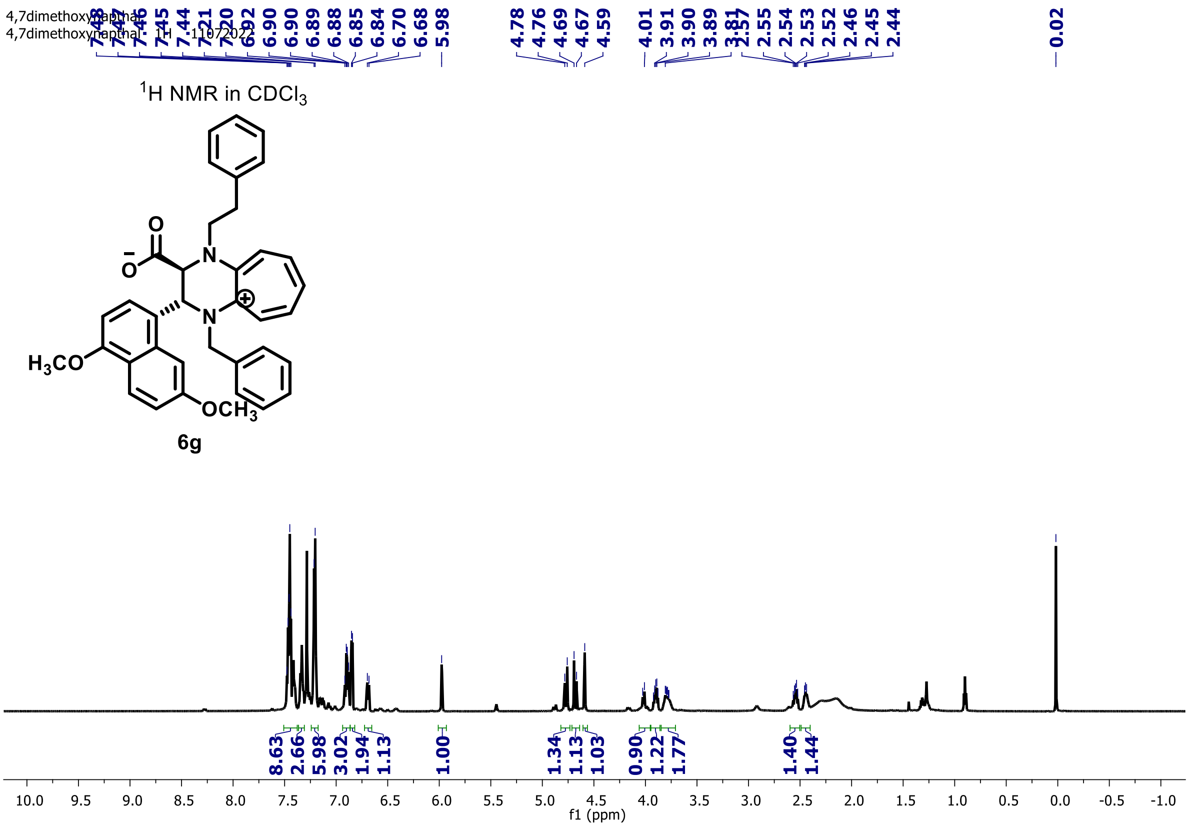

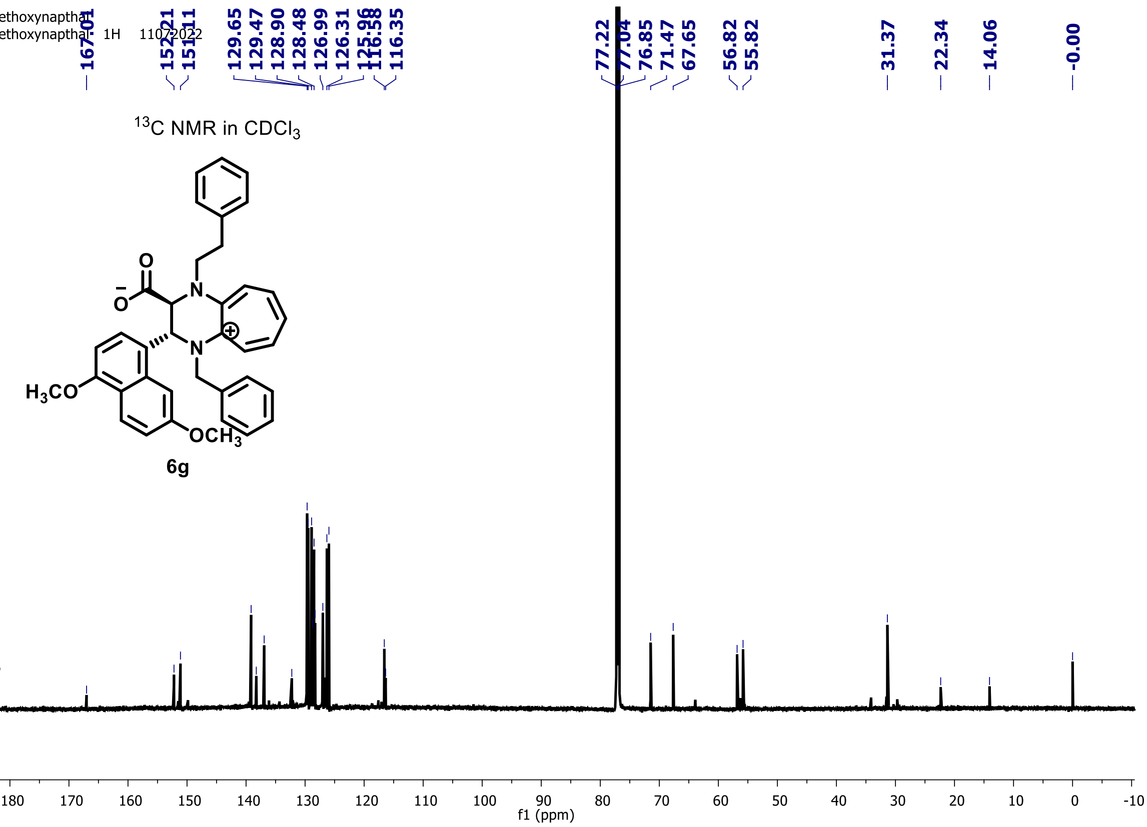

Fig **S14.** ^{1}H-/^{13}C-NMR spectra of **(6g)** in $CDCl_3$

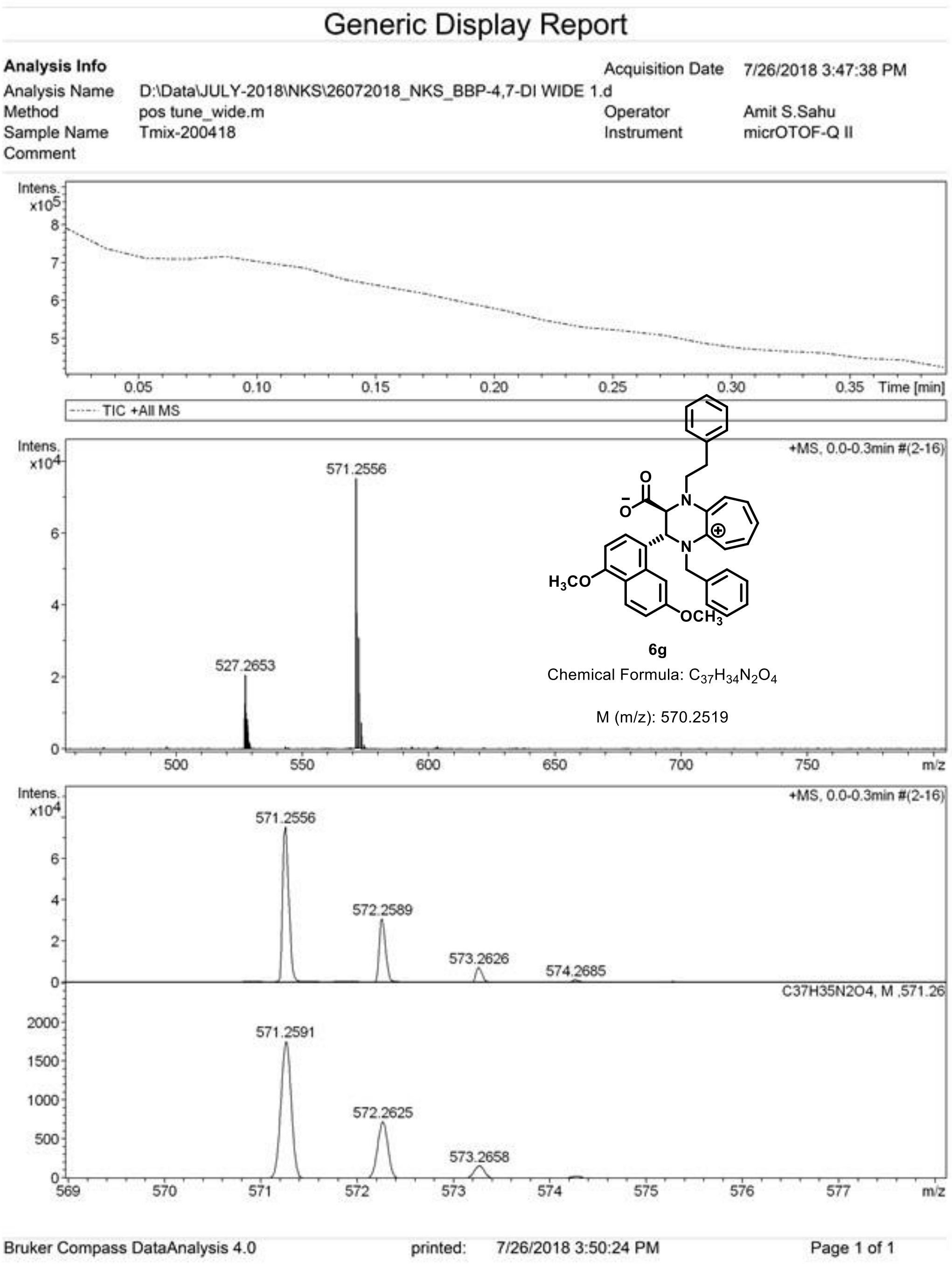

Fig S15. ESI/HRMS spectra of compound **(6g)**

8. NMR(^{1}H/^{13}C/^{13}CDEPT) and HRMS of Cyclic-Aminotropiminium Carboxylate **(6h)**

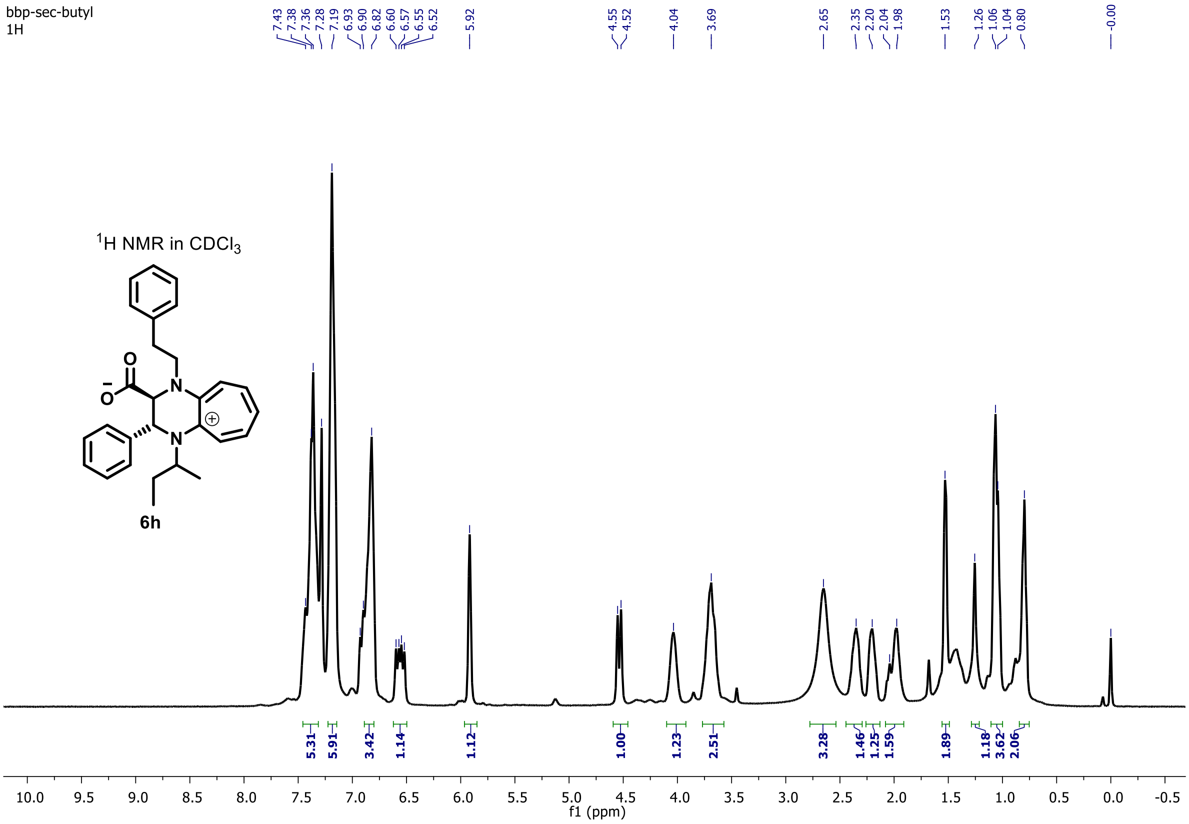

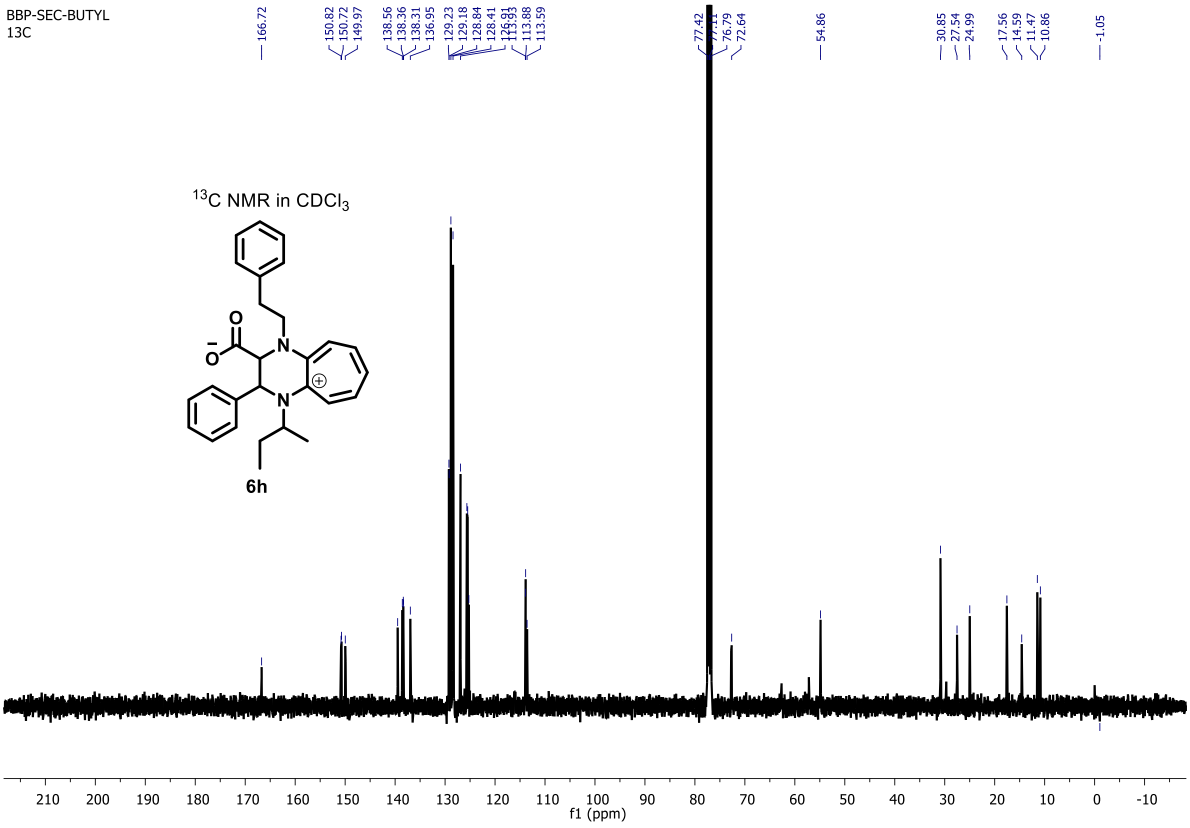

Fig S16. ^{1}H-/^{13}C-NMR spectra of **(6h)** in $CDCl_3$

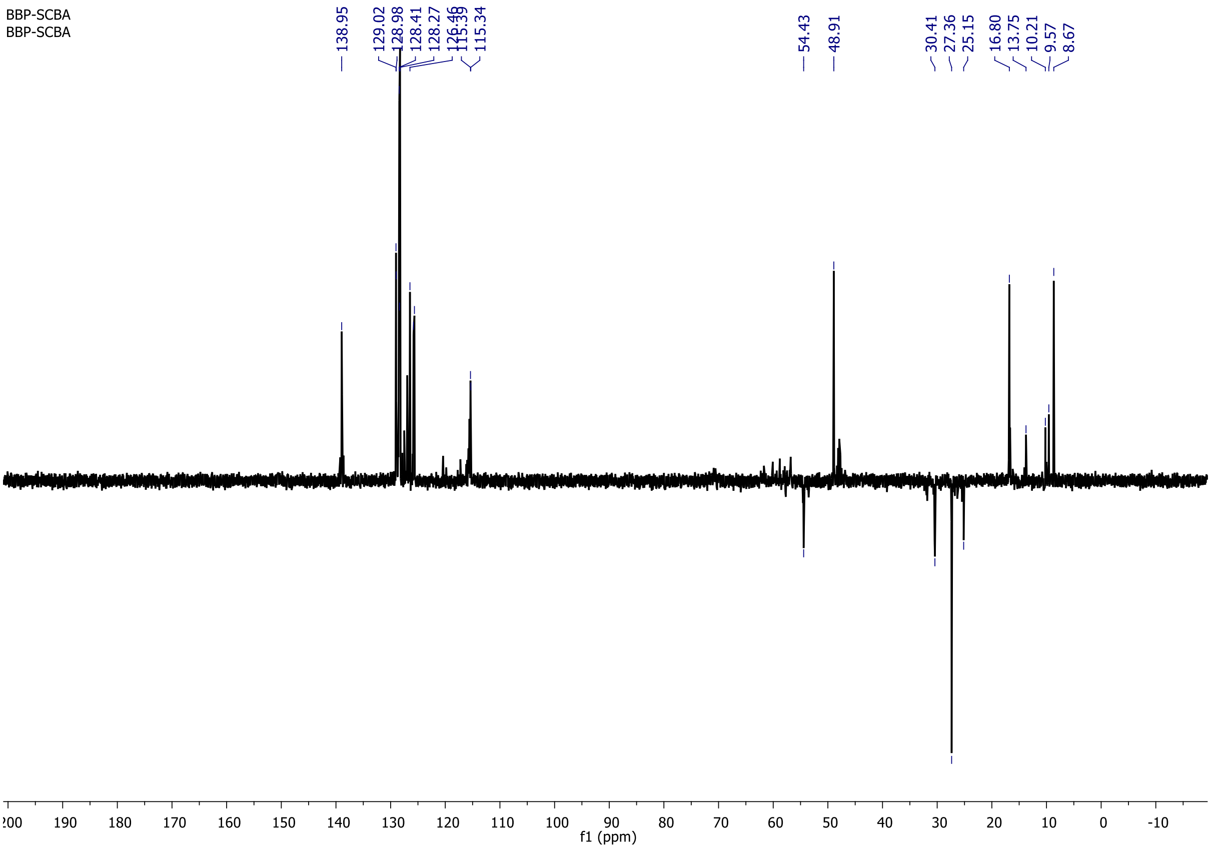

Fig S17. ^{13}CDEPT -135 NMR spectra of **(6h)** in $CDCl_3$

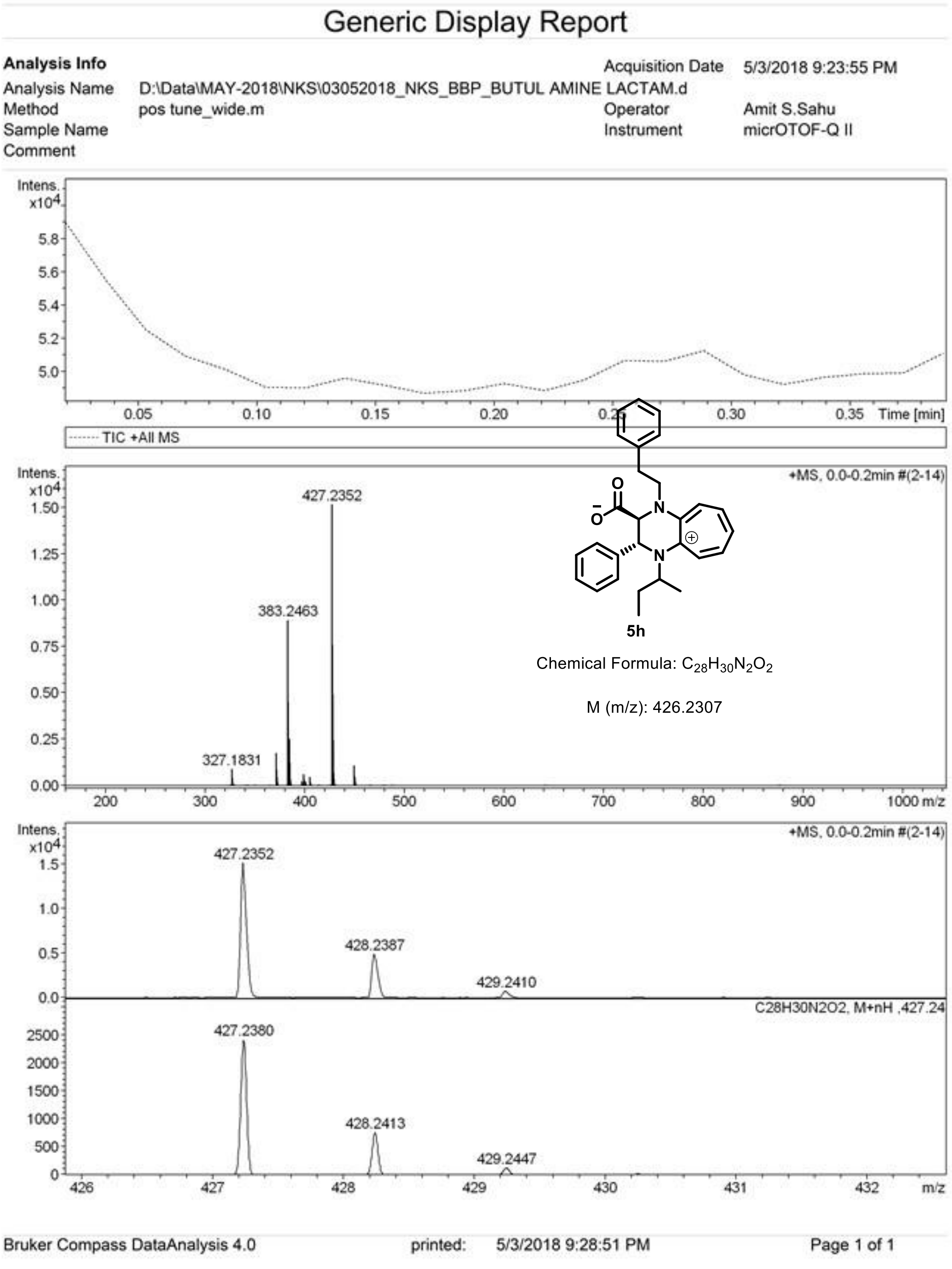

Fig S18. ESI/HRMS spectra of compound **(6h)**

9. NMR($^1H/^{13}C$) and HRMS of Cyclic-Aminotropiminium Carboxylate **(6i)**

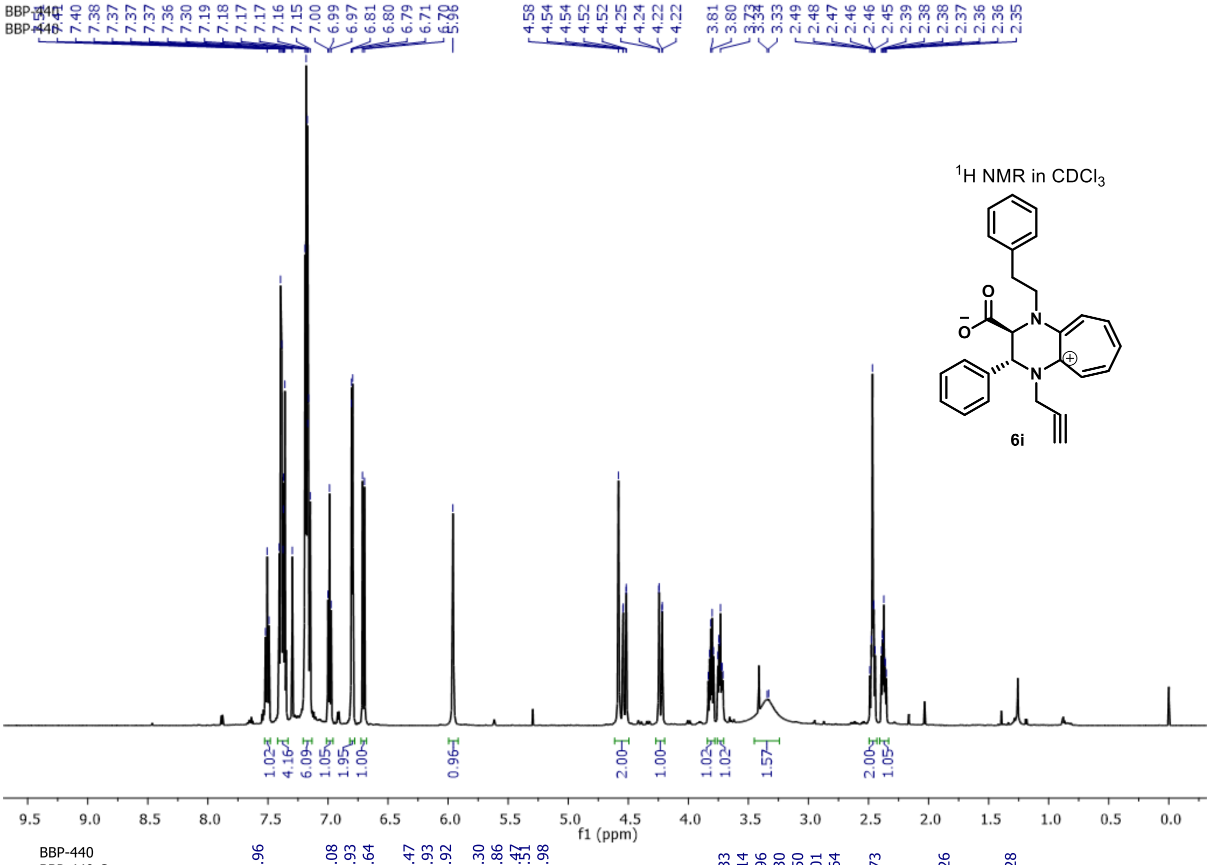

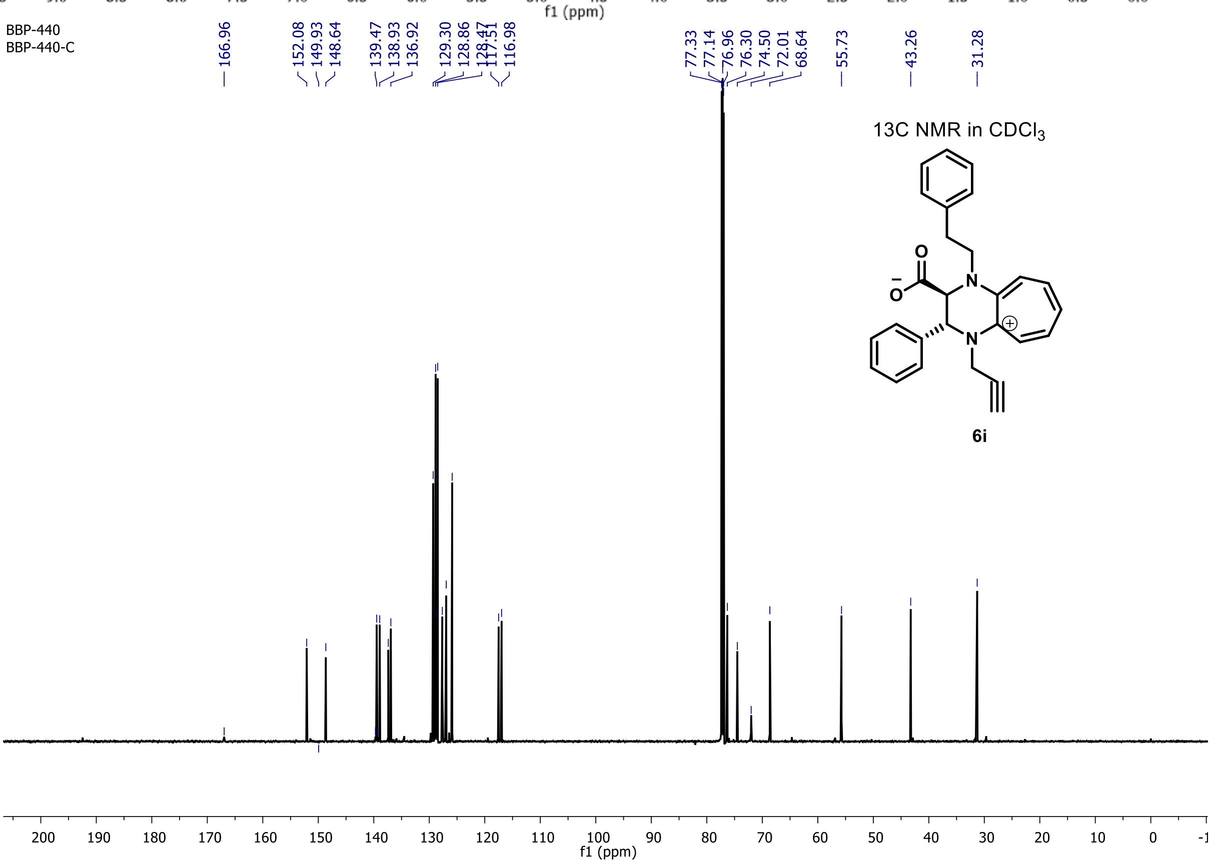

Fig S19. ^{1}H-/^{13}C NMR spectra of **(6i)** in $CDCl_3$

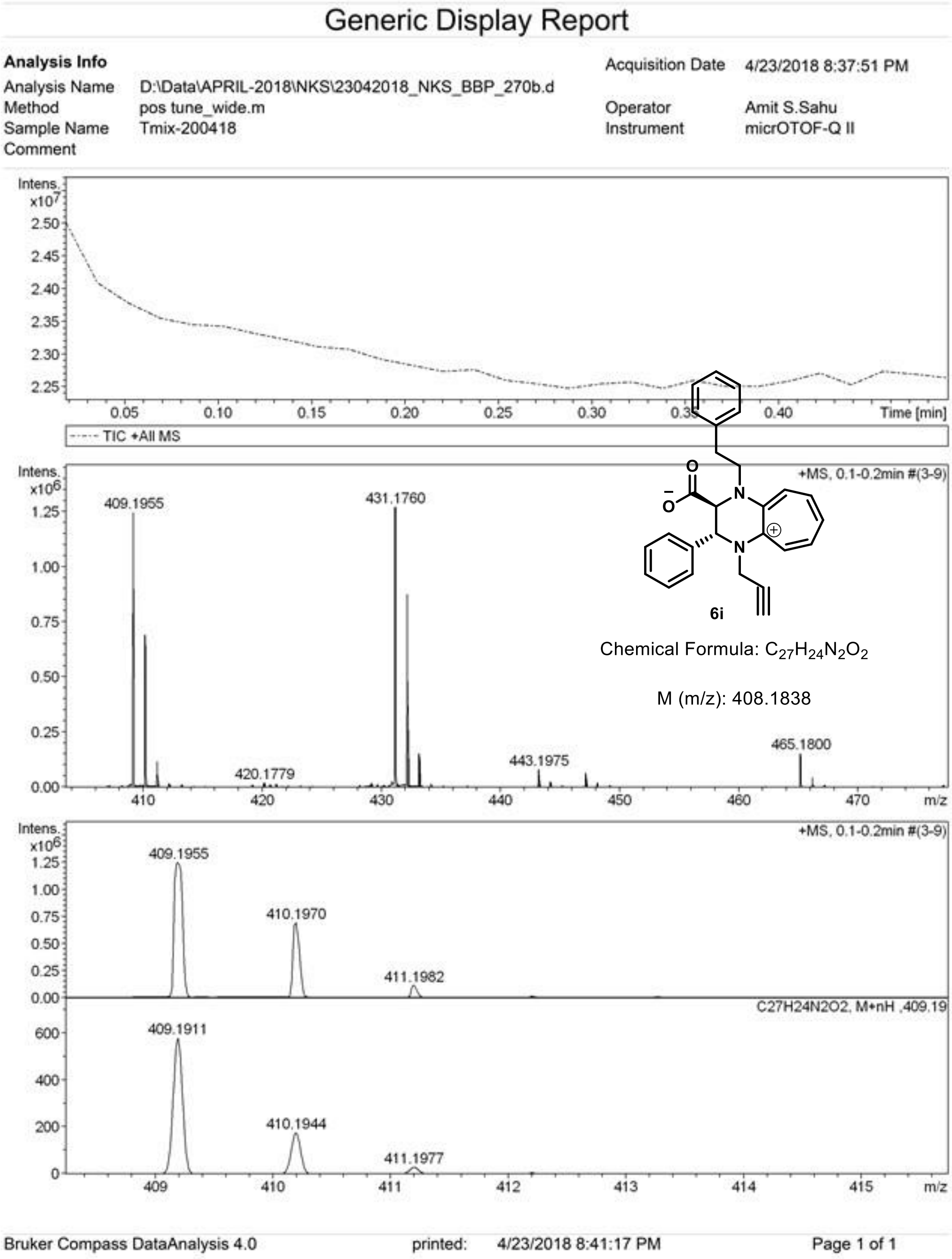

Fig S20. ESI/HRMS spectra of compound **(6i)**

10. NMR(^{1}H/^{13}C) and HRMS of Cyclic-Aminotropiminium Carboxylate **(6j)**

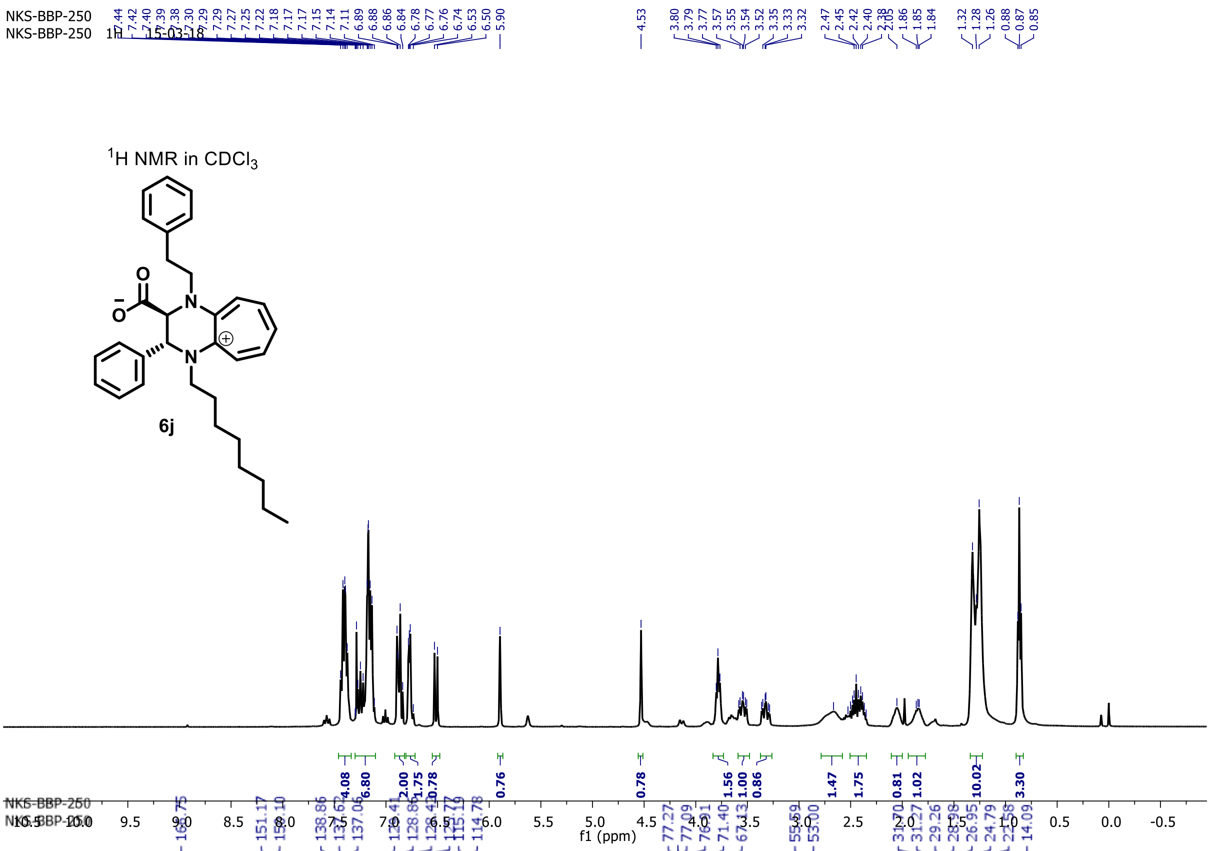

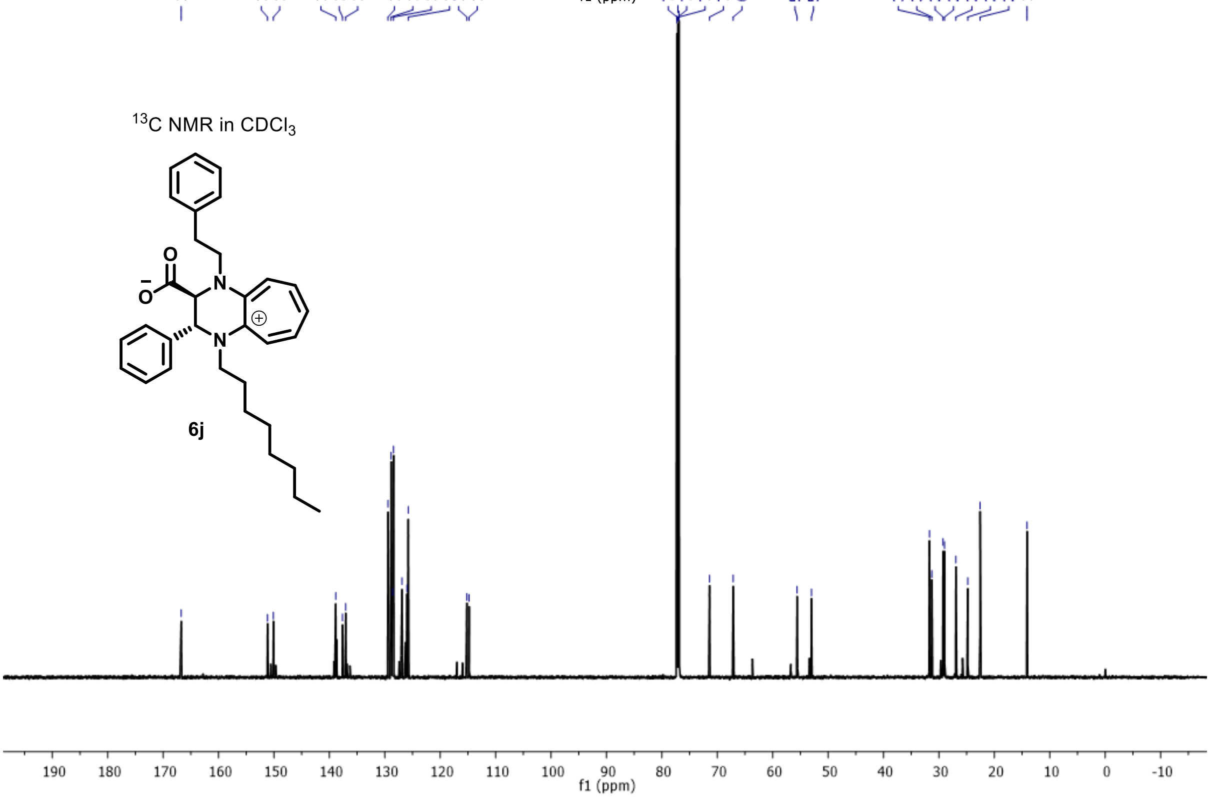

Fig S21. ^{1}H-/^{13}C- NMR spectra of **(6j)** in $CDCl_3$

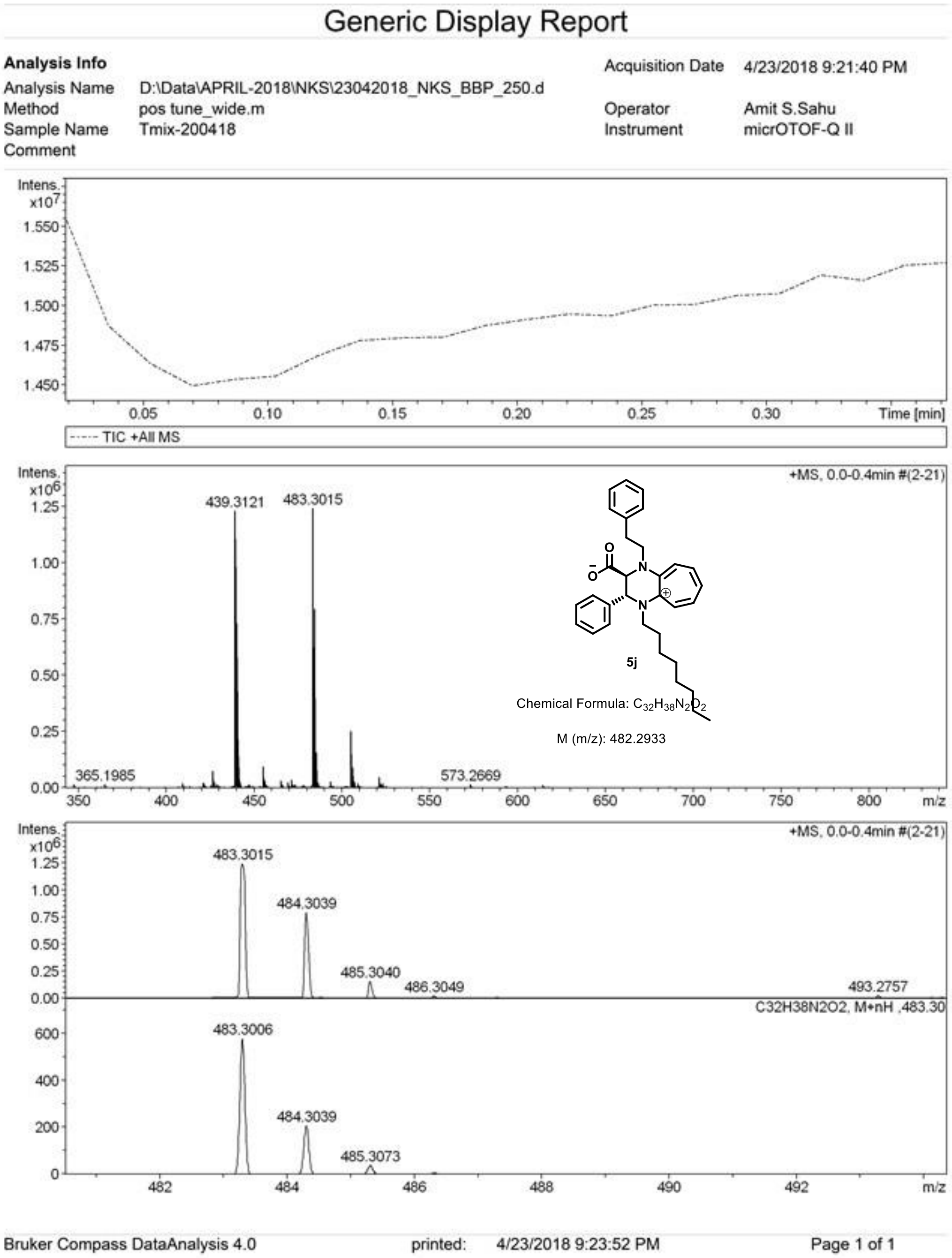

Fig S22. ESI/HRMS spectra of compound **(6j)**

11. NMR($^{1}H/^{13}C/^{13}$CDEPT) and HRMS of Cyclic-Aminotropiminium Carboxylate **(6k)**

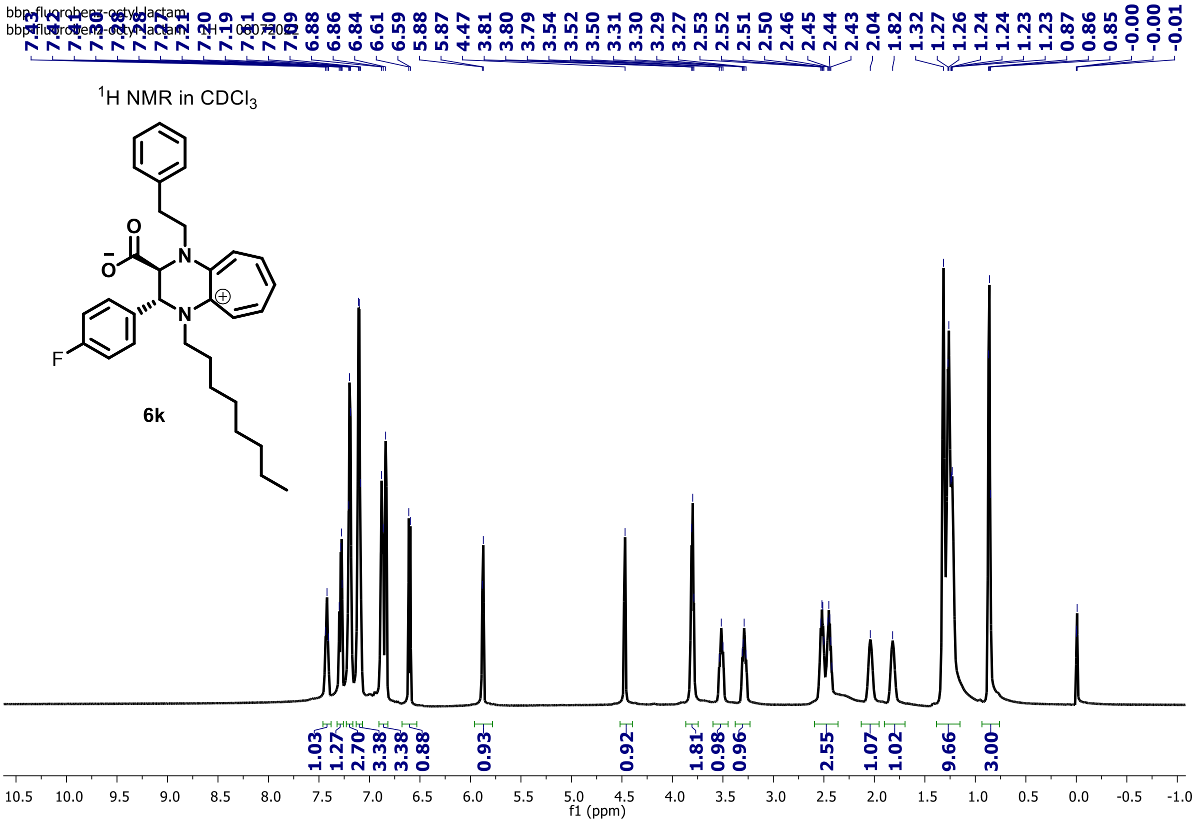

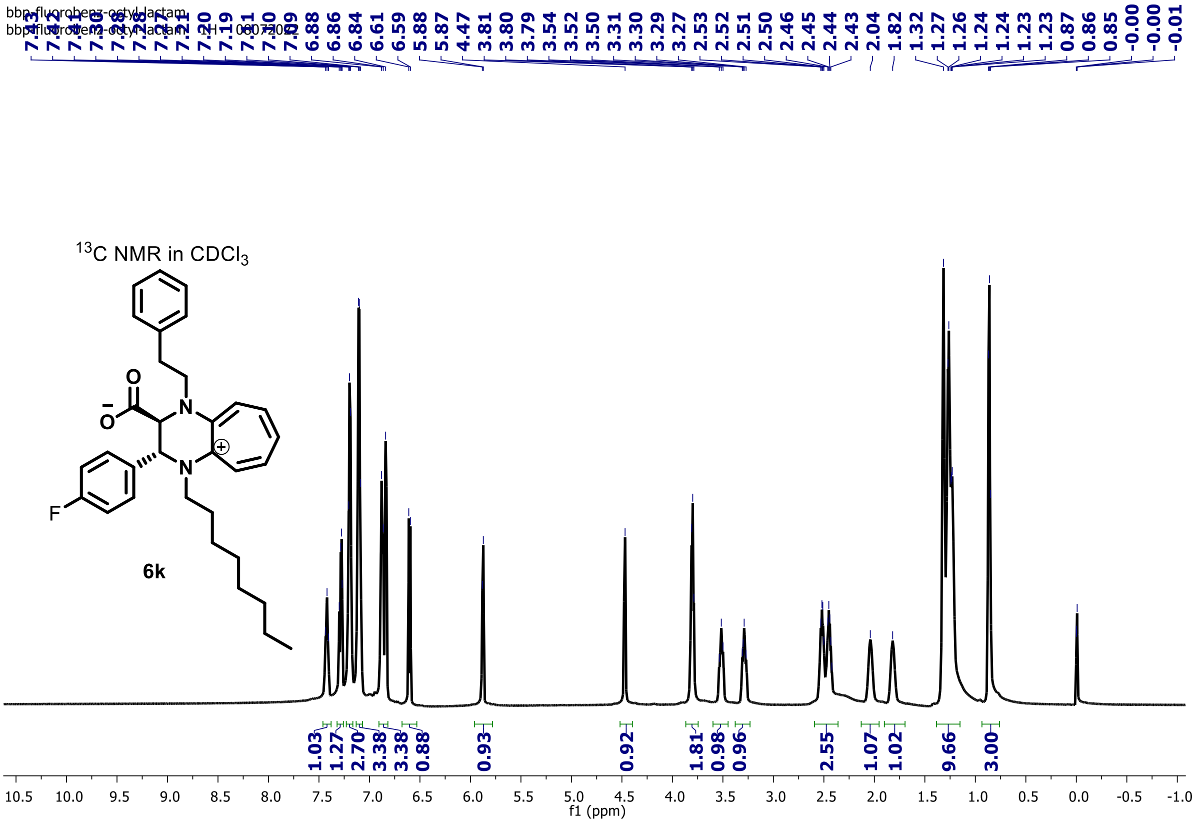

Fig S23. ^{1}H-/^{13}C-NMR spectra of **(6k)** in $CDCl_3$

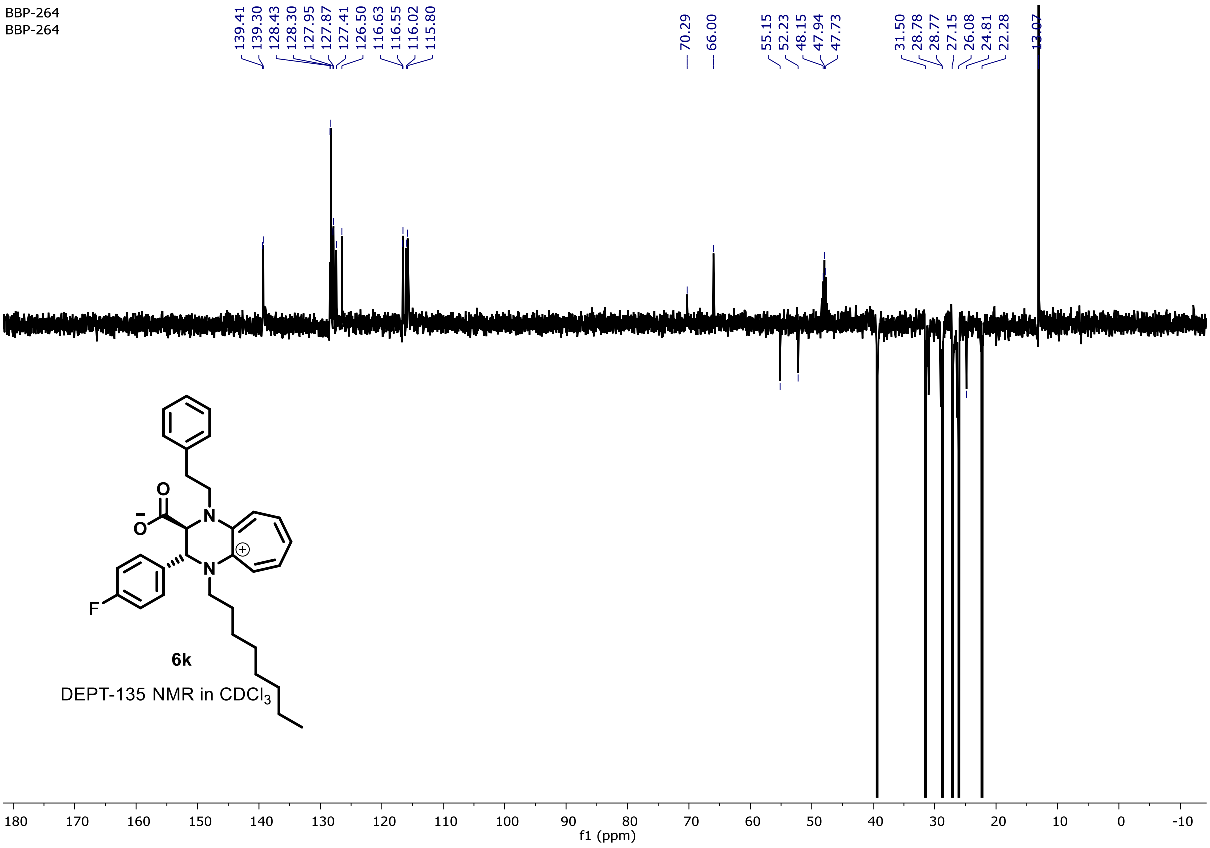

Fig S24. [13]CDEPT NMR spectra of **(6k)** in $CDCl_3$

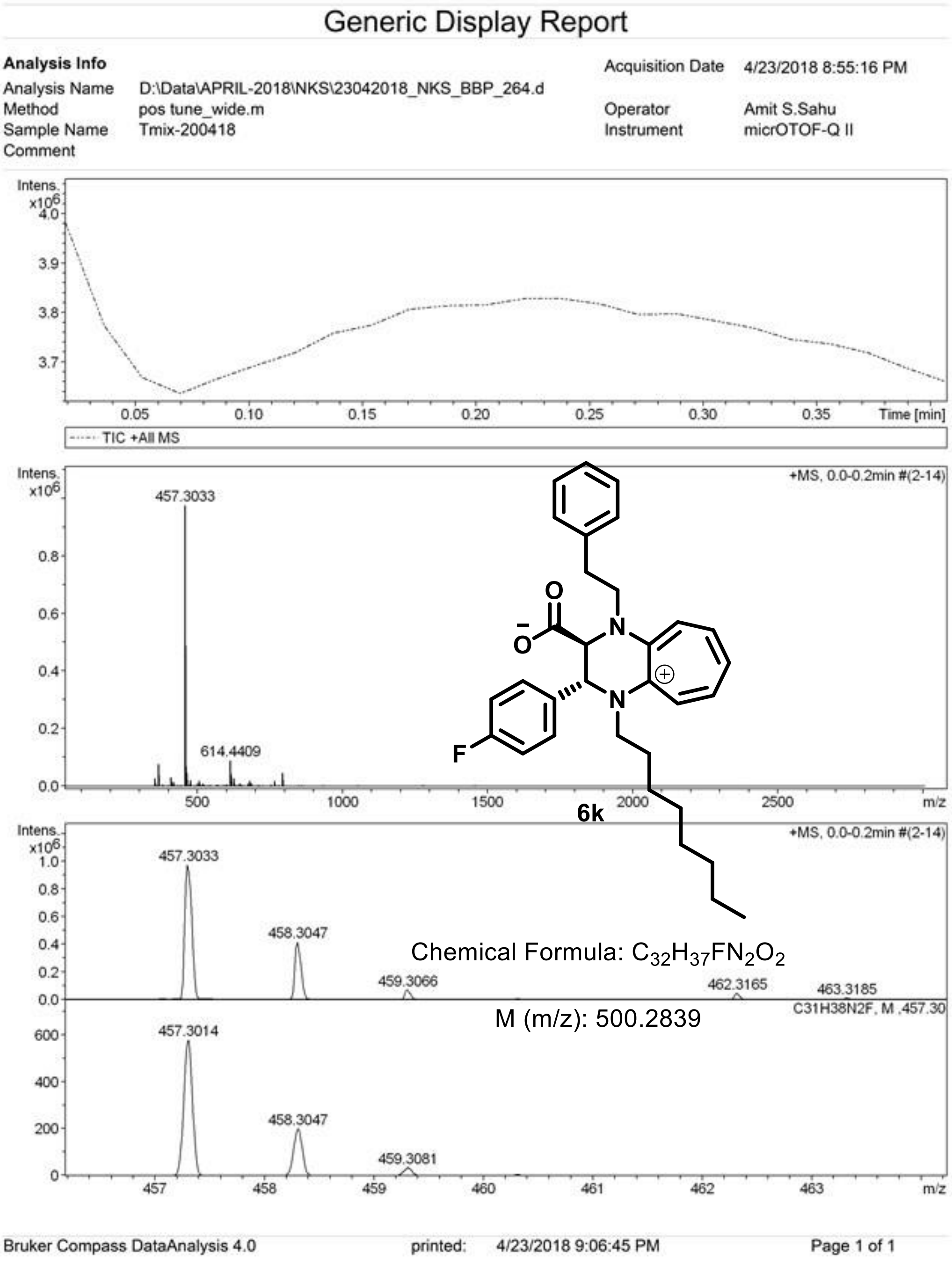

Fig S25. ESI/HRMS spectra of compound **(6k)**

12. NMR(1H/13C) and HRMS of Cyclic-Aminotropiminium Carboxylate **(6l)**

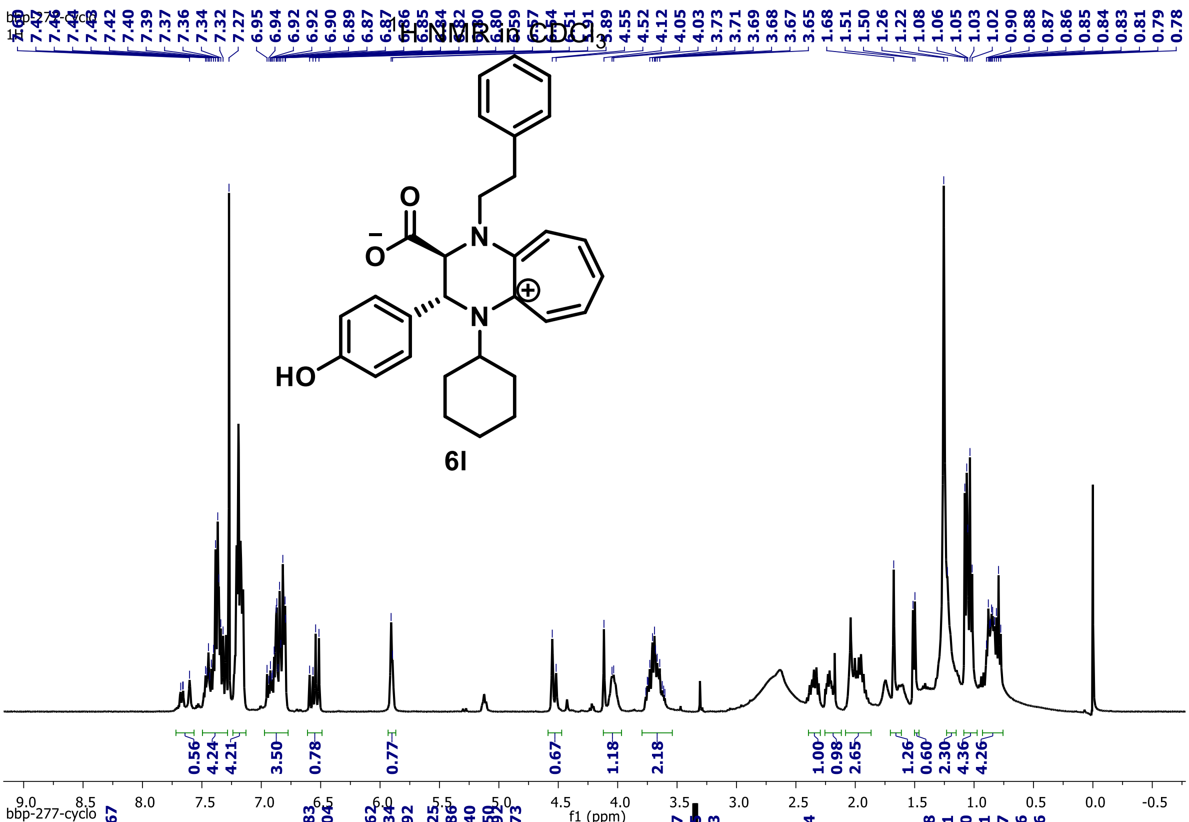

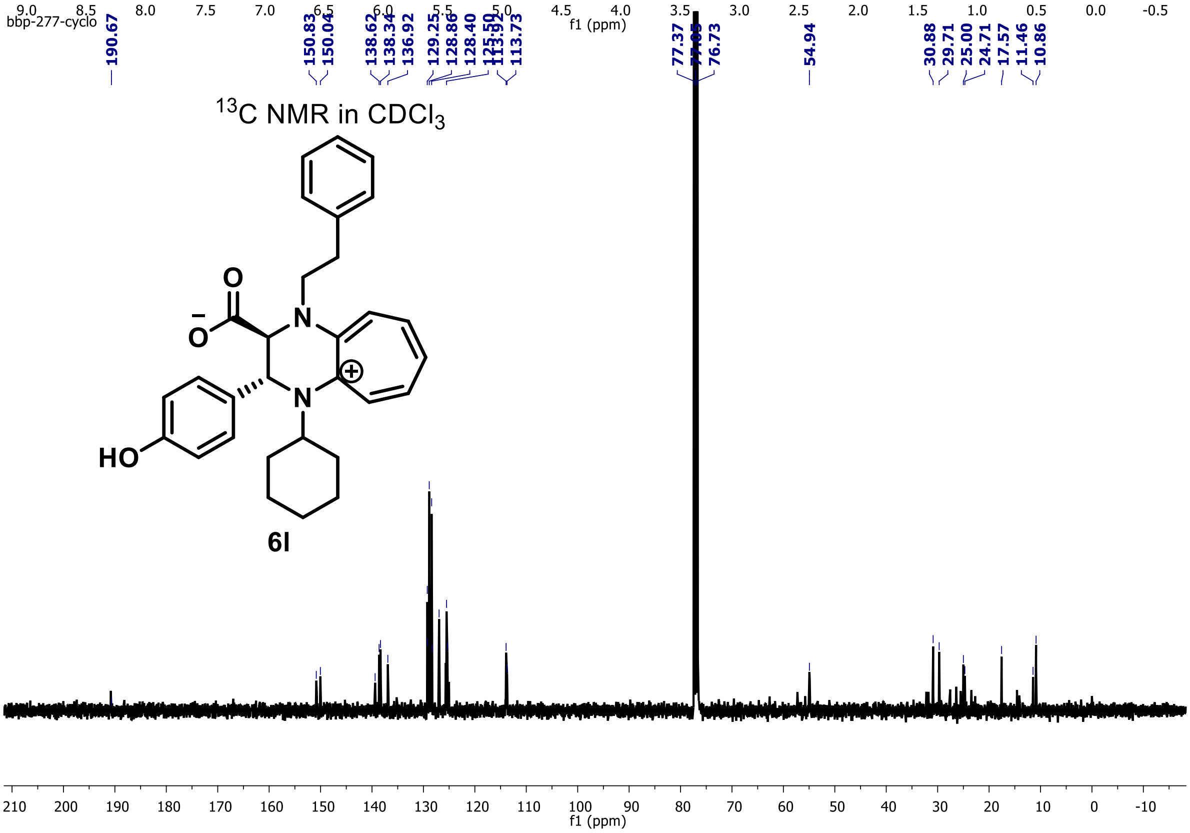

Fig S26. ^{1}H-/^{13}C NMR spectra of **(6m)** in $CDCl_3$

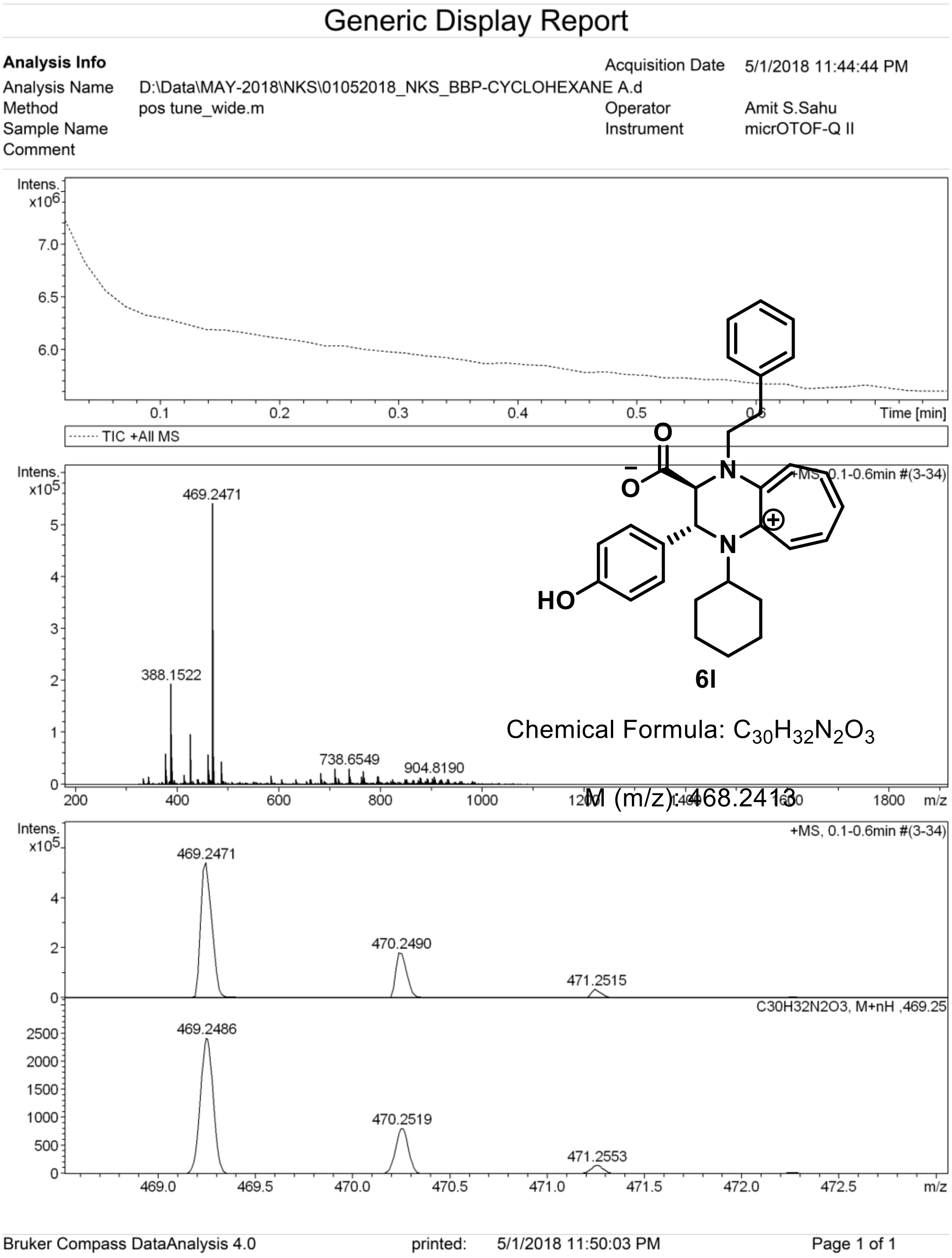

Fig S27. ESI/HRMS spectra of compound **(6l)**

13. NMR($^{1}H/^{13}C$) and HRMS of Cyclic-Aminotropiminium Carboxylate **(6m)**

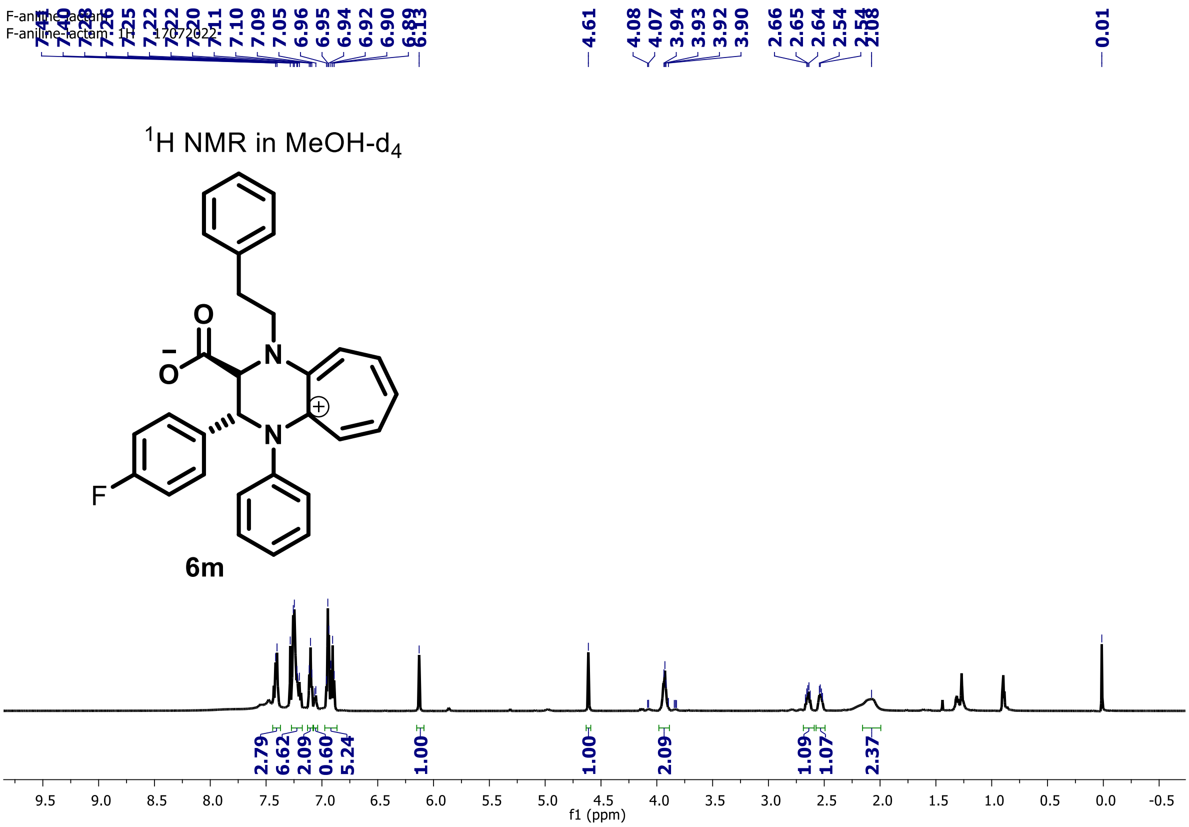

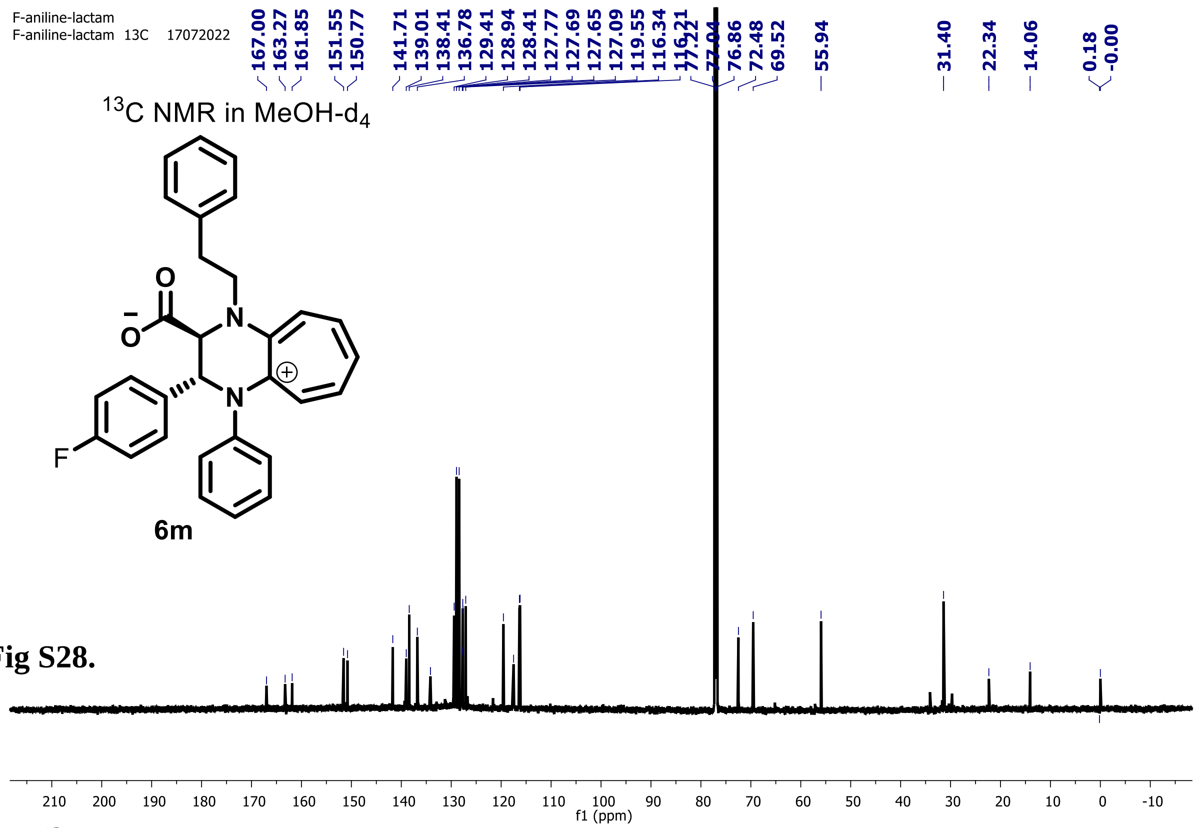

Fig S28.

^{1}H-/^{13}C-NMR spectra of **(6m)** in MeOH-d$_4$

Display Report

Analysis Info

Analysis Name	D:\Data\APRIL-2018\NKS\23042018_NKS_BBP_265.d	Acquisition Date	4/23/2018 9:11:23 PM
Method	pos tune_wide.m	Operator	Amit S.Sahu
Sample Name	Tmix-200418	Instrument	micrOTOF-Q II 10337
Comment			

Acquisition Parameter

Source Type	ESI	Ion Polarity	Positive	Set Nebulizer	0.4 Bar
Focus	Not active	Set Capillary	4500 V	Set Dry Heater	180 °C
Scan Begin	50 m/z	Set End Plate Offset	-500 V	Set Dry Gas	4.0 l/min
Scan End	3000 m/z	Set Collision Cell RF	650.0 Vpp	Set Divert Valve	Waste

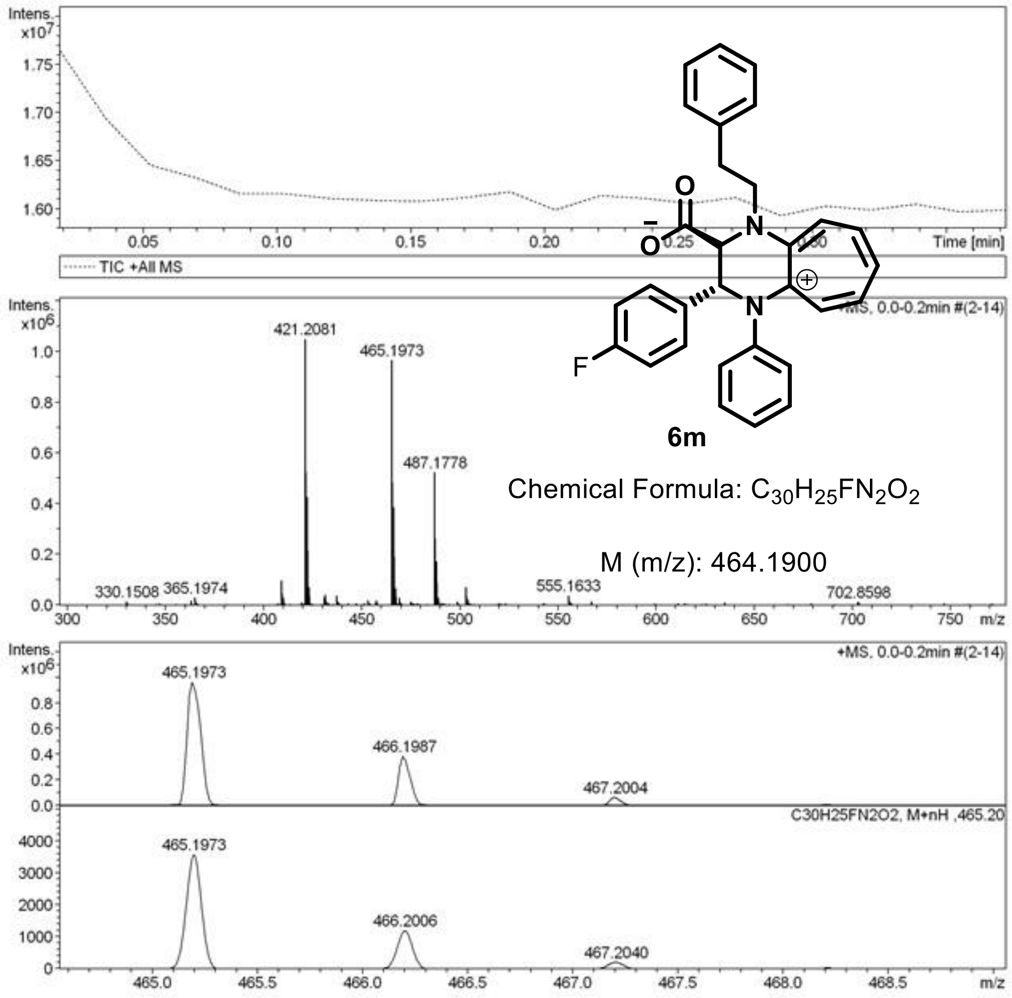

Bruker Compass DataAnalysis 4.0 printed: 10/18/2018 3:32:14 PM Page 1 of 1

Fig S29. ESI/HRMS spectra of compound **(6m)**

14. NMR($^1H/^{13}C$) and HRMS of Cyclic-Aminotropiminium Carboxylate **(6n)**

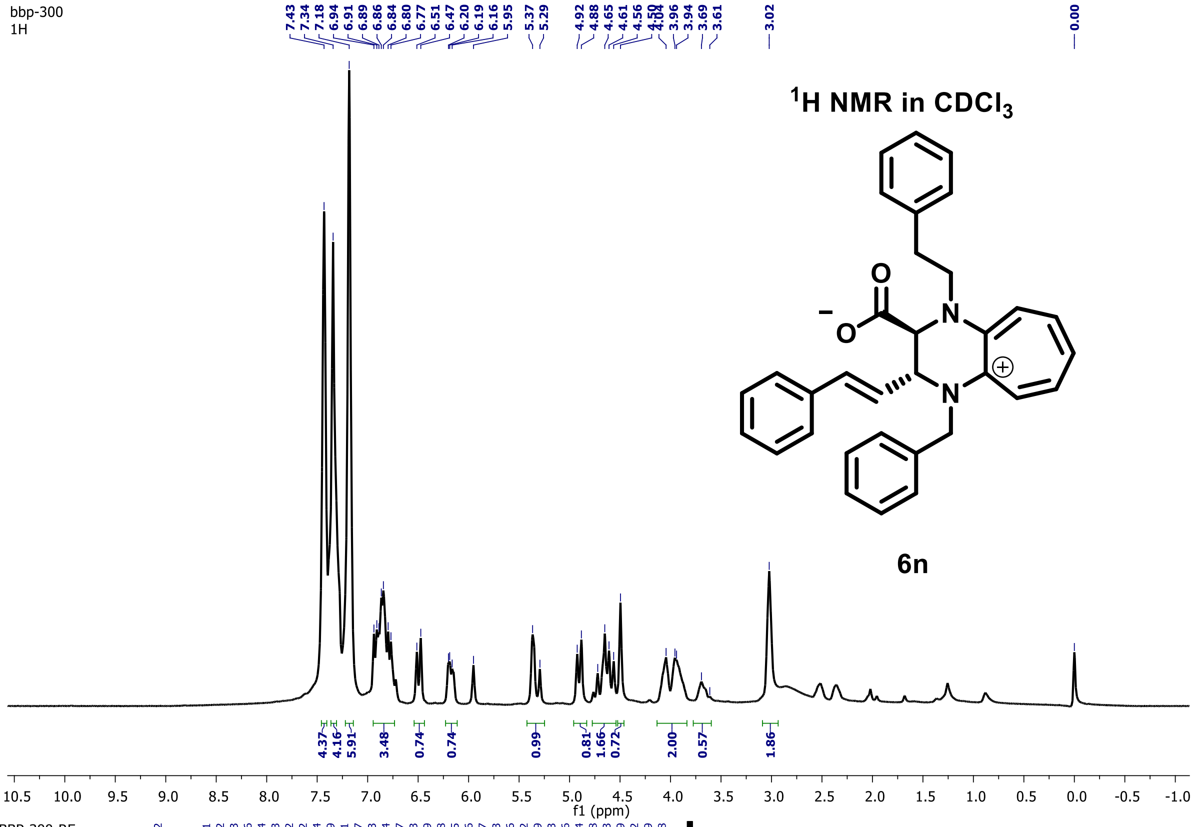

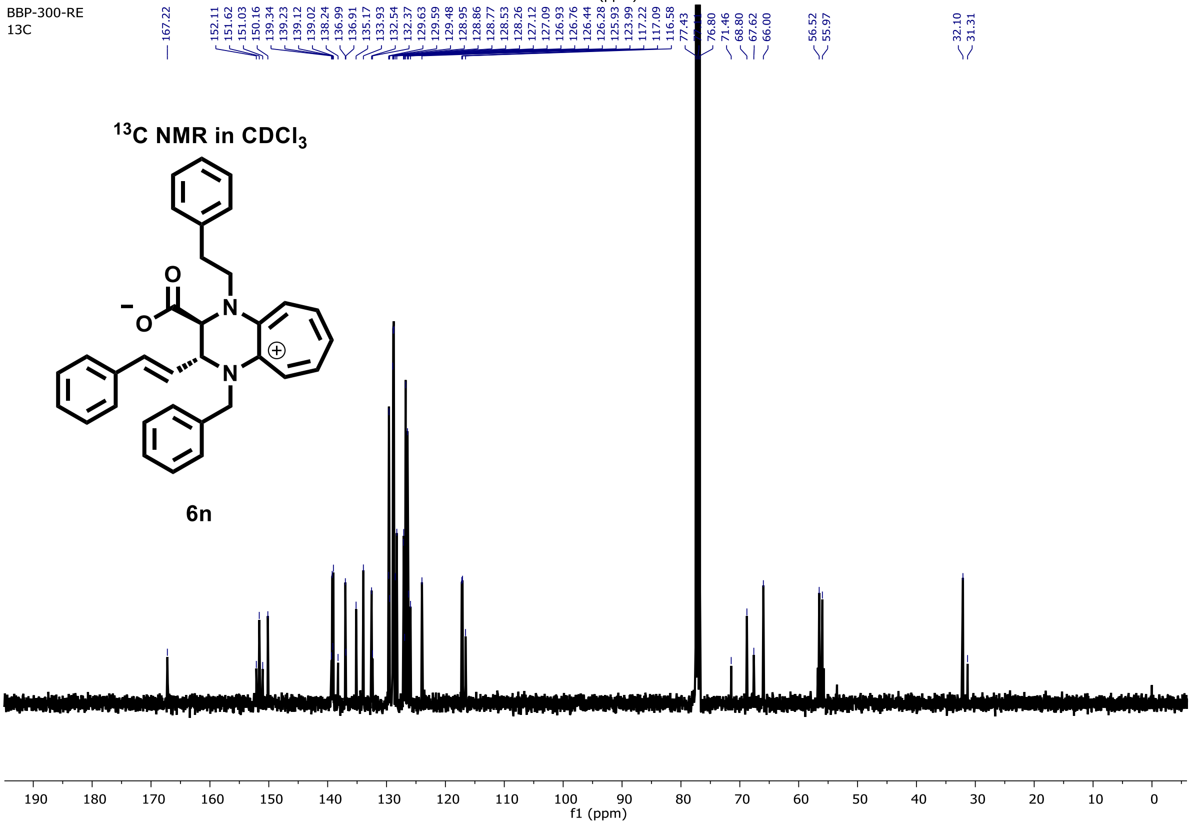

Fig S30. ^1H-/^{13}C-NMR spectra of **(6n)** in $CDCl_3$.

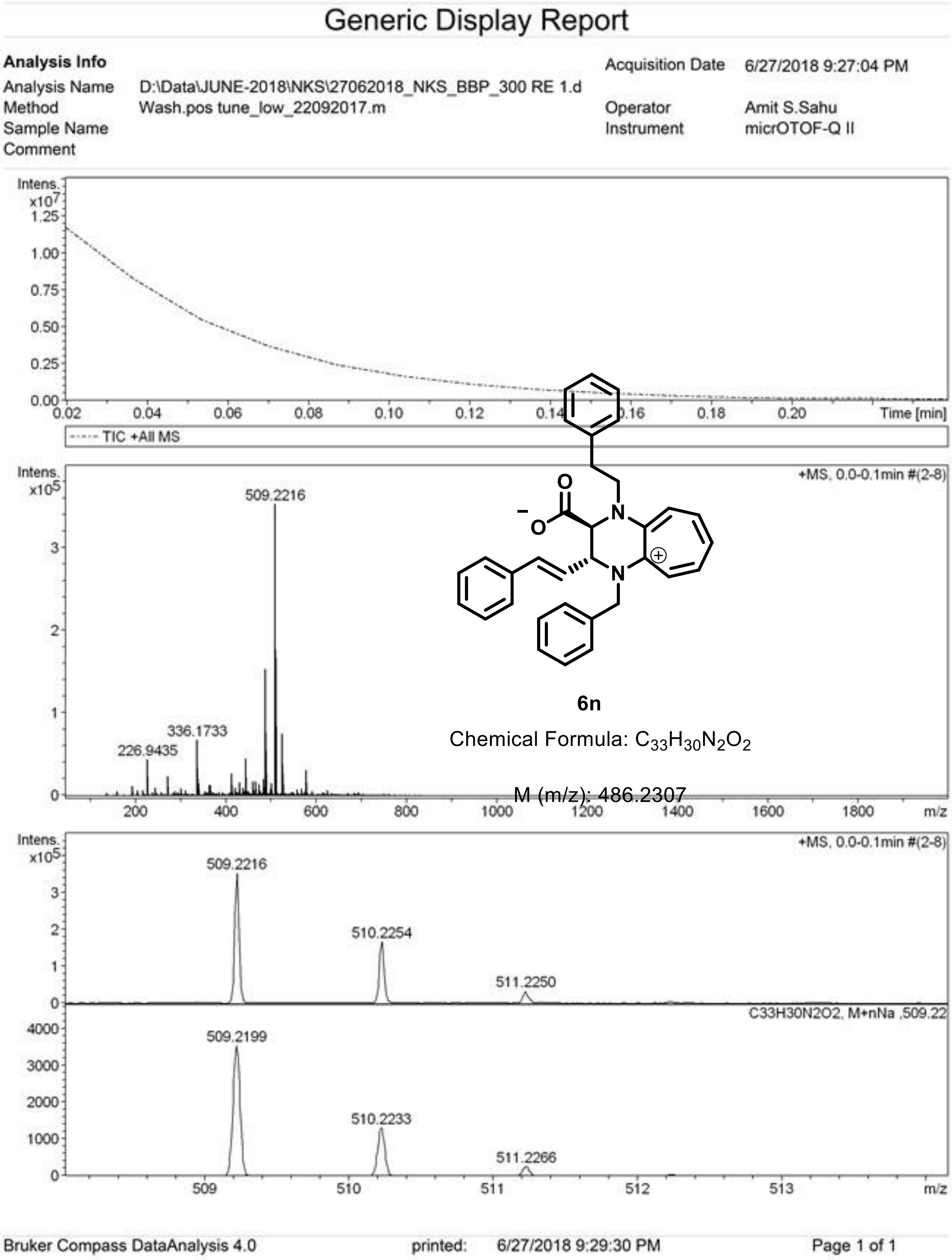

Fig S31. ESI/HRMS spectra of compound **(6n)**

15. NMR(^{1}H/^{13}C) and HRMS of Cyclic-Aminotropiminium Carboxylate **(6o)**

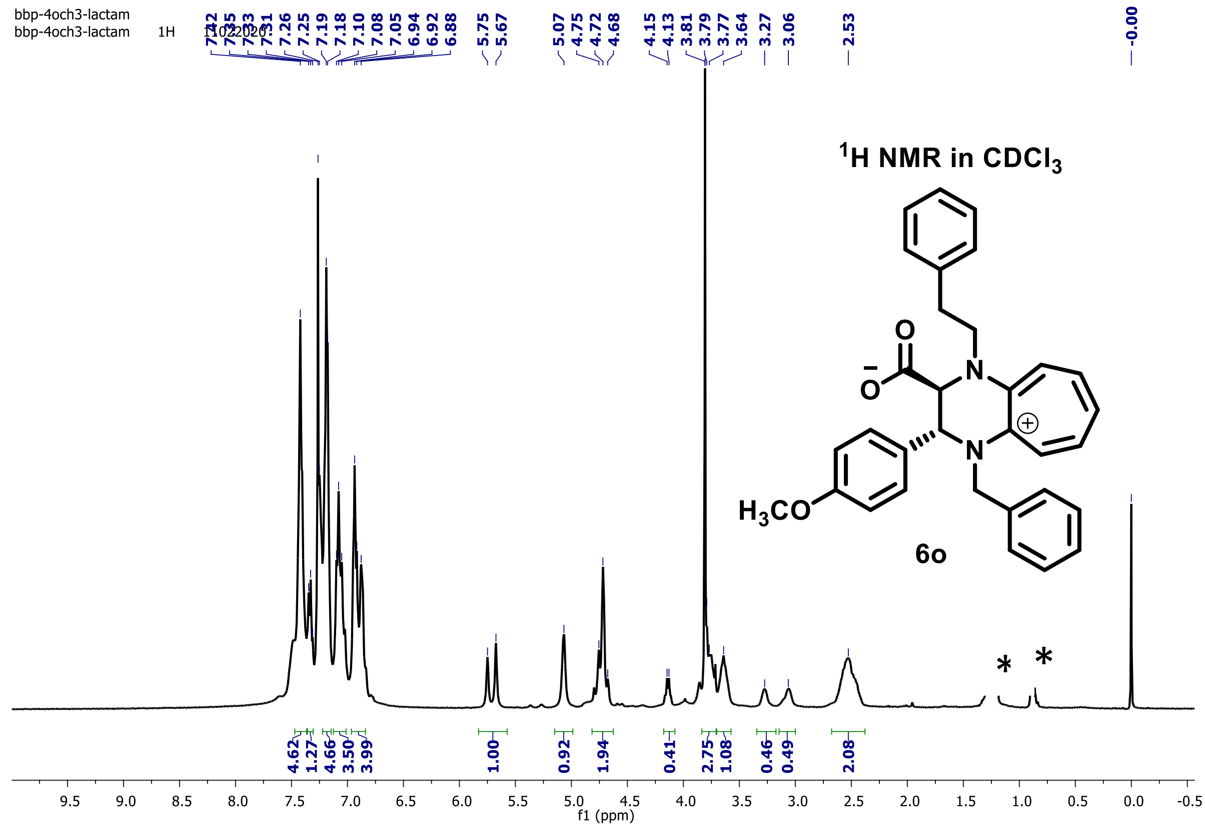

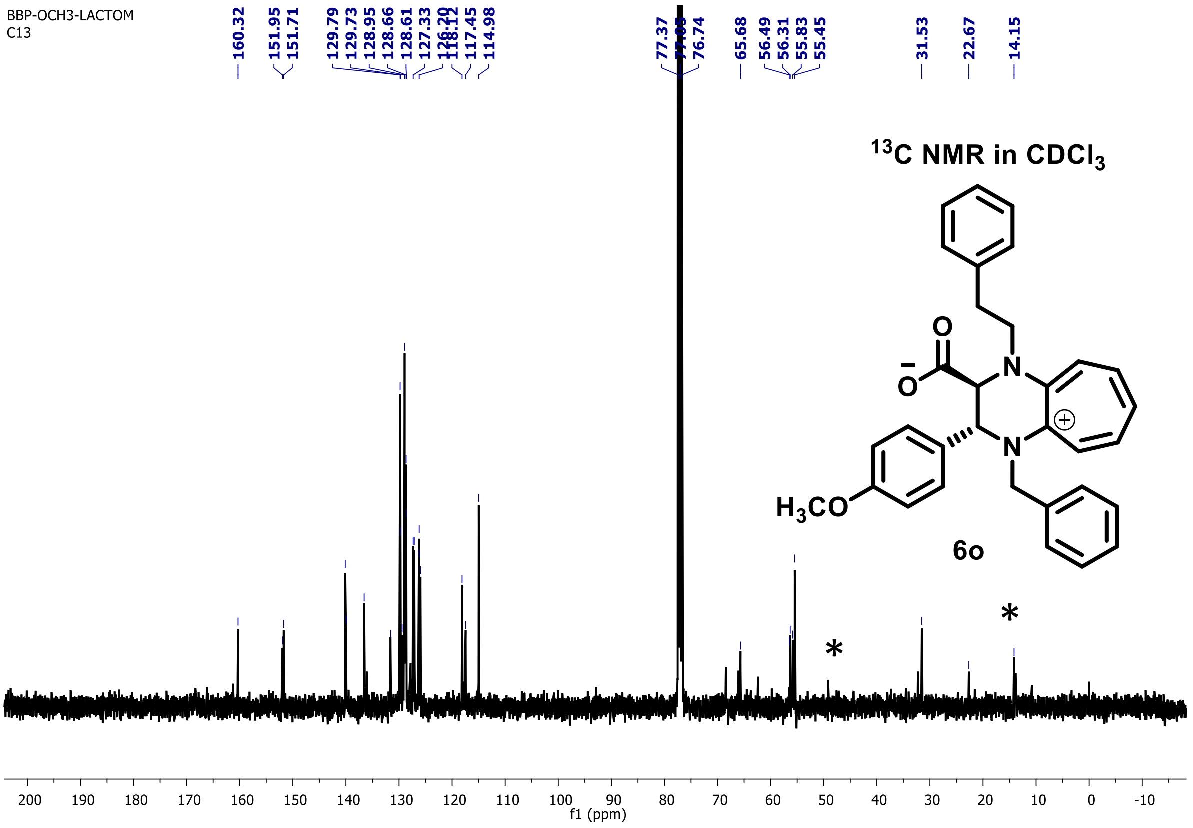

*Residual solvents and impurity grease

Fig S32. ^{1}H-/^{13}C- NMR spectra of **(6n)** in $CDCl_3$

Display Report

Analysis Info

Analysis Name	D:\Data\SEP-2020\NKS\12092020_NKS_BBP_4-OCH3-LAC.d	Acquisition Date	9/12/2020 12:37:45 PM
Method	pos tune_wide_030118.m	Operator	Amit S.Sahu
Sample Name	Tmix-131118	Instrument	micrOTOF-Q II 10337
Comment			

Acquisition Parameter

Source Type	ESI	Ion Polarity	Positive	Set Nebulizer	0.4 Bar
Focus	Not active	Set Capillary	4500 V	Set Dry Heater	180 °C
Scan Begin	50 m/z	Set End Plate Offset	-500 V	Set Dry Gas	4.0 l/min
Scan End	3000 m/z	Set Collision Cell RF	650.0 Vpp	Set Divert Valve	Waste

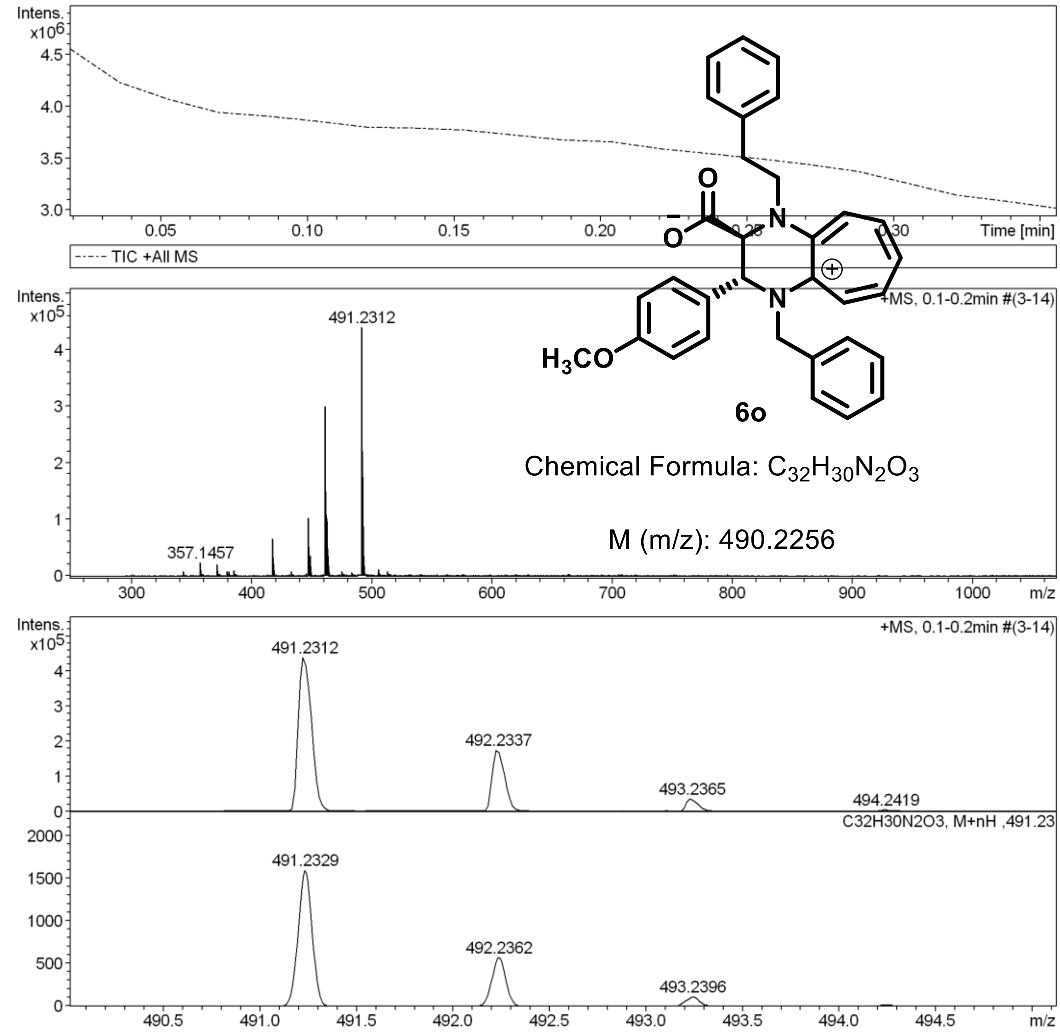

Fig S33. ESI/HRMS spectra of compound **(6o)**

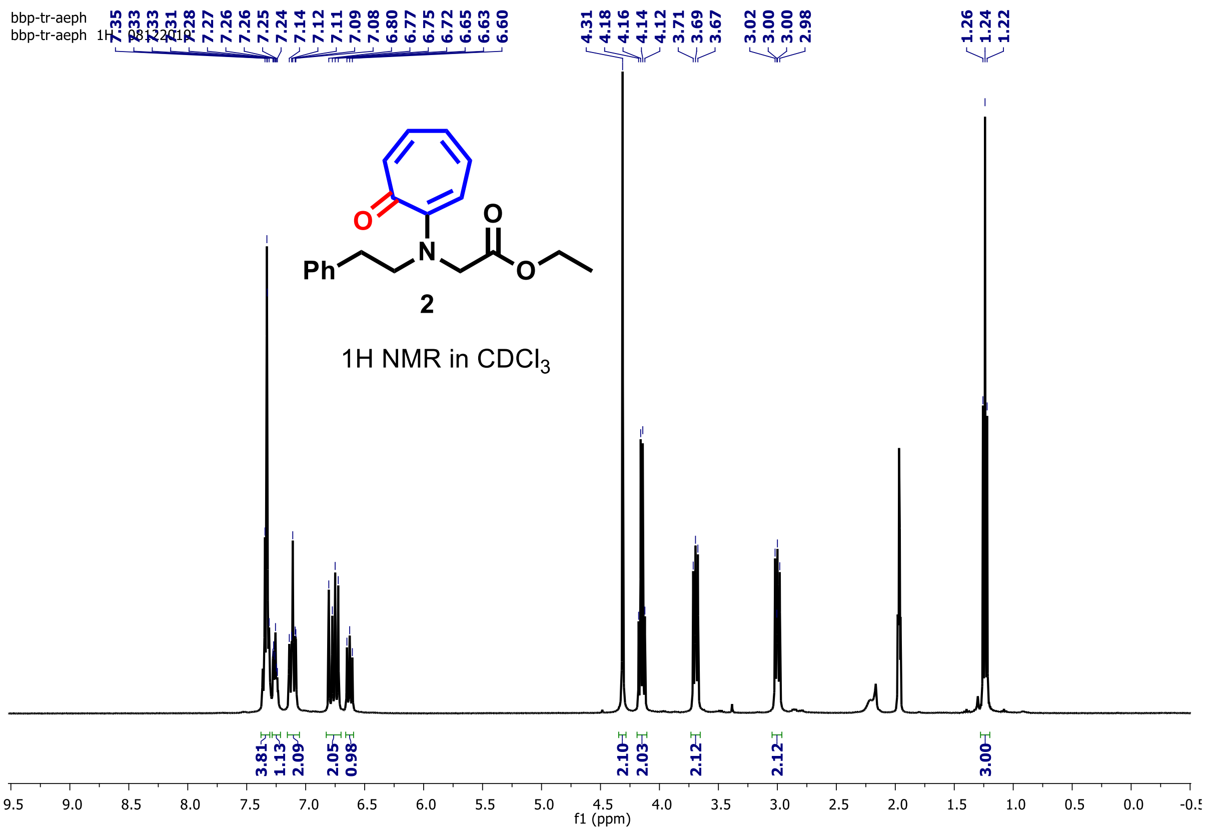

Fig S34. ^{1}H- NMR spectra of **(2)** in $CDCl_3$

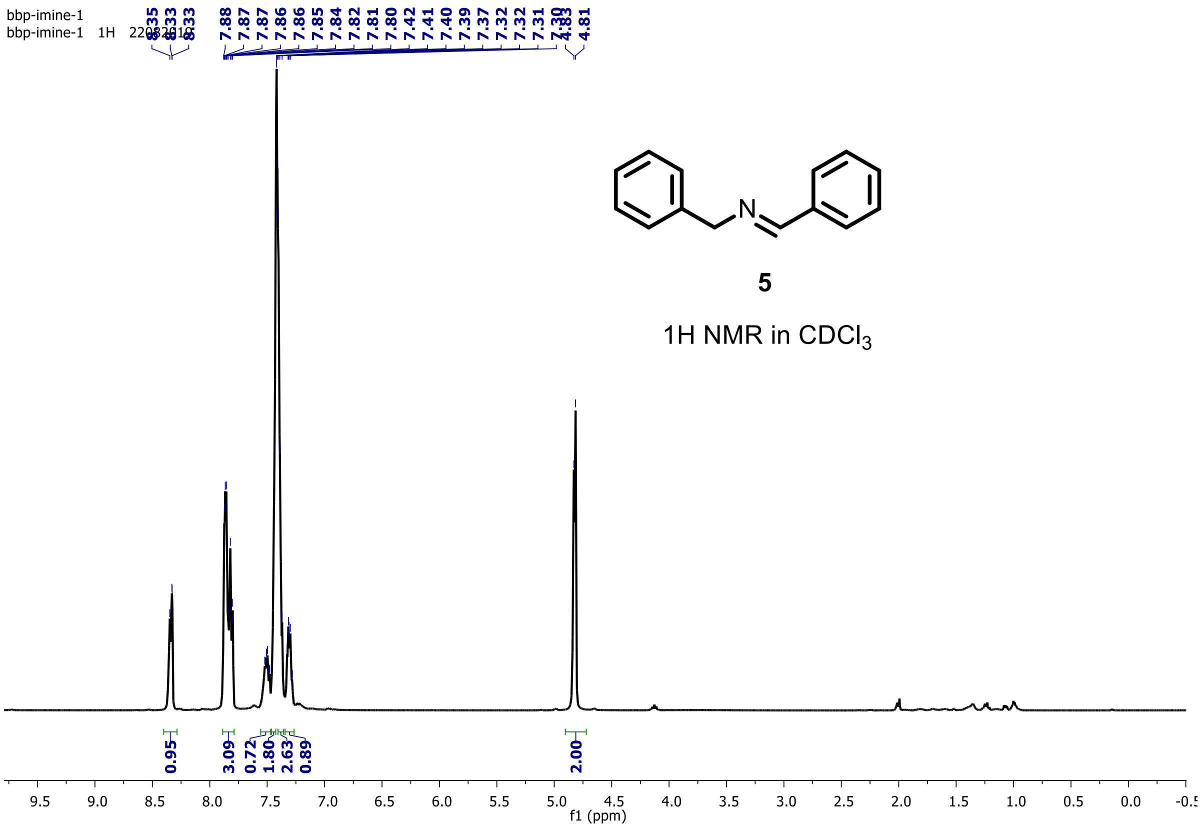

Fig S35. ^{1}H- NMR spectra of **(5)** in $CDCl_3$

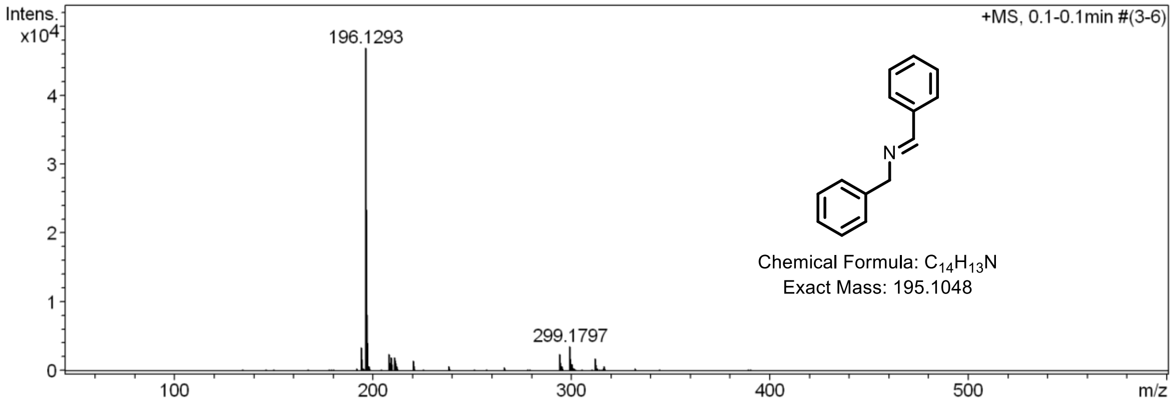

Fig S36. Mass spectra of compound **(5).** Other imines are characterized similarly

1. FT-IR spectra of 6a-6o

FT-IR spectra were recorded by dissolving respective compounds in degassed CH_2Cl_2, drop casted on KBr plate and dried thoroughly. The samples were scanned from 500-4500 ($\tilde{\nu}$,cm^{-1}), the spectra are obtained from average of 32 scans.

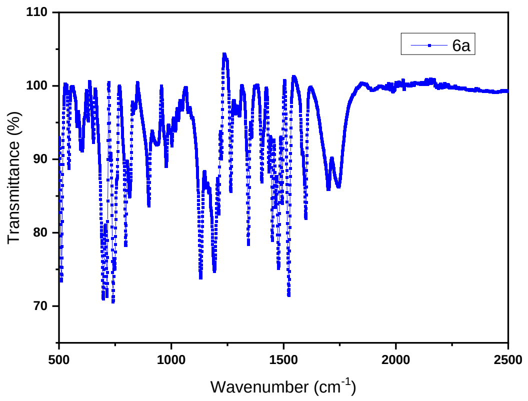

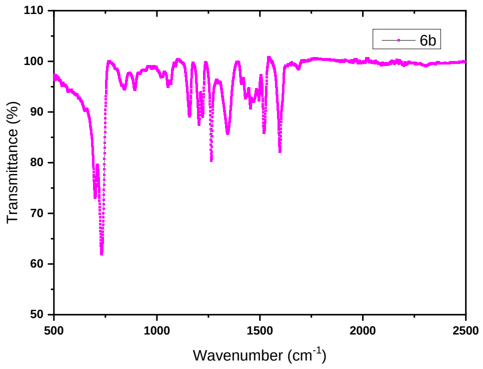

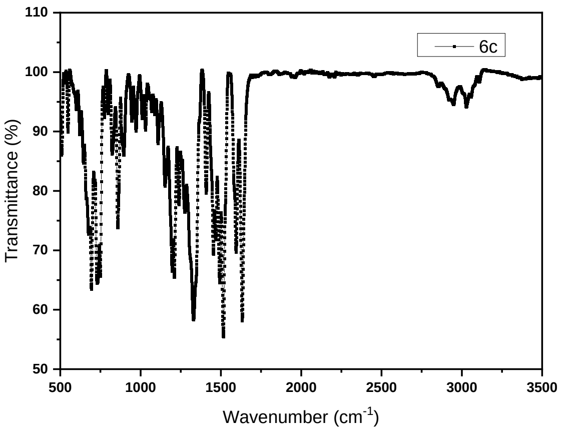

Fig S37: FT-IR spectra of cATC derivative **6a-6c.**

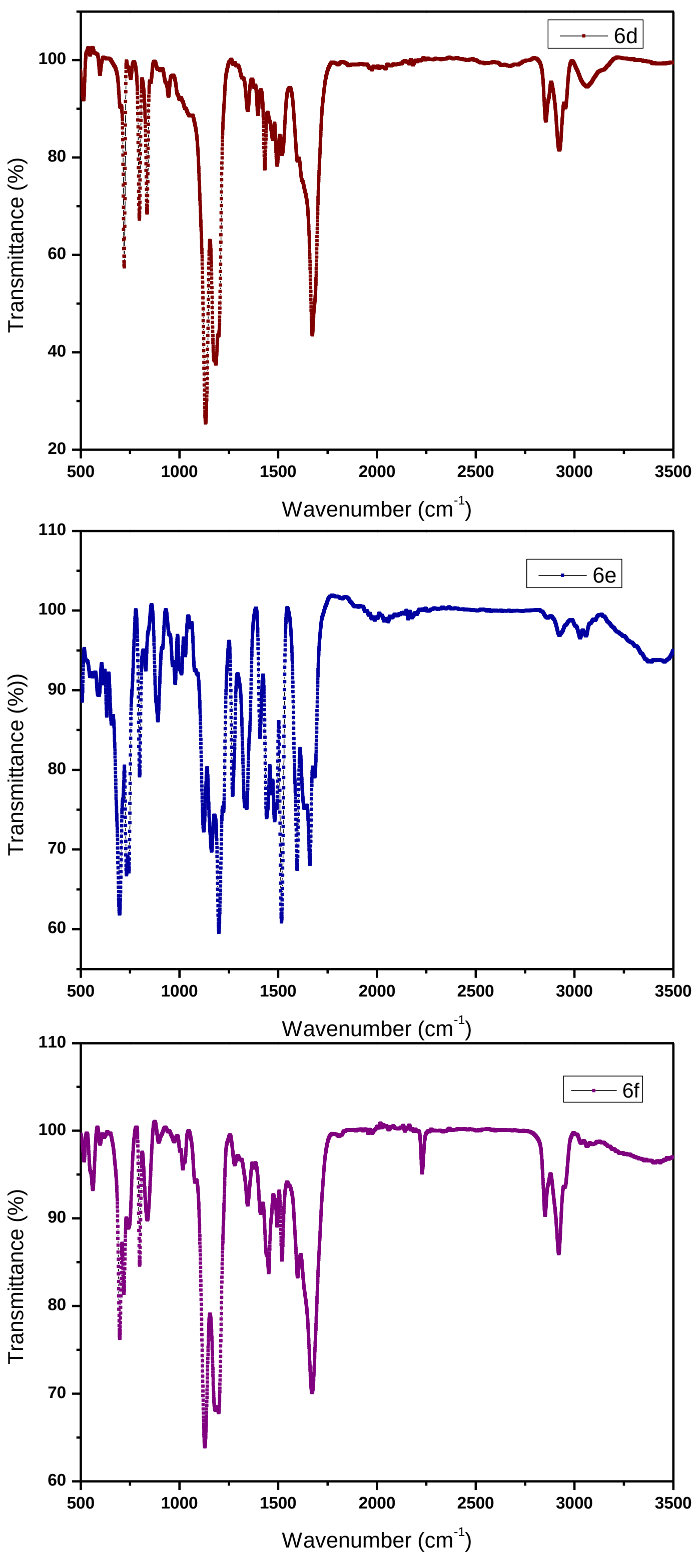

Fig S38: FT-IR spectra of cATC derivative **6d-6f**

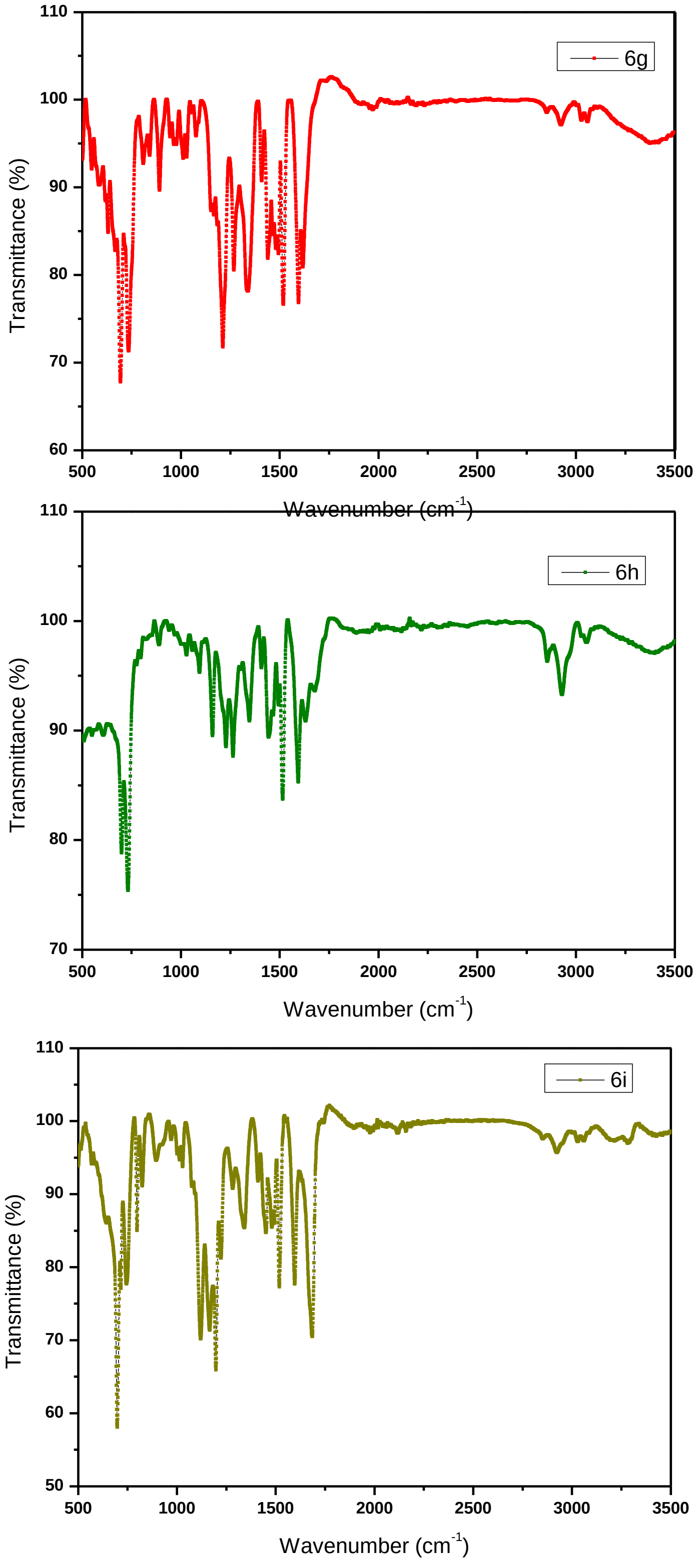

Fig S39: FT-IR spectra of cATC derivative **6g-6i**

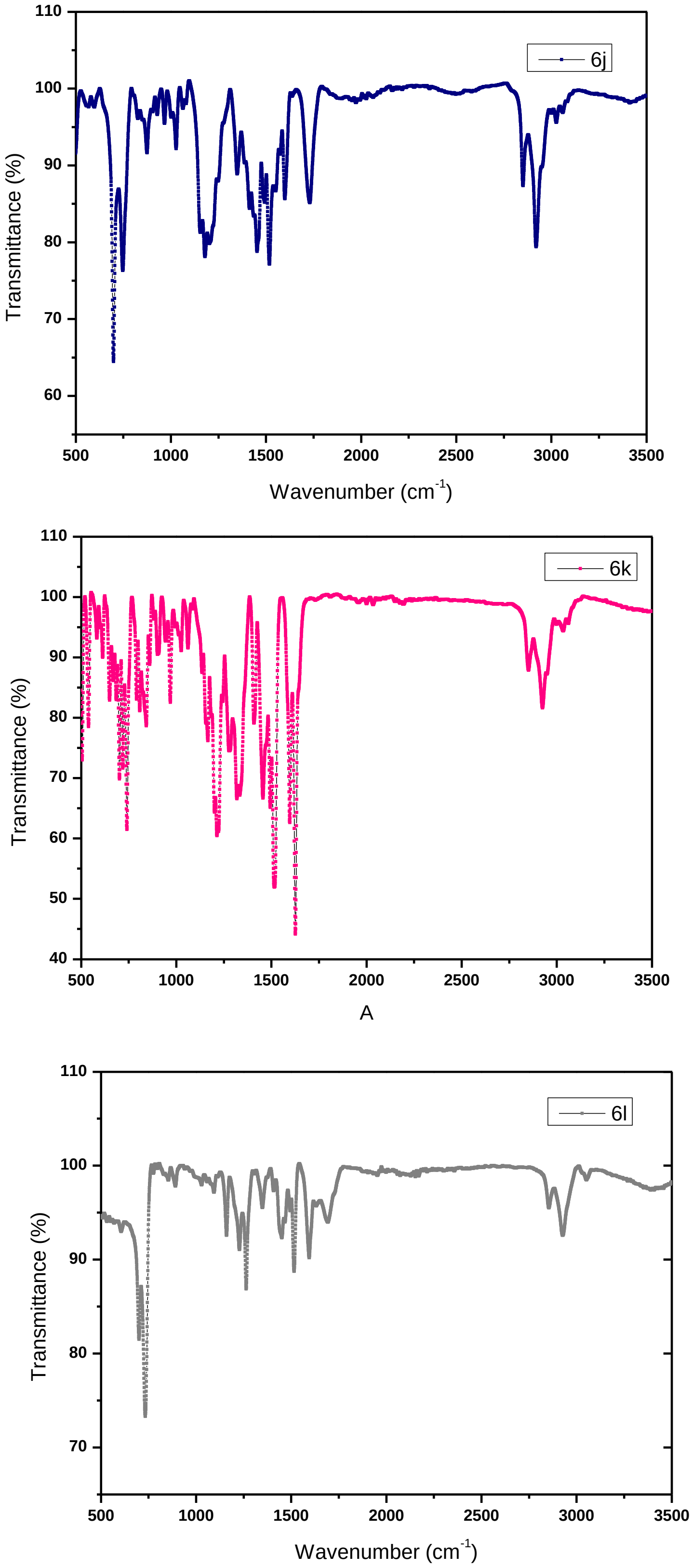

Fig S40: FT-IR spectra of cATC derivative **6j-6l**

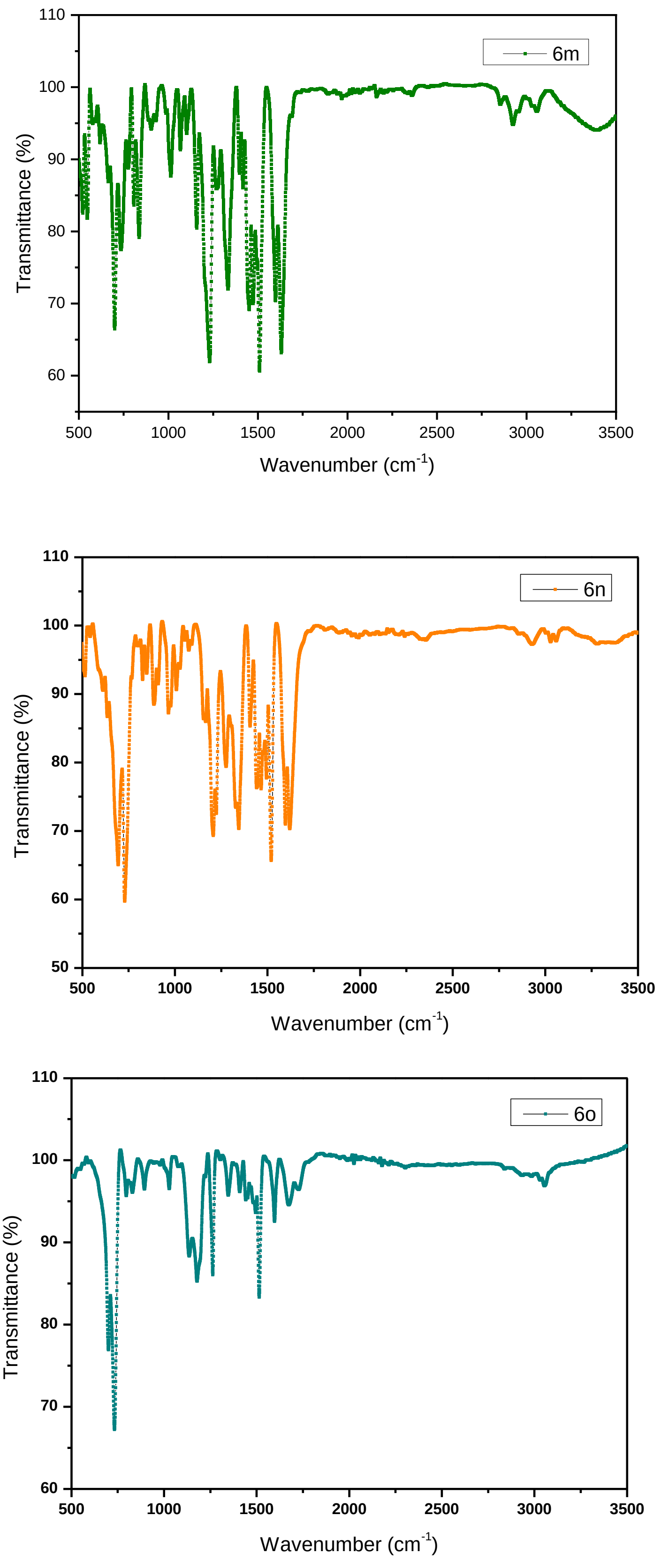

Fig S41: FT-IR spectra of cATC derivative **6m-6o.**
