# Supplementary material for: One-pot synthesis of cyclic-aminotropiminium carboxylate derivatives with DNA binding and anticancer properties
Source: Commun Chem. 2022 Dec 27;5:179. doi: 10.1038/s42004-022-00798-x (PMC9814901; doi:10.1038/s42004-022-00798-x)

checkCIF/PLATON report

You have not supplied any structure factors. As a result the full set of tests cannot be run.

THIS REPORT IS FOR GUIDANCE ONLY. IF USED AS PART OF A REVIEW PROCEDURE FOR PUBLICATION, IT SHOULD NOT REPLACE THE EXPERTISE OF AN EXPERIENCED CRYSTALLOGRAPHIC REFEREE.

No syntax errors found.   CIF dictionary   Interpreting this report

Datablock: nks_bbp_252_no

```
Bond precision:  C-C = 0.0134 A          Wavelength=1.54184

Cell:            a=9.6632(8)    b=22.944(4)          c=16.041(2)
                 alpha=90       beta=101.653(12)     gamma=90
Temperature:     297 K

                Calculated                     Reported
Volume          3483.2(8)                      3483.3(9)
Space group     P 21/n                         P 1 21/n 1
Hall group      -P 2yn                         -P 2yn
Moiety formula  C31 H28 N3 O4 [+ solvent]      C31 H28 N3 O4
Sum formula     C31 H28 N3 O4 [+ solvent]      C31 H28 N3 O4
Mr              506.56                         506.56
Dx,g cm-3       0.966                          0.966
Z               4                              4
Mu (mm-1)       0.521                          0.521
F000            1068.0                         1068.0
F000'           1071.23
h,k,lmax        11,27,19                       11,27,19
Nref            6386                           6151
Tmin,Tmax       0.882,0.949                    0.553,1.000
Tmin'           0.855

Correction method= # Reported T Limits: Tmin=0.553 Tmax=1.000
AbsCorr = MULTI-SCAN

Data completeness= 0.963          Theta(max)= 68.250

R(reflections)= 0.1592( 2266)     wR2(reflections)= 0.4536( 6151)

S = 1.192                  Npar= 344
```

The following ALERTS were generated. Each ALERT has the format
test-name_ALERT_alert-type_alert-level.
Click on the hyperlinks for more details of the test.

Alert level B

```
RINTA01_ALERT_3_B  The value of Rint is greater than 0.18
            Rint given   0.213
PLAT020_ALERT_3_B The Value of Rint is Greater Than 0.12 .........      0.213 Report
PLAT026_ALERT_3_B Ratio Observed / Unique Reflections (too) Low ..        37% Check
PLAT082_ALERT_2_B High R1 Value ..................................       0.16 Report
PLAT084_ALERT_3_B High wR2 Value (i.e. > 0.25) ...................       0.45 Report
PLAT230_ALERT_2_B Hirshfeld Test Diff for    N1        --C23      .      12.2 s.u.
PLAT230_ALERT_2_B Hirshfeld Test Diff for    N3        --C20      .       7.4 s.u.
PLAT230_ALERT_2_B Hirshfeld Test Diff for    C9        --C14      .       9.1 s.u.
PLAT230_ALERT_2_B Hirshfeld Test Diff for    C24       --C25      .      10.4 s.u.
PLAT340_ALERT_3_B Low Bond Precision on  C-C Bonds ...............    0.01342 Ang.
PLAT420_ALERT_2_B D-H Without Acceptor       O4        --H4       .     Please Check
```

Alert level C

```
PLAT029_ALERT_3_C _diffrn_measured_fraction_theta_full value Low .      0.963 Why?
PLAT213_ALERT_2_C Atom N1               has ADP max/min Ratio .....       3.9 prolat
PLAT213_ALERT_2_C Atom C2               has ADP max/min Ratio .....       3.4 oblate
PLAT213_ALERT_2_C Atom C15              has ADP max/min Ratio .....       3.2 oblate
PLAT230_ALERT_2_C Hirshfeld Test Diff for    C16       --C17      .       6.0 s.u.
PLAT230_ALERT_2_C Hirshfeld Test Diff for    C18       --C19      .       6.5 s.u.
PLAT230_ALERT_2_C Hirshfeld Test Diff for    C28       --C29      .       6.5 s.u.
PLAT234_ALERT_4_C Large Hirshfeld Difference O2        --N3       .      0.16 Ang.
PLAT234_ALERT_4_C Large Hirshfeld Difference O3        --C00E     .      0.16 Ang.
PLAT234_ALERT_4_C Large Hirshfeld Difference C3        --C4       .      0.16 Ang.
PLAT234_ALERT_4_C Large Hirshfeld Difference C11       --C12      .      0.22 Ang.
PLAT234_ALERT_4_C Large Hirshfeld Difference C17       --C22      .      0.19 Ang.
PLAT234_ALERT_4_C Large Hirshfeld Difference C19       --C20      .      0.18 Ang.
PLAT234_ALERT_4_C Large Hirshfeld Difference C20       --C21      .      0.17 Ang.
PLAT234_ALERT_4_C Large Hirshfeld Difference C21       --C22      .      0.23 Ang.
PLAT234_ALERT_4_C Large Hirshfeld Difference C23       --C24      .      0.18 Ang.
PLAT234_ALERT_4_C Large Hirshfeld Difference C25       --C26      .      0.17 Ang.
PLAT234_ALERT_4_C Large Hirshfeld Difference C26       --C27      .      0.19 Ang.
PLAT241_ALERT_2_C High   'MainMol' Ueq as Compared to Neighbors of        C6 Check
PLAT241_ALERT_2_C High   'MainMol' Ueq as Compared to Neighbors of       C11 Check
PLAT241_ALERT_2_C High   'MainMol' Ueq as Compared to Neighbors of       C12 Check
PLAT241_ALERT_2_C High   'MainMol' Ueq as Compared to Neighbors of       C14 Check
PLAT241_ALERT_2_C High   'MainMol' Ueq as Compared to Neighbors of       C19 Check
PLAT241_ALERT_2_C High   'MainMol' Ueq as Compared to Neighbors of       C21 Check
PLAT241_ALERT_2_C High   'MainMol' Ueq as Compared to Neighbors of       C22 Check
PLAT241_ALERT_2_C High   'MainMol' Ueq as Compared to Neighbors of       C24 Check
PLAT241_ALERT_2_C High   'MainMol' Ueq as Compared to Neighbors of       C29 Check
PLAT242_ALERT_2_C Low    'MainMol' Ueq as Compared to Neighbors of        C7 Check
PLAT242_ALERT_2_C Low    'MainMol' Ueq as Compared to Neighbors of        C9 Check
PLAT242_ALERT_2_C Low    'MainMol' Ueq as Compared to Neighbors of       C13 Check
PLAT242_ALERT_2_C Low    'MainMol' Ueq as Compared to Neighbors of       C17 Check
PLAT242_ALERT_2_C Low    'MainMol' Ueq as Compared to Neighbors of       C20 Check
PLAT242_ALERT_2_C Low    'MainMol' Ueq as Compared to Neighbors of       C30 Check
PLAT250_ALERT_2_C Large U3/U1 Ratio for Average U(i,j) Tensor ....        2.4 Note
PLAT260_ALERT_2_C Large Average Ueq of Residue Including       O1       0.101 Check
PLAT360_ALERT_2_C Short  C(sp3)-C(sp3) Bond  C23       - C24      .      1.34 Ang.
PLAT363_ALERT_2_C Long   C(sp3)-C(sp2) Bond  C24       - C25      .      1.71 Ang.
PLAT369_ALERT_2_C Long   C(sp2)-C(sp2) Bond  C17       - C22      .      1.53 Ang.
PLAT410_ALERT_2_C Short Intra H...H Contact  H7        ..H8A      .      1.94 Ang.
                                                       x,y,z  =      1_555 Check
```

Alert level G

```
PLAT007_ALERT_5_G Number of Unrefined Donor-H Atoms ..............          1 Report
PLAT012_ALERT_1_G No       _shelx_res_checksum Found in CIF ......     Please Check
```

```
PLAT072_ALERT_2_G SHELXL First  Parameter in WGHT  Unusually Large       0.20 Report
PLAT335_ALERT_2_G Check Large C6 Ring C-C Range C17      -C22            0.23 Ang.
PLAT606_ALERT_4_G Solvent Accessible VOID(S) in Structure ........          ! Info
PLAT720_ALERT_4_G Number of Unusual/Non-Standard Labels ..........          1 Note
PLAT773_ALERT_2_G Check long C-C Bond in CIF: C25      --C24             1.71 Ang.
PLAT793_ALERT_4_G Model has Chirality at C15         (Centro SPGR)          R Verify
PLAT793_ALERT_4_G Model has Chirality at C16         (Centro SPGR)          S Verify
PLAT868_ALERT_4_G ALERTS Due to the Use of _smtbx_masks Suppressed          ! Info
PLAT933_ALERT_2_G Number of OMIT Records in Embedded .res File ...        235 Note
```

```
  0 ALERT level A = Most likely a serious problem - resolve or explain
 11 ALERT level B = A potentially serious problem, consider carefully
 39 ALERT level C = Check. Ensure it is not caused by an omission or oversight
 11 ALERT level G = General information/check it is not something unexpected

  1 ALERT type 1 CIF construction/syntax error, inconsistent or missing data
 37 ALERT type 2 Indicator that the structure model may be wrong or deficient
  6 ALERT type 3 Indicator that the structure quality may be low
 16 ALERT type 4 Improvement, methodology, query or suggestion
  1 ALERT type 5 Informative message, check
```

It is advisable to attempt to resolve as many as possible of the alerts in all categories. Often the minor alerts point to easily fixed oversights, errors and omissions in your CIF or refinement strategy, so attention to these fine details can be worthwhile. In order to resolve some of the more serious problems it may be necessary to carry out additional measurements or structure refinements. However, the purpose of your study may justify the reported deviations and the more serious of these should normally be commented upon in the discussion or experimental section of a paper or in the "special_details" fields of the CIF. checkCIF was carefully designed to identify outliers and unusual parameters, but every test has its limitations and alerts that are not important in a particular case may appear. Conversely, the absence of alerts does not guarantee there are no aspects of the results needing attention. It is up to the individual to critically assess their own results and, if necessary, seek expert advice.

Publication of your CIF in IUCr journals

A basic structural check has been run on your CIF. These basic checks will be run on all CIFs submitted for publication in IUCr journals (*Acta Crystallographica*, *Journal of Applied Crystallography*, *Journal of Synchrotron Radiation*); however, if you intend to submit to *Acta Crystallographica Section C* or *E* or *IUCrData*, you should make sure that full publication checks are run on the final version of your CIF prior to submission.

Publication of your CIF in other journals

Please refer to the *Notes for Authors* of the relevant journal for any special instructions relating to CIF submission.

PLATON version of 10/08/2020; check.def file version of 06/08/2020

Datablock nks_bbp_252_no - ellipsoid plot

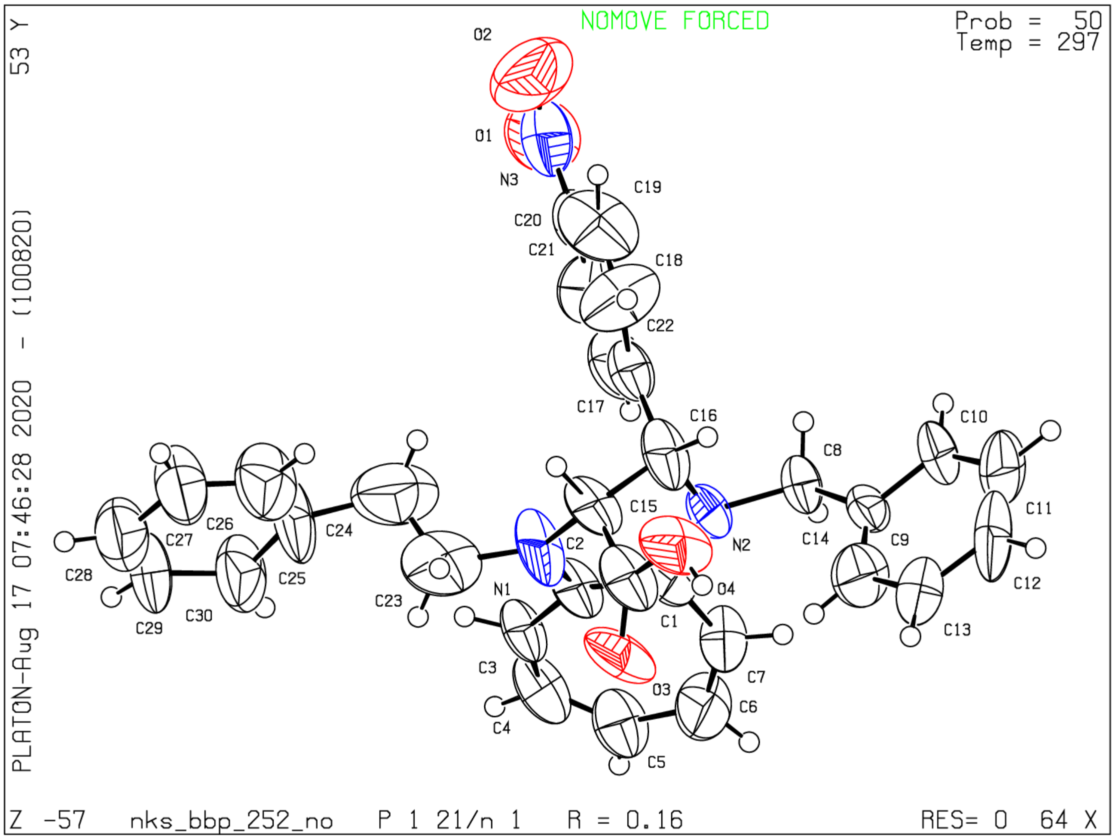

Supplement: Supplementary file 7 — Supplementary Data 4 [file 42004_2022_798_MOESM7_ESM.pdf]
