# Supplementary material for: One-pot synthesis of cyclic-aminotropiminium carboxylate derivatives with DNA binding and anticancer properties
Source: Commun Chem. 2022 Dec 27;5:179. doi: 10.1038/s42004-022-00798-x (PMC9814901; doi:10.1038/s42004-022-00798-x)
Supplement: Supplementary file 9 — Supplementary Data 6 [file 42004_2022_798_MOESM9_ESM.pdf]

Supplemental Material

One-Pot synthesis of cyclic-aminotropiminium carboxylate derivatives with DNA binding and anticancer properties

Bibhuti Bhusana Palai[a,c,#], Saket Awadhesbhai Patel[b,c,#], Nagendra K. Sharma*[,a.c], Manjusha Dixit*[,b,c]

[#]Authors equally contributed

*Corresponding Authors

[a]School of Chemical Sciences, National Institute of Science Education and Research (NISER) Bhubaneswar, PO: Jatani-752050, Odisha, India, Phone no. +91-674-249-4141; E-mail: nagendra@niser.ac.in

[b]School of Biological Sciences, National Institute of Science Education and Research (NISER) Bhubaneswar, PO: Jatani-752050, Odisha, India, Phone no. +91-674-249-4195; E-mail: manjusha@niser.ac.in

[c]Homi Bhabha National Institute, Training School Complex, Anushaktinagar, Mumbai 400094, India

Computational Data of Intermediates

Optimized Cartesian Coordinates of Various Stationary Points

Coordinates of geometries of all stationary points obtained at B3LYP/6-311G (d,p) level of theory

----

Rct-1

Number of imaginary frequencies: 0

Zero-point correction= 0.167963 (Hartree/Particle)

Thermal correction to Energy= 0.180006

Thermal correction to Enthalpy= 0.180950

Thermal correction to Gibbs free energy= 0.128237

Sum of electronic and zero-point Energies= -591.584927

Sum of electronic and thermal Energies= -591.572884

Sum of electronic and thermal Enthalpies= -591.571940

Sum of electronic and thermal Free Energies= -591.624653

Cartesian Coordinates

C	-0.419419000	-1.298749000	0.214341000
C	-1.659709000	-1.963105000	0.097938000
C	-2.930293000	-1.450501000	0.013806000
C	-3.274012000	-0.080480000	-0.047341000
C	-2.455083000	1.007726000	-0.169633000
C	-1.014513000	1.118412000	-0.330435000
C	-0.091333000	0.048190000	0.168236000

H	0.420791000	-1.962523000	0.382873000
H	-1.586523000	-3.046111000	0.141992000
H	-3.751142000	-2.159456000	0.006606000
H	-4.339117000	0.135902000	-0.038321000
H	-2.933062000	1.975980000	-0.285432000
N	1.202037000	0.452427000	0.449847000
C	1.529039000	1.842956000	0.831394000
H	0.706853000	2.296080000	1.385680000
H	1.736732000	2.462263000	-0.040396000
H	2.405340000	1.800372000	1.477753000
C	2.217147000	-0.513286000	0.710675000
H	2.326534000	-0.992681000	1.680601000
C	3.095467000	-0.789486000	-0.236359000
O	-0.539882000	2.129970000	-0.853836000
O	3.866831000	-1.030830000	-1.068165000

Rct-2

Number of imaginary frequencies: 0

Zero-point correction= 0.177659 (Hartree/Particle)

Thermal correction to Energy= 0.186995

Thermal correction to Enthalpy= 0.187939

Thermal correction to Gibbs free energy= 0.142502

Sum of electronic and zero-point Energies= -404.254732

Sum of electronic and thermal Energies= -404.245397

Sum of electronic and thermal Enthalpies= -404.244452

Sum of electronic and thermal Free Energies= -404.289890

Cartesian Coordinates

C	1.519843000	-1.272460000	0.089805000
C	0.564639000	-0.262623000	-0.078273000
C	2.873638000	-0.959538000	0.194925000
H	3.603914000	-1.750263000	0.324806000
C	0.988564000	1.073255000	-0.139855000
C	3.285046000	0.369844000	0.132814000
H	0.243443000	1.848421000	-0.270481000
H	4.337593000	0.617369000	0.214105000
C	2.338135000	1.384342000	-0.034987000
H	2.658307000	2.419275000	-0.083600000
H	1.198629000	-2.308385000	0.138044000
C	-0.859542000	-0.626951000	-0.186164000
H	-1.074241000	-1.706621000	-0.127070000
N	-1.790140000	0.223012000	-0.338245000
C	-3.159485000	-0.255707000	-0.429461000
H	-3.541627000	0.031590000	-1.416008000

H -3.213892000 -1.354736000 -0.362252000

C -4.036146000 0.384295000 0.649255000

H -3.995994000 1.473623000 0.579460000

H -3.697267000 0.095813000 1.647752000

H -5.076042000 0.066088000 0.534606000

Ts-1

Number of imaginary frequencies: 1

Zero-point correction= 0.352258 (Hartree/Particle)

Thermal correction to Energy= 0.372709

Thermal correction to Enthalpy= 0.373654

Thermal correction to Gibbs free energy= 0.301301

Sum of electronic and zero-point Energies= -995.874781

Sum of electronic and thermal Energies= -995.854330

Sum of electronic and thermal Enthalpies= -995.853386

Sum of electronic and thermal Free Energies= -995.925738

Cartesian Coordinates

C 1.817389000 1.074319000 -0.162850000

C 2.614865000 2.229922000 -0.325601000

C 3.972330000 2.388322000 -0.217685000

C 4.909187000 1.367347000 0.064948000

C 4.708003000 0.024786000 0.220865000

C	3.529335000	-0.821212000	0.099142000
C	2.136991000	-0.260242000	0.049329000
H	0.756996000	1.294281000	-0.195294000
H	2.044385000	3.133637000	-0.520595000
H	4.366777000	3.390879000	-0.343754000
H	5.942597000	1.693687000	0.149951000
H	5.591806000	-0.585664000	0.380190000
N	1.117342000	-1.191365000	0.122577000
C	1.204003000	-2.400584000	0.959576000
H	2.007638000	-2.310807000	1.682323000
H	1.367418000	-3.293851000	0.357875000
H	0.262982000	-2.503423000	1.509793000
C	-0.167273000	-0.924726000	-0.457546000
H	-0.057054000	-0.267067000	-1.319994000
C	-1.061230000	-2.144639000	-0.819428000
O	3.689115000	-2.043162000	0.035898000
O	-0.919406000	-3.166837000	-1.443789000
C	-1.911749000	1.876777000	1.195538000
C	-1.983773000	0.897815000	0.199293000
C	-2.398847000	3.163213000	0.968856000
H	-2.337999000	3.908581000	1.753937000

C	-2.560722000	1.233208000	-1.032218000
C	-2.969505000	3.486984000	-0.260310000
H	-2.637716000	0.481865000	-1.811048000
H	-3.353292000	4.485166000	-0.437685000
C	-3.049474000	2.516924000	-1.259996000
H	-3.495515000	2.760560000	-2.217826000
H	-1.472615000	1.629866000	2.157185000
C	-1.404617000	-0.471227000	0.443383000
H	-1.179368000	-0.603795000	1.506743000
N	-2.125815000	-1.624942000	-0.135846000
C	-3.414524000	-2.256669000	0.150848000
H	-3.905943000	-2.471382000	-0.802785000
H	-3.224498000	-3.223539000	0.631219000
C	-4.319890000	-1.402972000	1.032848000
H	-4.574676000	-0.454518000	0.557451000
H	-3.855735000	-1.187694000	1.998628000
H	-5.247550000	-1.946712000	1.225741000

Int-1

Number of imaginary frequencies: 0

Zero-point correction= 0.352341 (Hartree/Particle)

Thermal correction to Energy= 0.373611

Thermal correction to Enthalpy= 0.374556

Thermal correction to Gibbs free energy= 0.299636

Sum of electronic and zero-point Energies= -995.877275

Sum of electronic and thermal Energies= -995.856005

Sum of electronic and thermal Enthalpies= -995.855061

Sum of electronic and thermal Free Energies= -995.929980

Cartesian Coordinates

C	1.834337000	1.046357000	-0.206365000
C	2.636086000	2.186276000	-0.443066000
C	3.998297000	2.332502000	-0.396407000
C	4.935781000	1.311611000	-0.115086000
C	4.727827000	-0.021798000	0.099081000
C	3.536539000	-0.857931000	0.059159000
C	2.149244000	-0.283039000	0.041713000
H	0.775730000	1.277088000	-0.203756000
H	2.067288000	3.088988000	-0.647296000
H	4.397427000	3.325182000	-0.574986000
H	5.975062000	1.628864000	-0.085115000
H	5.611316000	-0.636111000	0.244694000
N	1.123931000	-1.199499000	0.187580000
C	1.228335000	-2.374513000	1.070170000

H	2.061124000	-2.266537000	1.756461000
H	1.356569000	-3.293758000	0.499934000
H	0.308778000	-2.441850000	1.660762000
C	-0.178883000	-0.937379000	-0.352432000
H	-0.091745000	-0.321661000	-1.248112000
C	-1.104943000	-2.158767000	-0.625876000
O	3.681080000	-2.083253000	0.039222000
O	-0.999505000	-3.212963000	-1.200237000
C	-1.907612000	1.907874000	1.345609000
C	-1.936020000	0.948638000	0.328128000
C	-2.382541000	3.199941000	1.123441000
H	-2.355319000	3.929513000	1.925022000
C	-2.457095000	1.310483000	-0.921209000
C	-2.896038000	3.549255000	-0.123348000
H	-2.502298000	0.575887000	-1.718072000
H	-3.269016000	4.551954000	-0.298395000
C	-2.932429000	2.599351000	-1.145132000
H	-3.334379000	2.863329000	-2.116968000
H	-1.509948000	1.641430000	2.319919000
C	-1.372371000	-0.426578000	0.573396000
H	-1.119203000	-0.542629000	1.633410000

N	-2.139028000	-1.580675000	0.064137000
C	-3.410900000	-2.155187000	0.479700000
H	-3.351815000	-3.219921000	0.238759000
H	-3.485571000	-2.070100000	1.570059000
C	-4.638252000	-1.530139000	-0.186553000
H	-4.585649000	-1.638651000	-1.272311000
H	-4.727158000	-0.468243000	0.050402000
H	-5.544294000	-2.033568000	0.162137000

Ts-2

Number of imaginary frequencies: 1

Zero-point correction= 0.351617 (Hartree/Particle)

Thermal correction to Energy= 0.371524

Thermal correction to Enthalpy= 0.372469

Thermal correction to Gibbs free energy= 0.302412

Sum of electronic and zero-point Energies= -995.814264

Sum of electronic and thermal Energies= -995.794357

Sum of electronic and thermal Enthalpies= -995.793413

Sum of electronic and thermal Free Energies= -995.863470

Cartesian Coordinates

C	-3.440646000	-1.632749000	-0.371556000
C	-4.721228000	-1.420216000	0.183423000

C	-5.249405000	-0.380107000	0.923171000
C	-4.621226000	0.815484000	1.333730000
C	-3.339188000	1.256628000	1.112878000
C	-2.317128000	0.581064000	0.395351000
C	-2.338523000	-0.788901000	-0.288998000
H	-3.348382000	-2.562630000	-0.915365000
H	-5.417080000	-2.231753000	-0.013763000
H	-6.285635000	-0.491171000	1.223798000
H	-5.251077000	1.490610000	1.907210000
H	-3.039975000	2.223001000	1.505208000
N	-1.095058000	-1.129794000	-0.853231000
C	-1.034675000	-2.452502000	-1.488954000
H	-1.333634000	-3.238149000	-0.788906000
H	-0.016252000	-2.662355000	-1.804302000
H	-1.681195000	-2.505534000	-2.372016000
C	0.225685000	-0.335047000	-0.988872000
H	0.831389000	-0.965932000	-1.640691000
C	0.311918000	1.179939000	-1.435003000
O	-1.167688000	0.904231000	0.140766000
O	-0.126916000	1.807344000	-2.363589000
C	2.669082000	-1.389904000	1.446435000

C	2.467816000	-0.484805000	0.397924000
C	3.879088000	-2.067700000	1.585511000
H	4.017630000	-2.761664000	2.407350000
C	3.513576000	-0.266764000	-0.506991000
C	4.911571000	-1.845974000	0.676036000
H	3.378807000	0.447104000	-1.311532000
H	5.855934000	-2.367513000	0.784282000
C	4.724573000	-0.941350000	-0.369030000
H	5.526045000	-0.757195000	-1.076180000
H	1.871774000	-1.560719000	2.163457000
C	1.121949000	0.182684000	0.242796000
H	0.576837000	0.151855000	1.185450000
N	1.121850000	1.548129000	-0.366656000
C	1.233481000	2.845382000	0.267744000
H	0.780471000	3.557654000	-0.426481000
H	0.616320000	2.842926000	1.175725000
C	2.668837000	3.256639000	0.596536000
H	3.275365000	3.317585000	-0.310122000
H	3.143881000	2.544285000	1.275560000
H	2.674709000	4.237841000	1.080317000

Int-2

Number of imaginary frequencies: 0

Zero-point correction= 0.354010 (Hartree/Particle)

Thermal correction to Energy= 0.373843

Thermal correction to Enthalpy= 0.374788

Thermal correction to Gibbs free energy= 0.305609

Sum of electronic and zero-point Energies= -995.868262

Sum of electronic and thermal Energies= -995.848429

Sum of electronic and thermal Enthalpies= -995.847485

Sum of electronic and thermal Free Energies= -995.916664

Cartesian Coordinates

N	-0.272752000	0.632210000	-0.884738000
O	-0.780963000	2.769594000	2.358267000
N	-0.134390000	-0.492230000	1.645779000
O	-1.789472000	1.487796000	0.816252000
C	-1.465084000	0.300348000	-0.140892000
C	-1.126295000	-0.868243000	0.775631000
C	0.398337000	0.848079000	1.424506000
H	1.192576000	1.065823000	2.132004000
C	2.128082000	0.154808000	-0.330193000
C	0.881643000	0.982321000	-0.044580000
H	1.142921000	2.039814000	-0.185111000

C	-1.616863000	-2.147787000	0.746876000
H	-1.144961000	-2.835578000	1.438621000
C	0.387187000	-1.358765000	2.686843000
H	-0.425968000	-1.751394000	3.305428000
H	1.055639000	-0.782342000	3.324515000
C	-0.764078000	1.826564000	1.610450000
C	-0.353791000	1.319083000	-2.179122000
H	-1.100342000	0.798395000	-2.781452000
H	0.608662000	1.152307000	-2.673025000
C	-2.712956000	0.247437000	-0.956030000
H	-3.011607000	1.235025000	-1.286887000
C	-2.614074000	-2.746918000	-0.078648000
H	-2.727295000	-3.817082000	0.071608000
C	2.047839000	-1.141931000	-0.842667000
H	1.076057000	-1.553020000	-1.088629000
C	3.387599000	0.691632000	-0.041405000
H	3.465658000	1.705296000	0.341634000
C	-3.509104000	-0.781653000	-1.315740000
H	-4.349635000	-0.500217000	-1.945903000
C	-3.440826000	-2.175673000	-0.994700000
H	-4.152876000	-2.819123000	-1.499743000

C	3.205514000	-1.890891000	-1.051779000
H	3.129767000	-2.895256000	-1.453957000
C	4.544932000	-0.055690000	-0.248196000
H	5.513981000	0.376389000	-0.024512000
C	4.455929000	-1.352482000	-0.753233000
H	5.355129000	-1.934564000	-0.920899000
H	0.949217000	-2.202046000	2.268197000
C	-0.660293000	2.823669000	-2.148523000
H	-0.670951000	3.214501000	-3.170390000
H	-1.629695000	3.034159000	-1.693063000
H	0.095002000	3.386490000	-1.593469000

Ts-3

Number of imaginary frequencies: 1

Zero-point correction= 0.353569 (Hartree/Particle)

Thermal correction to Energy= 0.372845

Thermal correction to Enthalpy= 0.373789

Thermal correction to Gibbs free energy= 0.305544

Sum of electronic and zero-point Energies= -995.866118

Sum of electronic and thermal Energies= -995.846842

Sum of electronic and thermal Enthalpies= -995.845897

Sum of electronic and thermal Free Energies= -995.914143

Cartesian Coordinates

N	-0.240099000	0.583460000	-0.920405000
O	-0.774205000	2.813174000	2.277039000
N	-0.128231000	-0.468402000	1.639966000
O	-1.765288000	1.514985000	0.736761000
C	-1.443508000	0.292336000	-0.182477000
C	-1.139125000	-0.850612000	0.781633000
C	0.404267000	0.867342000	1.388558000
H	1.197925000	1.096068000	2.094639000
C	2.150374000	0.106126000	-0.320235000
C	0.905250000	0.949002000	-0.075557000
H	1.176362000	1.999486000	-0.245702000
C	-1.668214000	-2.113335000	0.780904000
H	-1.221791000	-2.798165000	1.492297000
C	0.145195000	-1.064578000	2.940328000
H	-0.262803000	-0.446230000	3.749487000
H	1.223670000	-1.167521000	3.082561000
C	-0.749338000	1.861096000	1.540067000
C	-0.294540000	1.227136000	-2.238153000
H	-1.042152000	0.697986000	-2.831671000
H	0.671028000	1.028544000	-2.713815000

C	-2.684467000	0.241556000	-1.008257000
H	-2.961703000	1.227776000	-1.361120000
C	-2.664612000	-2.717444000	-0.041234000
H	-2.800075000	-3.779988000	0.140960000
C	2.069683000	-1.210001000	-0.780381000
H	1.098280000	-1.630465000	-1.010851000
C	3.409634000	0.652673000	-0.050228000
H	3.487818000	1.680694000	0.292456000
C	-3.500496000	-0.777624000	-1.351113000
H	-4.328418000	-0.494028000	-1.996711000
C	-3.465446000	-2.161501000	-0.989241000
H	-4.181917000	-2.807260000	-1.484965000
C	3.226925000	-1.967346000	-0.957566000
H	3.150971000	-2.987219000	-1.318517000
C	4.566938000	-0.103226000	-0.224607000
H	5.535917000	0.336544000	-0.016102000
C	4.477458000	-1.418668000	-0.678185000
H	5.376316000	-2.007942000	-0.820857000
H	-0.300987000	-2.052059000	3.012340000
C	-0.577128000	2.736545000	-2.262536000
H	-0.571943000	3.091604000	-3.297405000

H -1.547302000 2.978014000 -1.824406000

H 0.181950000 3.306613000 -1.720259000

Pdt

Number of imaginary frequencies: 0

Zero-point correction= 0.354657 (Hartree/Particle)

Thermal correction to Energy= 0.375081

Thermal correction to Enthalpy= 0.376025

Thermal correction to Gibbs free energy= 0.304314

Sum of electronic and zero-point Energies= -995.883121

Sum of electronic and thermal Energies= -995.862697

Sum of electronic and thermal Enthalpies= -995.861753

Sum of electronic and thermal Free Energies= -995.933465

Cartesian Coordinates

N 0.113515000 0.528083000 1.066143000

O -0.136346000 3.334776000 -0.858213000

N 0.373037000 -0.180929000 -1.595208000

O 1.593315000 2.227105000 -1.842483000

C 1.218915000 -0.133584000 0.661943000

C 1.231241000 -0.693049000 -0.702915000

C -0.404050000 1.000650000 -1.251669000

H -1.218676000 1.091978000 -1.968904000

C	-2.145550000	-0.154904000	0.239727000
C	-0.990005000	0.833760000	0.143487000
H	-1.349185000	1.831114000	0.403849000
C	2.032193000	-1.801560000	-1.079798000
H	1.732015000	-2.259217000	-2.012662000
C	0.465052000	-0.522332000	-3.014982000
H	1.495429000	-0.425076000	-3.362097000
H	-0.147765000	0.180556000	-3.572369000
C	0.473557000	2.361682000	-1.333053000
C	-0.049399000	1.085065000	2.420714000
H	0.415691000	0.409796000	3.141484000
H	-1.121976000	1.062592000	2.624424000
C	2.329552000	-0.214753000	1.529222000
H	2.285105000	0.464003000	2.368136000
C	3.140876000	-2.390739000	-0.497841000
H	3.540127000	-3.237926000	-1.047843000
C	-1.988410000	-1.473842000	0.672844000
H	-1.016931000	-1.827203000	0.998558000
C	-3.420745000	0.277112000	-0.147310000
H	-3.561324000	1.304004000	-0.470672000
C	3.477238000	-1.002219000	1.512085000

H	4.160849000	-0.807619000	2.333333000
C	3.846548000	-2.017257000	0.644329000
H	4.757666000	-2.560183000	0.867678000
C	-3.077412000	-2.345904000	0.705493000
H	-2.937809000	-3.365781000	1.046655000
C	-4.507316000	-0.592058000	-0.117857000
H	-5.487340000	-0.238600000	-0.418349000
C	-4.338531000	-1.909965000	0.307746000
H	-5.184673000	-2.586988000	0.336181000
H	0.096608000	-1.535462000	-3.203648000
C	0.480379000	2.516806000	2.553788000
H	0.219630000	2.910223000	3.540004000
H	1.565680000	2.562540000	2.442804000
H	0.050480000	3.164663000	1.786733000
