# Supplementary material for: One-pot synthesis of cyclic-aminotropiminium carboxylate derivatives with DNA binding and anticancer properties
Source: Commun Chem. 2022 Dec 27;5:179. doi: 10.1038/s42004-022-00798-x (PMC9814901; doi:10.1038/s42004-022-00798-x)
Supplement: Supplementary file 10 — Supplementary Data 7 [file 42004_2022_798_MOESM10_ESM.pdf]

Supplemental Material

One-Pot synthesis of cyclic-aminotropiminium carboxylate derivatives with DNA binding and anticancer properties

Bibhuti Bhusana Palai[a,c,#], Saket Awadhesbhai Patel[b,c,#], Nagendra K. Sharma*[,a.c], Manjusha Dixit*[,b,c]

[#]Authors equally contributed

*Corresponding Authors

[a]School of Chemical Sciences, National Institute of Science Education and Research (NISER) Bhubaneswar, PO: Jatani-752050, Odisha, India, Phone no. +91-674-249-4141; E-mail: nagendra@niser.ac.in

[b]School of Biological Sciences, National Institute of Science Education and Research (NISER) Bhubaneswar, PO: Jatani-752050, Odisha, India, Phone no. +91-674-249-4195; E-mail: manjusha@niser.ac.in

[c]Homi Bhabha National Institute, Training School Complex, Anushaktinagar, Mumbai 400094, India

Western blot uncropped images of all 3 rounds

- HeLa cells were treated with 6e compounds

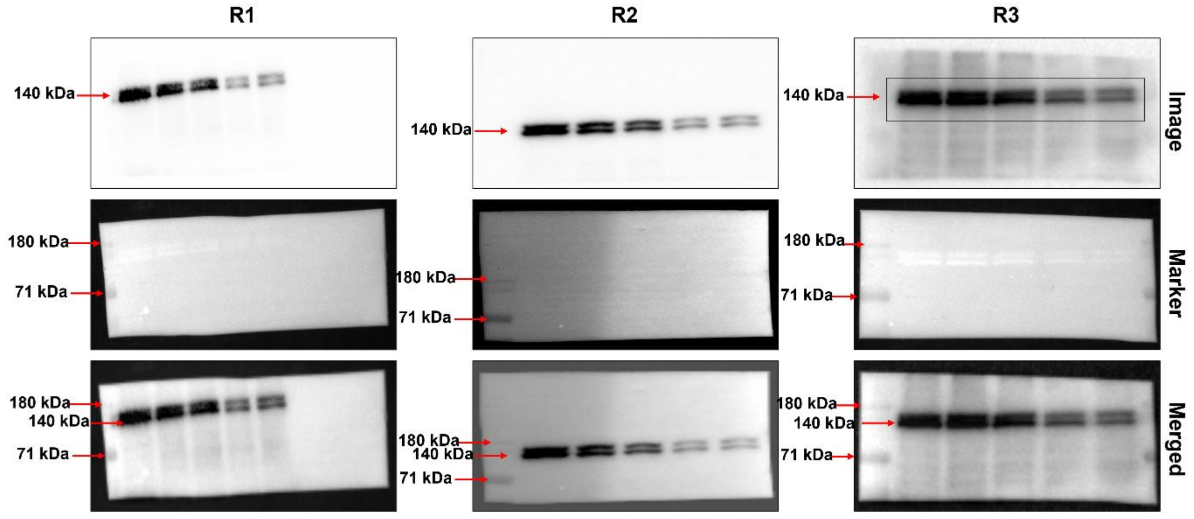

N-Cadherin

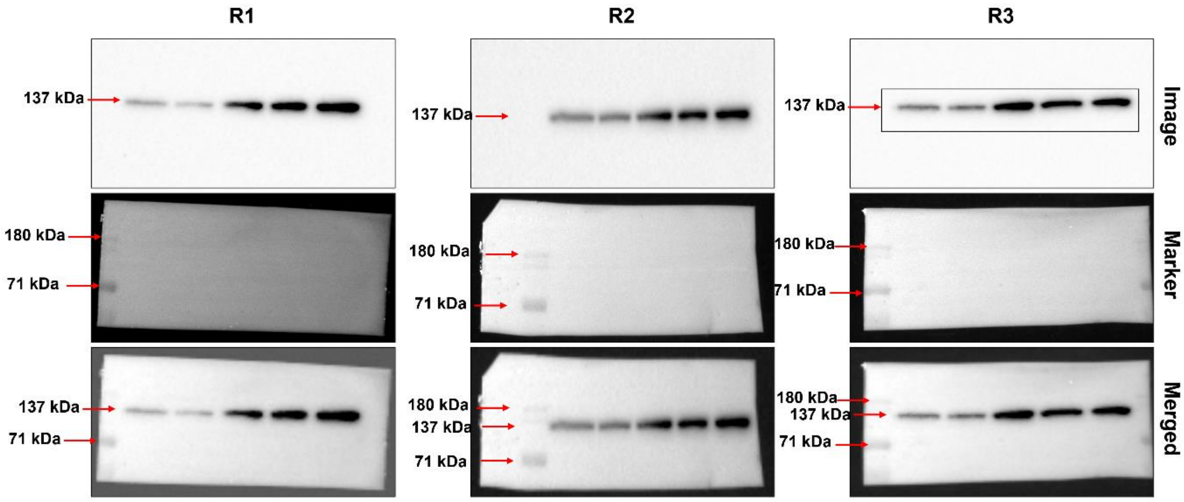

E-Cadherin

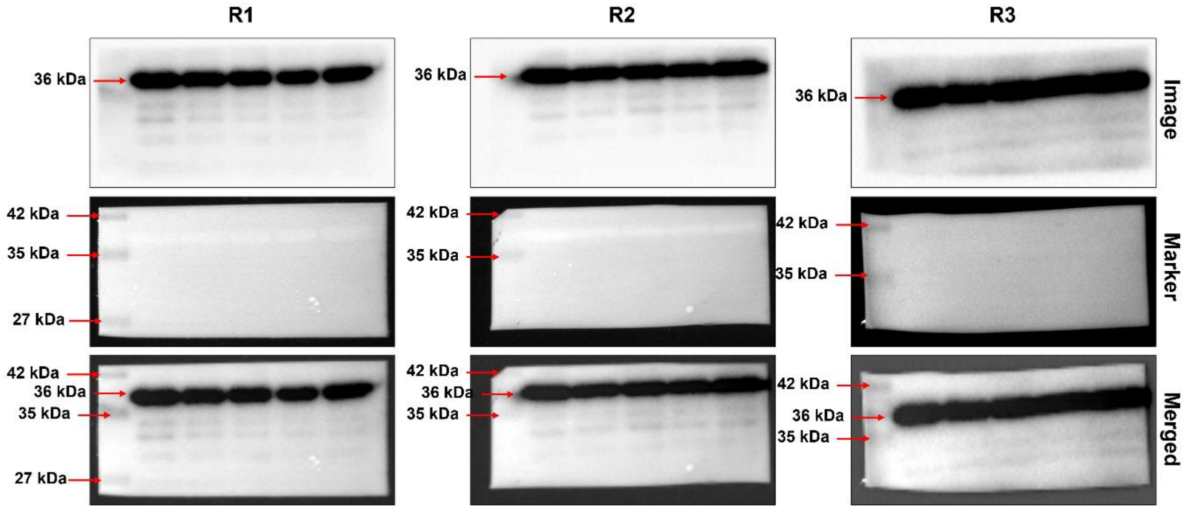

GAPDH

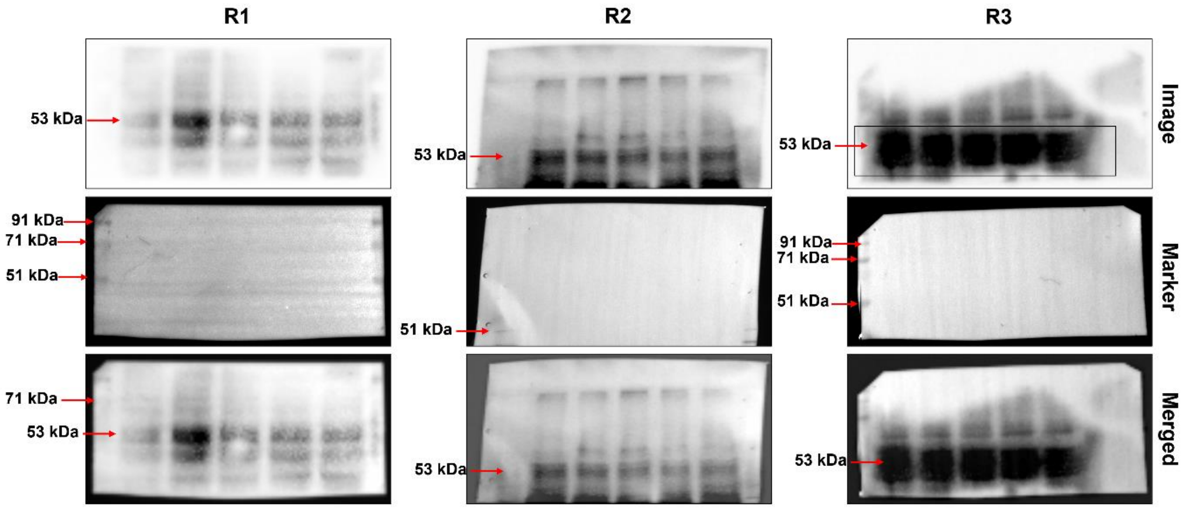
R1
R2
R3
Image
Marker
Merged
53 kDa
91 kDa
71 kDa
51 kDa

P53

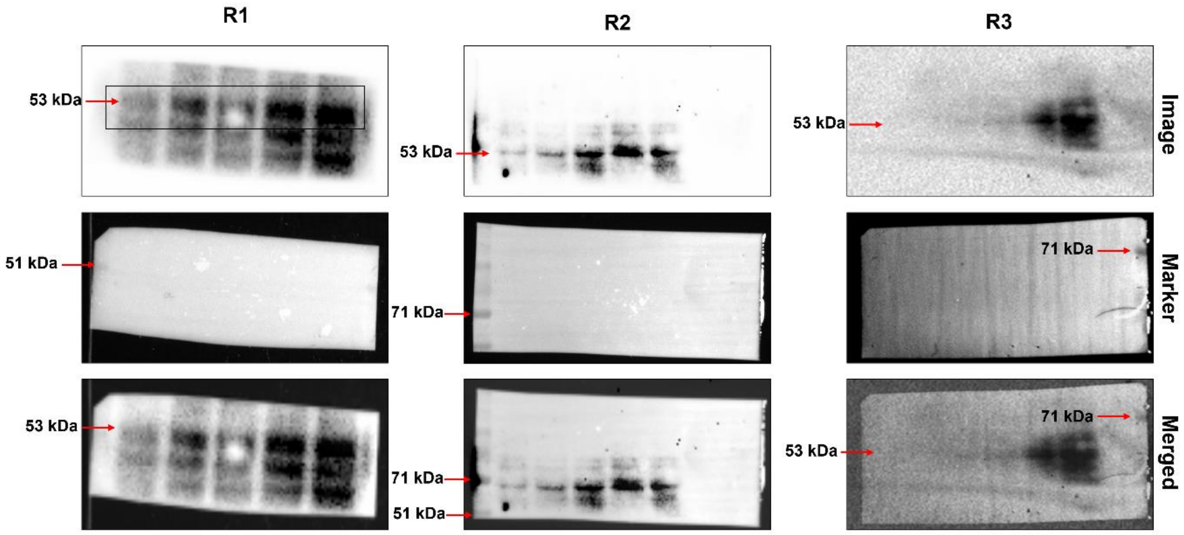
R1
R2
R3
Image
Marker
Merged
53 kDa
51 kDa
71 kDa

pP53

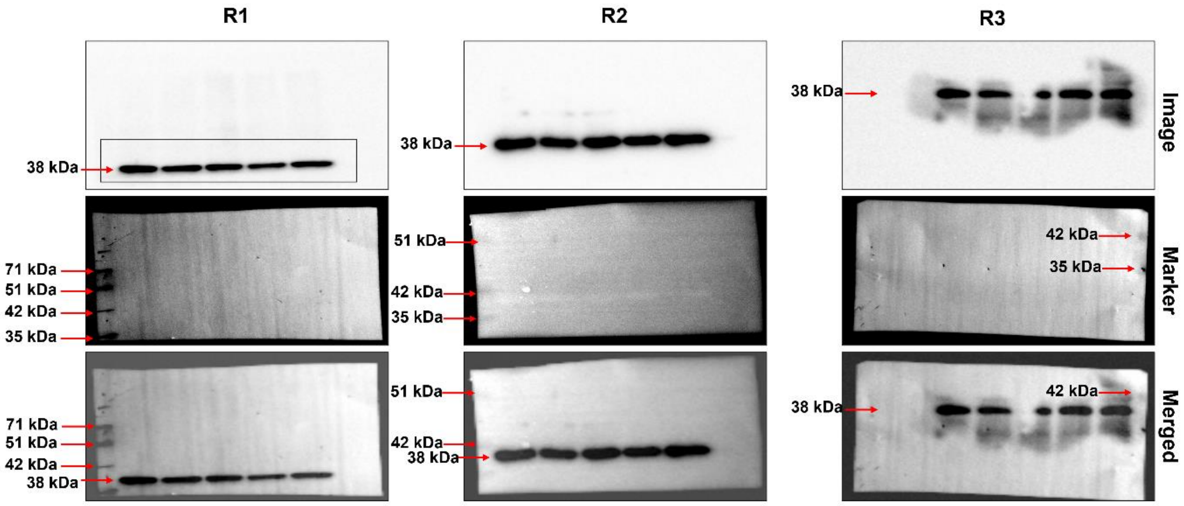

P38

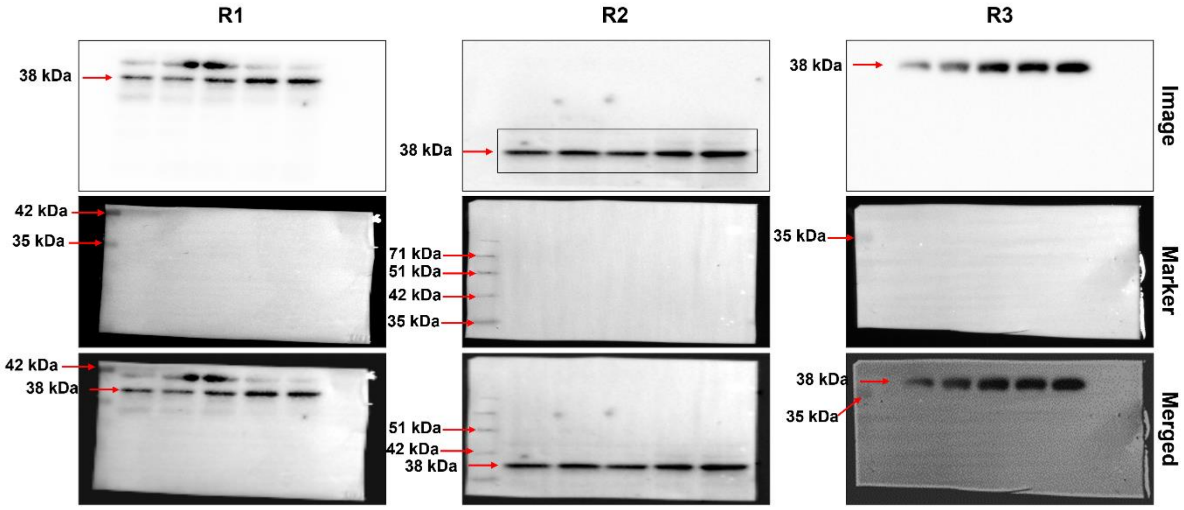

pP38

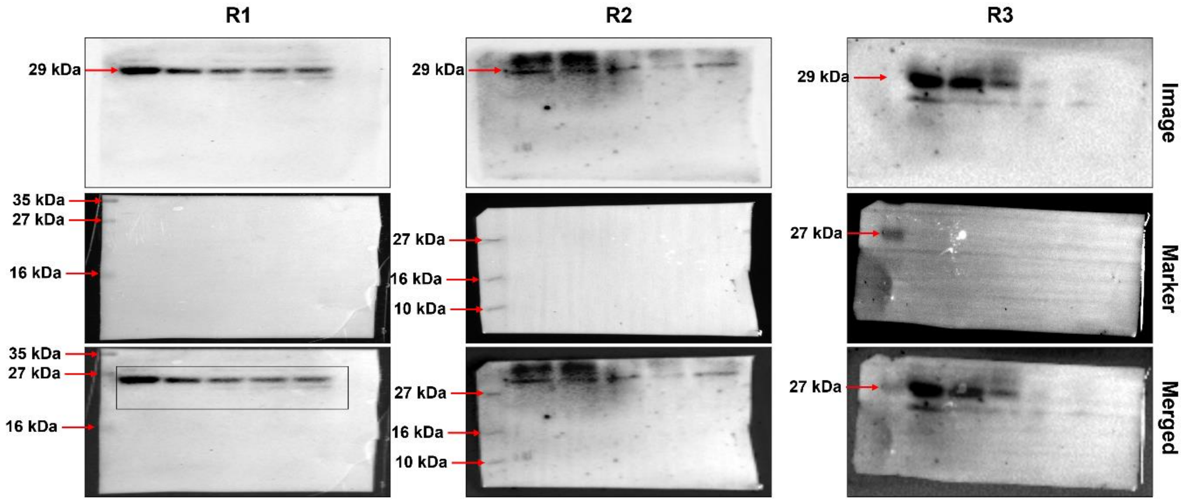

SNAIL

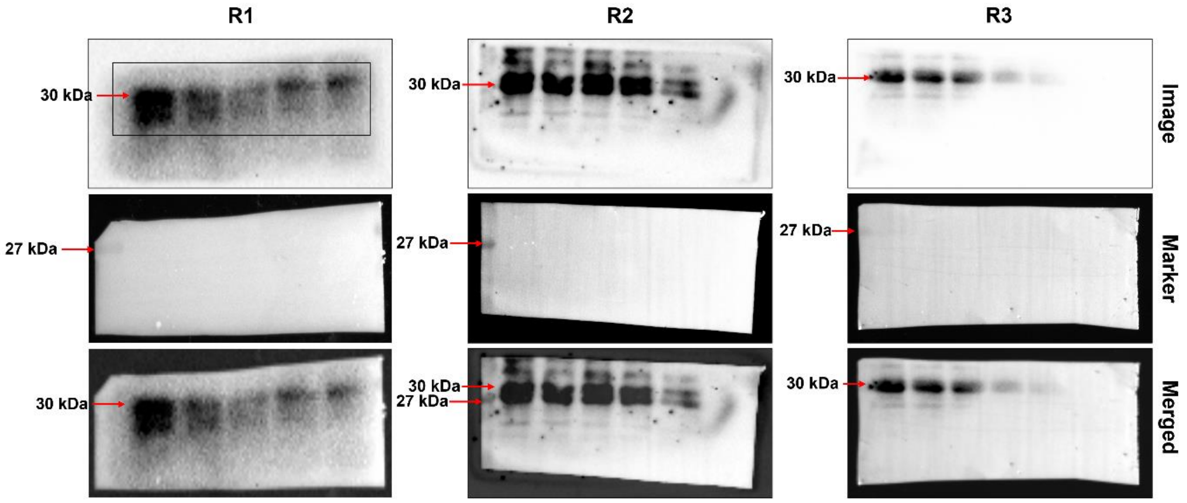

SLUG

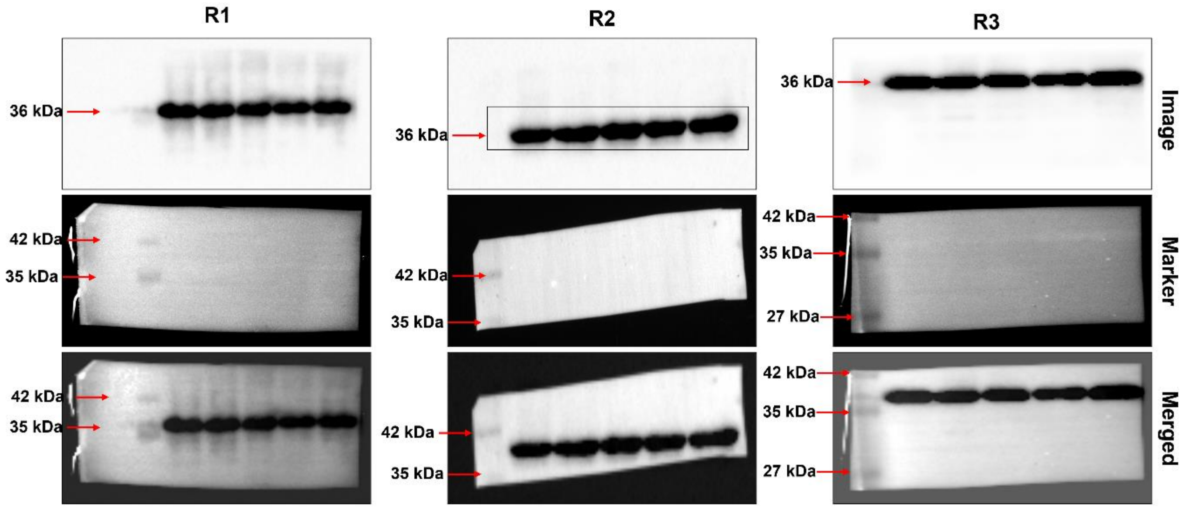

GAPDH

Above figures shows the uncropped images of three rounds treated with 6e. Rectangular bar used in the figures indicate that specific image used in the Main file of manuscript in a cropped format.

- **HeLa cells were treated with 6j compounds**

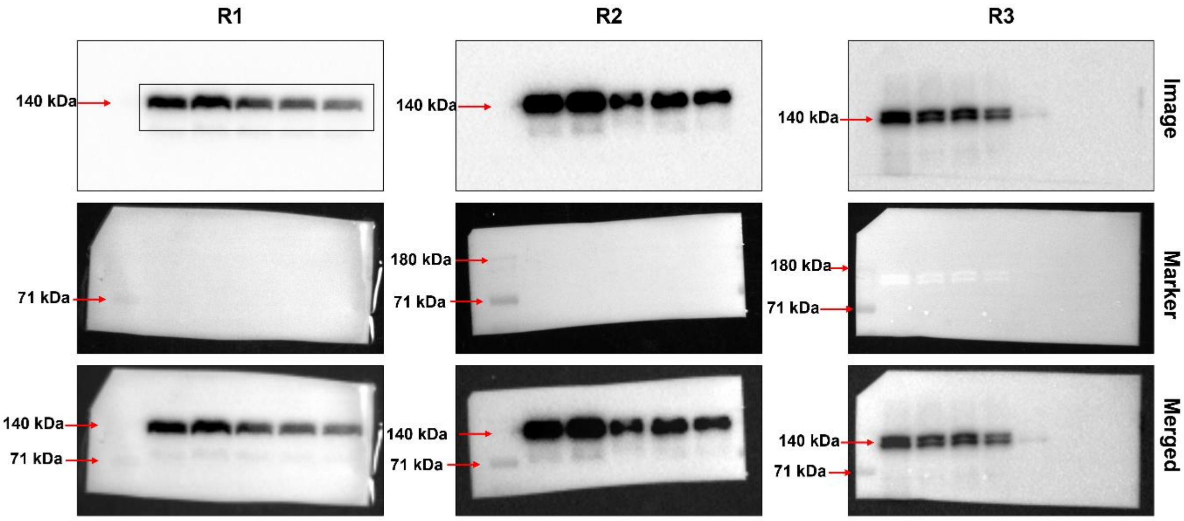

N-Cadherin

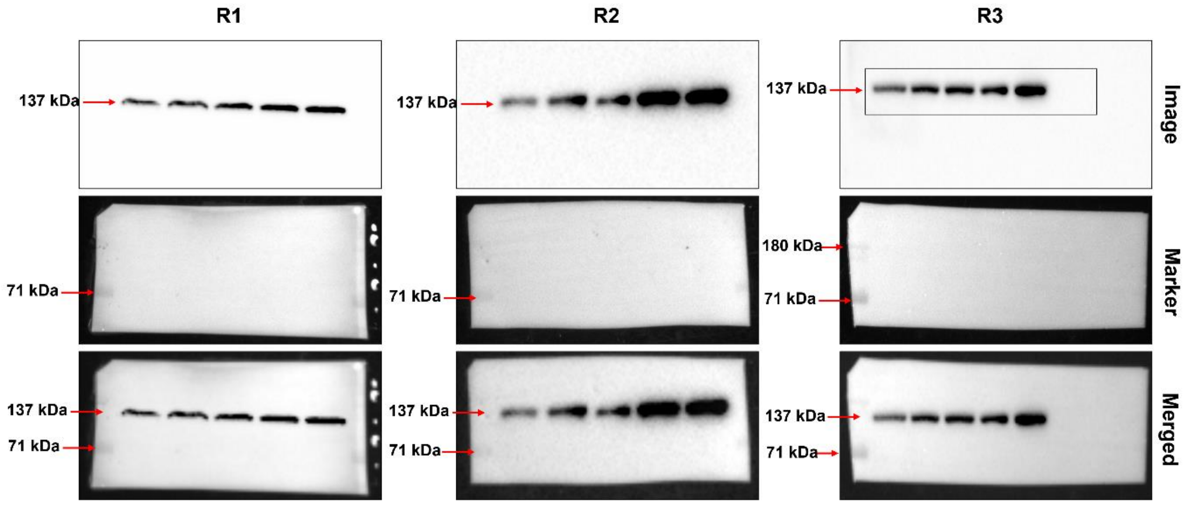

E-Cadherin

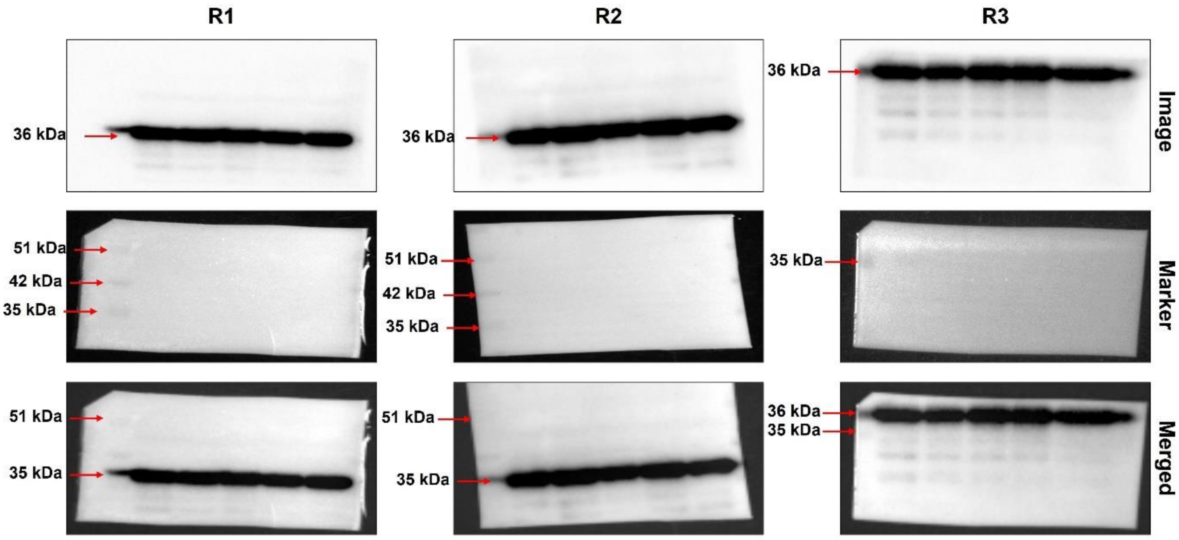
R1
R2
R3
Image
Marker
Merged
36 kDa
51 kDa
42 kDa
35 kDa
36 kDa
35 kDa

GAPDH

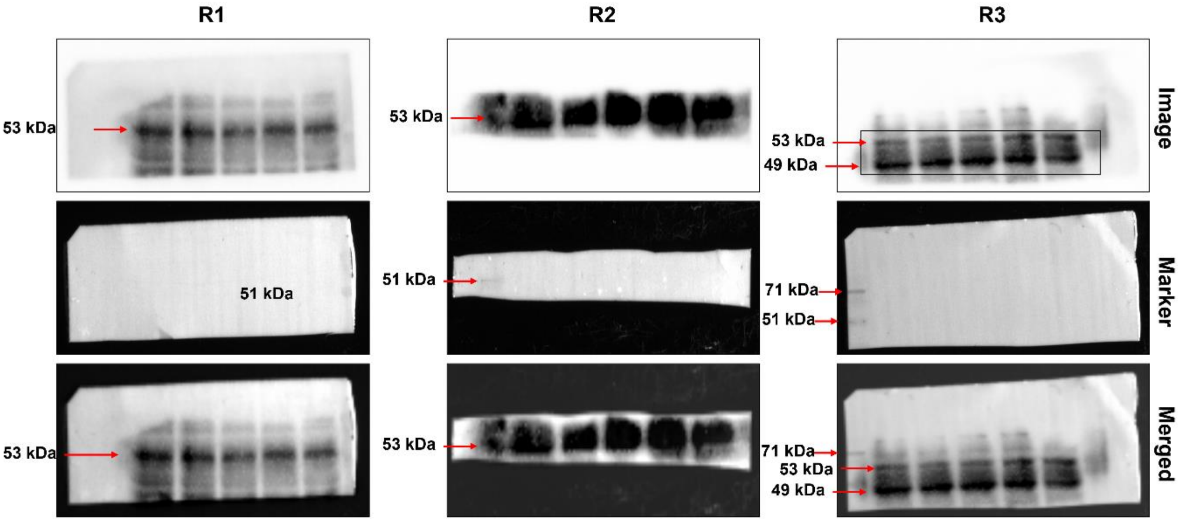
R1
R2
R3
Image
Marker
Merged
53 kDa
49 kDa
51 kDa
71 kDa

P53

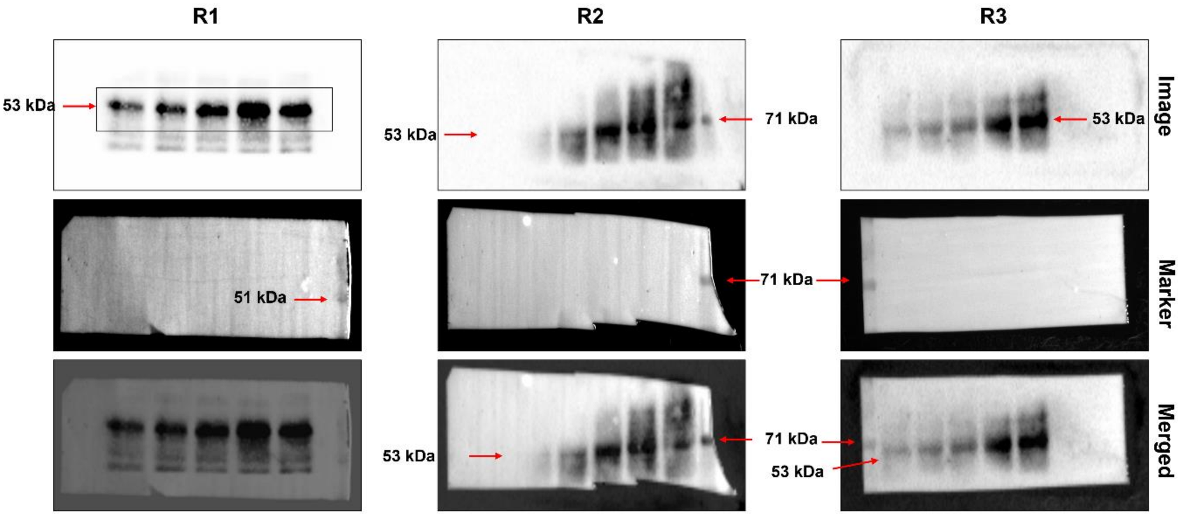
R1
R2
R3
53 kDa
53 kDa
71 kDa
53 kDa
Image
51 kDa
71 kDa
Marker
53 kDa
71 kDa
53 kDa
Merged

pP53

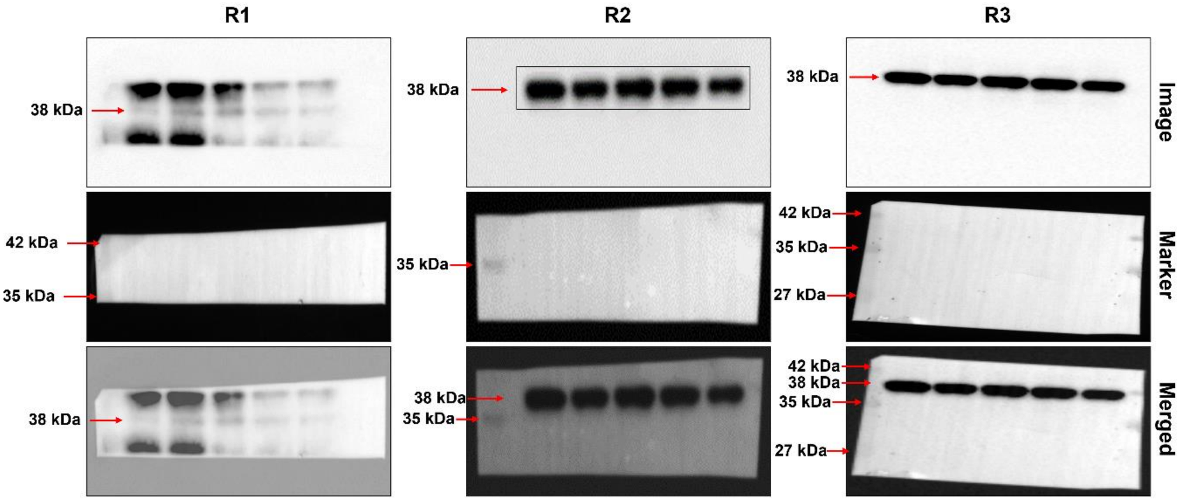
R1
R2
R3
38 kDa
38 kDa
38 kDa
Image
42 kDa
35 kDa
35 kDa
42 kDa
35 kDa
27 kDa
Marker
38 kDa
38 kDa
35 kDa
42 kDa
38 kDa
35 kDa
27 kDa
Merged

P38

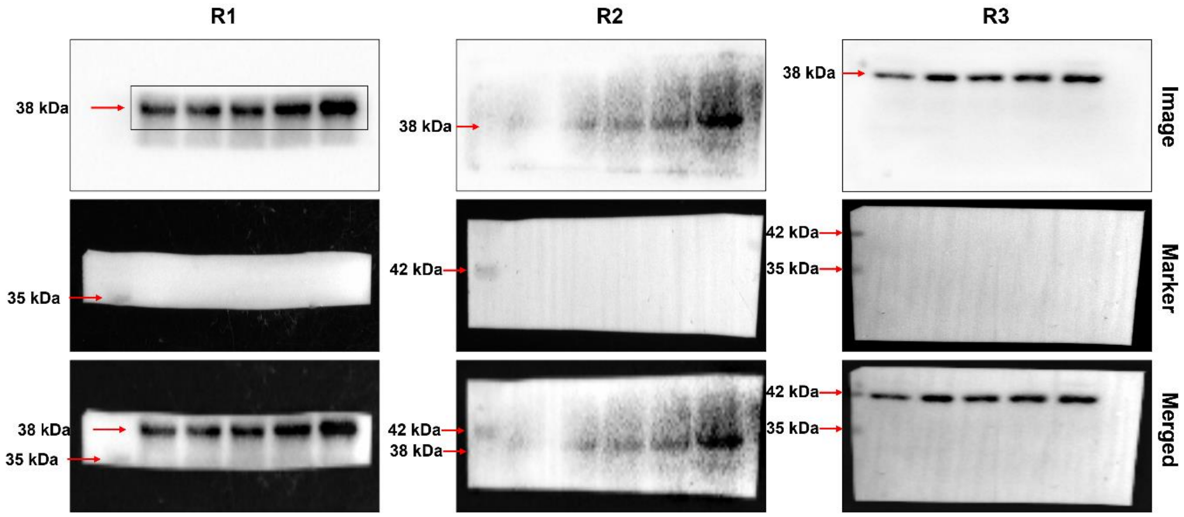
R1
R2
R3
38 kDa
38 kDa
38 kDa
Image
35 kDa
42 kDa
42 kDa
35 kDa
Marker
38 kDa
35 kDa
42 kDa
38 kDa
42 kDa
35 kDa
Merged

pP38

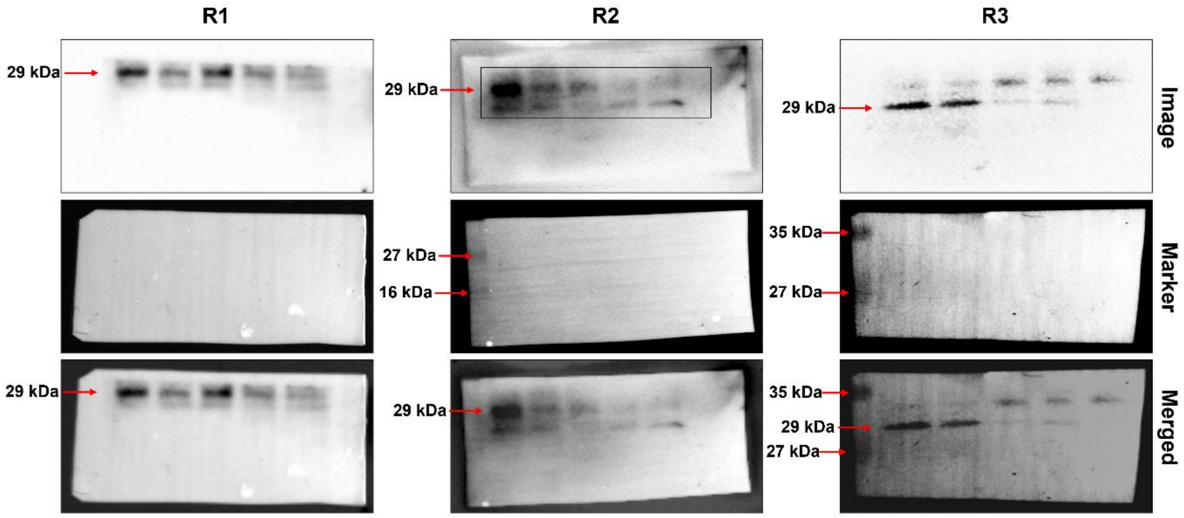
R1
R2
R3
29 kDa
29 kDa
29 kDa
Image
27 kDa
16 kDa
35 kDa
27 kDa
Marker
29 kDa
29 kDa
35 kDa
29 kDa
27 kDa
Merged

SNAIL

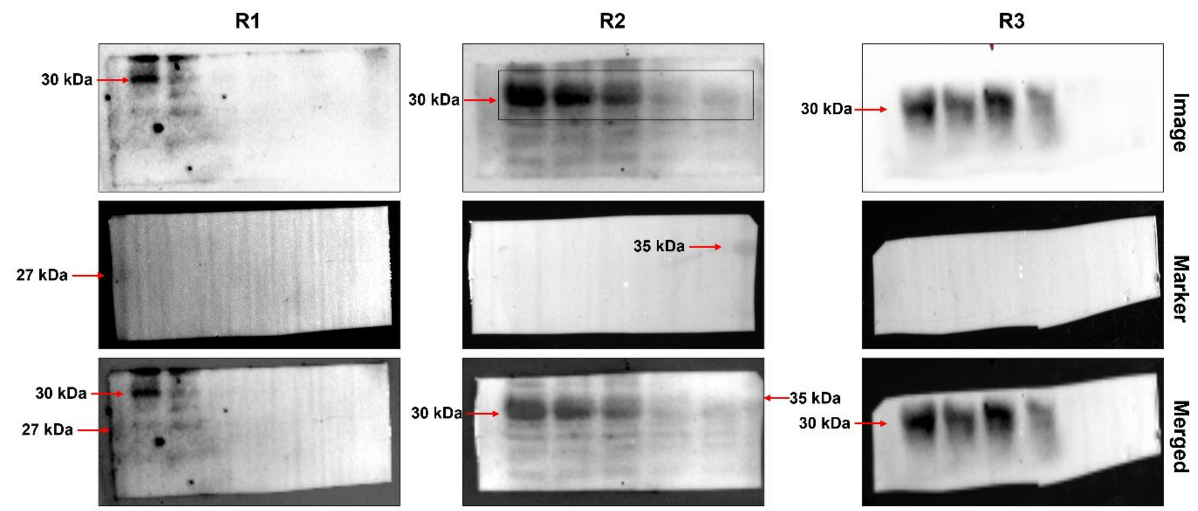

SLUG

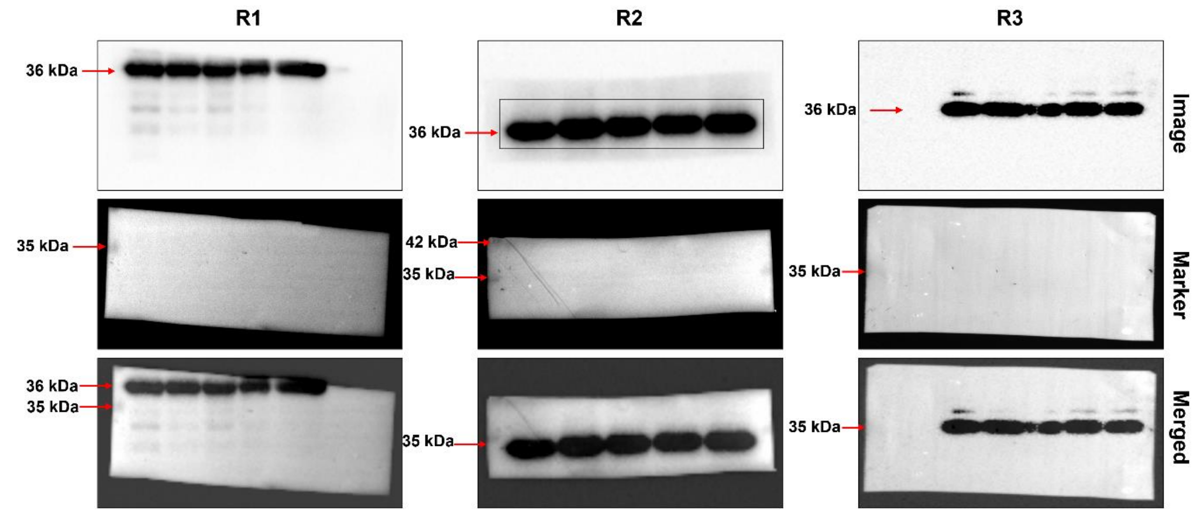

GAPDH

Above figures shows the uncropped images of three rounds treated with 6j. Rectangular bar used in the figures indicate that specific image used in the Main file of manuscript in a cropped format.

- **HeLa cells were treated with 6n compounds**

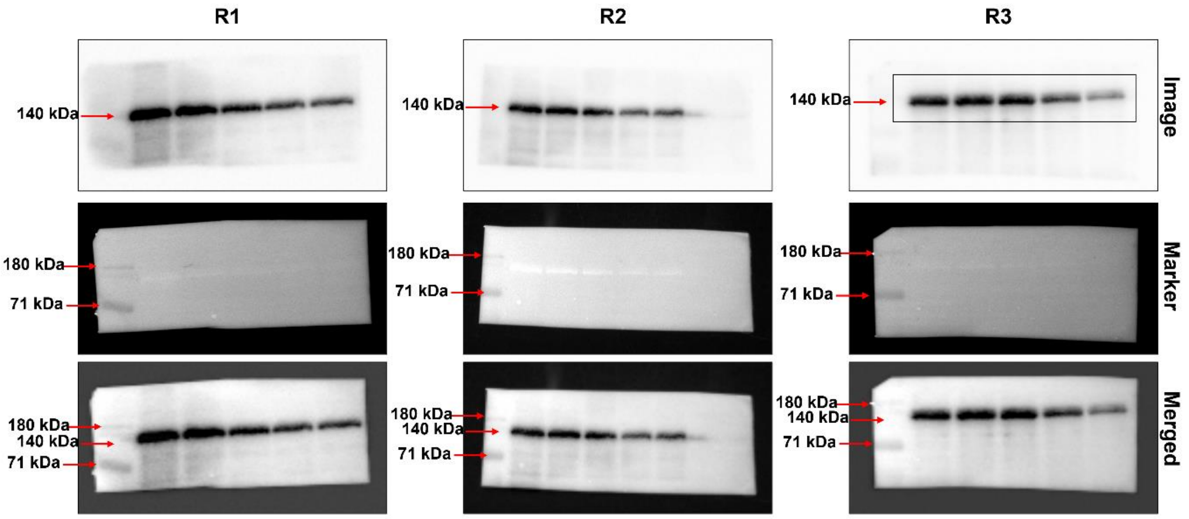

N-Cadherin

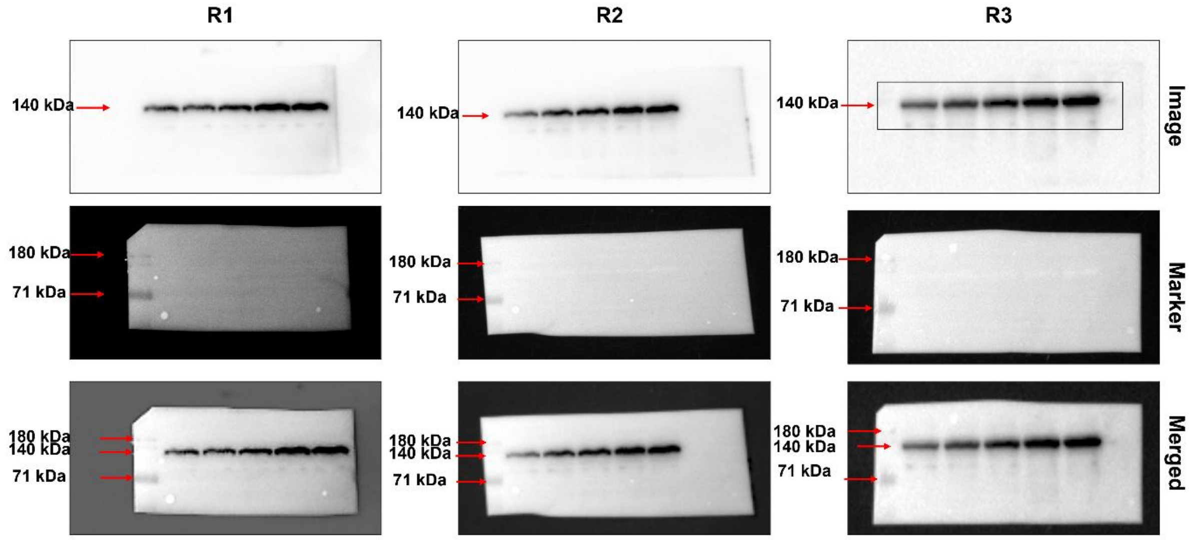

E-Cadherin

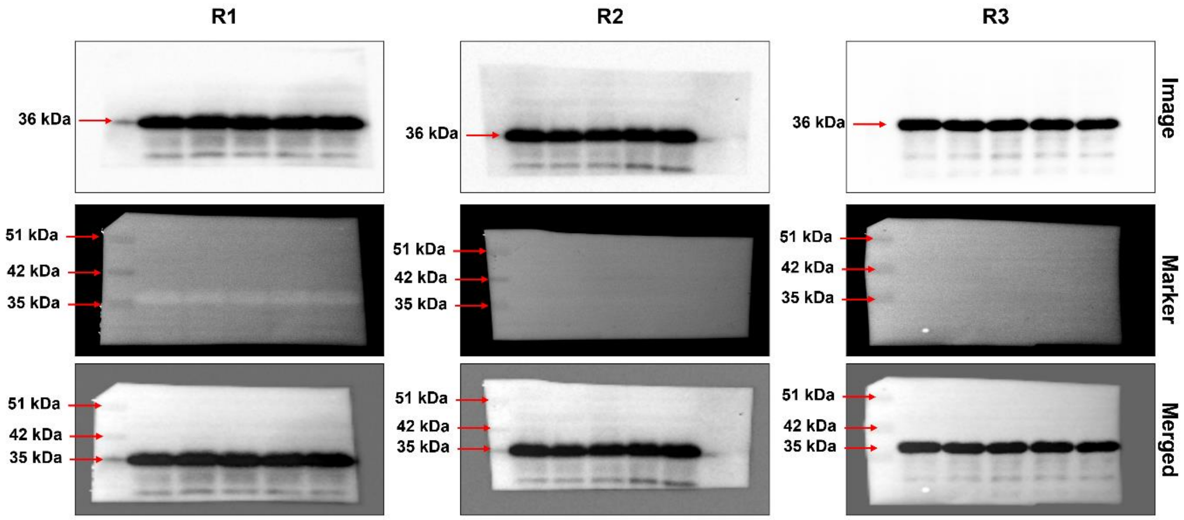
R1
R2
R3
Image
Marker
Merged
36 kDa
51 kDa
42 kDa
35 kDa

GAPDH

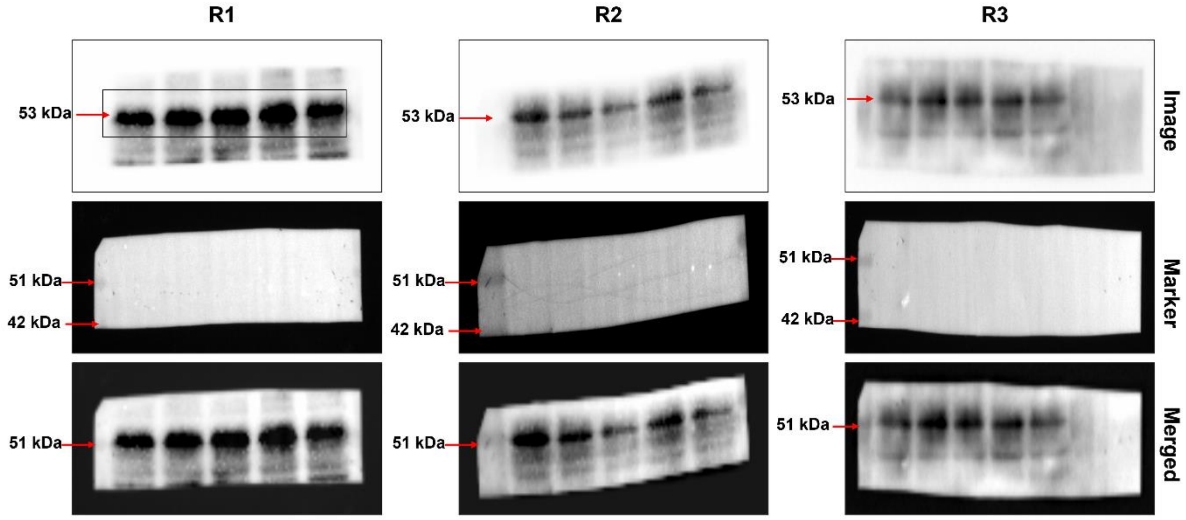
R1
R2
R3
Image
Marker
Merged
53 kDa
51 kDa
42 kDa

P53

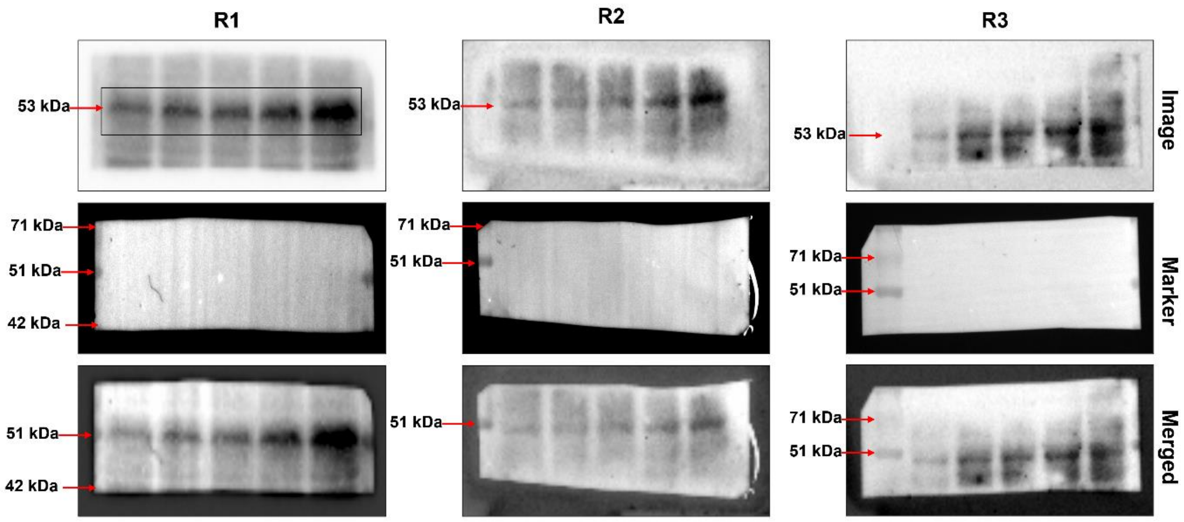
R1
R2
R3
53 kDa
53 kDa
53 kDa
Image
71 kDa
51 kDa
42 kDa
71 kDa
51 kDa
71 kDa
51 kDa
Marker
51 kDa
42 kDa
51 kDa
71 kDa
51 kDa
Merged

pP53

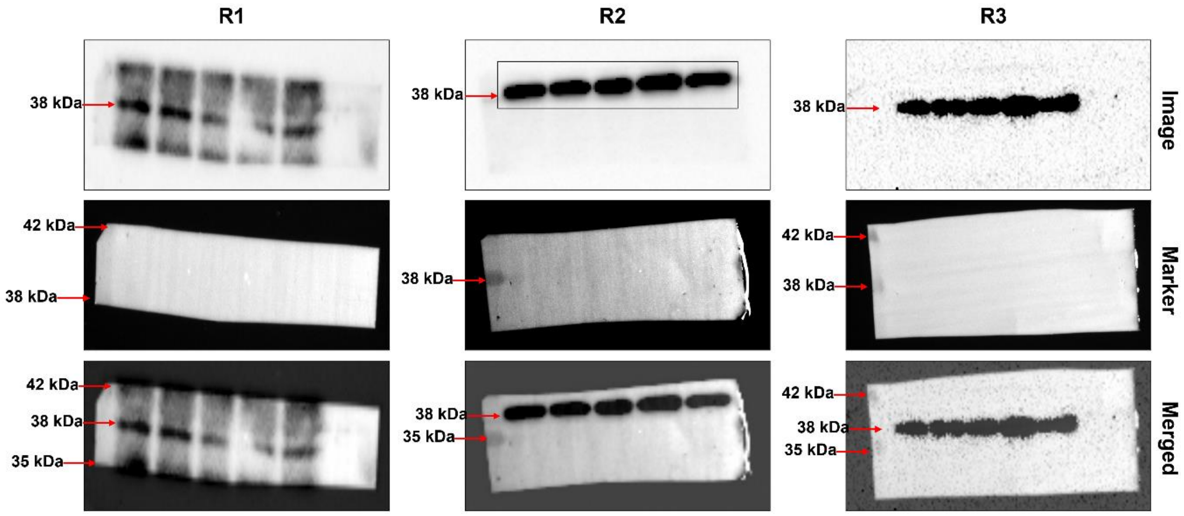
R1
R2
R3
38 kDa
38 kDa
38 kDa
Image
42 kDa
38 kDa
38 kDa
42 kDa
38 kDa
Marker
42 kDa
38 kDa
35 kDa
38 kDa
35 kDa
42 kDa
38 kDa
35 kDa
Merged

P38

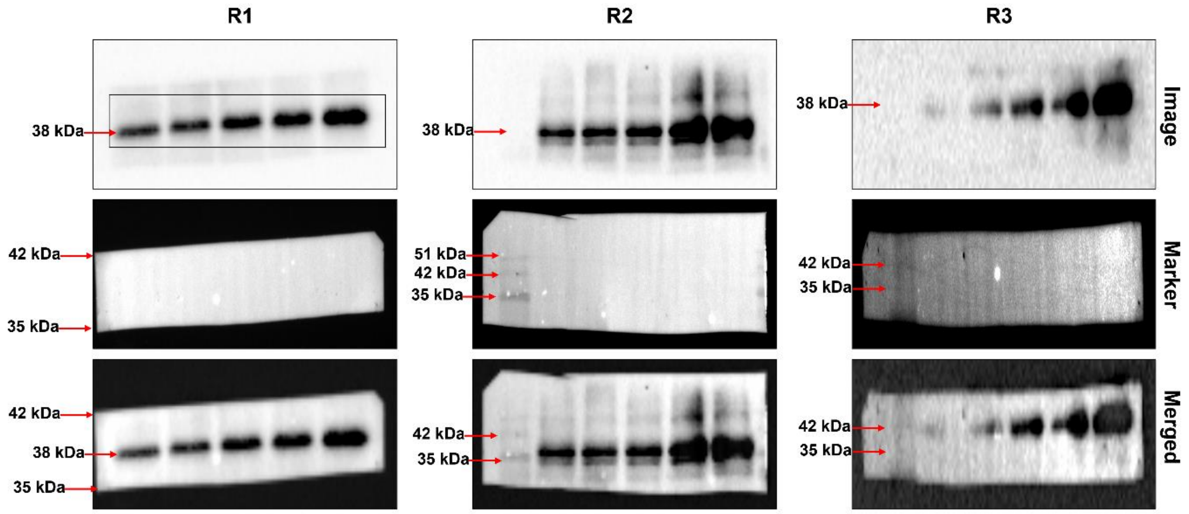
R1
R2
R3
38 kDa
38 kDa
38 kDa
Image
42 kDa
35 kDa
51 kDa
42 kDa
35 kDa
42 kDa
35 kDa
Marker
42 kDa
38 kDa
35 kDa
42 kDa
35 kDa
42 kDa
35 kDa
Merged

pP38

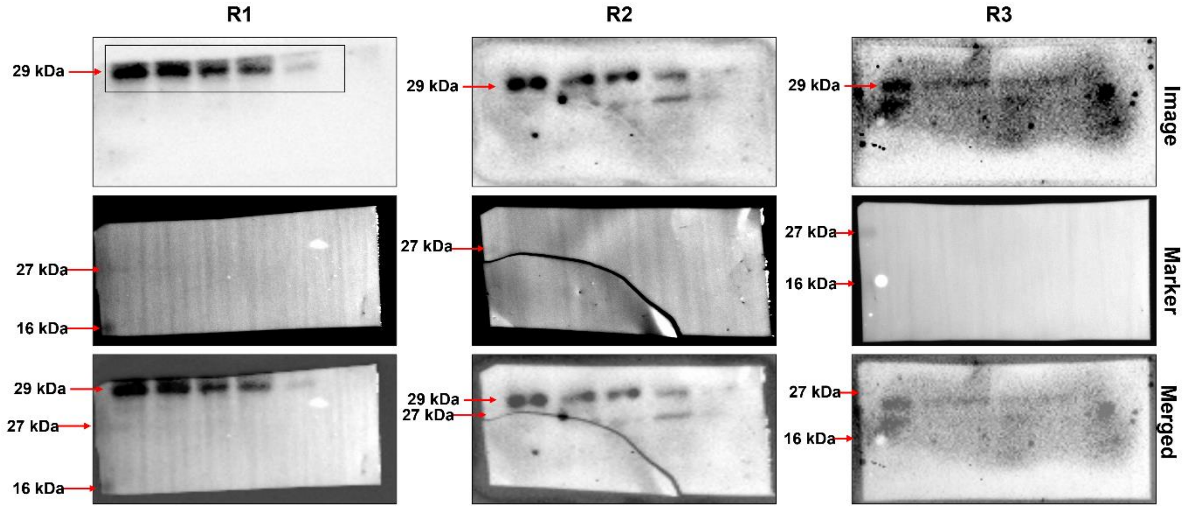
R1
R2
R3
29 kDa
29 kDa
29 kDa
Image
27 kDa
16 kDa
27 kDa
27 kDa
16 kDa
Marker
29 kDa
27 kDa
16 kDa
29 kDa
27 kDa
27 kDa
16 kDa
Merged

SNAIL

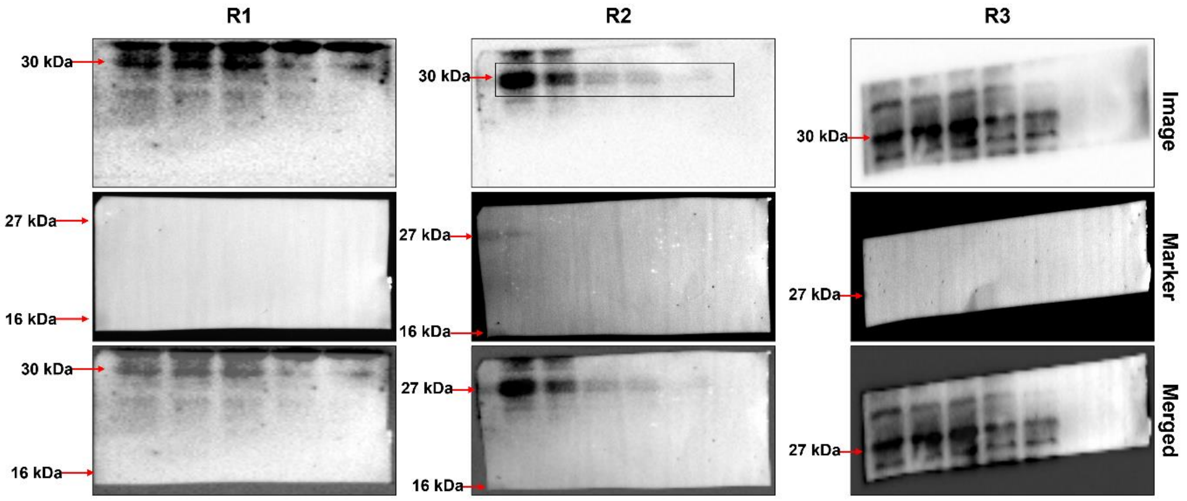

SLUG

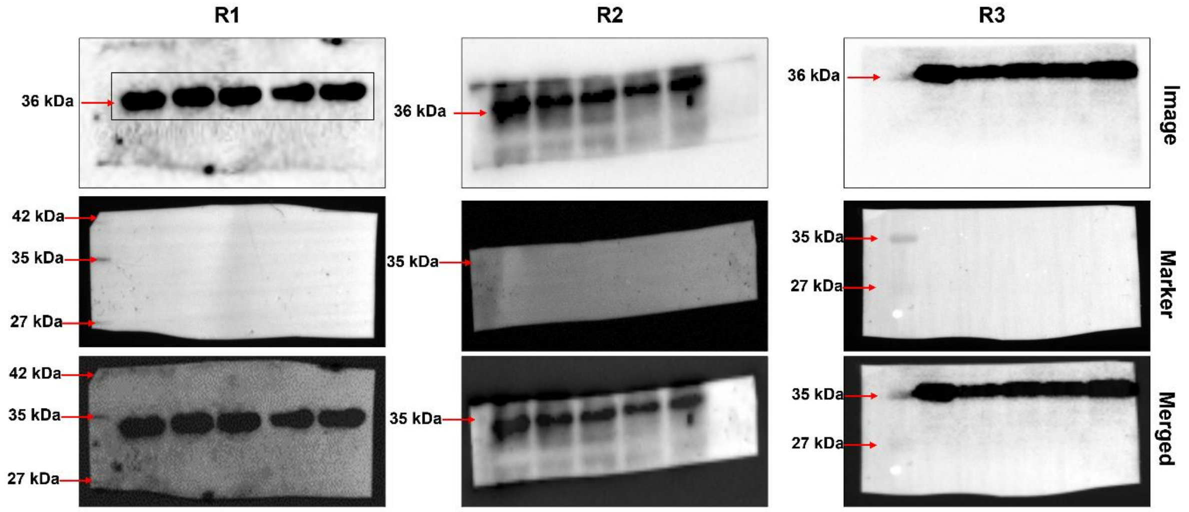

GAPDH

Above figures shows the uncropped images of three rounds treated with 6n. Rectangular bar used in the figures indicate that specific image used in the Main file of manuscript in a cropped format.
